# Childhood growth during recovery from acute illness in Africa and South Asia: a secondary analysis of the childhood acute illness and nutrition (CHAIN) prospective cohort

**DOI:** 10.1016/j.eclinm.2024.102530

**Published:** 2024-03-12

**Authors:** Celine Bourdon, Abdoulaye Hama Diallo, Abu Sadat Mohammad Sayeem Bin Shahid, Md Alfazal Khan, Ali Faisal Saleem, Benson O. Singa, Blaise Siézanga Gnoumou, Caroline Tigoi, Catherine Achieng Otieno, Chrisantus Odhiambo Oduol, Christina L. Lancioni, Christine Manyasi, Christine J. McGrath, Christopher Maronga, Christopher Lwanga, Daniella Brals, Dilruba Ahmed, Dinesh Mondal, Donna M. Denno, Dorothy I. Mangale, Emmanuel Chimwezi, Emmie Mbale, Ezekiel Mupere, Gazi Md Salauddin Mamun, Issaka Ouédraogo, James A. Berkley, James M. Njunge, Jenala Njirammadzi, John Mukisa, Johnstone Thitiri, Judd L. Walson, Julie Jemutai, Kirkby D. Tickell, Lubaba Shahrin, Macpherson Mallewa, Md Iqbal Hossain, Mohammod Jobayer Chisti, Molline Timbwa, Moses Mburu, Moses M. Ngari, Narshion Ngao, Peace Aber, Philliness Prisca Harawa, Priya Sukhtankar, Robert H.J. Bandsma, Roseline Maïmouna Bamouni, Sassy Molyneux, Shalton Mwaringa, Shamsun Nahar Shaima, Syed Asad Ali, Syeda Momena Afsana, Sayera Banu, Tahmeed Ahmed, Wieger P. Voskuijl, Zaubina Kazi

**Affiliations:** a Translational Medicine, Hospital for Sick Children, Toronto, ON, Canada; b Department of Public Health, University Joseph Ki-Zerbo, Ouagadougou, Burkina Faso; c Department of Public Health, Centre Muraz Research Institute, Bobo-Dioulasso, Burkina Faso; d Nutrition Research Division, International Centre for Diarrhoeal Disease Research, Bangladesh (icddr,b), Dhaka, Bangladesh; e Health System and Population Studies Division, International Centre for Diarrhoeal Disease Research, Bangladesh (icddr,b), Dhaka, Bangladesh; f Department of Pediatrics and Child Health, Aga Khan University, Karachi, Pakistan; g Kenya Medical Research Institute, Nairobi, Kenya; h Clinical Research Department, KEMRI–Wellcome Trust Research Programme, Kilifi, Kenya; i Centre for Tropical Medicine and Global Health, Nuffield Department of Medicine, University of Oxford, Oxford, United Kingdom; j Department of Pediatrics, Oregon Health and Science University, Portland, OR, USA; k Department of Paediatrics, Mbagathi Hospital, Nairobi, Kenya; l Department of Global Health, University of Washington, Seattle, WA, USA; m Department of Epidemiology, University of Washington, Seattle, WA, USA; n Uganda-Case Western Reserve University Research Collaboration, Kampala, Uganda; o Department of Global Health, Amsterdam UMC, University of Amsterdam, Amsterdam, Netherlands; p Clinical Microbiology and Immunology Laboratory, Office of Executive Director, International Centre for Diarrhoeal Disease Research, Bangladesh (icddr,b), Dhaka, Bangladesh; q Department of Pediatrics, University of Washington, Seattle, WA, USA; r Department of Paediatrics and Child Health, Kamuzu University of Health Sciences, Blantyre, Malawi; s Department of Paediatrics and Child Health, Makerere University College of Health Sciences, Kampala, Uganda; t Infectious Diseases Division, International Centre for Diarrhoeal Disease Research Bangladesh (icddr,b), Dhaka, Bangladesh; u Department of Pediatrics, Banfora Referral Regional Hospital, Banfora, Burkina Faso; v Department of Immunology and Department of Molecular Biology Makerere University College of Health Sciences, Kampala, Uganda; w Hospitals, Office of Executive Director, International Centre for Diarrhoeal Disease Research, Bangladesh (icddr,b), Dhaka, Bangladesh; x Department of Nutritional Sciences, Faculty of Medicine, University of Toronto, Toronto, ON, Canada; y Health Systems and Research Ethics Department, KEMRI–Wellcome Trust Research Programme, Kilifi, Kenya; z Clinical Biochemistry Laboratory, Office of Executive Director, International Centre for Diarrhoeal Disease Research, Bangladesh (icddr,b), Dhaka, Bangladesh; aa Office of Executive Director, International Centre for Diarrhoeal Disease Research, Bangladesh (icddr,b), Dhaka, Bangladesh; ab Amsterdam UMC, Location University of Amsterdam, Amsterdam Institute for Global Child Health, Emma Children’s Hospital, Meibergdreef 9, Amsterdam, the Netherlands; ac Amsterdam UMC, Location University of Amsterdam, Department of Global Health, Amsterdam Institute for Global Health and Development, Meibergdreef 9, Amsterdam, the Netherlands; ad Departments of International Health and Medicine, Bloomberg School of Public Health, Johns Hopkins University, Baltimore, MD, USA

**Keywords:** Growth, Weight, Length, Wasting, Stunting, Kwashiorkor, Children, “Acute illness”, Hospital, Post-discharge, Africa, “South asia”, Vulnerability, Malnutrition

## Abstract

**Background:**

Growth faltering is well-recognized during acute childhood illness and growth acceleration during convalescence, with or without nutritional therapy, may occur. However, there are limited recent data on growth after hospitalization in low- and middle-income countries.

**Methods:**

We evaluated growth following hospitalization among children aged 2–23 months in sub-Saharan Africa and South Asia. Between November 2016 and January 2019, children were recruited at hospital admission and classified as: not-wasted (NW), moderately-wasted (MW), severely-wasted (SW), or having nutritional oedema (NO). We describe earlier (discharge to 45-days) and later (45- to 180-days) changes in length-for-age [LAZ], weight-for-age [WAZ], mid-upper arm circumference [MUACZ], weight-for-length [WLZ] z-scores, and clinical, nutritional, and socioeconomic correlates.

**Findings:**

We included 2472 children who survived to 180-days post-discharge: NW, 960 (39%); MW, 572 (23%); SW, 682 (28%); and NO, 258 (10%). During 180-days, LAZ decreased in NW (−0.27 [−0.36, −0.19]) and MW (−0.23 [−0.34, −0.11]). However, all groups increased WAZ (NW, 0.21 [95% CI: 0.11, 0.32]; MW, 0.57 [0.44, 0.71]; SW, 1.0 [0.88, 1.1] and NO, 1.3 [1.1, 1.5]) with greatest gains in the first 45-days. Of children underweight (<−2 WAZ) at discharge, 66% remained underweight at 180-days. Lower WAZ post-discharge was associated with age-inappropriate nutrition, adverse caregiver characteristics, small size at birth, severe or moderate anaemia, and chronic conditions, while lower LAZ was additionally associated with household-level exposures but not with chronic medical conditions.

**Interpretation:**

Underweight and poor linear growth mostly persisted after an acute illness. Beyond short-term nutritional supplementation, improving linear growth post-discharge may require broader individual and family support.

**Funding:**

10.13039/100000865Bill & Melinda Gates FoundationOPP1131320; 10.13039/501100000272National Institute for Health ResearchNIHR201813.

**Research in context**

**Evidence before this study**

We searched PubMed for any published studies without date restriction using the following search terms: ((“infant” [MeSH Terms] OR “infant” [All Fields] OR “infants” [All Fields] OR “infant s” [All Fields] OR (“child” [MeSH Terms] OR “child” [All Fields] OR “children” [All Fields] OR “child s” [All Fields] OR “children s” [All Fields] OR “childrens” [All Fields] OR “childs” [All Fields]) AND ((“post-discharge growth” [All Fields] AND “hospital” [All Fields]) OR “post-discharge growth” [All Fields]) OR (“growth” [All Fields] AND “post-discharge” [All Fields]) OR (“growth” [All Fields] AND “after hospitalisation” [All Fields])) OR ((“wasting” [All Fields] AND “post-discharge” [All Fields]) OR (“wasting” [All Fields] AND “after hospitalisation” [All Fields])) OR ((“hospital” [All Fields] AND “discharge” [All Fields]) OR (“stunting” [All Fields] AND “post-discharge” [All Fields]) OR (“stunting” [All Fields] AND “after hospitalisation” [All Fields])) AND (“acute” [All Fields] OR “acutely” [All Fields] OR “acutes” [All Fields] OR (“inpatient s” [All Fields] OR “inpatients” [MeSH Terms] OR “inpatients” [All Fields] OR “inpatient” [All Fields]) OR “post-discharge” [All Fields])) AND (“africa” [MeSH Terms] OR “africa” [All Fields] OR “africa s” [All Fields] OR “africas” [All Fields] OR (“asia” [MeSH Terms] OR “asia” [All Fields])).

Acute illness is more frequent in undernourished children and itself can impact children’s growth, affecting both ponderal (weight) and linear (length or height) growth. During convalescence, children may experience a period of rapid growth, partly influenced by the child’s age, severity of illness, nutritional status, and underlying chronic conditions. Despite its importance, there is a lack of comprehensive data on child growth after hospitalisation, with existing studies predominantly focused on children recovering from severe malnutrition.

**Added value of this study**

Despite implementation of WHO and national guidelines, post-discharge weight gain in both moderately and severely wasted children was poor, and growth velocity significantly decelerated after 45-days. Weight-for-age and weight-for-length stabilized well below community norms in children who were moderately and severely wasted, respectively. For stunting, all groups experienced worsening or no improvement in linear growth. A varying weight recovery patterns suggest the necessity for improved stratification to allocate post-discharge management strategies.

**Implications of all the available evidence**

The initial period following hospital-discharge may hold the potential for proactive intervention and ongoing support, both for children and their families. Paediatric guidelines regarding discharge and post-discharge practices should be re-evaluated with a goal of implementing improved risk stratification and structured longer-term support. In some settings, expanding nutritional support may be appropriate, but identification and care for underlying comorbidities and support for carer and household challenges are needed.

## Introduction

Globally, 49 million children under 5 years of age are wasted and 149 million are stunted, with the highest burdens in sub-Saharan Africa and South Asia.^1^ Malnutrition is associated with increased vulnerability to infectious diseases and mortality,^2^^,^^3^ and with significant morbidity including cognitive impairments.^4^ Long-term sequelae include epigenetic modifications, metabolic alterations, and increased risk of non-communicable diseases.^5^ These factors cumulate and reduce economic capacity in adulthood, and maintain intergenerational poverty which ultimately impacts national economies.^6^

Apart from diet and socioeconomic adversities, malnutrition in early childhood is driven by acute and chronic illnesses. Importantly, the nutritional status of children with severe illness commonly worsens throughout hospitalisation^7^ as they can have increased energy expenditure and symptoms that impact feeding (e.g., reduced appetite or vomiting).^8^ After hospital discharge, children remain vulnerable and are at risk of continued poor nutritional status, re-hospitalisation and mortality.^2^^,^^3^ Contemporary data on how acute illness impacts child growth are scarce, especially in low- and middle-income countries. Children can experience accelerated growth post-discharge^4^^,^^9^^,^^10^ but it remains unclear whether this results in the recovery of growth deficits accrued prior to or during acute illness. Also, the extent to which accelerated growth post-discharge is sustained and dependent on initial nutritional status is unknown.

Patterns of ponderal versus linear growth can differ during recovery (i.e., children may show rapid weight gain but unchanged or slowed linear growth), suggesting that underlying mechanisms may be independent or counter-regulated.^10^ In severely malnourished children hospitalised in Malawi treated with high calorie ready-to-use therapeutic foods (RUTF), while weight-for-height z-scores improved, the children remained stunted post-discharge.^3^ Furthermore, while many countries offer short term re-feeding programs following hospitalisation, these largely only target children with severe malnutrition.

We describe patterns of ponderal and linear growth among young children with no, moderate or severe wasting, or with nutritional oedema during 180-days following acute illness and hospitalisation in sub-Saharan Africa and South Asia. We hypothesised that although growth can accelerate following illness, the extent of nutritional recovery in children with low nutritional status remains suboptimal. We also aimed to identify health and sociodemographic correlates which could guide future intervention trials aimed at improving nutritional status of children with an acute illness. By assessing weight and linear growth trajectories in children with differing nutritional status who survived severe illness, we aim to identify opportunities for interventions to improve childhood resilience during a critical period of vulnerability.

## Methods

### Study design

This is a secondary analysis of the Childhood Acute Illness and Nutrition (CHAIN) Network prospective cohort which, between November 2016 and January 2019, recruited 3101 children at nine hospitals in Africa and South Asia: Dhaka and Matlab Hospitals (Bangladesh), Banfora Referral Hospital (Burkina Faso), Kilifi County, Mbagathi County and Migori County Hospitals (Kenya), Queen Elizabeth Hospital (Malawi), Civil Hospital (Pakistan), and Mulago National Referral Hospital (Uganda). As described in the published study protocol,^11^ children were followed throughout hospital admission and after discharge with follow-up visits at 45, 90 and 180-days post-discharge. Catchment settings differed in urbanisation, access to health care and prevalence of background comorbidities such as HIV and malaria. Prior to study start, sites were audited to optimise care as per national and World Health Organisation (WHO) guidelines.^12^ Cross-network harmonisation of clinical definitions and methods was prioritised through staff training and the use of standard operation procedures and case report forms (available online, https://chainnetwork.org/resources/).

### Ethics statement

Ethical approvals were obtained from each site-affiliated or collaborating institution and from the University of Oxford and all caregivers provided written informed consent for their child to participate in the study. The ethical committees that approved the study are listed in Supplementary Methods S1. This study report follows STROBE recommendations.

### Study participants

Children (aged 2–23 months) were recruited at admission in three strata based on mid-upper arm circumference (MUAC) and the presence/absence of oedema.^2^ However, since access to nutritional programs both in-hospital and post-discharge depended on WHO criteria for malnutrition at admission, children were classified into four nutritional groups: no wasting, moderate wasting, severe wasting, or having nutritional oedema. Criteria for these groups and other anthropometric classifications are presented in Table 1. Children were excluded if they required immediate resuscitation at admission, were intolerant to oral feeds prior to becoming unwell, had an underlying terminal illness or condition requiring surgery in the next 6 months, had a syndromic or genetic diagnosis of chromosomal abnormality, or were admitted primarily for trauma or surgery. Clinical management and discharge were as per site, national and international guidelines. For every third discharged participant, non-ill children from the same age group and community were recruited during household visits to provide community reference values.

**Table 1:** Classification criteria of children hospitalised with acute illness.

Nutritional groups	Criteria
No wasting (NW)	WLZ ≥ −2 or, if age ≥6 months, MUAC ≥ 12.5 cm
Moderately wasted (MW)	WLZ < −2 but ≥ −3 or, if age ≥ 6 months, MUAC ≥ 11.5 to <12.5 cm
Severely wasted (SW)	WLZ < −3 or, if age ≥ 6 months, MUAC <11.5 cm
Nutritional oedema (NO)	having bilateral pitting nutritional oedema either: +, in feet only; ++, in feet and lower limbs, or +++, generalised including upper body and face.
**Other classifications**	**Criteria**
Stunted	LAZ < −2 z-score
Severely stunted	LAZ < −3 z-score
Underweight	WAZ < −2 z-score
Severely underweight	WAZ < −3 z-score
Wasted based on MUACZ	MUACZ < −2
Severely wasted based on MUACZ	MUACZ < −3
Wasted based on WLZ	WLZ < −2
Severely wasted based on WLZ	WLZ < −3

Growth metrics: LAZ, length-for-age z-score; WAZ, weight-for-age z-score; MUAC, mid-upper arm circumference; MUACZ, mid-upper arm circumference z-score; WLZ, weight-for-length z-score.

### Inclusion criteria for growth analysis

We included all children enrolled in CHAIN who survived until 180-days post-discharge and who had weight or length measurements at discharge and at one or both of 90- or 180-day follow-up visits.

### Procedures

At hospital admission, trained staff recorded the participant’s demographics, history, clinical presentation, and diagnosis. In hospital, children were followed daily to document clinical status and medical treatment. Fieldworkers guided caregivers through questionnaires to capture socio-demographic information such as education, income, employment, assets, water, and sanitation (e.g., Demographic and Health Survey questionnaires), food security (adapted from Household Food Insecurity Access Scale), caregiver mental health (Patient Health Questionnaire-9), and transport mode, time, and cost to hospital.^11^

### Growth outcomes

Children’s weight, MUAC and length were measured at admission, discharge, and each follow-up visit using standardised procedures and identical equipment across sites. Children were weighed undressed to the nearest 100 g using an electronic scale (Seca 376 scale, Birmingham, UK) calibrated bi-monthly. MUAC was measured twice to the nearest millimeter with a non-stretch tape (S0145620 Child 11.5 Red, UNICEF, Copenhagen, Denmark). Length was measured twice (nearest 1 mm) using a length board (Seca 416 infantometer). If the discrepancy between measures was more than 5 mm for MUAC or 7 mm for length, they were repeated. The average of the two most coherent measures were analysed. Age- and sex-adjusted z-scores were calculated for each time point with *anthro* R package using WHO 2006 growth references to obtain measures of weight-for-age (WAZ), MUAC-for-age, WLZ and length-for-age (LAZ) z-scores. MUACZ was calculated in children older than 3 months as growth references are not available for this metric in younger children. These children were included in the MUACZ analysis as they had sufficient data at later timepoints (i.e., by the 45-day visit, calculated MUACZ could be obtained). Data cleaning procedures, number and treatment of missing data, lost-to-follow up and number of visits that took place outside of planned follow-up windows are detailed in Supplementary Methods S1 and Supplementary Tables S1–S4. Briefly, outliers per and between timepoints were identified based on standardised z-scores calculated within each nutritional group split by age (≥ or < than 12 months). Multivariate outliers were also identified using clustering based on robust Mahalanobis distance applied to age, height, weight, and MUAC performed at each timepoint using the *mvoutlier* R package. All identified outliers were individually evaluated and removed only if inconsistent with the child’s overall growth trajectory. Missing data at discharge was replaced with either data at admission or, if applicable, readmission (replaced data are detailed in Supplementary Table S2). Values at admission were used for weight if duration of hospital stay was ≤2 days, and length was replaced if hospital stay was less than <20 days.

### Statistical analysis

As described in the study protocol,^11^ the initial study size was estimated to detect difference in mortality between the recruitment strata. The primary outcomes of this secondary analysis were the change in WAZ and LAZ over the 180-days following hospital discharge. WAZ was chosen as, compared to WLZ, it has been more strongly linked to mortality post-discharge.^13^ Secondary wasting-specific outcomes were change in MUACZ and WLZ. Linear regression and piecewise mixed-effects models were fit with *lme4* R package to compare post-discharge growth between nutritional groups. Models used a linear fit to facilitate interpretation and the inclusion of a knot/inflection point at either 45- or 90- days post-discharge was evaluated. The final knot position was chosen based on the overall growth trajectory and fit values (i.e., Akaike Information Criterion (AIC), corrected AIC (AICc) and Bayesian Information Criterion (BIC)). Nutritional groups, time, and their interaction, and age and sex were included *a priori* in the ‘base’ model. Different random structures were assessed, and full model specifications tested are detailed in Supplementary Table S5. From the selected ‘base’ models, marginal means were estimated to evaluate difference between groups at specific time points with *emmeans* and the *multcomp* R package was used to calculate linear combinations of estimates. As previously described (published Supplementary Materials ^2^), latent variables were built to represent key domains of exposures which were designed to reflect the UNICEF conceptual framework. Domains were validated using confirmatory factor analysis and these are described together with other clinical covariates in Table 2. The exposure domains are latent variables constructed to summarise correlated elements into a score attributed to each child (e.g., age-inappropriate nutrition is constructed from 3 variables: recommended appropriate diet, poor feeding, and recent weight loss). These latent scores were split into tertiles, and a child classified within the “high” or “most-adverse” tertile indicates higher risk with regards to the exposure domain. One domain, ‘underlying medical conditions’, was disaggregated for this analysis as it contained ‘stunting’. Additional variables tested for association included common conditions (i.e, severe or moderate anaemia, diarrhoea, severe pneumonia, and sepsis). More complex models were compared to ‘base’ models using anova function with Satterthwaite’s approximation of degrees of freedom as implemented in the *lmerTest* R package. AIC/AICc/BIC were evaluated in models with significantly improved fit (*p* < 0.025) to assess the contribution of each exposure domain and covariate. Selected variables were included in overall models and retained, if remaining significant at *p* < 0.025 amongst other predictors. All analyses and visualisations were performed in R (Version 4.3.1) and figures were finalised with Inkscape (https://inkscape.org/). R packages used and their versions are listed in Supplementary Table S6.

**Table 2:** List of domains and variables tested for association with 180-days post-discharge growth in children after hospitalisation for acute illness.

Exposure domains	
1 Signs of illness severity at admission *(low, medium, high)*	*Having at admission*: abnormal blood glucose; dehydration; reduced consciousness; respiratory distress; severe anaemia; shock; a systemic inflammatory response syndrome score ≥ 2.^a^
2 Signs of illness severity at discharge *(low, medium/high)*	*Having at discharge*: abnormal blood glucose; dehydration; reduced consciousness; respiratory distress; severe anaemia; shock; a systemic inflammatory response syndrome score ≥ 2.^a^
3 Age-inappropriate nutrition *(low, medium, high)*	Not consuming recommended appropriate diet for age (i.e., for age <6 months, exclusive breastfeeding; for age 6–9 months, consuming ≥ 2 food groups and breastmilk; for age 10–23 months, consuming ≥ 4 food groups and breastmilk); complaints of poor feeding; reported recent weight loss.
4 Caregiver characteristics *(least-, moderate-, most- adverse)*	Biological mother not primary caregiver; low education; low mental health score; caregiver illness; caregiver unemployed.
5 Household-level exposures *(least-, moderate-, most- adverse)*	Low household assets; low food security; unimproved toilet; poor water availability.
6 Access to health care *(least-, moderate-, most- adverse)*	High distance to nearest health facility; less efficient travel means; high travel cost; high travel time.
**Clinical variables**	
Underlying conditions (disaggregated)	Prior hospitalisation, small birth size, chronic conditions (i.e., thalassemia, cerebral palsy, sickle cell disease, congenital cardiac diseases and known tuberculosis).
Common syndromes or conditions	Severe pneumonia, diarrhoea, sepsis, anaemia, HIV.

a , defined as having two of the following: 1) abnormal heart rate (low, <90 bpm or high, >180 bpm), abnormal temperature (low, <36.0 °C or high, ≥38.5 °C), high respiratory rate (>34 breaths per min), and abnormal white blood cell count (low, <5.0 cells per μL or high, >17.5 cells per μL). Exposure domains were derived from latent variables previously constructed^2^ to summarise correlated elements into a score that can be attributed to each child. These scores were split into tertiles and a child classified within the “high” or “most-adverse” tertile indicates higher risk with regards to the specific exposure domain.

Three sensitivity analyses were performed to examined whether patterns in post-discharge growth differed: 1) if measurements outside the planned 14-day visit window were excluded; 2) if the analysis was limited to children with complete data; or 3) if all children with at least one data point post-discharge were included.

### Role of the funding source

The funders had no role in the study design, patient recruitment, data collection, analysis, preparation of the manuscript or the decision to publish. CB, NN, MMN and JAB had full access to the dataset. The decision to submit for publication was made by JLW and JAB.

## Results

Overall, 3101 hospitalised children (median age 11 months interquartile range (IQR) [7, 16]) in the CHAIN cohort and 1234 community children (median age, 12 months IQR [8, 17]) were enrolled. Since 182 children died in hospital and 42 families dropped from the study prior to discharge, 2877 children were eligible for inclusion. Following exclusion of post-discharge deaths (n = 168 [11%]) and children with no or insufficient data (n = 237 [10%]), 2472 children were included in the main analysis. These were classified by nutritional group as having: no wasting, 960 (39%); moderate wasting, 572 (23%); severe wasting, 682 (28%), or nutritional oedema 258 (10%). The study flow chart is presented in Supplementary Fig. S1 and Table S7 compares the characteristics of included versus excluded children. The crossover between enrolment strata and nutritional groups is presented in Supplementary Fig. S2. Participant, caregiver, and household characteristics are shown split by nutritional group in Table 3 and by site in Supplementary Tables S8–S12. Children were admitted to hospital with a variety of illnesses, most commonly diarrhoea, severe or moderate anaemia, severe pneumonia, or suspected sepsis. More than half of children (53%) had multiple conditions (Supplementary Fig. S3). Median duration of hospitalisation was 4 days IQR [2, 7] but varied by nutritional group: 3.9 days (95% CI: 3.6, 4.3) in not wasted, 4.6 days (95% CI: 4.1, 5.1) in moderately wasted, 7.9 days (95% CI: 7.4, 8.3) in severely wasted, and 12 days (95% CI: 11, 13) in children with nutritional oedema. The median follow-up time of children was 187 days IQR [182, 195].

**Table 3:** Characteristics of patients at admission split by nutritional groups and of community participants.

	NW n = 960	MW n = 572	SW n = 682	NO n = 258	CP n = 1234
**Demographics**					
Age					
≥12 mo.	382 (40%)	222 (39%)	297 (44%)	183 (71%)	627 (51%)
≥6 & <12 mo.	320 (33%)	245 (43%)	291 (43%)	56 (22%)	396 (32%)
<6 mo.	258 (27%)	105 (18%)	94 (14%)	19 (7.4%)	211 (17%)
Sex (male)	568 (59%)	302 (53%)	395 (58%)	142 (55%)	657 (53%)
**Clinical presentation at admission**					
Sepsis	130 (14%)	65 (11%)	77 (11%)	44 (17%)	0 (0%)
Severe pneumonia^a^	259 (27%)	113 (20%)	105 (15%)	19 (7.4%)	0 (0%)
Impaired consciousness	28 (2.9%)	25 (4.4%)	24 (3.5%)	4 (1.6%)	0 (0%)
Diarrhoea	443 (46%)	366 (64%)	454 (67%)	117 (45%)	0 (0%)
Malaria, positive	147 (15%)	93 (16%)	76 (11%)	36 (14%)	44 (3.6%)
Anaemia^b^					
None	218 (24%)	105 (19%)	152 (23%)	26 (10%)	328 (28%)
Mild	202 (22%)	103 (19%)	157 (24%)	58 (23%)	349 (30%)
Moderate/Severe	491 (54%)	337 (62%)	353 (53%)	166 (66%)	476 (41%)
Blood glucose, abnormal^c^	58 (6.1%)	40 (7.1%)	40 (5.9%)	25 (10.0%)	13 (1.1%)
**Anthropometry at admission**					
LAZ	−1.2 (−2.1, −0.3)	−1.8 (−2.7, −1.1)	−2.7 (−3.7, −1.9)	−2.9 (−3.8, −2.0)	−1.3 (−2.1, −0.5)
WAZ^g^	−1.2 (−1.9, −0.4)	−2.5 (−3.2, −2.0)	−3.9 (−4.6, −3.4)	−3.1 (−4.0, −2.0)	−1.0 (−1.8, −0.1)
MUAC, cm^g^	13.4 (12.7, 14.2)	12.1 (11.7, 12.4)	10.9 (10.2, 11.4)	11.6 (10.7, 12.6)	13.7 (13.0, 14.5)
MUACZ^g^	−0.85 (−1.4, −0.15)	−2.2 (−2.6, −1.8)	−3.4 (−4.1, −2.8)	−2.8 (−3.7, −1.7)	−0.6 (−1.3, 0.0)
WLZ^g^	−0.6 (−1.3, 0.1)	−2.2 (−2.6, −1.8)	−3.5 (−4.1, −3.1)	−2.1 (−3.1, −1.1)	−0.4 (−1.1, 0.4)
**Underlying chronic conditions**					
Stunted					
Not stunted	698 (73%)	317 (55%)	197 (29%)	67 (26%)	891 (72%)
Moderate stunting	161 (17%)	145 (25%)	205 (30%)	72 (28%)	230 (19%)
Severe stunting	101 (11%)	110 (19%)	280 (41%)	119 (46%)	111 (9.0%)
Small birth size^d^	130 (14%)	118 (21%)	144 (21%)	31 (12%)	137 (11%)
HIV status					
Unexposed, uninfected	884 (92%)	525 (92%)	610 (89%)	212 (82%)	1130 (92%)
Exposed, uninfected	59 (6.1%)	30 (5.2%)	38 (5.6%)	32 (12%)	91 (7.4%)
Infected	17 (1.8%)	17 (3.0%)	34 (5.0%)	14 (5.4%)	13 (1.1%)
Chronic conditions^e^	59 (6.1%)	43 (7.5%)	54 (7.9%)	15 (5.8%)	26 (2.1%)
Prior hospitalisation	226 (24%)	147 (26%)	194 (28%)	63 (24%)	121 (9.8%)
**Age-inappropriate nutrition**					
Recommended adequate diet^f^	528 (55%)	330 (58%)	309 (45%)	42 (16%)	753 (61%)
Reported recent weight loss	107 (11%)	210 (37%)	382 (56%)	144 (56%)	47 (3.8%)
Reported poor feeding	75 (7.8%)	54 (9.4%)	181 (27%)	80 (31%)	0 (0%)
Reported current breastfeeding	788 (82%)	473 (83%)	478 (70%)	72 (28%)	1028 (83%)
**Caregiver characteristics**					
Primary caregiver, not biological mother	29 (3.0%)	21 (3.7%)	31 (4.6%)	24 (9.3%)	51 (4.1%)
Caregiver education					
Secondary/Tertiary	338 (35%)	182 (32%)	165 (24%)	69 (27%)	380 (31%)
Primary	405 (42%)	217 (38%)	290 (43%)	133 (52%)	567 (46%)
None	211 (22%)	172 (30%)	222 (33%)	54 (21%)	273 (22%)
Mental health risk, moderate to severe	130 (14%)	91 (16%)	153 (23%)	73 (29%)	126 (10%)
Employment					
Employed	123 (13%)	81 (14%)	90 (13%)	47 (18%)	113 (9.3%)
Self-employed	164 (17%)	81 (14%)	79 (12%)	53 (21%)	284 (23%)
No income	664 (70%)	405 (71%)	505 (75%)	155 (61%)	821 (67%)
**Household-level exposures**					
Population density, 1000 people per km^2^	5.1 (0.6, 17.2)	4.1 (0.8, 20.9)	5.9 (0.8, 25.6)	3.3 (0.3, 9.4)	3.2 (0.4, 13.3)
Asset index					
Least poor	243 (25%)	132 (23%)	151 (22%)	23 (8.9%)	229 (19%)
Fourth	205 (21%)	133 (23%)	150 (22%)	43 (17%)	236 (19%)
Middle	174 (18%)	100 (17%)	151 (22%)	59 (23%)	235 (19%)
Second	181 (19%)	107 (19%)	110 (16%)	71 (28%)	264 (21%)
Poorest	157 (16%)	100 (17%)	120 (18%)	62 (24%)	270 (22%)
Household food insecurity					
Low	603 (63%)	373 (65%)	429 (63%)	115 (45%)	704 (57%)
Medium	265 (28%)	133 (23%)	175 (26%)	92 (36%)	372 (30%)
High	92 (9.6%)	66 (12%)	78 (11%)	51 (20%)	158 (13%)
Toilet, not improved	209 (22%)	104 (18%)	131 (19%)	83 (32%)	322 (26%)
Water source, not improved	132 (14%)	82 (14%)	103 (15%)	47 (18%)	198 (16%)
**Access to health care**					
Distance to hospital, km	7.6 (3.7, 17.2)	9.6 (4.6, 20.0)	9.5 (5.0, 20.0)	10.5 (6.2, 27.3)	8.5 (4.2, 17.8)
Distance to nearest health facility, km	1.2 (0.5, 3.1)	1.2 (0.5, 3.6)	1.2 (0.5, 3.3)	1.4 (0.8, 2.9)	1.4 (0.5, 2.9)
Means of travel to hospital					
Bus, car, ambulance, train	404 (42%)	208 (37%)	283 (42%)	148 (57%)	–
Walking, motorbike, tuktuk, rickshaw	550 (58%)	359 (63%)	394 (58%)	110 (43%)	–
Travel cost to study hospital, US$					
<1$	395 (42%)	188 (33%)	237 (35%)	93 (37%)	–
≥1 to <5$	485 (51%)	292 (52%)	358 (53%)	147 (58%)	–
≥5$	64 (6.8%)	82 (15%)	77 (11%)	14 (5.5%)	–
Travel time to study hospital					
Less than 1 hr	460 (49%)	239 (42%)	256 (38%)	79 (31%)	–
Between 1 hr and 2 hr	366 (39%)	187 (33%)	254 (38%)	110 (43%)	–
2 hr or more	122 (13%)	139 (25%)	165 (24%)	65 (26%)	–

Results presented as frequency (%) or median (IQR).

a Severe pneumonia: cough or difficulty breathing with oxygen saturation <90%, central cyanosis, or grunting; very severe chest indrawing or inability to breastfeed or drink; or lethargy, reduced level of consciousness, or convulsions.

b Anaemia: none, haemoglobin >110 g/L; mild 100–110 g/L; moderate/severe, <100 g/L.

c Blood glucose <3 mmol/L or >10 mmol/L.

d Birth size: reported premature or low birthweight (<2.5 kg).

e Chronic conditions: include thalassemia, cerebral palsy, sickle cell disease, congenital cardiac diseases and known tuberculosis.

f Recommended adequate diet: for age <6 months, exclusive breastfeeding; for age 6–9 months, consuming ≥ 2 food groups and breastmilk; for age 10–23 months, consuming ≥ 4 food groups and breastmilk, as reported by caregiver.

g Indicates metrics that can be influenced by fluid retention; MUACZ is considered less sensitive to oedema than WAZ or WLZ. Groups: NW, no wasting; MW, moderate wasting; SW, severe wasting; NO, nutritional oedema; CP, community participants. Growth metrics: LAZ, length-for-age z-score; WAZ, weight-for-age z-score; MUAC, mid-upper arm circumference; MUACZ, mid-upper arm circumference z-score; WLZ, weight-for-length z-score.

### Linear growth slowed but weight gain increased post-discharge

**Fig. 1:**
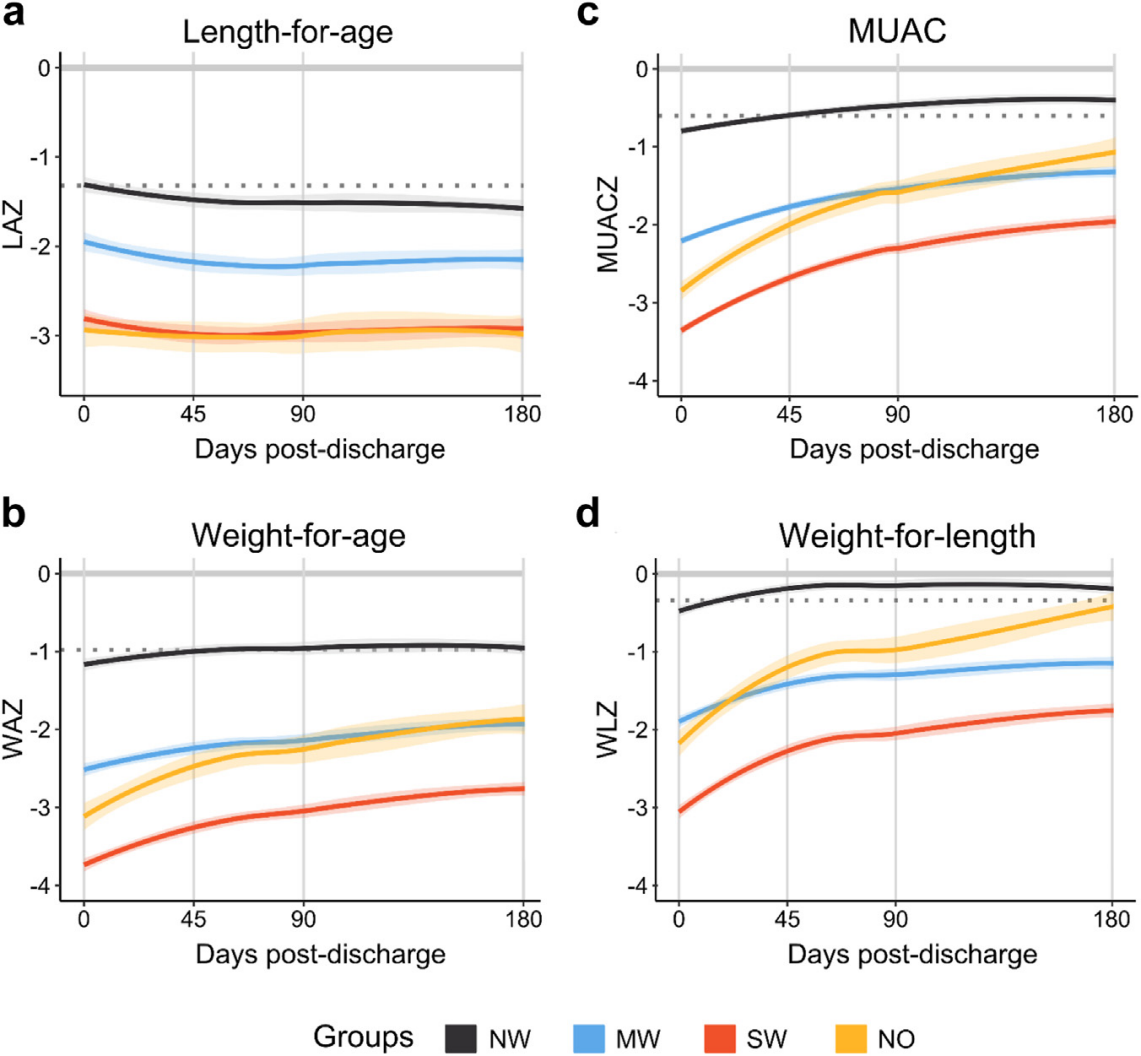
Post-discharge growth of children by nutritional group as classified at hospital admission: a) Length-for-age z-score (LAZ); b) Weight-for-age z-score (WAZ); c) Mid-upper arm circumference z-scores (MUACZ), d) Weight-for-length z-score (WLZ) in children who are not wasted (NW), moderately wasted (MW), severely wasted (SW) or have nutritional oedema (NO). Growth trajectories were fitted with locally estimated scatterplot smoothing (LOESS) curves; line color indicates nutritional groups as per legend; colored shaded areas represent the standard error of the mean. Grey dotted line shows average z-score of community participants.

Based on model selection (Supplementary Tables S5 and S13), the base growth trajectory models included a knot point at day-45 which allowed to test whether growth differed between the early (before 45-days) or late (45–180-days) post-discharge periods (Fig. 1, Table 4 and, Supplementary Table S14). LAZ showed a slowing of monthly linear growth in the early post-discharge period in children with no or moderate wasting (i.e., groups that also showed less initial stunting). This decline in LAZ stabilised after 45-days post-discharge. In contrast, the LAZ of children with severe wasting or nutritional oedema showed minimal change with no significant increase in monthly linear growth. The early post-discharge period also coincided with accelerated weight gain. Having larger weight deficits at discharge, children with moderate- or severe-wasting or with nutritional oedema showed greater monthly ponderal (WAZ, WLZ) and MUACZ growth rates. These gains were greatest in the first 45-days after discharge but later tapered. Children with nutritional oedema had greater monthly gains in WAZ, MUACZ and WLZ followed by those with severe wasting and then moderate wasting. Children without wasting had the smallest post-discharge gains since, by 45-days after discharge, their anthropometric measures reached or surpassed those of community participants (Supplementary Fig. S4). Post-discharge growth trajectories split by nutritional group and site are presented in Supplementary Figs. S5–S8 and change in absolute growth measures by nutritional group are in Supplementary Table S15 and by site and group in Supplementary Tables S16–S19. LAZ and WAZ were highly correlated (r = 0.77 (95% CI: 0.77, 0.78), t (9435) = 119, *p* < 0.0001, Supplementary Fig. S9). Post-discharge growth patterns were similar across geographical settings and near identical results were obtained from the three sensitivity analyses.

**Table 4:** Monthly growth across the 180-day post-discharge period and monthly growth rates during early (before 45-days) and late (after 45-days) periods presented by nutritional group at hospital admission.

	Overall		Monthly growth rate					
	180-days post-discharge growth		Early period		Late period		Difference Late vs. Early	
	Est.	95% CI	Est.	95% CI	Est.	95% CI	Est.	95% CI
**LAZ**								
NW	−0.27	−0.36, −0.19	−0.13	−0.15, −0.11	−0.02	−0.028, −0.0092	0.11	0.087, 0.13
MW	−0.23	−0.34, −0.11	−0.15	−0.17, −0.12	−0.0021	−0.014, 0.0099	0.14	0.12, 0.17
SW	−0.089	−0.19, 0.015	−0.12	−0.14, −0.094	0.019	0.0083, 0.030	0.14	0.11, 0.16
NO	−0.009	−0.18, 0.16	−0.056	−0.094, −0.018	0.017	−0.0014, 0.035	0.073	0.031, 0.11
**WAZ**								
NW	0.21	0.11, 0.32	0.12	0.09, 0.15	0.007	−0.0045, 0.018	−0.12	−0.15, −0.085
MW	0.57	0.44, 0.71	0.21	0.17, 0.24	0.059	0.044, 0.073	−0.15	−0.19, −0.11
SW	1.0	0.88, 1.1	0.36	0.32, 0.39	0.10	0.091, 0.12	−0.25	−0.29, −0.22
NO	1.3	1.1, 1.5	0.44	0.39, 0.50	0.13	0.11, 0.15	−0.31	−0.38, −0.25
**MUACZ**								
NW	0.42	0.31, 0.53	0.17	0.13, 0.20	0.038	−0.026, 0.051	−0.13	−0.17, −0.090
MW	0.91	0.76, 1.0	0.36	0.32, 0.40	0.082	0.066, 0.098	−0.28	−0.33, −0.23
SW	1.4	1.3, 1.5	0.54	0.50, 0.58	0.13	0.12, 0.15	−0.41	−0.45, −0.36
NO	1.7	1.5, 1.9	0.66	0.60, 0.73	0.16	0.14, 0.19	−0.50	−0.57, −0.43
**WLZ**								
NW	0.28	0.16, 0.41	0.22	0.18, 0.26	−0.010	−0.024, 0.0040	−0.23	−0.27, −0.18
MW	0.75	0.59, 0.92	0.35	0.30, 0.40	0.05	0.032, 0.069	−0.30	−0.36, −0.24
SW	1.3	1.2, 1.5	0.58	0.54, 0.63	0.099	0.082, 0.12	−0.48	−0.54, −0.43
NO	1.7	1.5, 2.0	0.66	0.58, 0.74	0.16	0.14, 0.19	−0.50	−0.59, −0.41

Growth across the 180-days post-discharge period was estimated from the pairwise difference between model-derived marginal means calculated at discharge and 180-days. Monthly growth rates are estimated from the slope coefficients calculated for the early (discharge to 45-days) and late (45-days to 180-days) post-discharge periods for each nutritional group. Anthropometry trajectories were modelled using piecewise linear mixed models with a knot point set at 45-days post-discharge. Knot position was chosen based on fit metrics. Slope coefficients and their difference between early vs. late post-discharge periods were tested against the null hypothesis where: H_0_, monthly growth rate/slope = 0; or H_0_, difference in growth rates/slopes before and after 45-days = 0. Full model results are presented in Supplementary Table S14. Groups: NW, no wasting; MW, moderately wasted; SW, severely wasted; NO, nutritional oedema. Growth metrics: LAZ, length-for-age z-score; WAZ, weight-for-age z-score; MUACZ, mid-upper arm circumference z-score; WLZ, weight-for-length z-score.

Anthropometry in malnourished children at 180-days post-discharge was lower than community participants.

At 180-days post-discharge, LAZ of children with moderate- or severe-wasting or with nutritional oedema was lower than community participants by 0.70 (95% CI: 0.51, 0.90), 1.4 (95% CI: 1.3, 1.6), and 1.3 (95% CI: 1.1, 1.6) z-scores, respectively; and their WAZ was lower by 0.85 (95% CI: 0.68, 1.0), 1.6 (95% CI: 1.5, 1.8), and 0.81 (95% CI: 0.57, 1.0) z-scores, respectively. Differences between nutritional groups at 180-days post-discharge and their comparison with community participants are presented in Table 5 and Supplementary Fig. S4. Children with severe wasting had the lowest mean MUACZ and WLZ followed by those with moderate wasting and nutritional oedema. At 180-days post-discharge, the MUACZ and WLZ of children with no wasting were marginally greater than that of community participant. However, the MUACZ of children with either moderate- or severe-wasting or with nutritional oedema remained lower by 0.67 (95% CI: 0.52, 0.82), 1.3 (95% CI: 1.2, 1.5), and 0.48 (95% CI: 0.27, 0.68) z-scores, respectively; while the WLZ of children with moderate- or severe-wasting remained lower by 0.7 (95% CI: 0.53, 0.87), and 1.3 (95% CI: 1.1, 1.4) z-scores respectively. In contrast, having higher monthly gains, the WLZ of children with nutritional oedema no longer differed from that of community participants.

**Table 5:** Means and group comparisons of anthropometry at 180-days post-discharge in children hospitalised with acute illness and with community participants.

	LAZ		WAZ		MUACZ		WLZ	
	est.	95% CI	est.	95% CI	est.	95% CI	est.	95% CI
NW	−1.5	−1.8, −1.3	−0.92	−1.1, −0.8	−0.36	−0.48, −0.23	−0.18	−0.31, −0.059
MW	−2.2	−2.4, −1.9	−1.9	−2.1, −1.8	−1.27	−1.41, −1.13	−1.1	−1.3, −1.0
SW	−2.9	−3.2, −2.7	−2.7	−2.9, −2.6	−1.92	−2.05, −1.78	−1.7	−1.8, −1.6
NO	−2.9	−3.2, −2.6	−2.0	−2.2, −1.8	−1.18	−1.35, −1.01	−0.54	−0.71, −0.36
Contrasts between nutritional groups								
NW vs MW	0.63	0.40, 0.86	0.99	0.79, 1.2	0.91	0.73, 1.1	0.95	0.75, 1.2
NW vs SW	1.4	1.2, 1.6	1.8	1.6, 2.0	1.6	1.4, 1.7	1.5	1.3, 1.7
NW vs NO	1.3	1.0, 1.7	1.1	0.77, 1.3	0.82	0.57, 1.1	0.35	0.077, 0.63
MW vs SW	0.75	0.51, 0.99	0.80	0.58, 1.0	0.65	0.45, 0.84	0.57	0.36, 0.79
MW vs NO	0.71	0.38, 1.0	0.056	−0.25, 0.36	−0.093	−0.36, 0.17	−0.60	−0.89, −0.31
SW vs NO	−0.041	−0.37, 0.28	−0.74	−1.0, −0.45	−0.74	−1.00, −0.48	−1.2	−1.5, −0.88
**LAZ**	est.	95% CI	est.	95% CI	est.	95% CI	est.	95% CI
CP	−1.4	−1.6, −1.2	−1.0	−1.2, −0.84	−0.6	−0.75, −0.46	−0.38	−0.56, −0.20
Contrasts community vs. nutritional groups								
CP vs NW	0.080	−0.082, 0.24	−0.13	−0.27, 0.016	−0.23	−0.35, −0.098	−0.22	−0.36, −0.076
CP vs MW	0.70	0.51, 0.90	0.85	0.68, 1.0	0.67	0.52, 0.82	0.70	0.53, 0.87
CP vs SW	1.4	1.3, 1.6	1.6	1.5, 1.8	1.30	1.2, 1.5	1.3	1.1, 1.4
CP vs NO	1.3	1.1, 1.6	0.81	0.57, 1.0	0.48	0.27, 0.69	0.09	−0.15, 0.33

Marginal means and contrasts were calculated at 180-days post-discharge for each growth metric as derived from piecewise linear mixed models with a knot point set at 45-days post-discharge. Full models are presented in Supplementary Table S14. Knot position was chosen based on fit metrics. Generalised linear models were used to compare community children and anthropometric measures of the groups at 180-days. NW, no wasting; MW, moderately wasted; SW, severely wasted; NO, nutritional oedema. LAZ, length-for-age z-score; WAZ, weight-for-age z-score; MUACZ, mid-upper arm circumference z-scores; WLZ, weight-for-length z-score.

### Most hospitalised children have sub-optimal nutritional status at 180-days post-discharge

**Fig. 2:**
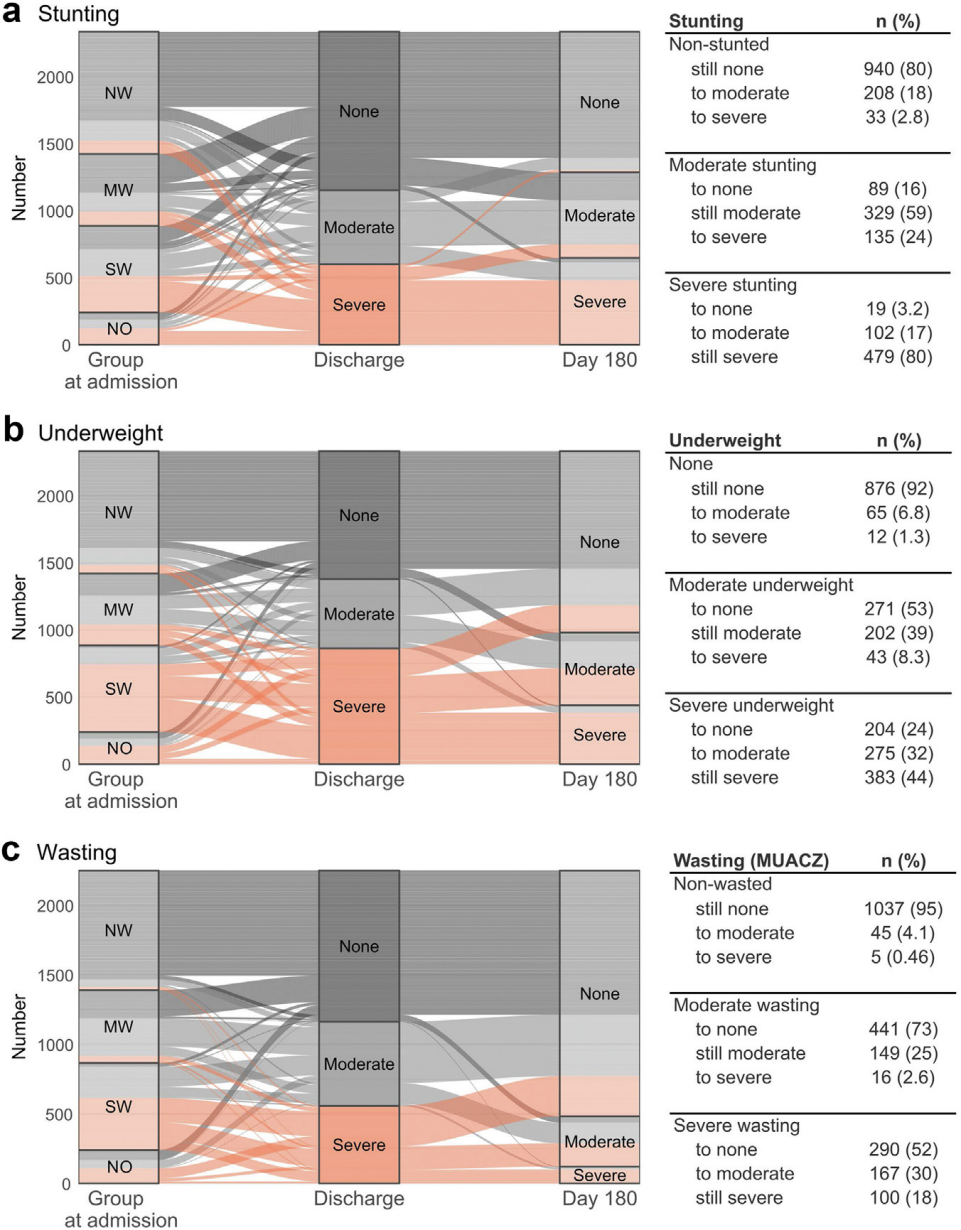
Alluvial plots detailing the change in nutritional classification of children from discharge (center) to 180-days post discharge (left) for: a) Stunting, based on LAZ; b) Underweight, based on WAZ; c) Wasting, based on MUACZ. Wasting based on WLZ is presented in Supplementary Fig. S9. Classification is as follows: None ≥ −2 z-score (dark grey); Moderate < −2 to ≥ −3 z-score (light grey); Severe < −3 z-score (orange). Each flow line depicts how an individual child changes between initial group at admission (x-axis, left) and their nutritional classification at discharge (center), and 180-days post-discharge (right). Y-axis presents stacked child counts. Tables detail the number and percentage of children in each trajectory pattern seen between discharge and 180-days. NW, no wasting; MW, moderately wasted; SW, severely wasted; NO, nutritional oedema. LAZ, length-for-age z-score; WAZ, weight-for-age z-score, MUACZ, mid upper arm circumference z-score; WLZ, weight-for-length z-score.

We then evaluated how children change nutritional classification from stunting, underweight, or wasting between discharge and 180-days post-discharge, focusing the change from the point of discharge (Fig. 2). Stunting, underweight and wasting categories are defined in Table 1. In all groups, stunting was largely unchanged with 80% (n = 479/600) of children severely stunted at discharge remaining as such by 180-days, but 135/553 (24%) of those with moderate stunting at discharge moved to severe by 180-days, and 241/1181 (20%) with no-stunting moved to either moderate or severe stunting by 180-days post-discharge. Among children discharged as severely underweight some improved to not-underweight after 180-days (n = 204/862, 24%), while half of children discharged as moderately underweight improved classification to not-underweight by 180-days (n = 271/516, 53%). The other half of moderately underweight children remained classified as either moderately or severely underweight (n = 245/516, 47%) and of these 43 (8.3%) move to severely underweight. Overall, most children who were underweight at discharge were still underweight after 180 days (n = 903/1378, 66%). For wasting, half of the children with severe wasting at discharge (based on MUACZ) were no longer wasted by 180-days (n = 290/557, 52%). However, 30% (n = 167/557) were still moderately wasted and 18% (n = 100/557) severely wasted; thus 48% of children discharge with wasting remained wasted at 180-days post-discharge. Wasting based on WLZ showed a similar pattern (Supplementary Fig. S10) with some children (n = 79/1048, 7.5%) declining from not-wasted at discharge to moderately or severely wasted and 5.2% (33/639) of those with moderate wasting decline to severe wasting by 180-days post-discharge.

### Factors associated with post-discharge growth

**Fig. 3:**
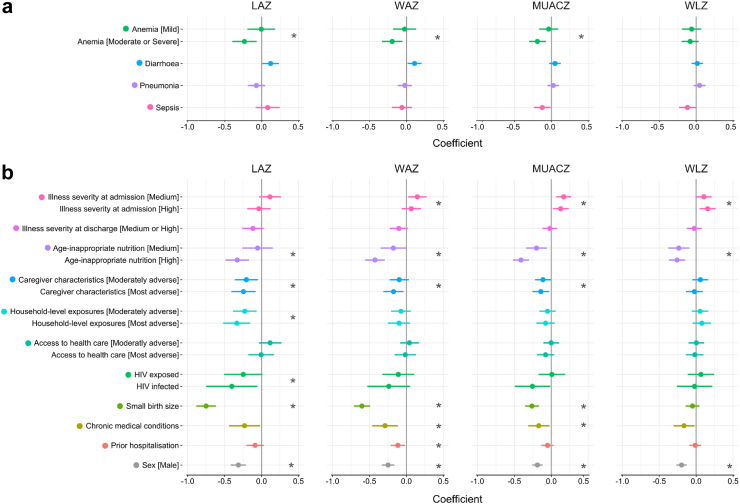
Forest plots present the association between each post-discharge growth metric and clinical variables or exposure domains. Plotted coefficients for a) common syndromes diagnosed at admission (i.e., anaemia, diarrhoea, severe pneumonia, and sepsis) and b) exposure domains and other clinical variables (i.e., HIV status, birth size, chronic medical condition, and prior hospitalisation). Stars indicate variables that significantly improved model fit compared to the ‘base’ models (Significance threshold, *p* < 0.025). The ‘base’ piecewise mixed models included a single knot point at 45-days post-discharge with adjustment for age, and sex with random slopes per participants and random intercepts for sites with participants nested within. Full model results for LAZ, WAZ, MUACZ, and WLZ are presented in Supplementary Tables S20–S47, respectively. LAZ, length-for-age z-score; WAZ, weight-for-age z-score; MUACZ, mid-upper arm circumference z-scores; WLZ, weight-for-length z-score.

Finally, we tested whether the most common clinical conditions diagnosed at admission and other clinical and exposure domains were associated with post-discharge growth (Fig. 3 and full model results in Supplementary Tables S20–S26 (LAZ), Supplementary Tables S27–S33 (WAZ), Supplementary Tables S34–S40 (MUACZ), Supplementary Tables S41–S47 (WLZ). Domains and variables showing significance were included in an overall multivariate model for each growth outcome as shown in Table 6 and detailed in Supplementary Tables S48 and S49. Of the clinical factors, moderate and severe anaemia showed the highest effect size and association with post-discharge LAZ, WAZ, and MUACZ. Lower post-discharge LAZ was associated with age-inappropriate nutrition, more adverse caregiver characteristics, household-level exposures, HIV exposure or infection, and small birth size. Lower WAZ in the 180-days post-discharge was associated with age-inappropriate nutrition, more adverse caregiver characteristics, small birth size (defined as low birth weight [<2.5 kg] or premature birth), having chronic conditions (i.e., cerebral palsy, sickle cell disease, congenital cardiac disease, and tuberculosis), and having experienced prior hospitalisation. Lower MUACZ was associated with age-inappropriate nutrition, more adverse caregiver characteristics, small birth size, and having chronic conditions. In turn, lower post-discharge WLZ was associated with age-inappropriate nutrition. Being male was associated with lower growth in all four metrics. Growth trajectories are presented by group split by each domain in Supplementary Figs. S11 and S12 or by additional clinical variables in Supplementary Figs. S13 and S14. Supplementary Table S50 presents the number of participants classified within each level of the domain variables split by nutritional group. Results were unchanged when further adjusted for caregiver stunting and BMI (Supplementary Figs. S15).

**Table 6:** Multivariable models associating domains variables with 180-days post-discharge growth.

Final models												
Predictors	LAZ			WAZ			MUACZ			WLZ		
	Est.	95% CI	*p*	Est.	95% CI	*p*	Est.	95% CI	*p*	Est.	95% CI	*p*
Age-inappropriate nutrition [medium]	−0.07	−0.24, 0.09	0.38	−0.19	−0.33, −0.05	0.0088	−0.2	−0.32, −0.09	0.00062	−0.23	−0.35, −0.11	0.00016
Age-inappropriate nutrition [high]	−0.33	−0.46, −0.20	<0.00001	−0.4	−0.51, −0.29	<0.00001	−0.4	−0.49, −0.31	<0.00001	−0.25	−0.35, −0.16	<0.00001
Caregiver characteristics [Moderatly adverse]	−0.19	−0.32, −0.06	0.0037	−0.1	−0.20, 0.01	0.07	−0.1	−0.19, −0.02	0.021			
Caregiver Characteristics [Most adverse]	−0.2	−0.33, −0.07	0.0032	−0.16	−0.27, −0.05	0.0053	−0.13	−0.22, −0.03	0.0073			
Small birth size	−0.74	−0.87, −0.61	<0.00001	−0.58	−0.69, −0.47	<0.00001	−0.24	−0.33, −0.15	<0.00001			
Anaemia [Mild]	0.00	−0.15, 0.15	0.98	−0.01	−0.13, 0.11	0.88	−0.02	−0.13, 0.08	0.66			
Anaemia [Moderate or Severe]	−0.18	−0.31, −0.05	0.0086	−0.14	−0.25, −0.03	0.012	−0.16	−0.25, −0.06	0.00091			
Illness severity at admission [medium]				0.13	0.03, 0.23	0.0093	0.16	0.07, 0.24	0.00025	0.10	0.01, 0.18	0.024
Illness severity at admission [high]				0.06	−0.05, 0.16	0.31	0.12	0.03, 0.21	0.0083	0.14	0.05, 0.23	0.0020
Household-level exposures [Moderatly adverse]	−0.25	−0.38, −0.11	0.00023									
Household-level exposures [Most adverse]	−0.34	−0.49, −0.19	<0.00001									
Chronic medical condition				−0.2	−0.37, −0.04	0.017						

Table presents fixed effects results of mixed piecewise models with a knot point at 45-days post-discharge which defines the earlier (before 45-days) and later periods (after 45-days post-discharge). Random structure included a slope per participant and a random intercept for sites with participants nested within. LAZ, length-for-age z-score; WAZ, weight-for-age z-score; MUACZ, mid-upper arm circumference z-scores; WLZ, weight-for-length z-score. Full results are presented in Supplementary Tables S48 and S49.

## Discussion

We studied post-discharge growth in a large cohort of acutely ill children hospitalised in six different countries, four in Sub-Saharan Africa and two in South Asia. To our knowledge, this data represents the largest cohort with post-illness growth assessment in low-resource settings.

Similar to previous studies,^3^^,^^9^^,^^10^^,^^14^, ^15^, ^16^ most children with severe malnutrition in this cohort experienced accelerated weight gain after hospital discharge. However, the higher ponderal gains early post-discharge tapered after 45-days. Thus, their weight gain was often sub-optimal as 48% of children severely wasted at discharge remaining wasted at 180-days post-discharge. This is despite implementing nutritional care guidelines for the management of severe malnutrition that includes the provision of RUTF. Furthermore, based on WLZ, the nutritional status of 7.5% of children without wasting at discharge declined to moderate or severe and 5.2% of those with moderate wasting declined to severe wasting by 180-days. Also, a large fraction of children (66%) who were underweight at discharge were still underweight after 180 days.

Most children with nutritional oedema experienced marked ponderal recovery. At discharge, their WAZ, MUACZ, and WLZ were below that of children with moderate wasting, yet by 180-days, their WAZ and MUACZ were similar to children with moderate wasting and their WLZ was comparable to children in the community, an improvement in WLZ not achieved by children with moderate or severe wasting. While possibly related to older age or differences in home environments, the pattern of growth recovery in children with nutritional oedema may indicate a distinct underlying pathophysiological mechanism compared to wasted children.^4^^,^^17^ In accordance with WHO guidelines, children with severe wasting or nutritional oedema received RUTF and antibiotics in-hospital and RUTF supplements post-discharge. However, better understanding their pathophysiological differences could open opportunities for more targeted and specified interventions.^4^^,^^17^^,^^18^ While higher post-discharge weight gain has been observed in African children with nutritional oedema compared to those with severe wasting,^18^ the comparison to children with moderate wasting helps contextualise the magnitude of their recovery. On the other hand, children with moderate wasting may benefit from the additional post-discharge support, which is recommended for children with nutritional oedema.

Improvement in linear growth was uncommon after hospitalisation in this large cohort. Slowed height accrual early post-discharge was observed in children with no or moderate wasting, i.e., in groups with less severe stunting, while they had no or minimal compensatory increase in LAZ later post-discharge. Children with severe wasting or nutritional oedema experienced continued linear growth faltering with largely unchanged height velocity. Short-term linear growth deterioration has been described during and after an episode of acute illness.^14^ However, nutritional-based strategies attempting to prevent stunting have had limited results. Among non-acutely ill children, the daily distribution of an egg for 6-months to young Ecuadorians initially improved stunting by 47%, but the effects did not persist beyond 2 years^19^ and were not replicated in a Malawian cohort.^20^ Lipid-based nutritional supplementation for 12-months among young Zimbabwean^21^ children reduced stunting by 8%, whereas nutrition and water, sanitation and hygiene (WASH) interventions have yielded modest, if any, impact on stunting.^22^ This implies that these interventions do not address the drivers of poor linear growth and there is a lack of standard management strategies for stunted children.

During acute illness, alterations in energy expenditure, inflammation due to infection, and protein metabolism may result in slowed growth in children.^23^ These processes could also, in part, explain the observed sex differences, where poorer growth in males^24^ may relate to having higher energy expenditure^25^ and higher vulnerability to infection linked to heightened immune reactivity.^26^ Acute illness itself and antibiotics could add to intestinal mucosa disruption and changes in microbiota which are reported to precede stunting.^27^ With continued exposure to infectious diseases, inadequate nutrition, and poor growth, children remain at high risk of relapse^18^ and mortality post-discharge.^2^^,^^3^ Thus, the period after hospitalisation may offer a critical point of intervention to promote longer-term resilience in children. There is evidence that a two-week supplementation with ready-to-use-therapeutic food given post-discharge to non-wasted children was beneficial in reducing the incidence of wasting in the 6-months after discharge.^28^ To generate novel interventions beyond nutritional supplementation, the biological mechanisms and social drivers of weight and length growth need deeper understanding.

The clinical and social determinants associated with post-discharge growth reflect the predictors of post-discharge mortality among children in this cohort as recently published.^2^ Several predictors that we had previously associated with mortality were also associated with post-discharge growth including: HIV infection, small birth size, chronic conditions, illness severity at admission, age-inappropriate nutrition, household level exposures, and more adverse caregiver characteristics. Interestingly, WLZ was less responsive to HIV, size at birth and the home environment (Fig. 3), likely because of proportionately low weight and height. Children with both low MUAC and high illness severity at admission had greater mortality risk whereas those with high illness severity at admission and higher MUAC predominantly survived to 180-days post-discharge.^2^ Thus, interventions that reduce child mortality may also improve growth and vice versa.

An important predictor of poor growth post-discharge was small size at birth, defined as low birth weight or prematurity. Early child growth is ideally corrected for gestational age^29^ but accurate estimates are largely unavailable in lower resource settings. Small for gestational age infants typically experience accelerated growth, with up to 85% achieving catch-up by 24-months.^30^ However, these children are also more susceptible to infection and early chronic conditions,^29^ which could disrupt their growth acceleration leaving them more vulnerable to growth failure. Interventions targeting pre-conception and in utero growth can be successful in improving growth and may be key in reducing vulnerability in early childhood.^31^ Age-inappropriate nutrition (including lack of age-appropriate diet, complaints of poor feeding and recent weight loss) were associated with all metrics of post-discharge growth. This reinforces the importance of adequate nutrition and feeding practices which depend on caregivers. Adverse caregiver characteristics, in terms of maternal education and mental health, were associated with low post-discharge LAZ and MUACZ. The impact of caregivers on early child health and development is well established^32^ but complications arise when caring for a severely ill child. Caregivers of hospitalised children have voiced issues regarding competing family stressors, their own health, and the reliance on providers for income and food.^33^ Social interventions have shown promising results including counselling for breast- or complementary feeding, as well as the provision of food, supplements, and/or conditional cash transfers.^31^ These interventions can be tailored to support caregivers of children recovering from severe illness.

Our study has several strengths. The CHAIN cohort was highly rigorous and followed numerous children from diverse geographical and epidemiological settings, which increases the generalisability of our results. Participant retention at 180-days was excellent. However, our study has limitations. Two main outcomes were chosen for their clinical importance (LAZ and WAZ), and while these are highly correlated and share a high degree of information, this can lead to multiplicity issues. Growth was evaluated at three visits (45-, 90- and 180- days), thus, not capturing very early post-discharge patterns. While we were unable to follow children beyond 180-days, although longer studies have shown similar results.^10^^,^^16^ This analysis focused on the average group trajectory which can mask heterogeneity of relevant sub-groups. Also, precise information on gestational age and birth size was not available. Finally, the domain variables were constructed based on expert knowledge and, while we believe this to be a strength, an agnostic data driven approach could also be considered.

In conclusion, young children recovering from acute illness following discharge from hospital in sub-Saharan Africa and South Asia represent a vulnerable population. Most do not reach their growth potential in the 180-days post-discharge despite hospital-based care provision in accordance with international, national, and local guidelines. Novel approaches are needed to support short- and long-term recovery and to reduce morbidity and mortality associated with poor growth. Hospitalisation represents an opportunity to identify high-risk children that would benefit from interventions beyond provision of nutritional supplements that also extends to supporting caregivers and ameliorate household-level barriers to sustain child growth recovery.

## Contributors

**Funding:** JAB and JLW. **Growth analysis concept and design:** AFS, CLL, CB, CJM, DMD, EMu, JAB, JLW, JJ, JMN, KDT, MMa, MJC, MMN, PS, RHJB, SMo, SAA, TA, and WPV. **Study coordination:** AHD, AFS, BOS, CT, CAO, CB, COO, CLL, CMar, CL, DIM, DC, JAB, JMN, JM, JT, JLW, KDT, MJC, MT, MMb, NN, PA, PS, RHJB, RMB, SMo, WPV, and ZK. **Study supervision**: AHD, AFS, BOS, CB, CLL, CMan, CJM, DMD, EMb, EMu, GMSM, JAB, JM, JT, JLW, KDT, MMa, MJC, MT, PS, RHJB, SMo, SMw, SAA, TA, WPV, and ZK. **Data collection:** ASMSBS, AFK, COO, CMan, CJM, CL, DA, DM, EMb, IO, JM, LS, MH, MT, PPH, PS, SMw, SNS, SMA, and SB. **Data management**: AHD, BSG, CAO, CB, CMar, EC, JDC, JJ, MMb, MMN, NN, PA, and RMB. **Data cleaning and verification**: CB, CJM, CMar, DB, JAB, JJ, JMN and MMN. **Data analysis**: CB, CMar, CJM, DB, JAB, JLW, JJ, JMN and MMN. **Data interpretation**: AHD, BSG, CAO, CB, CJM, CLL, DB, DMD, EMu, IO, JAB, JLW, JMN, KDT, MJC, MMN, PS, RHJB, RMB, SMo, SAA, TA, and WPV. **First draft**: CB and CJM. **Critical review**: AHD, ASMSBS, AFK, AFS, BOS, BSG, CT, CAO, CB, COO, CLL, CMan, CJM, CMar, CL, DB, DA, DM, DMD, DIM, DC, EMb, EMu, GMSM, IO, JAB, JDC, JJ, JLW, JM, JMN, JT, KDT, LS, MMa, MH, MJC, MT, MMb, MMN, NN, PA, PPH, PS, RHJB, RMB, SMo, SMw, SNS, SAA, SMA, SB, TA, WPV, and ZK. All members of the writing group could access the data and it was accessed and verified by CB, CJM, CMar, DD, JJ, JMN, KDT, MMN, JAB, and JLW. All members of the writing group were responsible for the final decision to submit for publication.

## Data sharing statement

Data compiled by CHAIN cohort and analysis code are deposited and may be requested through the Harvard Dataverse website (https://dataverse.harvard.edu/dataverse/chain).

## Declaration of interests

Members of the writing group declare having received support from the Bill and Melinda Gates Foundation (BMGF) for staff and research activities directly linked to this project which was paid to their universities or institutions (JAB, JLW, RHJB). Reimbursement for travel directly related to this project was also provided by BMGF and paid through the universities (JLW, JAB). JAB participated in a leadership role for the Commonwealth Association for Paediatic Gastroenterology & Nutrition (CAPGAN) and on a Data Safety Monitoring Board (DSMB) for a study regarding vitamin D.
