# Supplementary material for: Childhood growth during recovery from acute illness in Africa and South Asia: a secondary analysis of the childhood acute illness and nutrition (CHAIN) prospective cohort
Source: eClinicalMedicine. 2024 Mar 12;70:102530. doi: 10.1016/j.eclinm.2024.102530 (PMC10950691; doi:10.1016/j.eclinm.2024.102530)
Supplement: Supplementary Figs. S1–S15 and Tables S1–S49 [file mmc1.docx]

**Supplemental Materials**

Childhood growth during recovery from acute illness in Africa and South Asia: a secondary analysis of The Childhood Acute Illness and Nutrition (CHAIN) prospective cohort

By The Childhood Acute Illness and Nutrition (CHAIN) Network

Contents

[1. Supplemental Methods: Ethical approvals 5](#_Toc158195142)

[2. Supplemental Methods: Data cleaning 5](#_Toc158195143)

[**Supplemental Table 1**. Tally of implausible values for each anthropometric measure split by recruitment group and timepoint. 6](#_Toc158195144)

[**Supplemental Table 2**. Tally of missing discharge anthropometry replaced by admission or readmission values presented split by recruitment groups. 7](#_Toc158195145)

[**Supplemental Table 3.** Tally of missing anthropometry and proportion of missingness for each measure. 8](#_Toc158195146)

[**Supplemental Table 4.** Follow-up visits outside planned 14-day window split per group and timepoint in children included in the main analysis. 10](#_Toc158195147)

[**Supplemental Table 5**. List of base models and alternative random structures assessed for each anthropometric measure. 11](#_Toc158195148)

[**Supplemental Table 6**. List of R packages and version used. 12](#_Toc158195149)

[4. Supplemental Results 13](#_Toc158195150)

[**Supplemental Figure 1.** Flow chart of study participants. 13](#_Toc158195151)

[**Supplemental Table 7.** Comparison of clinical and sociodemographic characteristics between children discharged after acute illness with sufficient (included) vs. insufficient (excluded) growth data post-discharge presented split by nutritional group. 14](#_Toc158195152)

[**Supplemental Figure 2.** Alluvial plot and associated table presenting the redistribution of children grouped by enrolment criteria compared to study classification by nutritional groups at admission. 17](#_Toc158195153)

[**Supplemental Table 8.** Participant characteristics at admission by nutritional group for Banfora, and Blantyre. 18](#_Toc158195154)

[**Supplemental Table 9.** Participant characteristics at admission by nutritional group for Kampala and Kilifi. 21](#_Toc158195155)

[**Supplemental Table 10.** Participant characteristics at admission by nutritional group for Migori and Nairobi. 24](#_Toc158195156)

[**Supplemental Table 11.** Participant characteristics at admission by nutritional group for Karachi and Matlab. 27](#_Toc158195157)

[**Supplemental Table 12.** Participant characteristics at admission by nutritional group for Dhaka. 30](#_Toc158195158)

[**Supplemental Figure 3**. Venn diagram detailing number and percent of children admitted to hospital with multiple conditions 33](#_Toc158195159)

[**Supplemental Table 13**. Model selection results for weight-for-age comparing different specifications of random structures and linear fit with- or without a knot positioned at day-45. 34](#_Toc158195160)

[**Supplemental Table 14.** Change in WAZ, LAZ, MUACZ and WLZ in the 6-months post-discharge by WHO nutritional group as estimated with multivariate piecewise mixed models built to assess differences between early and late post-discharge periods. 37](#_Toc158195161)

[**Supplemental Figure 4.** Forest plots of coefficients derived from models comparing children from different nutritional groups to measures of community participants 38](#_Toc158195162)

[**Supplemental Figure 5.** Post-discharge length-for-age z-score split by site and nutritional group as classified at admission. 39](#_Toc158195163)

[**Supplemental Figure 6.** Post-discharge weight-for-age z-score split by site and nutritional group as classified at admission. 40](#_Toc158195164)

[**Supplemental Figure 7.** Post-discharge mid-upper arm circumference z-score split by site and nutritional group as classified at admission. 41](#_Toc158195165)

[**Supplemental Figure 8**. Post-discharge weight-for-length z-score split by site and nutritional group as classified at admission. 42](#_Toc158195166)

[**Supplemental Table 15**. Median growth and differences between study visits by nutritional group and in community participants. 43](#_Toc158195167)

[**Supplemental Table 16**. Summary of absolute length-for-age z-score (LAZ) at each timepoint and differences between time points split by nutritional groups and site 45](#_Toc158195168)

[**Supplemental Table 17.** Summary of absolute weight-for-age z-score (WAZ) at each timepoint and differences between time points split by nutritional groups and site. 46](#_Toc158195169)

[**Supplemental Table 18.** Summary of absolute mid upper arm circumference z-score (MUACZ) at each timepoint and differences between time points split by nutritional groups and site. 47](#_Toc158195170)

[**Supplemental Table 19.** Summary absolute of weight-for-length z-score (WLZ) at each timepoint and differences between time points split by nutritional groups and site. 48](#_Toc158195171)

[**Supplemental Figure 9**. Pairwise correlation plot between anthropometric measures 49](#_Toc158195172)

[**Supplemental Figure 10.** Alluvial plots detailing the change in nutritional classification of children from discharge (center) to 180-days post discharge (left) for wasting based on WLZ 50](#_Toc158195173)

[**Supplemental Table 20**. Change in LAZ in the 180-days post-discharge per nutritional group associated with anaemia or diarrhoea diagnosed at admission. 51](#_Toc158195174)

[**Supplemental Table 21**. Change in LAZ in the 180-days post-discharge per nutritional group associated with sepsis or pneumonia diagnosed at admission. 52](#_Toc158195175)

[**Supplemental Table 22**. Change in LAZ in the 180-days post-discharge per nutritional group associated with exposure domains of illness severity at admission and at discharge. 53](#_Toc158195176)

[**Supplemental Table 23**. Change in LAZ in the 180-days post-discharge per nutritional group associated with exposure domains of age-inappropriate nutrition and caregiver characteristics. 54](#_Toc158195177)

[**Supplemental Table 24**. Change in LAZ in the 180-days post-discharge per nutritional group associated with exposure domains of household-level exposures and access to health care. 55](#_Toc158195178)

[**Supplemental Table 25**. Change in LAZ in the 180-days post-discharge per nutritional group associated with HIV and small birth size. 56](#_Toc158195179)

[**Supplemental Table 26**. Change in LAZ in the 180-days post-discharge per nutritional group associated with chronic medical conditions and prior hospitalisation. 57](#_Toc158195180)

[**Supplemental Table 27**. Change in WAZ in the 180-days post-discharge per nutritional group associated with anaemia or diarrhoea diagnosed at admission. 58](#_Toc158195181)

[**Supplemental Table 28**. Change in WAZ in the 180-days post-discharge per nutritional group associated with sepsis or pneumonia diagnosed at admission. 59](#_Toc158195182)

[**Supplemental Table 29**. Change in WAZ in the 180-days post-discharge per nutritional group associated with exposure domains of illness severity at admission and at discharge. 60](#_Toc158195183)

[**Supplemental Table 30**. Change in WAZ in the 180-days post-discharge per nutritional group associated with exposure domains of age-inappropriate nutrition and caregiver characteristics. 61](#_Toc158195184)

[**Supplemental Table 31**. Change in WAZ in the 180-days post-discharge per nutritional group associated with exposure domains of household-level exposures and access to health care. 62](#_Toc158195185)

[**Supplemental Table 32**. Change in WAZ in the 180-days post-discharge per nutritional group associated with HIV and small birth size. 63](#_Toc158195186)

[**Supplemental Table 33**. Change in WAZ in the 180-days post-discharge per nutritional group associated with chronic medical conditions and prior hospitalisation. 64](#_Toc158195187)

[**Supplemental Table 34**. Change in MUACZ in the 180-days post-discharge per nutritional group associated with anaemia or diarrhoea diagnosed at admission. 65](#_Toc158195188)

[**Supplemental Table 35**. Change in MUACZ in the 180-days post-discharge per nutritional group associated with sepsis or pneumonia diagnosed at admission. 66](#_Toc158195189)

[**Supplemental Table 36**. Change in MUACZ in the 180-days post-discharge per nutritional group associated with exposure domains of illness severity at admission and at discharge. 67](#_Toc158195190)

[**Supplemental Table 37**. Change in MUACZ in the 180-days post-discharge per nutritional group associated with exposure domains of age-inappropriate nutrition and caregiver characteristics. 68](#_Toc158195191)

[**Supplemental Table 38**. Change in MUACZ in the 180-days post-discharge per nutritional group associated with exposure domains of household-level exposures and access to health care 69](#_Toc158195192)

[**Supplemental Table 39**. Change in MUACZ in the 180-days post-discharge per nutritional group associated with HIV and small birth size. 70](#_Toc158195193)

[**Supplemental Table 40**. Change in WAZ in the 180-days post-discharge per nutritional group associated with chronic medical conditions and prior hospitalisation. 71](#_Toc158195194)

[**Supplemental Table 41**. Change in WLZ in the 180-days post-discharge per nutritional group associated with anaemia or diarrhoea diagnosed at admission. 72](#_Toc158195195)

[**Supplemental Table 42**. Change in WLZ in the 180-days post-discharge per nutritional group associated with sepsis or pneumonia diagnosed at admission. 73](#_Toc158195196)

[**Supplemental Table 43**. Change in WLZ in the 180-days post-discharge per nutritional group associated with exposure domains of illness severity at admission and at discharge 74](#_Toc158195197)

[**Supplemental Table 44**. Change in WLZ in the 180-days post-discharge per nutritional group associated with exposure domains of age-inappropriate nutrition and caregiver characteristics. 75](#_Toc158195198)

[**Supplemental Table 45**. Change in WLZ in the 180-days post-discharge per nutritional group associated with exposure domains of household-level exposures and access to health care. 76](#_Toc158195199)

[**Supplemental Table 46**. Change in WLZ in the 180-days post-discharge per nutritional group associated with HIV and small birth size 77](#_Toc158195200)

[**Supplemental Table 47**. Change in WLZ in the 180-days post-discharge per nutritional group associated with chronic medical conditions and prior hospitalisation. 78](#_Toc158195201)

[**Supplemental Table 48**. Final multivariable models presenting differences in WAZ, LAZ, MUACZ and WLZ in the 180-days post-discharge per nutritional group associated with variables selected as showing an association with growth 79](#_Toc158195202)

[**Supplemental Table 49**. Final multivariable models presenting differences in WAZ, LAZ, MUACZ and WLZ in the 180-days post-discharge per nutritional strata associated with variables selected as showing an association with growth 80](#_Toc158195203)

[**Supplemental Figure 11**. Growth trajectory of children admitted to hospital with acute illness split by nutritional groups and by latent domains constructed to represent illness severity at admission, illness severity at discharge, and age-inappropriate nutrition 81](#_Toc158195204)

[**Supplemental Figure 12**. Growth trajectory of children admitted to hospital with acute illness split by nutritional groups and by latent domains constructed to represent caregiver characteristics, household-level exposures, and access to health care 82](#_Toc158195205)

[**Supplemental Figure 13**. Growth trajectory of children admitted to hospital with acute illness split by nutritional groups and by additional clinical variables. 83](#_Toc158195206)

[**Supplemental Figure 14**. Growth trajectory of children admitted to hospital with acute illness split by nutritional groups and by additional clinical variables including chronic medical conditions, and prior hospitalisation 84](#_Toc158195207)

[**Supplemental Table 50**. Counts and percentages of children classified within each level of latent domain variables split by nutritional group 85](#_Toc158195208)

[**Supplemental Figure 15**. Forest plots of coefficients present the fixed effects of the final multivariable models with or without further adjustment for caregiver stunting and caregiver BMI 86](#_Toc158195209)

## Supplemental Methods: Ethical approvals

The study was approved by the following ethical committees representing all recruiting and coordinating countries:

- UK: Oxford Tropical Research Ethics Committee
- Kenya: Scientific & Ethical Review Unit (SERU), Kenya Medical Research Institute
- USA: University of Washington Institutional Review Board; Oregon Health and Science University Institutional Review Board
- Uganda: Makerere University School of Biomedical Sciences Research Ethics Committee
- Pakistan: Ethical Review Board, Aga Khan University
- Bangladesh: International Centre for Diarrheal Disease Research: Research Review Committee (RRC) and Ethical Review Committee (ERC)
- Malawi: COMREC, Kamuzu University of Health Sciences, Malawi
- Burkina Faso: Comité d’éthique institutionnel du Centre MURAZ
- Canada: Research Ethics Board of the Hospital for Sick Children
- The Netherlands Medical Ethics Review Committee, Amsterdam UMC, The Netherlands

## Supplemental Methods: Data cleaning

#### Detection of implausible values

Implausible or unlikely growth measures for weight, height, weight-for-age z-score (WAZ), length-for-age z-score (LAZ), mid-upper arm circumference z-score (MUACZ) and weight-for-length z-score (WLZ) where flagged to be checked based on:

1. Being ±3 standard deviations from the mean of other participants within the child’s attributed recruitment strata and age group (≥12 months, or < 12 months). This was done at each timepoint.
2. Showing a change in anthropometry between timepoints that exceeds ±3 standard deviations of mean change calculated within each recruitment strata and age group (≥12 months, or < 12 months). Standardized scores for differences in anthropometry between each timepoints were evaluated for each pair of timepoints (i.e., admission vs. discharge or 45-days, 90-days, 180-days; discharge vs. 45-days, 90-days, 180-days; 45-days vs. 90-days, 180-days; and 90-days vs 180-days).
3. Showing a drop in height between any pair of timepoints of more than 1.5 cm.
4. Being flagged as an extreme multivariate outlier based on robust Mahalanobis distance with shrinkage estimators applied to age, height, weight, and MUAC at each timepoint using the “mvoutlier” R package.^1^

Collectively, these procedures flagged at total of 993 children with at least one possible outlier in any of their anthropometric scores across all timepoints (No wasting, n=356; Moderate wasting, n=242; Severe wasting or nutritional oedema, n=357; Community participants, n=38). Each flagged value was individually evaluated for biological plausibility and were only removed if judged to be inconsistent with the child’s overall growth pattern.

Large drops in weight were contextualized using clinical data at follow ups and deemed plausible if illness or fever was reported, or if coherent with MUAC measurements. Height values were evaluated considering the monotonic pattern of linear growth and cut-offs for implausible growth (i.e., for infants 2-6 months at enrolment, increase in length by >10 cm between discharge and 45-days or 90-days and >10 cm increase between 90-days and 180-days; for infants > 6 months at enrolment, increase in length by >6 cm between discharge and 45-days or 90-days and >10 cm increase between 90-days and 180-days).

Out of all possible anthropometric values (n=19,776), a total of 55 (0.2%) were classified as implausible and either replaced with appropriate surrogate values (e.g. discharge height replaced with that of admission) or removed. Detailed treatment of replaced or removed values are presented in **Supplemental Table 1**. ·

### **Supplemental Table 1**. Tally of implausible values for each anthropometric measure split by recruitment group and timepoint.

|  | Variable | Time point | n | Action |  |  | Variable | Time point | n | Action |
| --- | --- | --- | --- | --- | --- | --- | --- | --- | --- | --- |
| **NW** | Length, LAZ, WLZ | Discharge | 5 | replaced |  | **SW** | Length, LAZ, WLZ | Discharge | 2 | replaced |
|  |  |  | 0 | removed |  |  |  |  | 0 | removed |
|  |  | 45-days | 14 | removed |  |  |  | 45-days | 0 | removed |
|  |  | 90-days | 3 | removed |  |  |  | 90-days | 1 | removed |
|  |  | 180-days | 2 | removed |  |  |  | 180-days | 0 | removed |
|  | Weight and WAZ, WLZ | Discharge | 1 | replaced |  |  | Weight and WAZ, WLZ | Discharge | 0 | replaced |
|  |  |  | 0 | removed |  |  |  |  | 0 | removed |
|  |  | 45-days | 0 | removed |  |  |  | 45-days | 0 | removed |
|  |  | 90-days | 0 | removed |  |  |  | 90-days | 0 | removed |
|  |  | 180-days | 2 | removed |  |  |  | 180-days | 0 | removed |
|  | MUAC and MUACZ | Discharge | 0 | replaced |  |  | MUAC and MUACZ | Discharge | 0 | replaced |
|  |  |  | 5 | removed |  |  |  |  | 0 | removed |
|  |  | 45-days | 1 | removed |  |  |  | 45-days | 0 | removed |
|  |  | 90-days | 3 | removed |  |  |  | 90-days | 0 | removed |
|  |  | 180-days | 1 | removed |  |  |  | 180-days | 0 | removed |
| **MW** | Length, LAZ, WLZ | Discharge | 5 | replaced |  | **NO** | Length, LAZ, WLZ | Discharge | 3 | replaced |
|  |  |  | 0 | removed |  |  |  |  | 0 | removed |
|  |  | 45-days | 4 | removed |  |  |  | 45-days | 1 | removed |
|  |  | 90-days | 4 | removed |  |  |  | 90-days | 0 | removed |
|  |  | 180-days | 2 | removed |  |  |  | 180-days | 0 | removed |
|  | Weight and WAZ, WLZ | Discharge | 1 | replaced |  |  | Weight and WAZ, WLZ | Discharge | 0 | replaced |
|  |  |  | 0 | removed |  |  |  |  | 0 | removed |
|  |  | 45-days | 1 | removed |  |  |  | 45-days | 2 | removed |
|  |  | 90-days | 0 | removed |  |  |  | 90-days | 0 | removed |
|  |  | 180-days | 2 | removed |  |  |  | 180-days | 1 | removed |
|  | MUAC and MUACZ | Discharge | 0 | replaced |  |  | MUAC and MUACZ | Discharge | 0 | replaced |
|  |  |  | 3 | removed |  |  |  |  | 0 | removed |
|  |  | 45-days | 0 | removed |  |  |  | 45-days | 0 | removed |
|  |  | 90-days | 4 | removed |  |  |  | 90-days | 1 | removed |
|  |  | 180-days | 0 | removed |  |  |  | 180-days | 0 | removed |
|  |  |  |  |  |  | **CP** | Length, LAZ, WLZ | - | 1 | removed |
|  |  |  |  |  |  |  | Weight and WAZ, WLZ | - | 1 | removed |
|  |  |  |  |  |  |  | MUAC and MUACZ | - | 0 | removed |

Implausible values were replaced by appropriate surrogate value or removed. Groups: NW, no wasting; MW, moderate wasting; SW, severe wasting; NO, nutritional oedema; CP, community participants. LAZ, length-for-age z-score; WAZ, weight-for-age z-score; MUAC, mid-upper arm circumference; MUACZ, mid-upper arm circumference z-score; WLZ, weight-for-length z-score.

#### Replacement of missing discharge anthropometry

If anthropometry was missing at discharge, values at admission were used for weight if duration of hospital stay was ≤ 2 days, and length was replaced by admission values if duration of hospital stay was less than < 20 days. If discharge values were missing and child had long hospital stay, values were replaced by readmission data, if available and if within ≤ 2 days of discharge.

### **Supplemental Table 2**. Tally of missing discharge anthropometry replaced by admission or readmission values presented split by recruitment groups.

| Groups | n | Action |  |  |  |  |
| --- | --- | --- | --- | --- | --- | --- |
| NW | 2 | anthropometry replaced with admission | | |  |  |
|  |  |  |  |  |  |  |
| MW | 1 | anthropometry replaced with admission | | |  |  |
|  |  |  |  |  |  |  |
| SW | 1 | replaced by readmission (2 days after absconding) | | | | |
|  | 1 | replaced by admission | | |  |  |
|  | 1 | length replaced by admission (length of stay, 6 days) | | |  |  |
| NO | 1 | length replaced by admission (length of stay, 19 days) | | | | |

Groups: NW, no wasting; MW, moderate wasting; SW, severe wasting; NO, nutritional oedema.

#### Missing dates at discharge

If anthropometry was available at discharge but that the date of discharge was missing; it was replaced by the date where the child was indicated as having left hospital or was calculated based on the last available date from daily clinical reviews.

#### Failed classification into WHO nutritional groups

Children were classified into nutritional groups based on criteria defined by the World Health Organization (WHO) which for children under 6 months requires length measures at admission. Four children under the age of 6 months, failed to classify based on strict WHO criteria:

- Three were missing height: These children were attributed to nutritional groups based on MUAC (two children were classified as NW (a death case and a lost-to-follow-up indicated in Study Flow chart). These children were by inclusion/exclusion criteria not included in the main analysis. The other child was a community participant close to 6 months of age who was attributed to MW.
- One child was unclassified since their length was under 45 cm. WLZ are not calculated based on WHO growth standards, since this height value is considered implausible. Given our population of study and that the child’s length was coherent across time points, this child was classified as severely wasted based on their MUAC.

### **Supplemental Table 3.** Tally of missing anthropometry and proportion of missingness for each measure.

Tally of missing values for children that survived until discharge split by group and community participants (left column) and in children with either known survival status 6-months after discharge split by group or being lost-to-follow up (right column). * As per WHO growth standards, MUACZ was only calculated in children >3 months; there were 93 children who had missing MUACZ values at discharge due to being younger than 3 months at that time and MUACZ of 54 community participants were missing due to young age. Groups: NW, no wasting; MW, moderate wasting; SW, severe wasting; NO, nutritional oedema; CP, community participants. Metric: LAZ, length-for-age z-score; WAZ, weight-for-age z-score; MUAC, mid-upper arm circumference; MUACZ, mid-upper arm circumference z-score; WLZ, weight-for-length z-score.

### **Supplemental Table 4.** Follow-up visits outside planned 14-day window split per group and timepoint in children included in the main analysis.

| Groups | n | **Day 45** | | |  | **Day 90** | | |  | **Day 180** | | | |
| --- | --- | --- | --- | --- | --- | --- | --- | --- | --- | --- | --- | --- | --- |
|  |  | Early | Late | Out |  | Early | Late | Out |  | Early | Late* | Out |  |
| NW | 960 | 5 | 45 | 50 (5·2%) |  | 12 | 57 | 69 (7·2%) |  | 20 | 39 | 59 (6·1%) |  |
| MW | 572 | 0 | 35 | 35 (6·1%) |  | 4 | 29 | 33 (5·8%) |  | 6 | 32 | 38 (6·6%) |  |
| SW | 682 | 1 | 43 | 44 (6·6%) |  | 5 | 24 | 29 (4·3%) |  | 9 | 32 | 41 (6·0%) |  |
| NO | 258 | 2 | 13 | 15 (5·8%) |  | 8 | 11 | 19 (7·4%) |  | 11 | 9 | 22 (8·5%) |  |
| **Total** | **2472** | 8 | 136 | 144 (5·8%) |  | 29 | 121 | 150 (6·1%) |  | 46 | 112 | 158 (6·4%) |  |

Data presented as counts and percentages. *Late window for 180-days timepoint was 30 days. Groups: NW, no wasting; MW, moderate wasting; SW, severe wasting; NO, nutritional oedema; CP, community participants.

### **Supplemental Table 5**. List of base models and alternative random structures assessed for each anthropometric measure.

| **Model** | | **Formula** | **Knot** | **Characteristics** |
| --- | --- | --- | --- | --- |
| 1 | ~ | time * group+ age + sex +   (time\| site/record_id) | None/linear | Random slope per participants nested within site |
| 2 | ~ | time * group+ age + sex +  (1 \| site) + (time\| record_id) | None/linear | Random slope per participants; random intercept for site  (patient nesting within intercept implicit from data structure |
| 3 | ~ | time * group+ age + sex + (1 \| site/record_id) | None/linear | Random intercept only (patient nesting within site) |
| 4 | ~ | (time + I(pmax(time-1.5, 0)) ) * group + age + sex +   ((time + I(pmax(time-1.5, 0)) )\| site/record_id) | Day-45 | Random slope per participants nested within site |
| 5 | ~ | (time + I(pmax(time-1.5, 0)) ) * group + age + sex +  (1 \| site) + ((time + I(pmax(time-1.5, 0)) )\| record_id) | Day-45 | Random slope per participants; random intercept for site  (patient nesting within intercept implicit from data structure |
| 6 | ~ | (time + I(pmax(time-1.5, 0)) ) * group + age + sex +  (1 \| site/record_id) | Day-45 | Random intercept only (patient nesting within site) |
| 4 | ~ | (time + I(pmax(time-3, 0)) ) * group + age + sex +   ((time + I(pmax(time-3, 0)) )\| site/record_id) | Day-90 | Random slope per participants nested within site |
| 5 | ~ | (time + I(pmax(time-3, 0)) ) * group + age + sex +  (1 \| site) + ((time + I(pmax(time-3, 0)) )\| record_id) | Day-90 | Random slope per participants; random intercept for site  (patient nesting within intercept implicit from data structure |
| 6 | ~ | (time + I(pmax(time-3, 0)) ) * group + age + sex +  (1 \| site/record_id) | Day-90 | Random intercept only (patient nesting within site) |

Linear and piece-wise mixed effect models were fit using *lme4* R package (Version 1·1-33). Knots position at either Day-45 or Day-90 were evaluated. Time was coded in months (i.e., 1·5 months corresponds to 45-days; and 3 months to 90-days). Models were fit with maximum likelihood for comparison with anova using Satterthwaite's approximation of degrees of freedom as implemented in the *lmerTest* R package (Kuznetsova, A. et al., 2017). Restricted maximum likelihood was used to fit final models. This selection procedure was performed for each anthropometric measure (i.e., z-scores for height-for-age, weight-for-age, mid-upper arm circumference and weight-for-length). Parsimonious and consistent model structure across metrics were considered when choosing the final model. Models were evaluated using fit metrics AIC, AICc, and BIC and the *performance* R package (version 0·10·4).

### **Supplemental Table 6**. List of R packages and version used.

|  | **R packages used** | Version |  |  | **R packages used** | Version |
| --- | --- | --- | --- | --- | --- | --- |
| 1 | tidyverse | 2·0·0 |  | 25 | mosaic | 1·8·4·2 |
| 2 | here | 1·0·1 |  | 26 | venn | 1·11 |
| 3 | mgsub | 1·7·3 |  | 27 | anthro | 1·0·0 |
| 4 | gt | 0·9·0 |  | 28 | lavaan | 0·6-17 |
| 5 | gtsummary | 1·7·1 |  | 29 | lubridate | 1·9·2 |
| 6 | ggpubr | 0·6·0 |  | 30 | kableExtra | 1·3·4 |
| 7 | ggfan | 0·1·3 |  | 31 | summarytools | 1·0·1 |
| 8 | lme4 | 1·1-33 |  | 32 | outliers | 0·15 |
| 9 | lmerTest | 3·1-3 |  | 33 | visdat | 0·6·0 |
| 10 | emmeans | 1·8·7 |  | 34 | mvoutlier | 2·1·1 |
| 11 | sjplot | 2·8·14 |  | 35 | FactoMineR | 2·8 |
| 12 | janitor | 2·2·0 |  | 36 | stringr | 1·5·0 |
| 13 | naniar | 1·0·0 |  |  |  |  |
| 14 | corrplot | 0·92 |  |  |  |  |
| 15 | mice | 3·16·0 |  |  |  |  |
| 16 | rsvg | 2·5·0 |  |  |  |  |
| 17 | ggalluvial | 0·12·5 |  |  |  |  |
| 18 | rstatix | 0·7·2 |  |  |  |  |
| 19 | RColorBrewer | 1·1-3 |  |  |  |  |
| 20 | ggplot2 | 3·4·2 |  |  |  |  |
| 21 | performance | 0·10·4 |  |  |  |  |
| 22 | multcomp | 1·4-25 |  |  |  |  |
| 23 | AICCmodavg | 2·3-3 |  |  |  |  |
| 24 | pacman | 0·5·1 |  |  |  |  |
|  |  |  |  |  |  |  |

## Supplemental Results


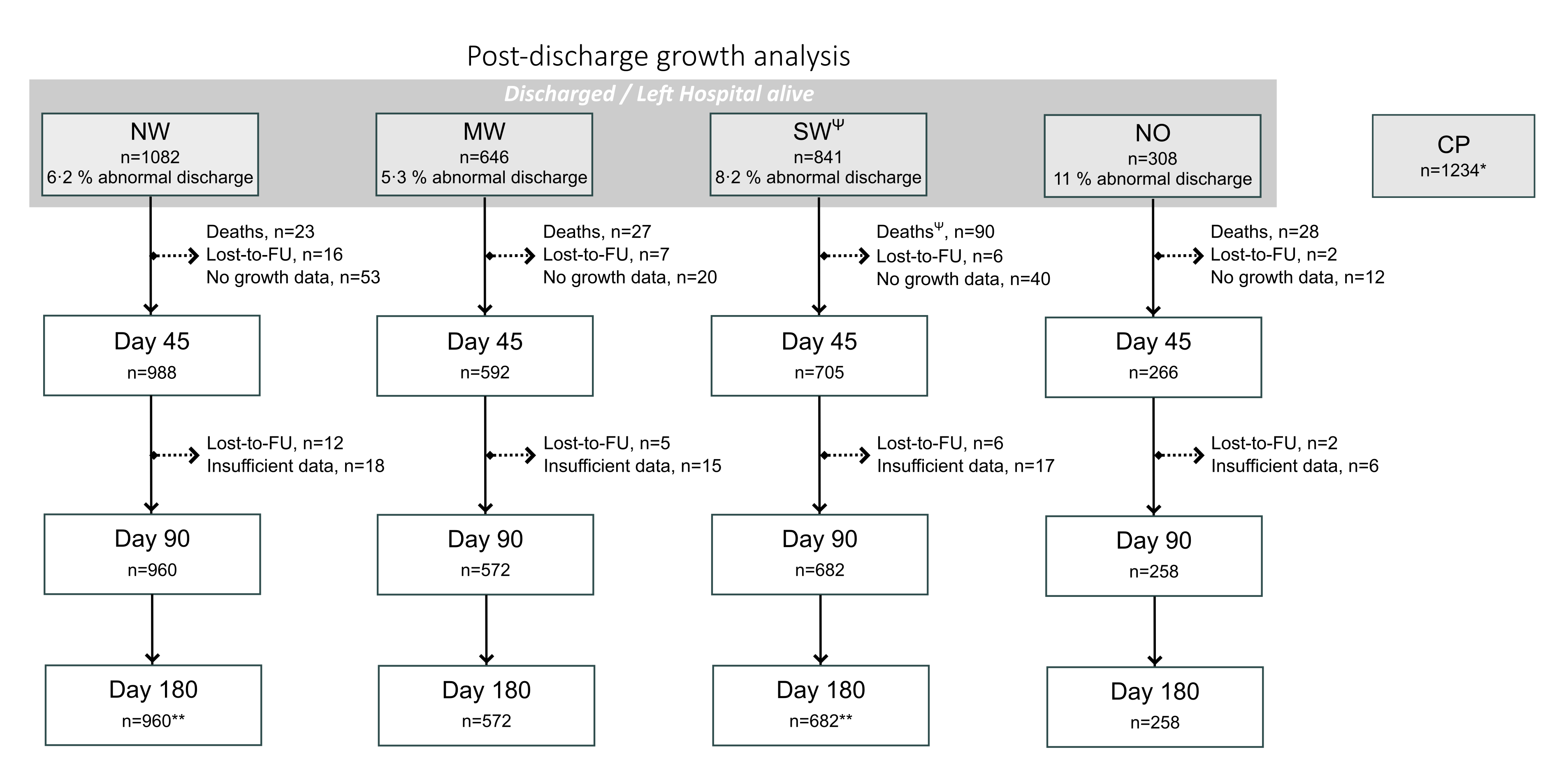


**Supplemental Figure 1.** Flow chart of study participants. Participants (n=3101) were enrolled at admission based on mid-upper arm circumference (MUAC) and classified into 4 groups: NW, MW, SW and NO (Defined in **Table 1**). Of these 182 patients died in hospital and 42 families dropped from the study prior to discharge. 2877 study participants were discharged (NW, n=1082; MW, n=646; SW, n=841; NO, n=308). Of these children, 168 died post-discharge and 237 had no or insufficient data. Thus, the main analysis included 2472 children. Community participants (n=1234) were also recruited to provide community norm values. Ψ, includes a child with height < 45 cm [age < 6months] that died which was classified as SW based on MUAC.*, includes a case age < 6months classified as MW based on MUAC. **, includes cases of lost-to-follow-up that had sufficient growth data (NW, n=8; SW, n=1). Groups: NW, no wasting; MW, moderate wasting; SW, severe wasting; NO, nutritional oedema; CP, community participants.

### **Supplemental Table 7.** Comparison of clinical and sociodemographic characteristics between children discharged after acute illness with sufficient (included) vs. insufficient (excluded) growth data post-discharge presented split by nutritional group.

**Supplemental Table 7.** Continued

**Supplemental Table 7.** Continued

**Supplemental Table 7.** Continued

**Supplemental Table 7.** Continued

Results presented as Frequency (%) or Median (IQR). Differences between included versus excluded children tested with either Fisher’s exact for categorical or Wilcoxon rank sum test for continuous variables. Excluded children are those who died post-discharge (n=168) or who had no or insufficient growth data (n=237). ^Ψ^, cough or difficulty breathing with oxygen saturation <90%, central cyanosis, or grunting; very severe chest indrawing or inability to breastfeed or drink; or lethargy, reduced level of consciousness, or convulsions. ^¥^, anaemia: none, haemoglobin >110 g/L; mild 100–110 g/L; moderate/severe, <100 g/L. ^λ^, blood glucose <3 mmol/L or >10 mmol/L. ^Ø^, reported premature or low birthweight (<2·5 kg). ^ϛ^, includes thalassemia, cerebral palsy, sickle cell disease, congenital cardiac diseases and known tuberculosis. ^ℸ^, recommended adequate diet: for age < 6 months, exclusive breastfeeding; for age 6-9 months, consuming ≥ 2 food groups and breastmilk; for age 10-23 months, consuming ≥ 4 food groups and breastmilk. *, indicates metrics that can be influenced by fluid retention; MUAC is considered less sensitive to oedema than other weight-based metrics. Groups: NW, no wasting; MW, moderate wasting; SW, severe wasting; NO, nutritional oedema. Growth metrics: LAZ, length-for-age z-score; WAZ, weight-for-age z-score; MUAC, mid-upper arm circumference; MUACZ, mid-upper arm circumference z-score; WLZ, weight-for-length z-score. Significance threshold, p<0·05.


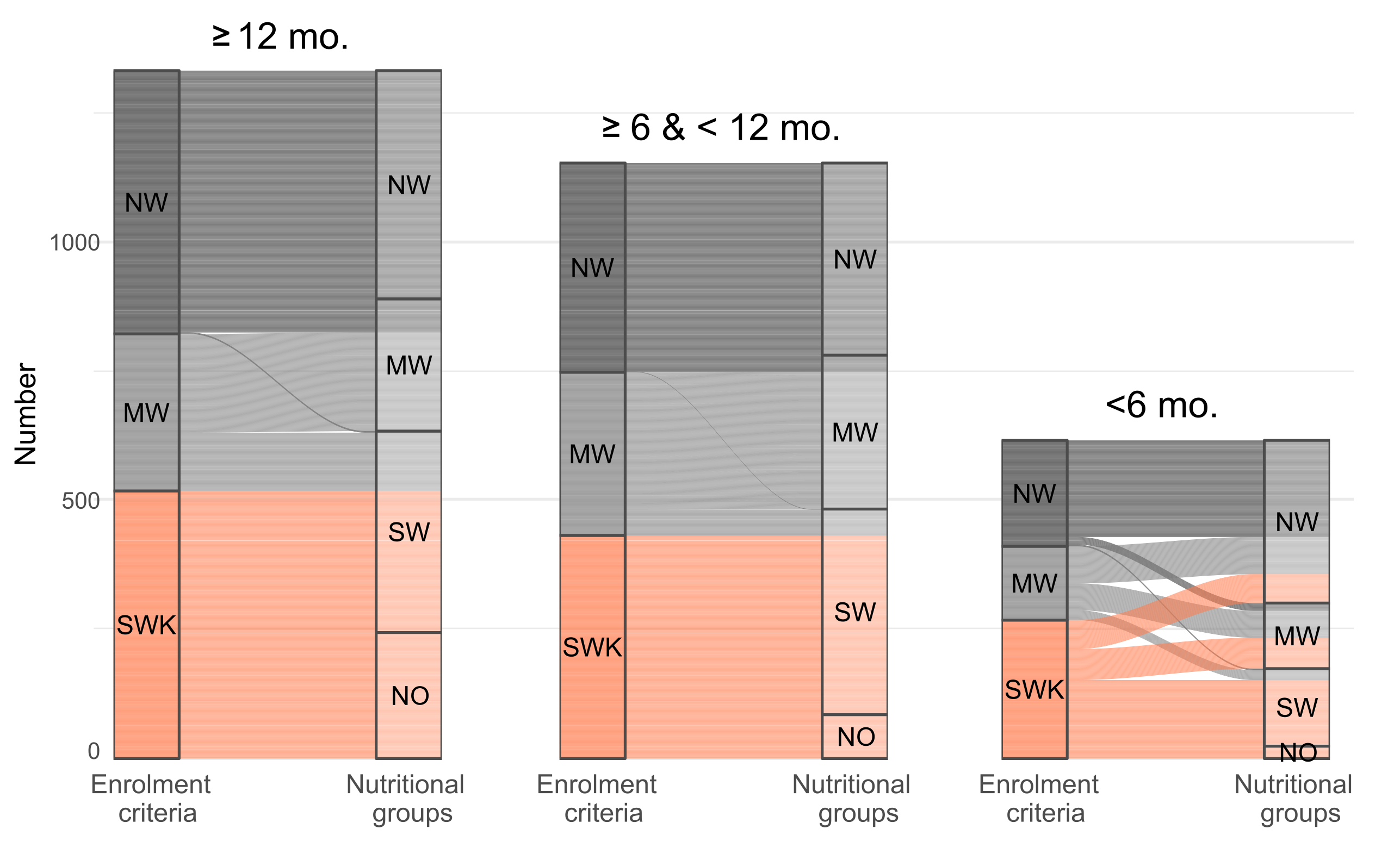


|  | **Enrolment criteria** | | | **Overall** n = 2472 |
| --- | --- | --- | --- | --- |
|  | **NW**  n = 957 | **MW** n = 641 | **SWK** n = 874 |  |
| **Nutritional groups** |  |  |  |  |
| NW | 855 (89%) | 58 (9·0%) | 47 (5·4%) | 960 (39%) |
| MW | 97 (10%) | 427 (67%) | 48 (5·5%) | 572 (23%) |
| SW | 5 (0·5%) | 156 (24%) | 521 (60%) | 682 (28%) |
| NO | 0 (0%) | 0 (0%) | 258 (30%) | 258 (10%) |

**Supplemental Figure 2.** Alluvial plot and associated table presenting the redistribution of children grouped by enrolment criteria compared to study classification by nutritional groups at admission. For the post-discharge growth analysis, children were reclassified based on World Health Organisation nutritional groups since this classification determined inpatient treatment and participation in nutritional programs post-discharge. The group cross-over are presented split by age in months as indicated above each alluvial. Groups: NW, no wasting; MW, moderate wasting; SW, severe wasting; NO, nutritional oedema; SWK, severe wasting based on mid-upper arm criteria only or having nutritional oedema.

### **Supplemental Table 8.** Participant characteristics at admission by nutritional group for Banfora, and Blantyre.

|  | **Banfora** | | | |  | **Blantyre** | | | |
| --- | --- | --- | --- | --- | --- | --- | --- | --- | --- |
|  | NW | MW | SW | NO |  | NW | MW | SW | NO |
|  | n=118 | n=87 | n=113 | n=30 |  | n=138 | n=37 | n=40 | n=20 |
| **Demographics** |  |  |  |  |  |  |  |  |  |
| Age |  |  |  |  |  |  |  |  |  |
| ≥*12 mo.* | 62 (53%) | 50 (57%) | 68 (60%) | 27 (90%) |  | 69 (50%) | 14 (38%) | 18 (45%) | 18 (90%) |
| ≥*6 & <12 mo.* | 35 (30%) | 27 (31%) | 31 (27%) | 3 (10%) |  | 40 (29%) | 21 (57%) | 19 (48%) | 2 (10%) |
| *<6 mo.* | 21 (18%) | 10 (11%) | 14 (12%) | 0 (0%) |  | 29 (21%) | 2 (5·4%) | 3 (7·5%) | 0 (0%) |
| Sex, male | 75 (64%) | 44 (51%) | 71 (63%) | 16 (53%) |  | 78 (57%) | 18 (49%) | 22 (55%) | 10 (50%) |
| **Clinical presentation at admission** |  |  |  |  |  |  |  |  |  |
| Sepsis | 3 (2·5%) | 0 (0%) | 5 (4·4%) | 0 (0%) |  | 44 (32%) | 16 (43%) | 9 (22%) | 7 (35%) |
| Severe pneumonia ^Ψ^ | 20 (17%) | 13 (15%) | 16 (14%) | 1 (3·3%) |  | 16 (12%) | 7 (19%) | 1 (2·5%) | 0 (0%) |
| Impaired consciousness | 5 (4·2%) | 9 (10%) | 6 (5·3%) | 2 (6·7%) |  | 1 (0·7%) | 0 (0%) | 0 (0%) | 0 (0%) |
| Diarrhoea | 48 (41%) | 39 (45%) | 46 (41%) | 15 (50%) |  | 44 (32%) | 20 (54%) | 21 (52%) | 12 (60%) |
| Malaria, positive | 60 (51%) | 59 (68%) | 54 (48%) | 14 (47%) |  | 22 (16%) | 2 (5·7%) | 1 (2·7%) | 4 (20%) |
| Anemia^¥^ |  |  |  |  |  |  |  |  |  |
| *none* | 6 (5·1%) | 6 (7·1%) | 9 (8·0%) | 2 (6·9%) |  | 51 (42%) | 7 (23%) | 10 (31%) | 3 (19%) |
| *mild* | 11 (9·3%) | 6 (7·1%) | 9 (8·0%) | 1 (3·4%) |  | 28 (23%) | 7 (23%) | 6 (19%) | 4 (25%) |
| *moderate/severe* | 101 (86%) | 73 (86%) | 95 (84%) | 26 (90%) |  | 41 (34%) | 16 (53%) | 16 (50%) | 9 (56%) |
| Blood glucose, abnormal ^λ^ | 8 (6·8%) | 5 (5·7%) | 5 (4·4%) | 2 (6·7%) |  | 7 (5·2%) | 2 (5·6%) | 2 (5·1%) | 0 (0%) |
| **Anthropometry at admission** |  |  |  |  |  |  |  |  |  |
| LAZ | -0·8 (-1·4, 0·0) | -1·2 (-1·8, -0·4) | -2·6 (-3·5, -1·8) | -2·3 (-2·9, -1·6) |  | -1·4 (-2·2, -0·5) | -2·4 (-3·2, -1·6) | -3·3 (-4·6, -2·1) | -3·4 (-3·8, -3·0) |
| WAZ* | -1·1 (-1·5, -0·4) | -2·1 (-2·7, -1·7) | -3·9 (-5·1, -3·3) | -2·7 (-3·5, -1·9) |  | -0·9 (-1·7, -0·1) | -2·8 (-3·3, -2·3) | -4·1 (-4·9, -3·6) | -3·5 (-3·8, -2·2) |
| MUAC*, cm | 13·4 (13·0, 13·9) | 12·2 (12·0, 12·4) | 10·8 (9·8, 11·3) | 11·4 (10·8, 12·4) |  | 13·9 (13·2, 14·8) | 12·3 (11·9, 12·4) | 11·1 (10·2, 11·4) | 11·9 (10·9, 12·4) |
| MUACZ* | 1·0 (-1·3, -0·43) | -2·0 (-2·4, -1·8) | -3·7 (-4·7, -3·2) | -3·2 (-3·8, -2·3) |  | -0·40 (-1·1, 0·27) | -2·0 (-2·4, -1·7) | -3·5 (-4·3, -2·9) | -2·8 (-4·0, -2·1) |
| WLZ* | -0·8 (-1·5, -0·2) | -2·3 (-2·6, -1·8) | -3·8 (-4·5, -3·2) | -2·2 (-3·2, -1·3) |  | -0·2 (-0·8, 0·6) | -2·0 (-2·5, -1·6) | -3·6 (-4·3, -3·0) | -2·2 (-2·8, -1·2) |
| **Underlying chronic conditions** |  |  |  |  |  |  |  |  |  |
| Stunted |  |  |  |  |  |  |  |  |  |
| *none* | 102 (86%) | 68 (78%) | 35 (31%) | 13 (43%) |  | 94 (68%) | 15 (41%) | 10 (25%) | 3 (15%) |
| *moderate* | 12 (10%) | 14 (16%) | 35 (31%) | 10 (33%) |  | 26 (19%) | 9 (24%) | 8 (20%) | 2 (10%) |
| *severe* | 4 (3·4%) | 5 (5·7%) | 43 (38%) | 7 (23%) |  | 18 (13%) | 13 (35%) | 22 (55%) | 15 (75%) |
| Small birth size | 12 (10%) | 7 (8·1%) | 22 (20%) | 3 (10%) |  | 19 (14%) | 13 (36%) | 7 (18%) | 4 (20%) |
| HIV status |  |  |  |  |  |  |  |  |  |
| *unexposed, uninfected* | 118 (100%) | 85 (98%) | 111 (98%) | 30 (100%) |  | 103 (75%) | 20 (54%) | 25 (62%) | 12 (60%) |
| *exposed, uninfected* | 0 (0%) | 1 (1·1%) | 0 (0%) | 0 (0%) |  | 28 (20%) | 9 (24%) | 6 (15%) | 4 (20%) |
| *infected* | 0 (0%) | 1 (1·1%) | 2 (1·8%) | 0 (0%) |  | 7 (5·1%) | 8 (22%) | 9 (22%) | 4 (20%) |
| Chronic conditions^ϛ^ | 4 (3·4%) | 5 (5·7%) | 4 (3·5%) | 2 (6·7%) |  | 4 (2·9%) | 3 (8·1%) | 5 (12%) | 0 (0%) |
| Prior hospitalisation | 23 (19%) | 22 (25%) | 19 (17%) | 8 (27%) |  | 33 (24%) | 13 (35%) | 16 (40%) | 5 (25%) |

**Supplemental Table 8.** Continued

**Supplemental Table 8.** Continued

|  | **Banfora** | | | |  | **Blantyre** | | | |
| --- | --- | --- | --- | --- | --- | --- | --- | --- | --- |
|  | NW | MW | SW | NO |  | NW | MW | SW | NO |
|  | n=118 | n=87 | n=113 | n=30 |  | n=138 | n=37 | n=40 | n=20 |
| **Age-inappropriate nutrition** |  |  |  |  |  |  |  |  |  |
| Recommended adequate diet^ℸ^ | 61 (52%) | 43 (50%) | 30 (27%) | 7 (23%) |  | 75 (54%) | 21 (57%) | 22 (55%) | 4 (20%) |
| Reported recent weight loss | 41 (35%) | 62 (71%) | 103 (91%) | 26 (87%) |  | 6 (4·3%) | 14 (38%) | 26 (65%) | 8 (40%) |
| Reported poor feeding | 1 (0·8%) | 3 (3·4%) | 48 (42%) | 9 (30%) |  | 26 (19%) | 10 (27%) | 16 (40%) | 5 (25%) |
| Reported current breastfeeding | 115 (97%) | 83 (95%) | 94 (83%) | 19 (63%) |  | 116 (84%) | 30 (81%) | 31 (78%) | 5 (25%) |
| **Caregiver characteristics** |  |  |  |  |  |  |  |  |  |
| Primary caregiver not biological mother | 0 (0%) | 0 (0%) | 4 (3·5%) | 1 (3·3%) |  | 8 (5·8%) | 3 (8·1%) | 0 (0%) | 1 (5·0%) |
| Caregiver education |  |  |  |  |  |  |  |  |  |
| *secondary/Tertiary* | 13 (11%) | 18 (21%) | 4 (3·5%) | 0 (0%) |  | 65 (49%) | 15 (41%) | 20 (51%) | 1 (5·0%) |
| *primary* | 22 (19%) | 11 (13%) | 14 (12%) | 5 (17%) |  | 62 (46%) | 18 (49%) | 17 (44%) | 18 (90%) |
| *none* | 83 (70%) | 58 (67%) | 95 (84%) | 25 (83%) |  | 7 (5·2%) | 4 (11%) | 2 (5·1%) | 1 (5·0%) |
| Mental health risk, moderate to severe | 17 (15%) | 12 (14%) | 25 (23%) | 4 (16%) |  | 5 (3·6%) | 4 (11%) | 4 (10%) | 1 (5·0%) |
| Employment |  |  |  |  |  |  |  |  |  |
| *employed* | 3 (2·5%) | 7 (8·0%) | 2 (1·8%) | 0 (0%) |  | 25 (19%) | 6 (17%) | 8 (21%) | 4 (21%) |
| *self-employed* | 16 (14%) | 10 (11%) | 15 (13%) | 2 (6·7%) |  | 25 (19%) | 11 (31%) | 8 (21%) | 2 (11%) |
| *no income* | 99 (84%) | 70 (80%) | 96 (85%) | 28 (93%) |  | 82 (62%) | 18 (51%) | 23 (59%) | 13 (68%) |
| **Household-level exposures** |  |  |  |  |  |  |  |  |  |
| Population density, 1000 people per km^2^ | 0·1 (0·0, 0·4) | 0·1 (0·0, 0·2) | 0·1 (0·0, 0·2) | 0·1 (0·0, 0·1) |  | 4·6 (0·8, 8·9) | 3·2 (0·4, 5·9) | 5·0 (1·8, 7·2) | 1·0 (0·2, 5·5) |
| Asset index |  |  |  |  |  |  |  |  |  |
| *least poor* | 8 (6·8%) | 3 (3·4%) | 3 (2·7%) | 0 (0%) |  | 18 (13%) | 8 (22%) | 4 (10%) | 0 (0%) |
| *fourth* | 6 (5·1%) | 4 (4·6%) | 5 (4·4%) | 0 (0%) |  | 16 (12%) | 4 (11%) | 4 (10%) | 0 (0%) |
| *middle* | 22 (19%) | 9 (10%) | 22 (19%) | 5 (17%) |  | 30 (22%) | 5 (14%) | 8 (20%) | 2 (10%) |
| *second* | 52 (44%) | 41 (47%) | 43 (38%) | 16 (53%) |  | 38 (28%) | 9 (24%) | 12 (30%) | 9 (45%) |
| *poorest* | 30 (25%) | 30 (34%) | 40 (35%) | 9 (30%) |  | 36 (26%) | 11 (30%) | 12 (30%) | 9 (45%) |
| Household food insecurity |  |  |  |  |  |  |  |  |  |
| *low* | 110 (93%) | 75 (86%) | 98 (87%) | 26 (87%) |  | 63 (46%) | 14 (38%) | 14 (35%) | 5 (25%) |
| *medium* | 8 (6·8%) | 12 (14%) | 15 (13%) | 4 (13%) |  | 43 (31%) | 12 (32%) | 16 (40%) | 9 (45%) |
| *high* | 0 (0%) | 0 (0%) | 0 (0%) | 0 (0%) |  | 32 (23%) | 11 (30%) | 10 (25%) | 6 (30%) |
| Toilet, not improved | 31 (26%) | 24 (28%) | 37 (33%) | 9 (30%) |  | 78 (57%) | 19 (51%) | 18 (45%) | 14 (70%) |
| Water source, not improved | 22 (19%) | 19 (22%) | 22 (19%) | 4 (13%) |  | 5 (3·6%) | 2 (5·4%) | 1 (2·5%) | 3 (15%) |
|  |  |  |  |  |  |  |  |  |  |
|  |  |  |  |  |  |  |  |  |  |
|  |  |  |  |  |  |  |  |  |  |
|  |  |  |  |  |  |  |  |  |  |
|  |  |  |  |  |  |  |  |  |  |
|  |  |  |  |  |  |  |  |  |  |
| **Supplemental Table 8.** Continued |  |  |  |  |  |  |  |  |  |
| **Supplemental Table 8.** Continued |  |  |  |  |  |  |  |  |  |
|  |  |  |  |  |  |  |  |  |  |
|  | **Banfora** | | | |  | **Blantyre** | | | |
|  | NW | MW | SW | NO |  | NW | MW | SW | NO |
|  | n=118 | n=87 | n=113 | n=30 |  | n=138 | n=37 | n=40 | n=20 |
| **Access to health care** |  |  |  |  |  |  |  |  |  |
| Distance to hospital, km | 35·2 (7·8, 57·6) | 48·5 (13·6, 73·3) | 42·6 (12·7, 63·5) | 44·9 (35·4, 55·4) |  | 6·3 (4·1, 9·3) | 7·1 (4·5, 14·7) | 7·5 (5·1, 8·4) | 7·6 (6·2, 14·3) |
| Distance to nearest health facility, km | 4·8 (1·1, 10·3) | 6·1 (1·0, 11·0) | 5·4 (1·4, 11·6) | 9·9 (2·5, 16·0) |  | 2·6 (1·3, 3·4) | 2·8 (2·2, 3·5) | 2·6 (1·4, 3·9) | 2·8 (1·7, 3·7) |
| Means of travel to hospital |  |  |  |  |  |  |  |  |  |
| *bus, car, ambulance, train* | 17 (15%) | 16 (19%) | 28 (25%) | 6 (20%) |  | 132 (96%) | 36 (100%) | 39 (98%) | 18 (90%) |
| *walking, motorbike, tuktuk, rickshaw* | 99 (85%) | 70 (81%) | 85 (75%) | 24 (80%) |  | 6 (4·3%) | 0 (0%) | 1 (2·5%) | 2 (10%) |
| Travel cost to study hospital |  |  |  |  |  |  |  |  |  |
| *<1$* | 13 (11%) | 9 (11%) | 14 (12%) | 1 (3·3%) |  | 103 (75%) | 24 (65%) | 32 (80%) | 13 (72%) |
| ≥*1 to <5$* | 89 (77%) | 53 (63%) | 80 (71%) | 25 (83%) |  | 33 (24%) | 12 (32%) | 5 (12%) | 4 (22%) |
| ≥ *5$* | 13 (11%) | 22 (26%) | 19 (17%) | 4 (13%) |  | 1 (0·7%) | 1 (2·7%) | 3 (7·5%) | 1 (5·6%) |
| Travel time to study hospital |  |  |  |  |  |  |  |  |  |
| *less than 1hr* | 41 (35%) | 23 (27%) | 28 (25%) | 2 (6·7%) |  | 83 (60%) | 21 (57%) | 28 (70%) | 9 (45%) |
| *between 1hr and 2hr* | 43 (37%) | 18 (21%) | 32 (28%) | 11 (37%) |  | 46 (33%) | 11 (30%) | 10 (25%) | 9 (45%) |
| *2hr or more* | 33 (28%) | 45 (52%) | 53 (47%) | 17 (57%) |  | 9 (6·5%) | 5 (14%) | 2 (5·0%) | 2 (10%) |

Results presented as Frequency (%) or Median (IQR). ^Ψ^, cough or difficulty breathing with oxygen saturation <90%, central cyanosis, or grunting; very severe chest indrawing or inability to breastfeed or drink; or lethargy, reduced level of consciousness, or convulsions. ^¥^, anaemia: none, haemoglobin >110 g/L; mild 100–110 g/L; moderate/severe, <100 g/L. ^λ^, blood glucose <3 mmol/L or >10 mmol/L. ^Ø^, reported premature or low birthweight (<2·5 kg). ^ϛ^, includes thalassemia, cerebral palsy, sickle cell disease, congenital cardiac diseases and known tuberculosis. ^ℸ^, recommended adequate diet: for age < 6 months, exclusive breastfeeding; for age 6-9 months, consuming ≥ 2 food groups and breastmilk; for age 10-23 months, consuming ≥ 4 food groups and breastmilk. *, indicates metrics that can be influenced by fluid retention; MUAC is considered less sensitive to oedema than other weight-based metrics. Groups: NW, no wasting; MW, moderate wasting; SW, severe wasting; NO, nutritional oedema. Growth metrics: LAZ, length-for-age z-score; WAZ, weight-for-age z-score; MUAC, mid-upper arm circumference; MUACZ, mid-upper arm circumference z-score; WLZ, weight-for-length z-score.

### **Supplemental Table 9.** Participant characteristics at admission by nutritional group for Kampala and Kilifi.

|  | **Kampala** | | | |  | **Kilifi** | | | | |
| --- | --- | --- | --- | --- | --- | --- | --- | --- | --- | --- |
|  | NW | MW | SW | NO |  | NW | MW | SW | NO |  |
|  | n=122 | n=82 | n=77 | n=103 |  | n=105 | n=31 | n=47 | n=17 |  |
| **Demographics** |  |  |  |  |  |  |  |  |  |  |
| Age |  |  |  |  |  |  |  |  |  |  |
| ≥*12 mo.* | 47 (39%) | 26 (32%) | 33 (43%) | 76 (74%) |  | 45 (43%) | 17 (55%) | 23 (49%) | 15 (88%) |  |
| ≥*6 & <12 mo.* | 51 (42%) | 50 (61%) | 34 (44%) | 26 (25%) |  | 33 (31%) | 12 (39%) | 17 (36%) | 2 (12%) |  |
| *<6 mo.* | 24 (20%) | 6 (7·3%) | 10 (13%) | 1 (1·0%) |  | 27 (26%) | 2 (6·5%) | 7 (15%) | 0 (0%) |  |
| Sex, male | 66 (54%) | 42 (51%) | 43 (56%) | 58 (56%) |  | 58 (55%) | 16 (52%) | 33 (70%) | 8 (47%) |  |
| **Clinical presentation at admission** |  |  |  |  |  |  |  |  |  |  |
| Sepsis | 51 (42%) | 34 (41%) | 22 (29%) | 32 (31%) |  | 13 (12%) | 2 (6·5%) | 9 (19%) | 2 (12%) |  |
| Severe pneumonia ^Ψ^ | 37 (30%) | 10 (12%) | 11 (14%) | 7 (6·8%) |  | 34 (32%) | 8 (26%) | 11 (23%) | 0 (0%) |  |
| Impaired consciousness | 1 (0·8%) | 1 (1·2%) | 0 (0%) | 1 (1·0%) |  | 9 (8·6%) | 0 (0%) | 3 (6·4%) | 0 (0%) |  |
| Diarrhoea | 60 (49%) | 57 (70%) | 42 (55%) | 30 (29%) |  | 24 (23%) | 13 (42%) | 21 (45%) | 7 (41%) |  |
| Malaria, positive | 12 (9·8%) | 7 (8·5%) | 6 (7·8%) | 8 (7·8%) |  | 19 (19%) | 4 (13%) | 4 (8·5%) | 0 (0%) |  |
| Anemia^¥^ |  |  |  |  |  |  |  |  |  |  |
| *none* | 48 (40%) | 29 (36%) | 25 (33%) | 13 (13%) |  | 12 (12%) | 2 (6·5%) | 5 (11%) | 2 (12%) |  |
| *mild* | 30 (25%) | 15 (19%) | 23 (31%) | 29 (29%) |  | 22 (22%) | 7 (23%) | 9 (20%) | 3 (18%) |  |
| *moderate/Severe* | 41 (34%) | 36 (45%) | 27 (36%) | 59 (58%) |  | 66 (66%) | 22 (71%) | 31 (69%) | 12 (71%) |  |
| Blood glucose, abnormal ^λ^ | 8 (6·6%) | 7 (8·5%) | 0 (0%) | 12 (12%) |  | 6 (5·8%) | 1 (3·2%) | 4 (8·5%) | 1 (5·9%) |  |
| **Anthropometry at admission** |  |  |  |  |  |  |  |  |  |  |
| LAZ | -1·4 (-2·4, -0·5) | -2·0 (-2·7, -1·3) | -3·2 (-4·0, -2·1) | -3·1 (-4·0, -2·2) |  | -0·9 (-1·8, 0·0) | -1·8 (-2·1, -1·1) | -2·7 (-3·9, -1·1) | -2·6 (-3·1, -2·2) |  |
| WAZ* | -1·2 (-1·9, -0·4) | -2·5 (-3·1, -2·1) | -4·3 (-5·0, -3·6) | -3·0 (-4·1, -2·0) |  | -1·0 (-1·7, -0·1) | -2·7 (-3·0, -2·4) | -4·0 (-4·9, -3·5) | -3·0 (-3·9, -1·5) |  |
| MUAC*, cm | 13·2 (12·8, 14·0) | 12·1 (11·8, 12·2) | 10·6 (10·0, 11·3) | 11·6 (10·7, 13·1) |  | 13·9 (13·2, 14·5) | 12·2 (12·0, 12·4) | 11·0 (10·2, 11·4) | 11·8 (11·1, 13·5) |  |
| MUACZ* | -0·92 (-1·4, -0·33) | -2·2 (-2·5, -1·9) | -3·6 (-4·4, -2·9) | -2·7 (-3·7, -1·5) |  | -0·36 (-1·0, 0·20) | -2·1 (-2·4, -1·9) | -3·4 (-4·5, -2·8) | -3·0 (-3·5, -1·1) |  |
| WLZ* | -0·5 (-1·2, 0·2) | -2·0 (-2·4, -1·7) | -3·5 (-4·2, -2·8) | -1·7 (-2·8, -0·8) |  | -0·7 (-1·2, 0·2) | -2·4 (-2·7, -2·1) | -3·8 (-4·4, -3·2) | -1·9 (-3·2, -0·1) |  |
| **Underlying chronic conditions** |  |  |  |  |  |  |  |  |  |  |
| Stunted |  |  |  |  |  |  |  |  |  |  |
| *none* | 80 (66%) | 45 (55%) | 16 (21%) | 21 (20%) |  | 81 (77%) | 21 (68%) | 19 (40%) | 3 (18%) |  |
| *moderate* | 22 (18%) | 22 (27%) | 16 (21%) | 29 (28%) |  | 16 (15%) | 7 (23%) | 9 (19%) | 9 (53%) |  |
| *severe* | 20 (16%) | 15 (18%) | 45 (58%) | 53 (51%) |  | 8 (7·6%) | 3 (9·7%) | 19 (40%) | 5 (29%) |  |
| Small birth size | 17 (14%) | 17 (21%) | 16 (22%) | 11 (11%) |  | 15 (14%) | 5 (16%) | 7 (15%) | 2 (12%) |  |
| HIV status |  |  |  |  |  |  |  |  |  |  |
| *unexposed, uninfected* | 106 (87%) | 65 (79%) | 57 (74%) | 83 (81%) |  | 101 (96%) | 30 (97%) | 40 (85%) | 14 (82%) |  |
| *exposed, uninfected* | 11 (9·0%) | 12 (15%) | 12 (16%) | 15 (15%) |  | 4 (3·8%) | 0 (0%) | 2 (4·3%) | 1 (5·9%) |  |
| *infected* | 5 (4·1%) | 5 (6·1%) | 8 (10%) | 5 (4·9%) |  | 0 (0%) | 1 (3·2%) | 5 (11%) | 2 (12%) |  |
| Chronic conditions^ϛ^ | 5 (4·1%) | 5 (6·1%) | 4 (5·2%) | 3 (2·9%) |  | 11 (10%) | 7 (23%) | 9 (19%) | 2 (12%) |  |
| Prior hospitalisation | 35 (29%) | 21 (26%) | 22 (29%) | 29 (28%) |  | 18 (17%) | 11 (35%) | 15 (32%) | 3 (18%) |  |
| **Age-inappropriate nutrition** |  |  |  |  |  |  |  |  |  |  |
| Recommended adequate diet^ℸ^ | 68 (56%) | 38 (47%) | 21 (28%) | 6 (5·9%) |  | 54 (51%) | 18 (58%) | 19 (40%) | 1 (6·2%) |  |
| Reported recent weight loss | 10 (8·2%) | 42 (51%) | 56 (73%) | 70 (68%) |  | 6 (5·8%) | 12 (39%) | 33 (70%) | 9 (53%) |  |
| Reported poor feeding | 6 (4·9%) | 11 (13%) | 32 (42%) | 41 (40%) |  | 4 (3·8%) | 3 (9·7%) | 18 (38%) | 4 (24%) |  |
| Reported current breastfeeding | 91 (75%) | 50 (61%) | 32 (42%) | 8 (7·8%) |  | 93 (89%) | 29 (94%) | 31 (66%) | 3 (18%) |  |

**Supplemental Table 9.** Continued

**Supplemental Table 9.** Continued

|  | **Kampala** | | | |  | **Kilifi** | | | |
| --- | --- | --- | --- | --- | --- | --- | --- | --- | --- |
|  | NW | MW | SW | NO |  | NW | MW | SW | NO |
|  | n=122 | n=82 | n=77 | n=103 |  | n=105 | n=31 | n=47 | n=17 |
| **Caregiver characteristics** |  |  |  |  |  |  |  |  |  |
| Primary caregiver not biological mother | 3 (2·5%) | 10 (12%) | 14 (18%) | 14 (14%) |  | 6 (5·7%) | 0 (0%) | 2 (4·3%) | 0 (0%) |
| Caregiver education |  |  |  |  |  |  |  |  |  |
| *secondary/Tertiary* | 68 (56%) | 51 (62%) | 36 (49%) | 48 (48%) |  | 23 (22%) | 7 (23%) | 10 (21%) | 1 (5·9%) |
| *primary* | 51 (42%) | 27 (33%) | 34 (46%) | 50 (50%) |  | 61 (58%) | 17 (57%) | 27 (57%) | 10 (59%) |
| *none* | 3 (2·5%) | 4 (4·9%) | 4 (5·4%) | 3 (3·0%) |  | 21 (20%) | 6 (20%) | 10 (21%) | 6 (35%) |
| Mental health risk, moderate to severe | 31 (26%) | 18 (22%) | 26 (34%) | 37 (36%) |  | 8 (7·6%) | 7 (23%) | 9 (19%) | 10 (59%) |
| Employment |  |  |  |  |  |  |  |  |  |
| *employed* | 24 (20%) | 23 (28%) | 15 (20%) | 28 (27%) |  | 10 (9·7%) | 4 (13%) | 15 (33%) | 3 (18%) |
| *self-employed* | 36 (30%) | 26 (32%) | 15 (20%) | 20 (20%) |  | 39 (38%) | 11 (35%) | 8 (17%) | 5 (29%) |
| *no income* | 62 (51%) | 32 (40%) | 44 (59%) | 54 (53%) |  | 54 (52%) | 16 (52%) | 23 (50%) | 9 (53%) |
| **Household-level exposures** |  |  |  |  |  |  |  |  |  |
| Population density, 1000 people per km^2^ | 9·0 (5·7, 10·8) | 8·0 (4·6, 10·8) | 6·8 (5·3, 9·4) | 5·7 (2·8, 9·3) |  | 0·4 (0·1, 1·0) | 0·4 (0·2, 1·1) | 0·6 (0·3, 3·0) | 0·3 (0·1, 1·7) |
| Asset index |  |  |  |  |  |  |  |  |  |
| *least poor* | 21 (17%) | 11 (13%) | 13 (17%) | 8 (7·8%) |  | 11 (10%) | 3 (9·7%) | 2 (4·3%) | 0 (0%) |
| *fourth* | 49 (40%) | 30 (37%) | 21 (27%) | 23 (22%) |  | 10 (9·5%) | 3 (9·7%) | 7 (15%) | 2 (12%) |
| *middle* | 40 (33%) | 27 (33%) | 31 (40%) | 36 (35%) |  | 10 (9·5%) | 5 (16%) | 8 (17%) | 3 (18%) |
| *second* | 8 (6·6%) | 14 (17%) | 10 (13%) | 31 (30%) |  | 29 (28%) | 2 (6·5%) | 4 (8·5%) | 1 (5·9%) |
| *poorest* | 4 (3·3%) | 0 (0%) | 2 (2·6%) | 5 (4·9%) |  | 45 (43%) | 18 (58%) | 26 (55%) | 11 (65%) |
| Household food insecurity |  |  |  |  |  |  |  |  |  |
| *low* | 50 (41%) | 29 (35%) | 37 (48%) | 36 (35%) |  | 60 (57%) | 15 (48%) | 30 (64%) | 8 (47%) |
| *medium* | 58 (48%) | 40 (49%) | 28 (36%) | 46 (45%) |  | 32 (30%) | 10 (32%) | 8 (17%) | 3 (18%) |
| *high* | 14 (11%) | 13 (16%) | 12 (16%) | 21 (20%) |  | 13 (12%) | 6 (19%) | 9 (19%) | 6 (35%) |
| Toilet, not improved | 17 (14%) | 14 (17%) | 13 (17%) | 19 (18%) |  | 28 (27%) | 10 (32%) | 14 (30%) | 8 (47%) |
| Water source, not improved | 6 (4·9%) | 5 (6·1%) | 5 (6·5%) | 6 (5·8%) |  | 9 (8·6%) | 4 (13%) | 6 (13%) | 3 (18%) |
| **Access to health care** |  |  |  |  |  |  |  |  |  |
| Distance to hospital, km | 5·4 (2·7, 8·4) | 6·1 (3·1, 9·4) | 6·8 (4·5, 9·9) | 8·9 (5·1, 12·5) |  | 21·0 (9·2, 34·0) | 20·4 (11·8, 30·8) | 27·8 (17·6, 35·9) | 34·5 (27·6, 40·1) |
| Distance to nearest health facility, km | 0·9 (0·5, 1·4) | 1·0 (0·6, 1·5) | 0·8 (0·5, 1·5) | 1·1 (0·8, 1·7) |  | 2·2 (1·4, 3·9) | 1·2 (0·9, 2·4) | 2·1 (1·5, 2·6) | 2·2 (1·6, 2·8) |
| Means of travel to hospital |  |  |  |  |  |  |  |  |  |
| *bus, car, ambulance, train* | 64 (52%) | 42 (51%) | 50 (65%) | 75 (73%) |  | 67 (64%) | 21 (68%) | 39 (85%) | 14 (82%) |
| *walking, motorbike, tuktuk, rickshaw* | 58 (48%) | 40 (49%) | 27 (35%) | 28 (27%) |  | 38 (36%) | 10 (32%) | 7 (15%) | 3 (18%) |
| Travel cost to study hospital |  |  |  |  |  |  |  |  |  |
| *<1$* | 59 (49%) | 42 (51%) | 44 (58%) | 48 (47%) |  | 44 (44%) | 11 (38%) | 19 (43%) | 4 (24%) |
| ≥*1 to <5$* | 59 (49%) | 38 (46%) | 31 (41%) | 52 (51%) |  | 45 (45%) | 15 (52%) | 22 (50%) | 11 (65%) |
| ≥ *5$* | 3 (2·5%) | 2 (2·4%) | 1 (1·3%) | 2 (2·0%) |  | 11 (11%) | 3 (10%) | 3 (6·8%) | 2 (12%) |
| Travel time to study hospital |  |  |  |  |  |  |  |  |  |
| *less than 1hr* | 66 (54%) | 42 (51%) | 32 (42%) | 30 (29%) |  | 51 (49%) | 12 (39%) | 13 (28%) | 3 (19%) |
| *between 1hr and 2hr* | 44 (36%) | 33 (40%) | 31 (40%) | 47 (46%) |  | 43 (41%) | 14 (45%) | 28 (60%) | 6 (38%) |
| *2hr or more* | 12 (9·8%) | 7 (8·5%) | 14 (18%) | 26 (25%) |  | 10 (9·6%) | 5 (16%) | 6 (13%) | 7 (44%) |

Results presented as Frequency (%) or Median (IQR). ^Ψ^, cough or difficulty breathing with oxygen saturation <90%, central cyanosis, or grunting; very severe chest indrawing or inability to breastfeed or drink; or lethargy, reduced level of consciousness, or convulsions. ^¥^, anaemia: none, haemoglobin >110 g/L; mild 100–110 g/L; moderate/severe, <100 g/L. ^λ^, blood glucose <3 mmol/L or >10 mmol/L. ^Ø^, reported premature or low birthweight (<2·5 kg). ^ϛ^, includes thalassemia, cerebral palsy, sickle cell disease, congenital cardiac diseases and known tuberculosis. ^ℸ^, recommended adequate diet: for age < 6 months, exclusive breastfeeding; for age 6-9 months, consuming ≥ 2 food groups and breastmilk; for age 10-23 months, consuming ≥ 4 food groups and breastmilk. *, indicates metrics that are influenced by fluid retention; MUAC is considered less sensitive to oedema than other weight-based metrics. Groups: NW, no wasting; MW, moderate wasting; SW, severe wasting; NO, nutritional oedema; CP, community participants. Growth metrics: LAZ, length-for-age z-score; WAZ, weight-for-age z-score; MUAC, mid-upper arm circumference; MUACZ, mid-upper arm circumference z-score; WLZ, weight-for-length z-score.

### **Supplemental Table 10.** Participant characteristics at admission by nutritional group for Migori and Nairobi.

|  | **Migori** | | | |  | **Nairobi** | | | |
| --- | --- | --- | --- | --- | --- | --- | --- | --- | --- |
|  | NW | MW | SW | NO |  | NW | MW | SW | NO |
|  | n=67 | n=29 | n=36 | n=35 |  | n=77 | n=45 | n=70 | n=10 |
| **Demographics** |  |  |  |  |  |  |  |  |  |
| Age |  |  |  |  |  |  |  |  |  |
| ≥*12 mo.* | 24 (36%) | 5 (17%) | 17 (47%) | 29 (83%) |  | 30 (39%) | 14 (31%) | 33 (47%) | 6 (60%) |
| ≥*6 & <12 mo.* | 18 (27%) | 14 (48%) | 12 (33%) | 4 (11%) |  | 28 (36%) | 14 (31%) | 29 (41%) | 3 (30%) |
| *<6 mo.* | 25 (37%) | 10 (34%) | 7 (19%) | 2 (5·7%) |  | 19 (25%) | 17 (38%) | 8 (11%) | 1 (10%) |
| Sex, male | 40 (60%) | 15 (52%) | 20 (56%) | 21 (60%) |  | 49 (64%) | 19 (42%) | 37 (53%) | 4 (40%) |
| **Clinical presentation at admission** |  |  |  |  |  |  |  |  |  |
| Sepsis | 2 (3·0%) | 0 (0%) | 5 (14%) | 0 (0%) |  | 4 (5·2%) | 1 (2·2%) | 4 (5·7%) | 1 (10%) |
| Severe pneumonia ^Ψ^ | 11 (16%) | 1 (3·4%) | 3 (8·3%) | 1 (2·9%) |  | 45 (58%) | 22 (49%) | 27 (39%) | 4 (40%) |
| Impaired consciousness | 0 (0%) | 0 (0%) | 0 (0%) | 0 (0%) |  | 4 (5·2%) | 2 (4·4%) | 5 (7·1%) | 0 (0%) |
| Diarrhoea | 33 (49%) | 14 (48%) | 22 (61%) | 14 (40%) |  | 27 (35%) | 18 (40%) | 35 (50%) | 4 (40%) |
| Malaria, positive | 19 (28%) | 14 (48%) | 5 (15%) | 9 (26%) |  | 7 (9·2%) | 4 (8·9%) | 1 (1·4%) | 0 (0%) |
| Anemia^¥^ |  |  |  |  |  |  |  |  |  |
| *none* | 7 (11%) | 2 (8·3%) | 1 (2·8%) | 2 (5·7%) |  | 9 (13%) | 3 (6·8%) | 14 (21%) | 0 (0%) |
| *mild* | 8 (12%) | 3 (12%) | 4 (11%) | 9 (26%) |  | 20 (29%) | 6 (14%) | 17 (25%) | 2 (20%) |
| *moderate/Severe* | 49 (77%) | 19 (79%) | 31 (86%) | 24 (69%) |  | 41 (59%) | 35 (80%) | 37 (54%) | 8 (80%) |
| Blood glucose, abnormal ^λ^ | 7 (12%) | 6 (23%) | 6 (18%) | 3 (10%) |  | 4 (5·2%) | 3 (6·7%) | 5 (7·1%) | 2 (20%) |
| **Anthropometry at admission** |  |  |  |  |  |  |  |  |  |
| LAZ | -0·9 (-1·8, -0·1) | -1·5 (-2·4, -0·7) | -2·9 (-4·0, -1·1) | -2·9 (-4·3, -2·3) |  | -1·1 (-1·9, -0·1) | -1·5 (-2·3, -0·8) | -2·5 (-3·7, -1·9) | -2·1 (-2·5, -1·9) |
| WAZ* | -0·9 (-1·4, 0·0) | -1·7 (-3·0, -1·4) | -4·1 (-5·2, -2·9) | -3·5 (-4·3, -2·5) |  | -1·1 (-1·8, -0·1) | -2·5 (-2·8, -2·1) | -4·1 (-4·7, -3·3) | -2·8 (-3·5, -2·5) |
| MUAC*, cm | 13·4 (12·6, 14·2) | 12·2 (11·6, 12·4) | 10·6 (9·4, 11·6) | 11·7 (10·7, 12·1) |  | 13·6 (12·8, 14·7) | 11·9 (11·3, 12·3) | 11·2 (10·2, 11·6) | 12·2 (11·5, 13·1) |
| MUACZ* | -0·85 (-1·3, -0·24) | -2·0 (-2·5, -1·7) | -3·9 (-5·1, -3·1) | -2·9 (-3·9, -2·3) |  | -0·67 (-1·3, 0·20) | -2·3 (-2·6, -1·7) | -3·4 (-4·3, -2·8) | -1·9 (-2·7, -1·4) |
| WLZ* | -0·5 (-1·0, 0·4) | -2·2 (-2·5, -1·2) | -3·8 (-4·3, -3·3) | -3·0 (-3·5, -2·2) |  | -0·4 (-1·0, 0·2) | -2·4 (-2·7, -1·9) | -3·8 (-4·3, -3·1) | -2·4 (-2·9, -1·9) |
| **Underlying chronic conditions** |  |  |  |  |  |  |  |  |  |
| Stunted |  |  |  |  |  |  |  |  |  |
| *none* | 57 (85%) | 19 (66%) | 12 (33%) | 8 (23%) |  | 60 (78%) | 31 (69%) | 19 (27%) | 4 (40%) |
| *moderate* | 9 (13%) | 5 (17%) | 10 (28%) | 11 (31%) |  | 12 (16%) | 10 (22%) | 26 (37%) | 4 (40%) |
| *severe* | 1 (1·5%) | 5 (17%) | 14 (39%) | 16 (46%) |  | 5 (6·5%) | 4 (8·9%) | 25 (36%) | 2 (20%) |
| Small birth size | 0 (0%) | 1 (3·4%) | 5 (15%) | 2 (5·9%) |  | 9 (12%) | 9 (20%) | 16 (23%) | 1 (10%) |
| HIV status |  |  |  |  |  |  |  |  |  |
| *unexposed, uninfected* | 53 (79%) | 24 (83%) | 19 (53%) | 20 (57%) |  | 71 (92%) | 42 (93%) | 59 (84%) | 10 (100%) |
| *exposed, uninfected* | 10 (15%) | 4 (14%) | 9 (25%) | 12 (34%) |  | 5 (6·5%) | 2 (4·4%) | 9 (13%) | 0 (0%) |
| *infected* | 4 (6·0%) | 1 (3·4%) | 8 (22%) | 3 (8·6%) |  | 1 (1·3%) | 1 (2·2%) | 2 (2·9%) | 0 (0%) |
| Chronic conditions^ϛ^ | 3 (4·5%) | 2 (6·9%) | 0 (0%) | 0 (0%) |  | 4 (5·2%) | 3 (6·7%) | 3 (4·3%) | 0 (0%) |
| Prior hospitalisation | 5 (7·5%) | 3 (10%) | 7 (19%) | 3 (8·6%) |  | 15 (19%) | 5 (11%) | 19 (27%) | 2 (20%) |
| **Age-inappropriate nutrition** |  |  |  |  |  |  |  |  |  |
| Recommended adequate diet^ℸ^ | 44 (66%) | 15 (52%) | 9 (25%) | 3 (8·6%) |  | 52 (68%) | 33 (73%) | 49 (70%) | 7 (70%) |
| Reported recent weight loss | 2 (3·0%) | 4 (14%) | 11 (31%) | 16 (46%) |  | 1 (1·3%) | 4 (8·9%) | 5 (7·1%) | 0 (0%) |
| Reported poor feeding | 12 (18%) | 4 (14%) | 11 (31%) | 12 (34%) |  | 4 (5·2%) | 9 (20%) | 21 (30%) | 5 (50%) |
| Reported current breastfeeding | 58 (87%) | 23 (79%) | 15 (42%) | 3 (8·6%) |  | 66 (86%) | 42 (93%) | 55 (79%) | 9 (90%) |

**Supplemental Table 10.** Continued

**Supplemental Table 10.** Continued

|  | **Migori** | | | |  | **Nairobi** | | | |
| --- | --- | --- | --- | --- | --- | --- | --- | --- | --- |
|  | NW | MW | SW | NO |  | NW | MW | SW | NO |
|  | n=67 | n=29 | n=36 | n=35 |  | n=77 | n=45 | n=70 | n=10 |
| **Caregiver characteristics** |  |  |  |  |  |  |  |  |  |
| Primary caregiver not biological mother | 4 (6·1%) | 2 (6·9%) | 3 (8·6%) | 6 (17%) |  | 2 (2·6%) | 2 (4·4%) | 2 (2·9%) | 0 (0%) |
| Caregiver education |  |  |  |  |  |  |  |  |  |
| *secondary/Tertiary* | 21 (31%) | 4 (14%) | 10 (28%) | 7 (20%) |  | 42 (55%) | 27 (60%) | 31 (45%) | 6 (60%) |
| *primary* | 45 (67%) | 22 (76%) | 25 (69%) | 25 (71%) |  | 33 (43%) | 17 (38%) | 37 (54%) | 4 (40%) |
| *none* | 1 (1·5%) | 3 (10%) | 1 (2·8%) | 3 (8·6%) |  | 1 (1·3%) | 1 (2·2%) | 1 (1·4%) | 0 (0%) |
| Mental health risk, moderate to severe | 16 (24%) | 10 (34%) | 12 (33%) | 13 (37%) |  | 24 (31%) | 15 (33%) | 34 (49%) | 4 (40%) |
| Employment |  |  |  |  |  |  |  |  |  |
| *employed* | 12 (18%) | 3 (11%) | 6 (17%) | 5 (14%) |  | 16 (21%) | 14 (31%) | 14 (21%) | 5 (50%) |
| *self-employed* | 31 (46%) | 16 (57%) | 19 (53%) | 21 (60%) |  | 13 (17%) | 4 (8·9%) | 11 (16%) | 0 (0%) |
| *no income* | 24 (36%) | 9 (32%) | 11 (31%) | 9 (26%) |  | 48 (62%) | 27 (60%) | 43 (63%) | 5 (50%) |
| **Household-level exposures** |  |  |  |  |  |  |  |  |  |
| Population density, 1000 people per km^2^ | 0·3 (0·2, 0·8) | 0·2 (0·2, 0·3) | 0·3 (0·2, 0·4) | 0·2 (0·2, 0·3) |  | 21·6 (12·4, 34·5) | 14·4 (6·5, 31·9) | 22·5 (15·8, 43·3) | 30·4 (14·4, 50·0) |
| Asset index |  |  |  |  |  |  |  |  |  |
| *least poor* | 1 (1·5%) | 1 (3·4%) | 1 (2·8%) | 0 (0%) |  | 24 (31%) | 13 (29%) | 23 (33%) | 1 (10%) |
| *fourth* | 8 (12%) | 2 (6·9%) | 1 (2·8%) | 2 (5·7%) |  | 28 (36%) | 18 (40%) | 26 (37%) | 3 (30%) |
| *middle* | 8 (12%) | 1 (3·4%) | 4 (11%) | 3 (8·6%) |  | 20 (26%) | 12 (27%) | 17 (24%) | 4 (40%) |
| *second* | 20 (30%) | 6 (21%) | 8 (22%) | 4 (11%) |  | 4 (5·2%) | 2 (4·4%) | 4 (5·7%) | 2 (20%) |
| *poorest* | 30 (45%) | 19 (66%) | 22 (61%) | 26 (74%) |  | 1 (1·3%) | 0 (0%) | 0 (0%) | 0 (0%) |
| Household food insecurity |  |  |  |  |  |  |  |  |  |
| *low* | 34 (51%) | 14 (48%) | 15 (42%) | 9 (26%) |  | 32 (42%) | 20 (44%) | 23 (33%) | 1 (10%) |
| *medium* | 27 (40%) | 7 (24%) | 11 (31%) | 15 (43%) |  | 34 (44%) | 19 (42%) | 38 (54%) | 7 (70%) |
| *high* | 6 (9·0%) | 8 (28%) | 10 (28%) | 11 (31%) |  | 11 (14%) | 6 (13%) | 9 (13%) | 2 (20%) |
| Toilet, not improved | 36 (54%) | 22 (76%) | 26 (72%) | 29 (83%) |  | 14 (18%) | 3 (6·7%) | 9 (13%) | 3 (30%) |
| Water source, not improved | 21 (31%) | 8 (28%) | 12 (33%) | 18 (51%) |  | 39 (51%) | 22 (49%) | 32 (46%) | 7 (70%) |
| **Access to health care** |  |  |  |  |  |  |  |  |  |
| Distance to hospital, km | 18·8 (5·0, 28·8) | 20·6 (15·2, 32·0) | 20·7 (14·5, 23·5) | 21·8 (15·7, 29·6) |  | 7·0 (4·2, 9·5) | 7·9 (5·0, 11·0) | 8·2 (5·9, 11·7) | 5·3 (2·8, 7·0) |
| Distance to nearest health facility, km | 1·7 (0·8, 3·0) | 1·9 (1·3, 3·3) | 2·0 (1·3, 2·8) | 2·2 (1·5, 3·0) |  | 0·3 (0·2, 0·6) | 0·5 (0·2, 0·7) | 0·4 (0·2, 0·6) | 0·3 (0·3, 0·4) |
| Means of travel to hospital |  |  |  |  |  |  |  |  |  |
| *bus, car, ambulance, train* | 21 (32%) | 13 (46%) | 11 (31%) | 17 (49%) |  | 63 (84%) | 38 (86%) | 58 (88%) | 8 (80%) |
| *walking, motorbike, tuktuk, rickshaw* | 45 (68%) | 15 (54%) | 25 (69%) | 18 (51%) |  | 12 (16%) | 6 (14%) | 8 (12%) | 2 (20%) |
| Travel cost to study hospital |  |  |  |  |  |  |  |  |  |
| *<1$* | 22 (33%) | 6 (22%) | 12 (34%) | 3 (8·6%) |  | 40 (55%) | 21 (49%) | 32 (49%) | 7 (70%) |
| ≥*1 to <5$* | 42 (64%) | 21 (78%) | 22 (63%) | 32 (91%) |  | 29 (40%) | 17 (40%) | 28 (43%) | 3 (30%) |
| ≥ *5$* | 2 (3·0%) | 0 (0%) | 1 (2·9%) | 0 (0%) |  | 4 (5·5%) | 5 (12%) | 5 (7·7%) | 0 (0%) |
| Travel time to study hospital |  |  |  |  |  |  |  |  |  |
| *less than 1hr* | 39 (59%) | 13 (46%) | 20 (56%) | 14 (40%) |  | 16 (21%) | 18 (41%) | 22 (33%) | 4 (44%) |
| *between 1hr and 2hr* | 19 (29%) | 13 (46%) | 15 (42%) | 16 (46%) |  | 44 (59%) | 19 (43%) | 28 (42%) | 3 (33%) |
| *2hr or more* | 8 (12%) | 2 (7·1%) | 1 (2·8%) | 5 (14%) |  | 15 (20%) | 7 (16%) | 16 (24%) | 2 (22%) |

Results presented as Frequency (%) or Median (IQR). ^Ψ^, cough or difficulty breathing with oxygen saturation <90%, central cyanosis, or grunting; very severe chest indrawing or inability to breastfeed or drink; or lethargy, reduced level of consciousness, or convulsions. ^¥^, anaemia: none, haemoglobin >110 g/L; mild 100–110 g/L; moderate/severe, <100 g/L. ^λ^, blood glucose <3 mmol/L or >10 mmol/L. ^Ø^, reported premature or low birthweight (<2·5 kg). ^ϛ^, includes thalassemia, cerebral palsy, sickle cell disease, congenital cardiac diseases and known tuberculosis. ^ℸ^, recommended adequate diet: for age < 6 months, exclusive breastfeeding; for age 6-9 months, consuming ≥ 2 food groups and breastmilk; for age 10-23 months, consuming ≥ 4 food groups and breastmilk. *, indicates metrics that are influenced by fluid retention; MUAC is considered less sensitive to oedema than other weight-based metrics. Groups: NW, no wasting; MW, moderate wasting; SW, severe wasting; NO, nutritional oedema; CP, community participants. Growth metrics: LAZ, length-for-age z-score; WAZ, weight-for-age z-score; MUAC, mid-upper arm circumference; MUACZ, mid-upper arm circumference z-score; WLZ, weight-for-length z-score.

### **Supplemental Table 11.** Participant characteristics at admission by nutritional group for Karachi and Matlab.

|  | **Karachi** | | | |  | **Matlab** | | | |
| --- | --- | --- | --- | --- | --- | --- | --- | --- | --- |
|  | NW | MW | SW | NO |  | NW | MW | SW | NO |
|  | n=128 | n=64 | n=72 | n=18 |  | n=89 | n=100 | n=89 | n=9 |
| **Demographics** |  |  |  |  |  |  |  |  |  |
| Age |  |  |  |  |  |  |  |  |  |
| ≥*12 mo.* | 41 (32%) | 25 (39%) | 24 (33%) | 10 (56%) |  | 30 (34%) | 44 (44%) | 34 (38%) | 0 (0%) |
| ≥*6 & <12 mo.* | 38 (30%) | 24 (38%) | 30 (42%) | 7 (39%) |  | 36 (40%) | 43 (43%) | 52 (58%) | 6 (67%) |
| *<6 mo.* | 49 (38%) | 15 (23%) | 18 (25%) | 1 (5·6%) |  | 23 (26%) | 13 (13%) | 3 (3·4%) | 3 (33%) |
| Sex, male | 76 (59%) | 29 (45%) | 39 (54%) | 8 (44%) |  | 54 (61%) | 61 (61%) | 42 (47%) | 7 (78%) |
| **Clinical presentation at admission** |  |  |  |  |  |  |  |  |  |
| Sepsis | 11 (8·6%) | 7 (11%) | 20 (28%) | 1 (5·6%) |  | 0 (0%) | 1 (1·0%) | 1 (1·1%) | 0 (0%) |
| Severe pneumonia ^Ψ^ | 72 (56%) | 35 (55%) | 22 (31%) | 3 (17%) |  | 2 (2·2%) | 2 (2·0%) | 1 (1·1%) | 2 (22%) |
| Impaired consciousness | 1 (0·8%) | 2 (3·1%) | 4 (5·6%) | 0 (0%) |  | 0 (0%) | 0 (0%) | 0 (0%) | 0 (0%) |
| Diarrhoea | 32 (25%) | 20 (31%) | 45 (62%) | 11 (61%) |  | 68 (76%) | 90 (90%) | 85 (96%) | 8 (89%) |
| Malaria, positive | 7 (5·6%) | 3 (4·8%) | 5 (7·2%) | 1 (5·9%) |  | 0 (0%) | 0 (0%) | 0 (0%) | 0 (0%) |
| Anemia^¥^ |  |  |  |  |  |  |  |  |  |
| *none* | 25 (20%) | 10 (16%) | 13 (18%) | 1 (5·6%) |  | 21 (28%) | 27 (30%) | 35 (42%) | 2 (25%) |
| *mild* | 29 (23%) | 9 (14%) | 17 (24%) | 2 (11%) |  | 23 (30%) | 24 (26%) | 26 (31%) | 3 (38%) |
| *moderate/Severe* | 74 (58%) | 44 (70%) | 42 (58%) | 15 (83%) |  | 32 (42%) | 40 (44%) | 22 (27%) | 3 (38%) |
| Blood glucose, abnormal ^λ^ | 7 (5·6%) | 4 (6·3%) | 3 (4·4%) | 1 (5·9%) |  | 2 (2·2%) | 3 (3·0%) | 5 (5·6%) | 2 (22%) |
| **Anthropometry at admission** |  |  |  |  |  |  |  |  |  |
| LAZ | -1·6 (-2·5, -0·9) | -1·9 (-2·9, -1·1) | -3·0 (-3·7, -2·3) | -2·7 (-3·6, -1·5) |  | -1·2 (-1·9, -0·5) | -2·1 (-2·7, -1·3) | -2·4 (-3·2, -1·9) | -0·4 (-3·0, -0·1) |
| WAZ* | -1·5 (-2·3, -0·8) | -2·2 (-3·2, -1·7) | -4·1 (-4·8, -3·5) | -3·4 (-3·9, -2·1) |  | -1·5 (-2·1, -0·7) | -2·8 (-3·2, -2·2) | -3·5 (-3·8, -3·1) | -1·6 (-2·9, -0·4) |
| MUAC*, cm | 12·8 (12·4, 13·5) | 11·9 (11·6, 12·3) | 10·5 (9·7, 11·1) | 11·5 (9·8, 11·8) |  | 12·9 (12·6, 13·7) | 12·0 (11·7, 12·3) | 11·2 (10·9, 11·6) | 13·1 (12·6, 13·8) |
| MUACZ* | -1·3 (-1·7, -0·79) | -2·3 (-2·6, -1·9) | -3·9 (-4·5, -3·1) | -3·1 (-3·8, -2·1) |  | -1·1 (-1·7, -0·55) | -2·3 (-2·7, -2·1) | -3·1 (-3·3, -2·8) | -1·2 (-1·4, -0·52) |
| WLZ* | -0·9 (-1·3, -0·2) | -2·1 (-2·4, -1·3) | -3·4 (-4·0, -3·1) | -2·1 (-3·4, -1·2) |  | -0·9 (-1·4, -0·4) | -2·3 (-2·6, -1·9) | -3·1 (-3·4, -2·7) | -0·9 (-1·6, -0·2) |
| **Underlying chronic conditions** |  |  |  |  |  |  |  |  |  |
| Stunted |  |  |  |  |  |  |  |  |  |
| *none* | 75 (59%) | 37 (58%) | 15 (21%) | 6 (33%) |  | 67 (75%) | 45 (45%) | 30 (34%) | 6 (67%) |
| *moderate* | 37 (29%) | 12 (19%) | 18 (25%) | 4 (22%) |  | 11 (12%) | 36 (36%) | 35 (39%) | 1 (11%) |
| *severe* | 16 (12%) | 15 (23%) | 39 (54%) | 8 (44%) |  | 11 (12%) | 19 (19%) | 24 (27%) | 2 (22%) |
| Small birth size | 11 (8·7%) | 6 (9·4%) | 4 (5·6%) | 2 (11%) |  | 25 (28%) | 31 (31%) | 34 (38%) | 3 (33%) |
| HIV status |  |  |  |  |  |  |  |  |  |
| *unexposed, uninfected* | 127 (99%) | 64 (100%) | 72 (100%) | 18 (100%) |  | 89 (100%) | 100 (100%) | 89 (100%) | 9 (100%) |
| *exposed, uninfected* | 1 (0·8%) | 0 (0%) | 0 (0%) | 0 (0%) |  | 0 (0%) | 0 (0%) | 0 (0%) | 0 (0%) |
| *infected* | 0 (0%) | 0 (0%) | 0 (0%) | 0 (0%) |  | 0 (0%) | 0 (0%) | 0 (0%) | 0 (0%) |
| Chronic conditions^ϛ^ | 26 (20%) | 17 (27%) | 26 (36%) | 8 (44%) |  | 1 (1·1%) | 0 (0%) | 0 (0%) | 0 (0%) |
| Prior hospitalisation | 41 (32%) | 18 (28%) | 27 (38%) | 5 (28%) |  | 16 (18%) | 21 (21%) | 29 (33%) | 3 (33%) |
| **Age-inappropriate nutrition** |  |  |  |  |  |  |  |  |  |
| Recommended adequate diet^ℸ^ | 49 (38%) | 26 (41%) | 19 (26%) | 7 (39%) |  | 68 (76%) | 83 (83%) | 77 (87%) | 6 (67%) |
| Reported recent weight loss | 29 (23%) | 29 (45%) | 51 (71%) | 9 (50%) |  | 10 (11%) | 36 (36%) | 60 (67%) | 2 (22%) |
| Reported poor feeding | 1 (0·8%) | 1 (1·6%) | 2 (2·8%) | 0 (0%) |  | 0 (0%) | 1 (1·0%) | 6 (6·7%) | 1 (11%) |
| Reported current breastfeeding | 89 (70%) | 47 (73%) | 40 (56%) | 11 (61%) |  | 77 (87%) | 91 (91%) | 79 (89%) | 7 (78%) |

**Supplemental Table 11.** Continued

**Supplemental Table 11.** Continued

|  | **Karachi** | | | |  | **Matlab** | | | |
| --- | --- | --- | --- | --- | --- | --- | --- | --- | --- |
|  | NW | MW | SW | NO |  | NW | MW | SW | NO |
|  | n=128 | n=64 | n=72 | n=18 |  | n=89 | n=100 | n=89 | n=9 |
| **Caregiver characteristics** |  |  |  |  |  |  |  |  |  |
| Primary caregiver not biological mother | 4 (3·1%) | 1 (1·6%) | 1 (1·4%) | 1 (5·6%) |  | 1 (1·1%) | 0 (0%) | 1 (1·1%) | 0 (0%) |
| Caregiver education |  |  |  |  |  |  |  |  |  |
| *secondary/Tertiary* | 42 (33%) | 7 (11%) | 17 (24%) | 4 (22%) |  | 34 (38%) | 32 (32%) | 25 (28%) | 2 (22%) |
| *primary* | 28 (22%) | 18 (28%) | 20 (28%) | 4 (22%) |  | 42 (47%) | 45 (45%) | 53 (60%) | 7 (78%) |
| *none* | 57 (45%) | 39 (61%) | 35 (49%) | 10 (56%) |  | 13 (15%) | 23 (23%) | 11 (12%) | 0 (0%) |
| Mental health risk, moderate to severe | 7 (5·6%) | 5 (7·8%) | 7 (9·7%) | 0 (0%) |  | 0 (0%) | 0 (0%) | 0 (0%) | 0 (0%) |
| Employment |  |  |  |  |  |  |  |  |  |
| *employed* | 8 (6·3%) | 0 (0%) | 4 (5·6%) | 0 (0%) |  | 5 (5·6%) | 3 (3·0%) | 1 (1·1%) | 0 (0%) |
| *self-employed* | 3 (2·4%) | 1 (1·6%) | 2 (2·8%) | 2 (12%) |  | 1 (1·1%) | 1 (1·0%) | 1 (1·1%) | 0 (0%) |
| *no income* | 116 (91%) | 62 (98%) | 65 (92%) | 15 (88%) |  | 83 (93%) | 96 (96%) | 87 (98%) | 9 (100%) |
| **Household-level exposures** |  |  |  |  |  |  |  |  |  |
| Population density, 1000 people per km^2^ | 26·2 (10·9, 34·1) | 25·3 (14·9, 32·2) | 19·2 (11·0, 27·9) | 13·9 (9·4, 17·4) |  | 1·4 (1·1, 1·8) | 1·3 (1·0, 1·8) | 1·3 (1·1, 1·8) | 1·5 (1·1, 1·8) |
| Asset index |  |  |  |  |  |  |  |  |  |
| *least poor* | 83 (65%) | 37 (58%) | 43 (60%) | 10 (56%) |  | 12 (13%) | 9 (9·0%) | 13 (15%) | 0 (0%) |
| *fourth* | 35 (27%) | 22 (34%) | 22 (31%) | 5 (28%) |  | 16 (18%) | 18 (18%) | 16 (18%) | 1 (11%) |
| *middle* | 8 (6·2%) | 3 (4·7%) | 7 (9·7%) | 3 (17%) |  | 25 (28%) | 22 (22%) | 19 (21%) | 2 (22%) |
| *second* | 2 (1·6%) | 1 (1·6%) | 0 (0%) | 0 (0%) |  | 25 (28%) | 30 (30%) | 23 (26%) | 4 (44%) |
| *poorest* | 0 (0%) | 1 (1·6%) | 0 (0%) | 0 (0%) |  | 11 (12%) | 21 (21%) | 18 (20%) | 2 (22%) |
| Household food insecurity |  |  |  |  |  |  |  |  |  |
| *low* | 75 (59%) | 31 (48%) | 31 (43%) | 8 (44%) |  | 88 (99%) | 100 (100%) | 87 (98%) | 9 (100%) |
| *medium* | 41 (32%) | 22 (34%) | 28 (39%) | 6 (33%) |  | 1 (1·1%) | 0 (0%) | 2 (2·2%) | 0 (0%) |
| *high* | 12 (9·4%) | 11 (17%) | 13 (18%) | 4 (22%) |  | 0 (0%) | 0 (0%) | 0 (0%) | 0 (0%) |
| Toilet, not improved | 1 (0·8%) | 1 (1·6%) | 0 (0%) | 0 (0%) |  | 2 (2·2%) | 4 (4·0%) | 1 (1·1%) | 0 (0%) |
| Water source, not improved | 30 (24%) | 18 (28%) | 19 (27%) | 4 (22%) |  | 0 (0%) | 3 (3·0%) | 3 (3·4%) | 0 (0%) |
| **Access to health care** |  |  |  |  |  |  |  |  |  |
| Distance to hospital, km | 2·7 (1·7, 8·3) | 4·2 (1·7, 8·8) | 3·2 (2·2, 9·1) | 8·9 (4·8, 10·0) |  | 10·2 (5·5, 17·8) | 14·1 (7·6, 22·5) | 17·7 (12·5, 25·8) | 26·3 (11·7, 28·5) |
| Distance to nearest health facility, km | 0·4 (0·3, 0·8) | 0·5 (0·3, 0·8) | 0·5 (0·4, 0·9) | 0·8 (0·5, 1·1) |  | 5·0 (3·2, 6·9) | 5·3 (3·5, 7·5) | 4·8 (2·9, 6·3) | 5·2 (2·6, 5·7) |
| Means of travel to hospital |  |  |  |  |  |  |  |  |  |
| *bus, car, ambulance, train* | 17 (13%) | 11 (17%) | 9 (12%) | 2 (11%) |  | 1 (1·1%) | 4 (4·0%) | 2 (2·2%) | 0 (0%) |
| *walking, motorbike, tuktuk, rickshaw* | 110 (87%) | 52 (83%) | 63 (88%) | 16 (89%) |  | 88 (99%) | 96 (96%) | 87 (98%) | 9 (100%) |
| Travel cost to study hospital |  |  |  |  |  |  |  |  |  |
| *<1$* | 82 (65%) | 35 (56%) | 34 (47%) | 11 (65%) |  | 12 (13%) | 16 (16%) | 7 (7·9%) | 1 (11%) |
| ≥*1 to <5$* | 43 (34%) | 27 (43%) | 38 (53%) | 5 (29%) |  | 54 (61%) | 41 (41%) | 42 (47%) | 5 (56%) |
| ≥ *5$* | 2 (1·6%) | 1 (1·6%) | 0 (0%) | 1 (5·9%) |  | 23 (26%) | 43 (43%) | 40 (45%) | 3 (33%) |
| Travel time to study hospital |  |  |  |  |  |  |  |  |  |
| *less than 1hr* | 94 (74%) | 57 (89%) | 57 (80%) | 10 (56%) |  | 35 (42%) | 27 (28%) | 15 (17%) | 2 (29%) |
| *between 1hr and 2hr* | 32 (25%) | 7 (11%) | 13 (18%) | 8 (44%) |  | 37 (45%) | 33 (34%) | 34 (39%) | 2 (29%) |
| *2hr or more* | 1 (0·8%) | 0 (0%) | 1 (1·4%) | 0 (0%) |  | 11 (13%) | 36 (38%) | 38 (44%) | 3 (43%) |

Results presented as Frequency (%) or Median (IQR). ^Ψ^, cough or difficulty breathing with oxygen saturation <90%, central cyanosis, or grunting; very severe chest indrawing or inability to breastfeed or drink; or lethargy, reduced level of consciousness, or convulsions. ^¥^, anaemia: none, haemoglobin >110 g/L; mild 100–110 g/L; moderate/severe, <100 g/L. ^λ^, blood glucose <3 mmol/L or >10 mmol/L. ^Ø^, reported premature or low birthweight (<2·5 kg). ^ϛ^, includes thalassemia, cerebral palsy, sickle cell disease, congenital cardiac diseases and known tuberculosis. ^ℸ^, recommended adequate diet: for age < 6 months, exclusive breastfeeding; for age 6-9 months, consuming ≥ 2 food groups and breastmilk; for age 10-23 months, consuming ≥ 4 food groups and breastmilk. *, indicates metrics that are influenced by fluid retention; MUAC is considered less sensitive to oedema than other weight-based metrics. Groups: NW, no wasting; MW, moderate wasting; SW, severe wasting; NO, nutritional oedema; CP, community participants. Growth metrics: LAZ, length-for-age z-score; WAZ, weight-for-age z-score; MUAC, mid-upper arm circumference; MUACZ, mid-upper arm circumference z-score; WLZ, weight-for-length z-score.

### **Supplemental Table 12.** Participant characteristics at admission by nutritional group for Dhaka.

|  | **Dhaka** | | | |
| --- | --- | --- | --- | --- |
|  | NW | MW | SW | NO |
|  | n=116 | n=97 | n=138 | n=16 |
| **Demographics** |  |  |  |  |
| Age |  |  |  |  |
| ≥*12 mo.* | 34 (29%) | 27 (28%) | 47 (34%) | 2 (12%) |
| ≥*6 & <12 mo.* | 41 (35%) | 40 (41%) | 67 (49%) | 3 (19%) |
| *<6 mo.* | 41 (35%) | 30 (31%) | 24 (17%) | 11 (69%) |
| Sex, male | 72 (62%) | 58 (60%) | 88 (64%) | 10 (62%) |
| **Clinical presentation at admission** |  |  |  |  |
| Sepsis | 2 (1·7%) | 4 (4·1%) | 2 (1·4%) | 1 (6·2%) |
| Severe pneumonia ^Ψ^ | 22 (19%) | 15 (15%) | 13 (9·4%) | 1 (6·2%) |
| Impaired consciousness | 7 (6·0%) | 11 (11%) | 6 (4·3%) | 1 (6·2%) |
| Diarrhoea | 107 (92%) | 95 (98%) | 137 (99%) | 16 (100%) |
| Malaria, positive | 1 (0·9%) | 0 (0%) | 0 (0%) | 0 (0%) |
| Anemia^¥^ |  |  |  |  |
| *none* | 39 (34%) | 19 (20%) | 40 (29%) | 1 (6·2%) |
| *mild* | 31 (27%) | 26 (27%) | 46 (33%) | 5 (31%) |
| *moderate/Severe* | 46 (40%) | 52 (54%) | 52 (38%) | 10 (62%) |
| Blood glucose, abnormal ^λ^ | 9 (7·8%) | 9 (9·3%) | 10 (7·2%) | 2 (12%) |
| **Anthropometry at admission** |  |  |  |  |
| LAZ | -1·1 (-2·1, -0·3) | -2·3 (-3·3, -1·4) | -2·6 (-3·3, -2·0) | -3·3 (-4·0, -2·6) |
| WAZ* | -1·3 (-2·1, -0·6) | -3·2 (-3·7, -2·4) | -3·9 (-4·4, -3·5) | -4·2 (-4·6, -3·4) |
| MUAC*, cm | 13·5 (12·6, 14·3) | 11·9 (11·3, 12·4) | 11·1 (10·5, 11·6) | 10·9 (10·4, 12·0) |
| MUACZ* | -0·68 (-1·2, 0·02) | -2·2 (-2·7, -1·8) | -3·3 (-3·7, -2·7) | -2·9 (-3·6, -2·1) |
| WLZ* | -0·9 (-1·4, -0·2) | -2·5 (-2·8, -2·2) | -3·4 (-3·8, -3·2) | -2·1 (-3·2, -1·2) |
| **Underlying chronic conditions** |  |  |  |  |
| Stunted |  |  |  |  |
| *none* | 82 (71%) | 36 (37%) | 41 (30%) | 3 (19%) |
| *moderate* | 16 (14%) | 30 (31%) | 48 (35%) | 2 (12%) |
| *severe* | 18 (16%) | 31 (32%) | 49 (36%) | 11 (69%) |
| Small birth size | 22 (19%) | 29 (31%) | 33 (24%) | 3 (20%) |
| HIV status |  |  |  |  |
| *unexposed, uninfected* | 116 (100%) | 95 (98%) | 138 (100%) | 16 (100%) |
| *exposed, uninfected* | 0 (0%) | 2 (2·1%) | 0 (0%) | 0 (0%) |
| *infected* | 0 (0%) | 0 (0%) | 0 (0%) | 0 (0%) |
| Chronic conditions^ϛ^ | 1 (0·9%) | 1 (1·0%) | 3 (2·2%) | 0 (0%) |
| Prior hospitalisation | 40 (34%) | 33 (34%) | 40 (29%) | 5 (31%) |
| **Age-inappropriate nutrition** |  |  |  |  |
| Recommended adequate diet^ℸ^ | 57 (49%) | 53 (55%) | 63 (46%) | 1 (6·2%) |
| Reported recent weight loss | 2 (1·7%) | 7 (7·2%) | 37 (27%) | 4 (25%) |
| Reported poor feeding | 21 (18%) | 12 (12%) | 27 (20%) | 3 (19%) |
| Reported current breastfeeding | 83 (72%) | 78 (80%) | 101 (73%) | 7 (44%) |

**Supplemental Table 12.** Continued

**Supplemental Table 12.** Continued

|  | **Dhaka** | | | |
| --- | --- | --- | --- | --- |
|  | NW | MW | SW | NO |
|  | n=116 | n=97 | n=138 | n=16 |
| **Caregiver characteristics** |  |  |  |  |
| Primary caregiver not biological mother | 1 (0·9%) | 3 (3·1%) | 4 (2·9%) | 1 (6·2%) |
| Caregiver education |  |  |  |  |
| *secondary/Tertiary* | 30 (26%) | 21 (22%) | 12 (8·7%) | 0 (0%) |
| *primary* | 61 (53%) | 42 (43%) | 63 (46%) | 10 (62%) |
| *none* | 25 (22%) | 34 (35%) | 63 (46%) | 6 (38%) |
| Mental health risk, moderate to severe | 22 (19%) | 20 (21%) | 36 (26%) | 4 (25%) |
| Employment |  |  |  |  |
| *employed* | 20 (17%) | 21 (22%) | 25 (18%) | 2 (12%) |
| *self-employed* | 0 (0%) | 1 (1·0%) | 0 (0%) | 1 (6·2%) |
| *no income* | 96 (83%) | 75 (77%) | 113 (82%) | 13 (81%) |
| **Household-level exposures** |  |  |  |  |
| Population density, 1000 people per km^2^ | 36·9 (25·0, 77·1) | 40·6 (27·5, 70·6) | 47·4 (31·4, 70·6) | 32·0 (23·0, 62·1) |
| Asset index |  |  |  |  |
| *least poor* | 65 (56%) | 47 (48%) | 49 (36%) | 4 (25%) |
| *fourth* | 37 (32%) | 32 (33%) | 48 (35%) | 7 (44%) |
| *middle* | 11 (9·5%) | 16 (16%) | 35 (25%) | 1 (6·2%) |
| *second* | 3 (2·6%) | 2 (2·1%) | 6 (4·3%) | 4 (25%) |
| *poorest* | 0 (0%) | 0 (0%) | 0 (0%) | 0 (0%) |
| Household food insecurity |  |  |  |  |
| *low* | 91 (78%) | 75 (77%) | 94 (68%) | 13 (81%) |
| *medium* | 21 (18%) | 11 (11%) | 29 (21%) | 2 (12%) |
| *high* | 4 (3·4%) | 11 (11%) | 15 (11%) | 1 (6·2%) |
| Toilet, not improved | 2 (1·7%) | 7 (7·2%) | 13 (9·4%) | 1 (6·2%) |
| Water source, not improved | 0 (0%) | 1 (1·0%) | 3 (2·2%) | 2 (12%) |
| **Access to health care** |  |  |  |  |
| Distance to hospital, km | 6·4 (4·3, 9·7) | 7·0 (5·1, 10·7) | 6·2 (4·1, 9·5) | 7·1 (3·7, 11·6) |
| Distance to nearest health facility, km | 0·5 (0·2, 1·1) | 0·6 (0·3, 1·1) | 0·6 (0·3, 1·2) | 0·6 (0·3, 1·3) |
| Means of travel to hospital |  |  |  |  |
| *bus, car, ambulance, train* | 22 (19%) | 27 (28%) | 47 (34%) | 8 (50%) |
| *walking, motorbike, tuktuk, rickshaw* | 94 (81%) | 70 (72%) | 91 (66%) | 8 (50%) |
| Travel cost to study hospital |  |  |  |  |
| *<1$* | 20 (17%) | 24 (25%) | 43 (31%) | 5 (31%) |
| ≥*1 to <5$* | 91 (78%) | 68 (70%) | 90 (65%) | 10 (62%) |
| ≥ *5$* | 5 (4·3%) | 5 (5·2%) | 5 (3·6%) | 1 (6·2%) |
| Travel time to study hospital |  |  |  |  |
| *less than 1hr* | 35 (30%) | 26 (27%) | 41 (30%) | 5 (31%) |
| *between 1hr and 2hr* | 58 (50%) | 39 (40%) | 63 (46%) | 8 (50%) |
| *2hr or more* | 23 (20%) | 32 (33%) | 34 (25%) | 3 (19%) |

Results presented as Frequency (%) or Median (IQR). ^Ψ^, cough or difficulty breathing with oxygen saturation <90%, central cyanosis, or grunting; very severe chest indrawing or inability to breastfeed or drink; or lethargy, reduced level of consciousness, or convulsions. ^¥^, anaemia: none, haemoglobin >110 g/L; mild 100–110 g/L; moderate/severe, <100 g/L. ^λ^, blood glucose <3 mmol/L or >10 mmol/L. ^Ø^, reported premature or low birthweight (<2·5 kg). ^ϛ^, includes thalassemia, cerebral palsy, sickle cell disease, congenital cardiac diseases and known tuberculosis. ^ℸ^, recommended adequate diet: for age < 6 months, exclusive breastfeeding; for age 6-9 months, consuming ≥ 2 food groups and breastmilk; for age 10-23 months, consuming ≥ 4 food groups and breastmilk. *, indicates metrics that are influenced by fluid retention; MUAC is considered less sensitive to oedema than other weight-based metrics. Groups: NW, no wasting; MW, moderate wasting; SW, severe wasting; NO, nutritional oedema; CP, community participants. Growth metrics: LAZ, length-for-age z-score; WAZ, weight-for-age z-score; MUAC, mid-upper arm circumference; MUACZ, mid-upper arm circumference z-score; WLZ, weight-for-length z-score.


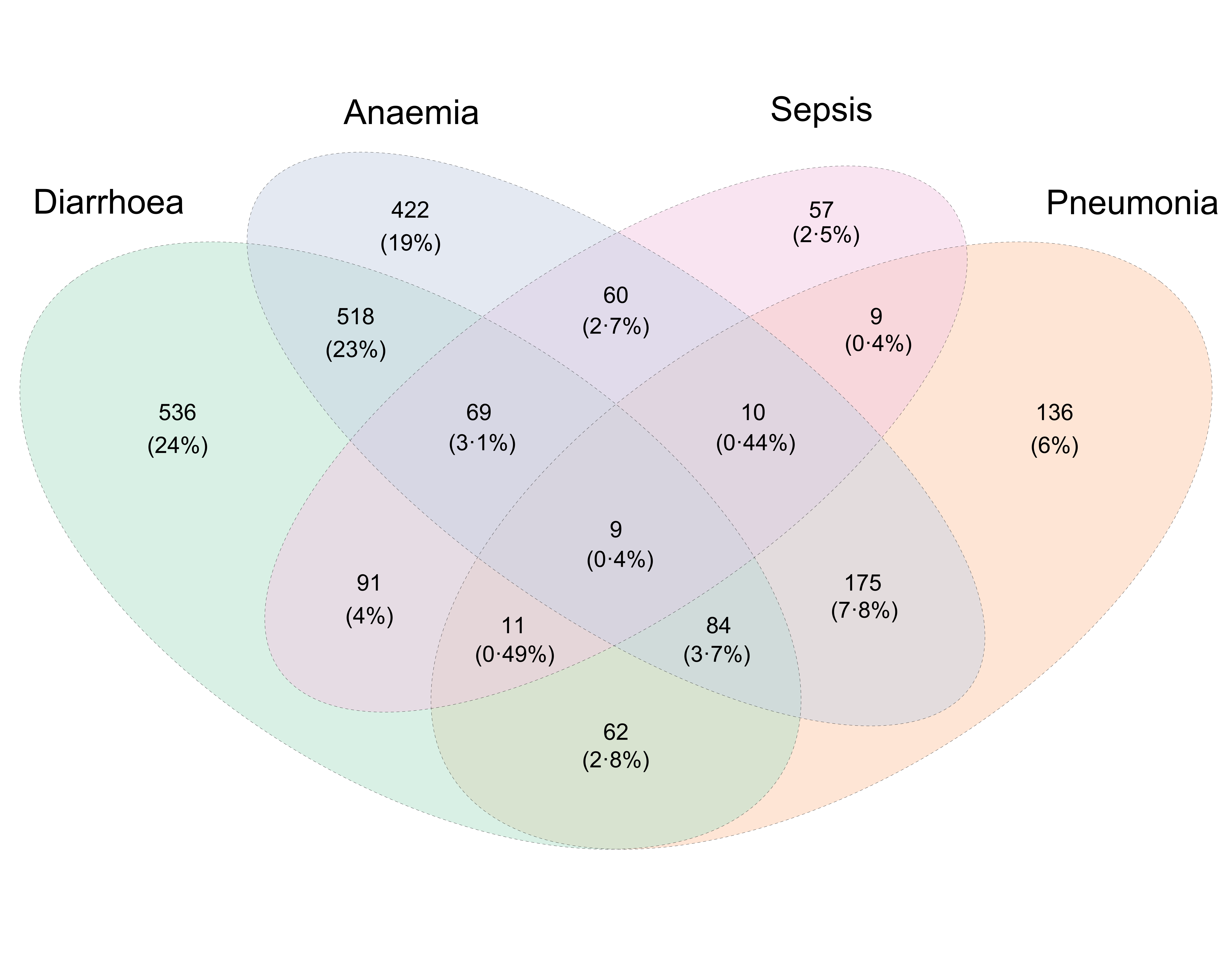


**Supplemental Figure 3**. Venn diagram detailing number and percent of children admitted to hospital with multiple conditions. The intersection of diarrhoea, moderate or severe anemia, sepsis or severe pneumonia is presented. Half of children (n=1321, 53%) had at least 2 conditions with diarrhoea and anaemia most often co-occurring (n=680). Only 205 children (8·3%) did not present with at least one of these conditions.

|  |  | Model 1 |  |  | Model 2 |  |  | Model 3 |  |
| --- | --- | --- | --- | --- | --- | --- | --- | --- | --- |
| *Predictors* | *Est.* | *95% CI* | *p* | *Est.* | *95% CI* | *p* | *Est.* | *95% CI* | *p* |
| Intercept [NW] | -1·05 | -1·23, -0·87 | **<0·0001** | -1·05 | -1·22, -0·89 | **<0·0001** | -1·05 | -1·21, -0·89 | **<0·0001** |
| Slope early discharge [NW] | 0·03 | 0·02, 0·04 | **<0·0001** | 0·03 | 0·02, 0·04 | **<0·0001** | 0·03 | 0·02, 0·04 | **<0·0001** |
| Difference in intercept early discharge [MW - NW] | -1·35 | -1·47, -1·23 | **<0·0001** | -1·35 | -1·47, -1·22 | **<0·0001** | -1·34 | -1·46, -1·23 | **<0·0001** |
| Difference in intercept early discharge [SW - NW] | -2·52 | -2·63, -2·40 | **<0·0001** | -2·52 | -2·63, -2·41 | **<0·0001** | -2·52 | -2·63, -2·40 | **<0·0001** |
| Difference in intercept early discharge [NO - NW] | -1·99 | -2·15, -1·82 | **<0·0001** | -1·99 | -2·16, -1·82 | **<0·0001** | -1·98 | -2·15, -1·82 | **<0·0001** |
| Difference in slope early discharge  [MW - NW] | 0·06 | 0·04, 0·07 | **<0·0001** | 0·06 | 0·04, 0·07 | **<0·0001** | 0·06 | 0·04, 0·07 | **<0·0001** |
| Difference in slope early discharge  [SW - NW] | 0·13 | 0·11, 0·14 | **<0·0001** | 0·13 | 0·11, 0·14 | **<0·0001** | 0·12 | 0·11, 0·13 | **<0·0001** |
| Difference in slope early discharge  [NO - NW] | 0·17 | 0·14, 0·19 | **<0·0001** | 0·17 | 0·14, 0·19 | **<0·0001** | 0·16 | 0·15, 0·18 | **<0·0001** |
| Age, months | 0·01 | 0·00, 0·02 | **0·020** | 0·01 | 0·00, 0·02 | **0·020** | 0·01 | 0·00, 0·02 | **0·023** |
| Sex, male | -0·25 | -0·33, -0·16 | **<0·0001** | -0·25 | -0·34, -0·16 | **<0·0001** | -0·25 | -0·33, -0·16 | **<0·0001** |
| **Random Effects** | | | | | | | | | |
| σ^2^ | 0·16 | | | 0·16 | | | 0·27 | | |
| τ_00_ | 1·22 record_id:site | | | 1·22 record_id | | | 1·09 record_id:site | | |
|  | 0·04 site | | | 0·03 site | | | 0·03 site | | |
| τ_11_ | 0·02 record_id:site·time_months | | | 0·02 record_id·time_months | | |  | | |
|  | 0·00 site·time_months | | |  | | |  | | |
| ρ_01_ | -0·29 record_id:site | | | -0·29 record_id | | |  | | |
|  | -0·70 site | | |  | | |  | | |
|  |  | | |  | | |  | | |
|  |  | | |  | | |  | | |
| ICC | 0·88 | | | 0·88 | | | 0·8 | | |
| N | 2461 _record_id_ | | | 9 _site_ | | | 2461 _record_id_ | | |
|  | 9 _site_ | | | 2461 _record_id_ | | | 9 _site_ | | |
| Observations | 9448 | | | 9448 | | | 9448 | | |
| Marginal R^2^ / Conditional R^2^ | 0·391 / 0·928 | | | 0·391 / 0·928 | | | 0·392 / 0·881 | | |
| AIC | 20523·547 | | | 20534·16 | | | 21464·634 | | |
| AICc | 20523·612 | | | 20534·211 | | | 21464·673 | | |
| BIC | 20585 |  |  | 20580 |  |  | 21494 |  |  |

### **Supplemental Table 13**. Model selection results for weight-for-age comparing different specifications of random structures and linear fit with- or without a knot positioned at day-45.

**Supplemental Table 13.** Continued

**Supplemental Table 13.** Continued

|  |  | Model 4 |  |  | Model 5 |  |  | Model 6 |  |
| --- | --- | --- | --- | --- | --- | --- | --- | --- | --- |
| *Predictors* | *Est.* | *95% CI* | *p* | *Est.* | *95% CI* | *p* | *Est.* | *95% CI* | *p* |
| Intercept [NW] | -1·09 | -1·28, -0·91 | **<0·0001** | -1·1 | -1·27, -0·93 | **<0·0001** | -1·11 | -1·28, -0·95 | **<0·0001** |
| Slope early discharge [NW] | 0·12 | 0·07, 0·16 | **<0·0001** | 0·12 | 0·09, 0·15 | **<0·0001** | 0·12 | 0·10, 0·15 | **<0·0001** |
| Difference in slope early vs late discharge [NW] | -0·11 | -0·16, -0·06 | **<0·0001** | -0·12 | -0·15, -0·09 | **<0·0001** | -0·12 | -0·16, -0·08 | **<0·0001** |
| Difference in intercept early discharge [MW - NW] | -1·36 | -1·48, -1·24 | **<0·0001** | -1·36 | -1·48, -1·24 | **<0·0001** | -1·36 | -1·49, -1·24 | **<0·0001** |
| Difference in intercept early discharge [SW - NW] | -2·59 | -2·71, -2·48 | **<0·0001** | -2·59 | -2·71, -2·48 | **<0·0001** | -2·6 | -2·71, -2·48 | **<0·0001** |
| Difference in intercept early discharge [NO - NW] | -2·1 | -2·27, -1·93 | **<0·0001** | -2·09 | -2·26, -1·93 | **<0·0001** | -2·1 | -2·27, -1·93 | **<0·0001** |
| Difference in slope early discharge  [MW - NW] | 0·08 | 0·03, 0·12 | **0·0010** | 0·08 | 0·04, 0·13 | **0·0010** | 0·08 | 0·04, 0·13 | **0·0010** |
| Difference in slope early discharge  [SW - NW] | 0·23 | 0·19, 0·28 | **<0·0001** | 0·23 | 0·19, 0·28 | **<0·0001** | 0·24 | 0·19, 0·29 | **<0·0001** |
| Difference in slope early discharge  [NO - NW] | 0·33 | 0·27, 0·40 | **<0·0001** | 0·32 | 0·26, 0·38 | **<0·0001** | 0·33 | 0·26, 0·39 | **<0·0001** |
| Difference in change of slope between early and late discharge [MW - NW] | -0·02 | -0·08, 0·03 | 0·39 | -0·03 | -0·08, 0·02 | 0·25 | -0·04 | -0·10, 0·02 | 0·24 |
| Difference in change of slope between early and late discharge [SW - NW] | -0·13 | -0·18, -0·08 | **<0·0001** | -0·14 | -0·19, -0·09 | **<0·0001** | -0·15 | -0·20, -0·09 | **<0·0001** |
| Difference in change of slope between early and late discharge [NO - NW] | -0·21 | -0·28, -0·14 | **<0·0001** | -0·2 | -0·27, -0·13 | **<0·0001** | -0·21 | -0·29, -0·13 | **<0·0001** |
| Age, months | 0·01 | -0·00, 0·02 | 0·052 | 0·01 | -0·00, 0·02 | 0·054 | 0·01 | 0·00, 0·02 | **0·023** |
| Sex, male | -0·25 | -0·33, -0·16 | **<0·0001** | -0·25 | -0·33, -0·16 | **<0·0001** | -0·25 | -0·34, -0·16 | **<0·0001** |
| **Random Effects** |  |  |  |  |  |  |  |  |  |
| σ^2^ | 0·1 | | | 0·1 | | | 0·26 | | |
| τ_00_ | 1·24 record_id:site | | | 1·24 record_id | | | 1·09 record_id:site | | |
|  | 0·04 site | | | 0·03 site | | | 0·03 site | | |
| τ_11_ | 0·12 record_id:site·time_months | | | 0·12 record_id·time_months | | |  | | |
|  | 0·12 record_id:site·I(pmax(time_months - 1·5, 0)) | | | 0·12 record_id·I(pmax(time_months - 1·5, 0)) | | |  | | |
|  | 0·00 site·time_months | | |  | | |  | | |
|  | 0·00 site·I(pmax(time_months - 1·5, 0)) | | |  | | |  | | |
| ρ_01_ | -0·19 | | | -0·19 record_id·time_months | | |  | | |
|  | 0·04 | | | 0·05 record_id·I(pmax(time_months - 1·5, 0)) | | |  | | |
|  | -0·58 | | |  | | |  | | |
|  | 0·4 | | |  | | |  | | |
| ICC | 0·93 | | | 0·93 | | | 0·81 | | |
| N | 2461 _record_id_ | | | 9 _site_ | | | 2461 _record_id_ | | |
|  | 9 _site_ | | | 2461 _record_id_ | | | 9 _site_ | | |
| Observations | 9448 | | | 9448 | | | 9448 | | |
| Marginal R^2^ / Conditional R^2^ | 0·393 / 0·957 | | | 0·393 / 0·957 | | | 0·395 / 0·886 | | |
| AIC | 19650·936 | | | 19680·466 | | | 21189·66 | | |
| AICc | 19651·096 | | | 19680·573 | | | 21189·725 | | |
| BIC | 19762 |  |  | 19753 |  |  | 21225 |  |  |

Table presents results for both fixed and random terms of either linear (Models 1-3) or mixed piecewise models (Models 4-6) fit using *lme4* R package. Different random structures were tested as defined in **Supplemental Table 5**. A knot point was either omitted or included (positioned at 45-days post-discharge) to define two discharge phases (i.e., early, before 45-days and, late, after 45-days). Time was coded in months (i.e., 1·5 months corresponds to 45-days; and 3 months to 90-days). Models were fit with maximum likelihood for comparison with anova using Satterthwaite's approximation of degrees of freedom as implemented in the *lmerTest* R package (Kuznetsova, A. et al., 2017). Models were evaluated using fit metrics AIC, AICc, and BIC. Groups: NW, no wasting; MW, moderate wasting; SW, severe wasting; NO, nutritional oedema. WAZ, weight-for-age z-score.

### **Supplemental Table 14.** Change in WAZ, LAZ, MUACZ and WLZ in the 6-months post-discharge by WHO nutritional group as estimated with multivariate piecewise mixed models built to assess differences between early and late post-discharge periods.

|  | **LAZ** | | | **WAZ** | | | **MUACZ** | | | **WHZ** | | |
| --- | --- | --- | --- | --- | --- | --- | --- | --- | --- | --- | --- | --- |
| *Predictors* | *Est.* | *95% CI* | *p* | *Est.* | *95% CI* | *p* | *Est.* | *95% CI* | *p* | *Est.* | *95% CI* | *p* |
| Intercept [NW] | -0·99 | -1·24, -0·73 | **<0·0001** | -1·1 | -1·27, -0·92 | **<0·0001** | -0·88 | -1·03, -0·73 | **<0·0001** | -0·28 | -0·42, -0·13 | **0·00015** |
| Slope early discharge [NW] | -0·13 | -0·15, -0·11 | **<0·0001** | 0·12 | 0·09, 0·15 | **<0·0001** | 0·17 | 0·13, 0·20 | **<0·0001** | 0·22 | 0·18, 0·26 | **<0·0001** |
| Difference in slope early vs late discharge [NW] | 0·11 | 0·09, 0·13 | **<0·0001** | -0·12 | -0·15, -0·09 | **<0·0001** | -0·13 | -0·17, -0·09 | **<0·0001** | -0·23 | -0·27, -0·18 | **<0·0001** |
| Difference in intercept early discharge [MW - NW] | -0·68 | -0·83, -0·52 | **<0·0001** | -1·36 | -1·48, -1·24 | **<0·0001** | -1·4 | -1·50, -1·29 | **<0·0001** | -1·42 | -1·53, -1·32 | **<0·0001** |
| Difference in intercept early discharge [SW - NW] | -1·57 | -1·71, -1·42 | **<0·0001** | -2·59 | -2·71, -2·48 | **<0·0001** | -2·55 | -2·65, -2·45 | **<0·0001** | -2·56 | -2·66, -2·46 | **<0·0001** |
| Difference in intercept early discharge [NO - NW] | -1·6 | -1·81, -1·40 | **<0·0001** | -2·1 | -2·26, -1·93 | **<0·0001** | -2·13 | -2·28, -1·99 | **<0·0001** | -1·8 | -1·94, -1·65 | **<0·0001** |
| Difference in slope early discharge [MW - NW] | -0·02 | -0·05, 0·01 | 0·26 | 0·08 | 0·04, 0·13 | **0·00059** | 0·19 | 0·14, 0·25 | **<0·0001** | 0·13 | 0·07, 0·20 | **0·0001** |
| Difference in slope early discharge [SW - NW] | 0·01 | -0·02, 0·04 | 0·51 | 0·23 | 0·19, 0·28 | **<0·0001** | 0·37 | 0·32, 0·43 | **<0·0001** | 0·37 | 0·30, 0·43 | **<0·0001** |
| Difference in slope early discharge [NO - NW] | 0·07 | 0·03, 0·11 | **0·0011** | 0·32 | 0·26, 0·38 | **<0·0001** | 0·5 | 0·42, 0·57 | **<0·0001** | 0·44 | 0·36, 0·53 | **<0·0001** |
| Difference in change of slope between early and late discharge [MW - NW] | 0·03 | -0·00, 0·07 | 0·050 | -0·03 | -0·08, 0·02 | 0·25 | -0·15 | -0·21, -0·08 | **<0·0001** | -0·07 | -0·15, 0·00 | 0·054 |
| Difference in change of slope between early and late discharge [SW - NW] | 0·03 | -0·01, 0·06 | 0·10 | -0·14 | -0·19, -0·09 | **<0·0001** | -0·28 | -0·34, -0·22 | **<0·0001** | -0·26 | -0·33, -0·18 | **<0·0001** |
| Difference in change of slope between early and late discharge [NO - NW] | -0·04 | -0·08, 0·01 | 0·13 | -0·2 | -0·27, -0·13 | **<0·0001** | -0·37 | -0·45, -0·29 | **<0·0001** | -0·27 | -0·37, -0·17 | **<0·0001** |
| Age, months | -0·01 | -0·02, -0·00 | **0·020** | 0·01 | -0·00, 0·02 | 0·057 | 0·02 | 0·01, 0·02 | **<0·0001** | -0·01 | -0·01, -0·00 | 0·026 |
| Sex, male | -0·31 | -0·42, -0·21 | **<0·0001** | -0·25 | -0·33, -0·16 | **<0·0001** | -0·19 | -0·26, -0·12 | **<0·0001** | -0·2 | -0·27, -0·13 | **<0·0001** |
| **Random Effects** | | | | | | | | | | | | |
| σ^2^ | 0·07 |  |  | 0·1 |  |  | 0·14 | | | 0·23 |  |  |
| τ_00_ | 2·03 _record_id_ | | | 1·24 _record_id_ | | | 0·87 _record_id_ | | | 0·77 _record_id_ | | |
|  | 0·10 _site_ |  |  | 0·03 _site_ | | | 0·03 _site_ | | | 0·02 _site_ | | |
| τ_11_ | 0·04 _record_id·time_months_ | | | 0·12 _record_id·time_months_ | | | 0·15 _record_id·time_months_ | | | 0·20 _record_id·time_months_ | | |
|  | 0·02 _record_id·I(pmax(time_months - 1·5, 0))_ | | | 0·12 _record_id·I(pmax(time_months - 1·5, 0))_ | | | 0·18 _record_id·I(pmax(time_months - 1·5, 0))_ | | | 0·22 _record_id·I(pmax(time_months - 1·5, 0))_ | | |
| ρ_01_ | -0·41 _record_id·time_months_ | | | -0·19 _record_id·time_months_ | | | -0·20 _record_id·time_months_ | | | -0·20 _record_id·time_months_ | | |
|  | 0·20 _record_id·I(pmax(time_months - 1·5, 0))_ | | | 0·05 _record_id·I(pmax(time_months - 1·5, 0))_ | | | 0·04 _record_id·I(pmax(time_months - 1·5, 0))_ | | | 0·09 _record_id·I(pmax(time_months - 1·5, 0))_ | | |
| ICC | 0·96 |  |  | 0·93 | | | 0·87 | | | 0·81 |  |  |
| N | 9 _site_ |  |  | 9 _site_ | | | 9 _site_ | | | 9 _site_ |  |  |
|  | 2461 _record_id_ | | | 2461 _record_id_ | | | 2461 _record_id_ | | | 2461 _record_id_ | | |
| Observations | 9425 | | | 9448 | | | 9364 | | | 9413 | | |
| Marginal R^2^ / Conditional R^2^ | 0·196 / 0·972 | | | 0·392 / 0·957 | | | 0·434 / 0·927 | | | 0·406 / 0·885 | | |

Table presents results for both fixed and random terms of mixed piecewise models fit using *lme4* R package. A knot point was positioned at 45-days post-discharge which defines two discharge phases (i.e., early, before 45-days and, late, after 45-days). Time was coded in months (i.e., 1·5 months corresponds to 45-days; and 3 months to 90-days). Random structure included random slopes per participant and random intercepts for site with participants nested within. Models were fit with maximum likelihood for comparison with anova using Satterthwaite's approximation of degrees of freedom as implemented in the *lmerTest* R package (Kuznetsova, A. et al., 2017). Models were evaluated using fit metrics AIC, AICc, and BIC. Restricted maximum likelihood was used to fit final models. Groups: NW, no wasting; MW, moderate wasting; SW, severe wasting; NO, nutritional oedema. LAZ, length-for-age z-score; WAZ, weight-for-age z-score; MUACZ, mid-upper arm circumference z-scores; WLZ, weight-for-length z-score.

**
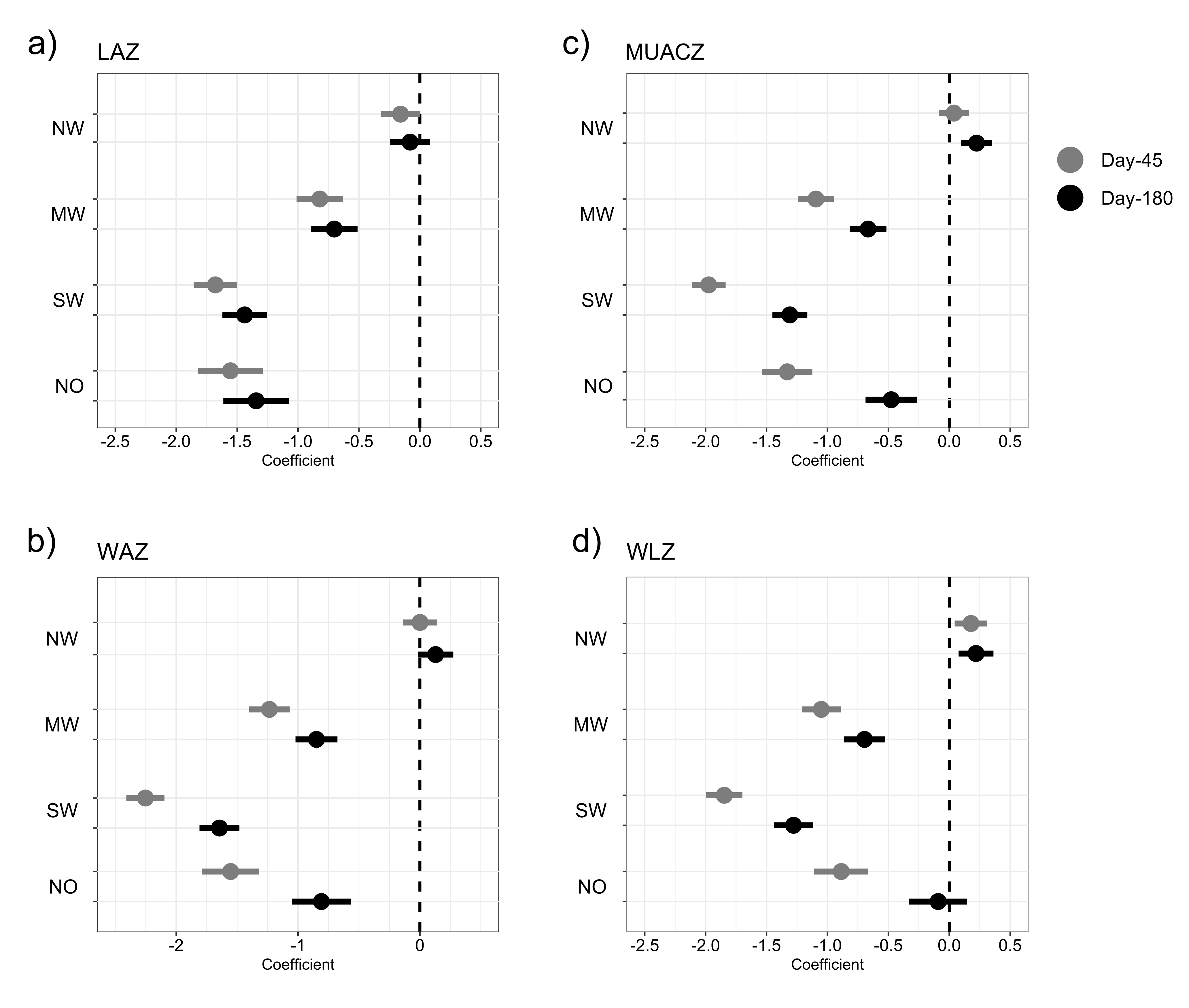
**

**Supplemental Figure 4.** Forest plots of coefficients derived from models comparing children from different nutritional groups to measures of community participants. a) LAZ, b) WAZ, c) MUACZ and d) WLZ at either 45-days (grey) or at 180-days (black). Models were adjusted for age and sex and included a random intercept to account for clustering of sites. Coefficients with whiskers that cross zero (vertical dashed line) do not differ from those of community participants. Coefficients and 95% CI for contrasts between groups were derived from models using emmeans R package. Groups: NW, no wasting; MW, moderate wasting; SW, severe wasting; NO, nutritional oedema; CP, community participants. LAZ, length-for-age z-score; WAZ, weight-for-age z-score, MUACZ, mid-upper arm circumference z-score; WLZ, weight-for-length z-score.

**
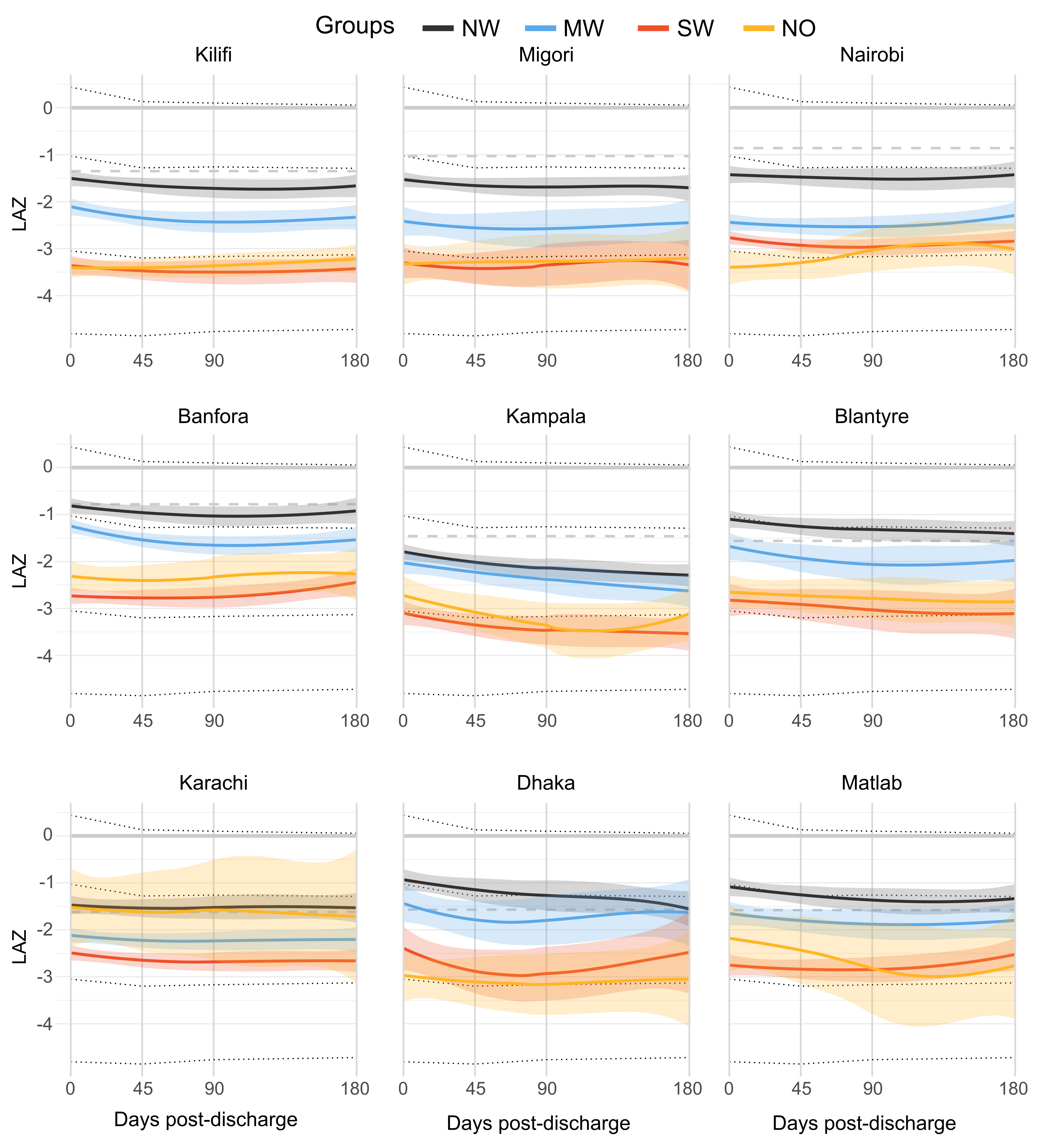
**

**Supplemental Figure 5.** Post-discharge length-for-age z-score split by site and nutritional group as classified at admission. Growth trajectories for each group were fitted with locally estimated scatterplot smoothing (LOESS) curves; line color indicates nutritional groups as per legend; colored shaded areas represent the standard error of the mean. Gray dotted line shows z-score of community participants. Black dotted lines represent the 50^th^ and 90^th^ intervals of the overall cohort. Groups: NW, no wasting; MW, moderate wasting; SW, severe wasting; NO, nutritional oedema. LAZ, length-for-age z-score.

**
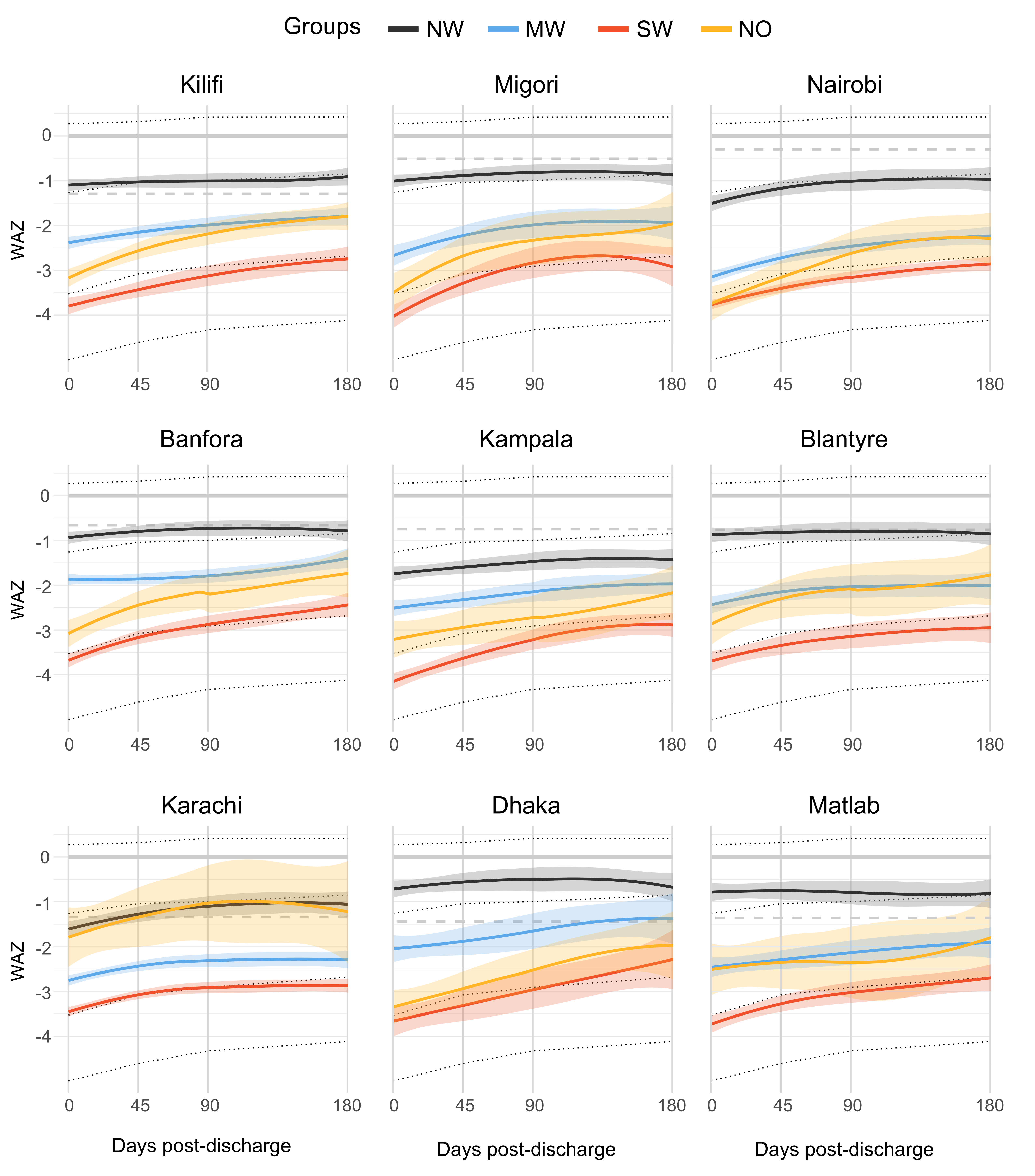
**

**Supplemental Figure 6.** Post-discharge weight-for-age z-score split by site and nutritional group as classified at admission. Growth trajectories for each group were fitted with locally estimated scatterplot smoothing (LOESS) curves; line color indicates nutritional groups as per legend; colored shaded areas represent the standard error of the mean. Gray dotted line shows z-score of community participants. Black dotted lines represent the 50^th^ and 90^th^ intervals of the overall cohort. Groups: NW, no wasting; MW, moderate wasting; SW, severe wasting; NO, nutritional oedema. WAZ, weight-for-age z-score.


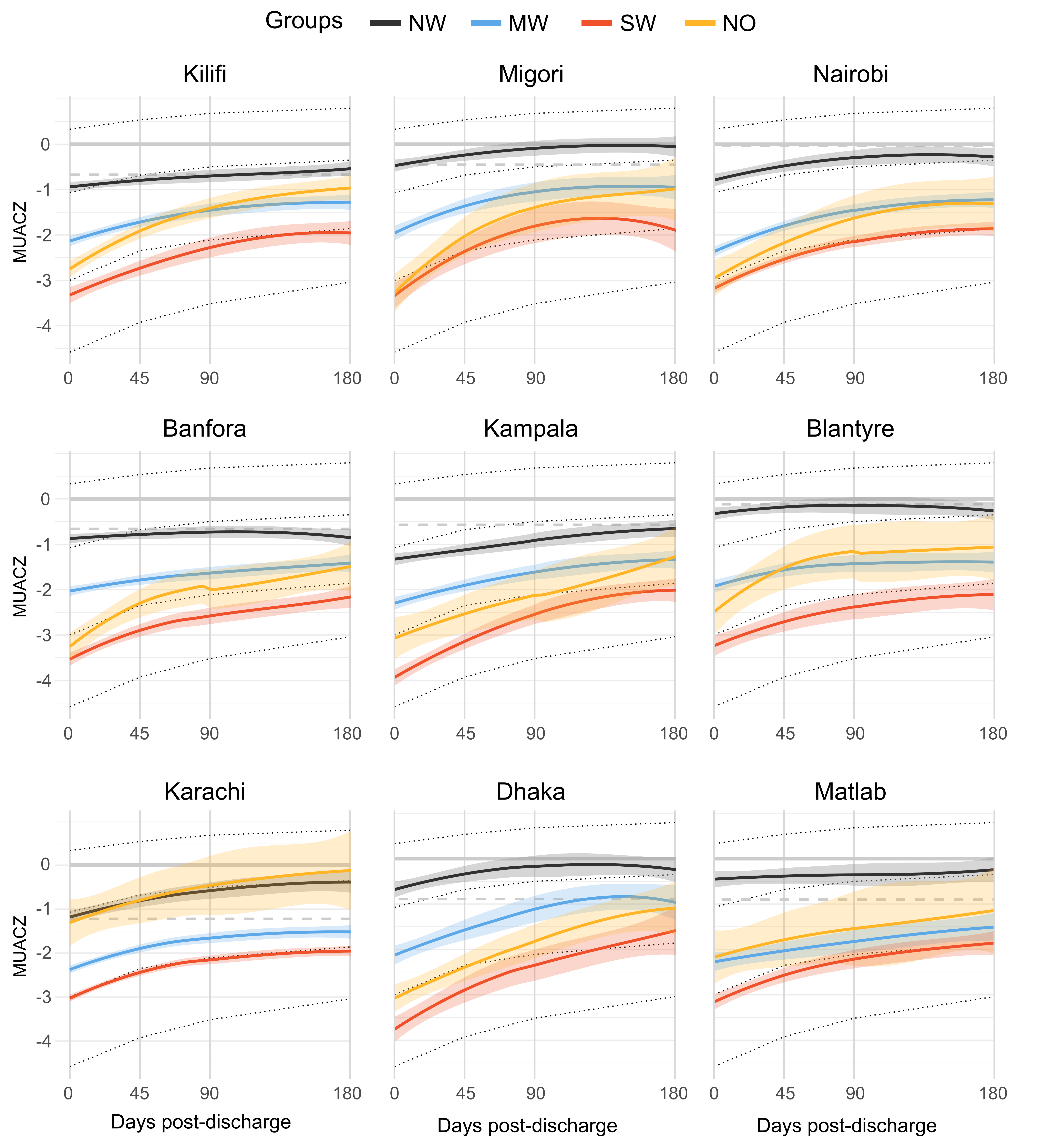


**Supplemental Figure 7.** Post-discharge mid-upper arm circumference z-score split by site and nutritional group as classified at admission. Growth trajectories for each group were fitted with locally estimated scatterplot smoothing (LOESS) curves; line color indicates nutritional groups as per legend; colored shaded areas represent the standard error of the mean. Gray dotted line shows z-score of community participants. Black dotted lines represent the 50^th^ and 90^th^ intervals of the overall cohort. Groups: NW, no wasting; MW, moderate wasting; SW, severe wasting; NO, nutritional oedema. MUACZ, mid upper arm circumference.

**
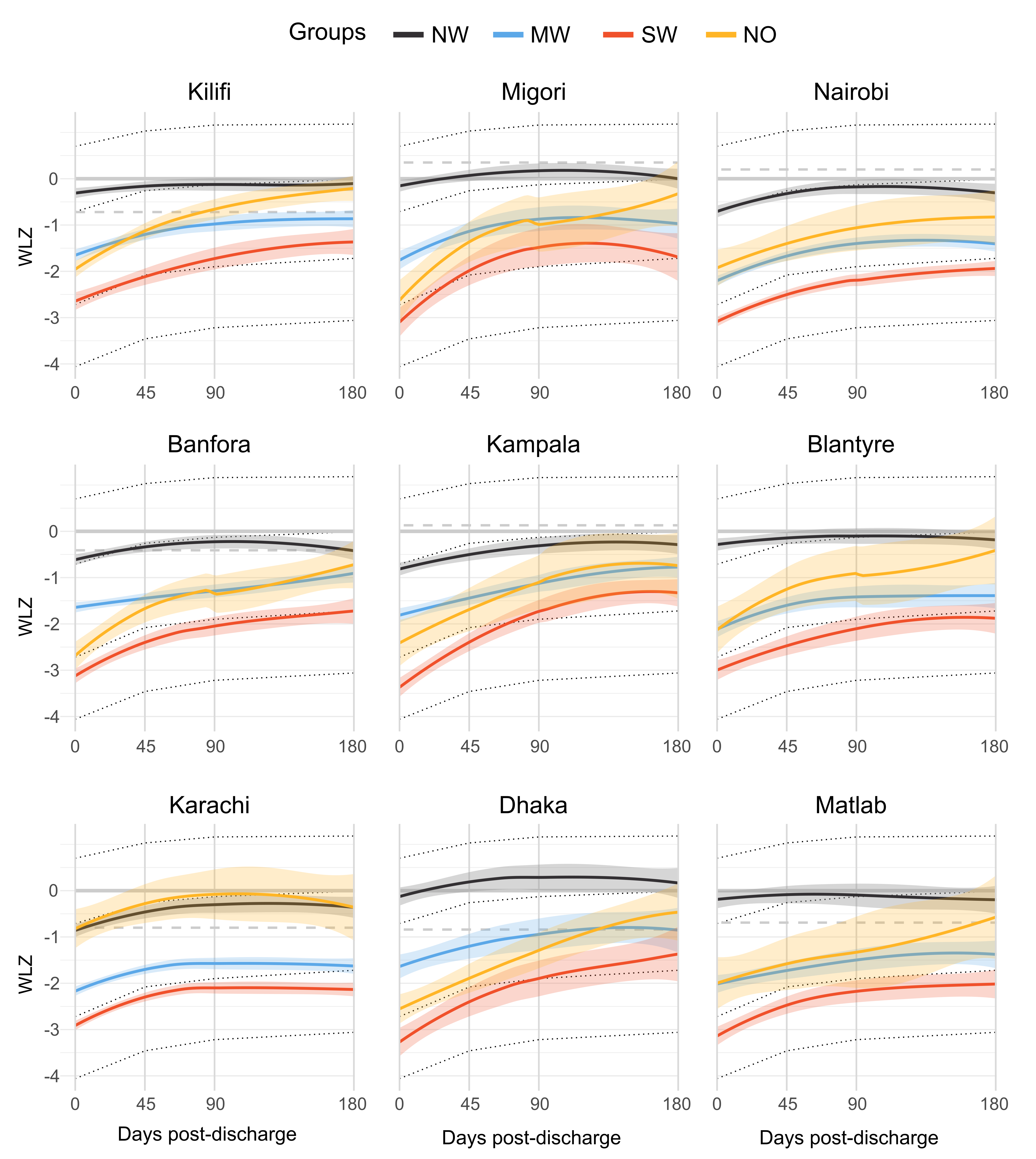
**

**Supplemental Figure 8**. Post-discharge weight-for-length z-score split by site and nutritional group as classified at admission. Growth trajectories for each group were fitted with locally estimated scatterplot smoothing (LOESS) curves; line color indicates nutritional groups as per legend; colored shaded areas represent the standard error of the mean. Gray dotted line shows z-score of community participants. Black dotted lines represent the 50th and 90th intervals of the overall cohort. Groups: NW, no wasting; MW, moderate wasting; SW, severe wasting; NO, nutritional oedema. WLZ, weight-for-length z-score.

### **Supplemental Table 15**. Median growth and differences between study visits by nutritional group and in community participants.

|  | |  | **NW**  n = 960 | **MW** n = 572 | **SW** n = 682 | **NO** n = 258 |
| --- | --- | --- | --- | --- | --- | --- |
| **Length-for-age, z-score** | |  |  |  |  |  |
| Discharge | |  | -1·3 (-2·2, -0·4) | -1·9 (-2·7, -1·1) | -2·8 (-3·7, -2·0) | -3·1 (-4·0, -2·1) |
| 45-days | |  | -1·5 (-2·3, -0·7) | -2·1 (-2·9, -1·4) | -2·9 (-3·9, -2·2) | -3·0 (-4·0, -2·2) |
| 90-days | |  | -1·5 (-2·4, -0·6) | -2·2 (-2·9, -1·4) | -2·9 (-3·9, -2·1) | -3·0 (-3·9, -2·2) |
| 180-days | |  | -1·5 (-2·5, -0·7) | -2·1 (-2·9, -1·4) | -2·9 (-3·8, -2·1) | -3·0 (-3·9, -2·0) |
| **Delta** | |  |  |  |  |  |
| △Discharge-to-D45 | |  | -0·22 (-0·47, 0·06) | -0·21 (-0·47, 0·04) | -0·16 (-0·44, 0·09) | -0·095 (-0·33, 0·15) |
| △D45-to-D90 | |  | -0·04 (-0·28, 0·24) | -0·020 (-0·27, 0·19) | -0·010 (-0·23, 0·27) | 0·04 (-0·19, 0·30) |
| △D90-to-D180 | |  | -0·07 (-0·37, 0·23) | 0·020 (-0·28, 0·28) | 0·060 (-0·25, 0·34) | 0·04 (-0·28, 0·36) |
| Total growth | |  | -0·28 (-0·71, 0·13) | -0·22 (-0·66, 0·14) | -0·060 (-0·52, 0·39) | 0·080 (-0·46, 0·48) |
|  | **Weight-for-age, z-score** | | | | | |
| Discharge | |  | -1·1 (-1·9, -0·4) | -2·5 (-3·2, -1·9) | -3·7 (-4·4, -3·1) | -3·3 (-4·2, -2·2) |
| 45-days | |  | -0·9 (-1·7, -0·3) | -2·2 (-2·9, -1·6) | -3·2 (-4·0, -2·5) | -2·4 (-3·3, -1·6) |
| 90-days | |  | -0·91 (-1·6, -0·18) | -2·1 (-2·7, -1·5) | -3·0 (-3·7, -2·4) | -2·1 (-3·1, -1·4) |
| 180-days | |  | -0·90 (-1·6, -0·21) | -1·9 (-2·5, -1·3) | -2·8 (-3·5, -2·0) | -1·8 (-2·8, -0·9) |
| **Delta** | |  |  |  |  |  |
| △Discharge-to-D45 | |  | 0·15 (-0·13, 0·45) | 0·30 (-0·043, 0·66) | 0·46 (0·05, 0·90) | 0·66 (0·11, 1·2) |
| △D45-to-D90 | |  | 0·045 (-0·19, 0·27) | 0·095 (-0·15, 0·41) | 0·18 (-0·10, 0·51) | 0·21 (-0·073, 0·62) |
| △D90-to-D180 | |  | -0·035 (-0·31, 0·26) | 0·11 (-0·16, 0·42) | 0·20 (-0·11, 0·59) | 0·37 (-0·010, 0·79) |
| Total growth | |  | 0·17 (-0·27, 0·59) | 0·47 (0·07, 0·94) | 0·81 (0·28, 1·6) | 1·3 (0·53, 2·0) |
|  | **MUAC, z-score** | | | | | |
| Discharge | |  | -0·85 (-1·4, -0·17) | -2·2 (-2·6, -1·8) | -3·2 (-3·9, -2·7) | -2·9 (-3·9, -1·8) |
| Day45 follow-up | |  | -0·57 (-1·2, 0·07) | -1·7 (-2·2, -1·2) | -2·5 (-3·1, -1·9) | -1·8 (-2·6, -1·0) |
| Day90 follow-up | |  | -0·43 (-1·0, 0·19) | -1·5 (-2·0, -1·0) | -2·2 (-2·9, -1·6) | -1·4 (-2·3, -0·069) |
| Day180 follow-up | |  | -0·38 (-1·0, 0·24) | -1·3 (-1·8, -0·8) | -1·9 (-2·5, -1·3) | -0·97 (-1·8, -0·32) |
| **Delta** | |  |  |  |  |  |
| △Discharge-to-D45 | |  | 0·19 (-0·10, 0·55) | 0·49 (0·08, 0·89) | 0·71 (0·25, 1·2) | 0·88 (0·37, 1·6) |
| △D45-to-D90 | |  | 0·13 (-0·16, 0·41) | 0·22 (-0·06, 0·54) | 0·22 (-0·06, 0·66) | 0·32 (-0·04, 0·70) |
| △D90-to-D180 | |  | 0·07 (-0·24, 0·43) | 0·16 (-0·17, 0·48) | 0·26 (-0·10, 0·79) | 0·38 (-0·07, 0·83) |
| Total growth | |  | 0·37 (-0·13, 0·89) | 0·80 (0·35, 1·3) | 1·2 (0·62, 2·0) | 1·8 (0·97, 2·6) |
|  | **Weight-for-length, z-score** | | | | | |
| Discharge | |  | -0·55 (-1·2, 0·22) | -2·0 (-2·4, -1·5) | -3·1 (-3·7, -2·5) | -2·2 (-3·1, -1·2) |
| Day45 follow-up | |  | -0·23 (-0·83, 0·50) | -1·4 (-2·0, -0·85) | -2·2 (-3·0, -1·5) | -1·1 (-2·0, -0·44) |
| Day90 follow-up | |  | -0·18 (-0·83, 0·57) | -1·3 (-1·9, -0·76) | -2·1 (-2·7, -1·2) | -0·85 (-1·6, -0·16) |
| Day180 follow-up | |  | -0·24 (-0·92, 0·45) | -1·2 (-1·7, -0·55) | -1·8 (-2·5, -0·94) | -0·32 (-1·3, 0·35) |
| **Delta** | |  |  |  |  |  |
| △Discharge-to-D45 | |  | 0·31 (-0·11, 0·74) | 0·53 (0·05, 1·0) | 0·75 (0·20, 1·3) | 0·92 (0·28, 1·8) |
| △D45-to-D90 | |  | 0·06 (-0·31, 0·39) | 0·14 (-0·25, 0·52) | 0·18 (-0·28, 0·66) | 0·22 (-0·18, 0·75) |
| △D90-to-D180 | |  | -0·035 (-0·48, 0·37) | 0·11 (-0·27, 0·47) | 0·17 (-0·24, 0·69) | 0·38 (-0·12, 1·1) |
| Total growth | |  | 0·32 (-0·32, 0·87) | 0·69 (0·14, 1·28) | 1·1 (0·40, 2·1) | 1·7 (0·84, 2·7) |

Data presented as median (IQR). Anthropometry of community Participants (n=1234) is as follows: LAZ, -1·3 (95% CI, -2·1, -0·54); WAZ, -0·98 (95% CI, -1·8, -0·14); MUACZ, -0·61 (95% CI, -1·3, 0·04); WLZ, -0·39 (95% CI, -1·1, -0·43). Groups: NW, no wasting; MW, moderate wasting; SW, severe wasting; NO, nutritional oedema. Growth metrics: LAZ, length-for-age z-score; WAZ, weight-for-age z-score; MUACZ, mid-upper arm circumference z-score; WLZ, weight-for-length z-score.

**Supplemental Table 16**. Summary of absolute length-for-age z-score (LAZ) at each timepoint and differences between time points split by nutritional groups and site.

|  |  |  | Length-for-age | | | | Delta of length-for-age | | | |
| --- | --- | --- | --- | --- | --- | --- | --- | --- | --- | --- |
|  | | n | Discharge | 45-days | 90-days | 180-days | △Discharge-to-D45 | △D45-to-D90 | △D90-to-D180 | Total growth |
| Banfora | NW | 118 | -0·8 (-1·6, 0·0) | -1·1 (-1·7, -0·3) | -1·1 (-1·8, -0·4) | -1·0 (-1·7, -0·2) | -0·2 (-0·4, 0·0) | -0·1 (-0·3, 0·2) | 0·0 (-0·3, 0·2) | -0·2 (-0·6, 0·1) |
|  | MW | 87 | -1·3 (-2·0, -0·6) | -1·6 (-2·4, -0·9) | -1·6 (-2·4, -1·0) | -1·5 (-2·3, -1·1) | -0·3 (-0·5, 0·0) | -0·1 (-0·3, 0·1) | 0·1 (-0·2, 0·3) | -0·3 (-0·6, 0·0) |
|  | SW | 113 | -2·7 (-3·5, -1·8) | -2·6 (-3·5, -1·9) | -2·6 (-3·5, -1·8) | -2·5 (-3·2, -1·7) | 0·0 (-0·3, 0·2) | 0·0 (-0·2, 0·3) | 0·1 (-0·1, 0·4) | 0·1 (-0·3, 0·5) |
|  | NO | 30 | -2·3 (-3·0, -1·6) | -2·5 (-2·9, -1·6) | -2·3 (-2·9, -1·7) | -2·2 (-3·0, -1·3) | -0·1 (-0·3, 0·1) | 0·0 (-0·2, 0·2) | 0·1 (-0·2, 0·5) | 0·2 (-0·3, 0·6) |
| Blantyre | NW | 138 | -1·5 (-2·2, -0·6) | -1·6 (-2·3, -0·8) | -1·6 (-2·3, -0·7) | -1·6 (-2·4, -0·8) | -0·2 (-0·4, 0·1) | 0·0 (-0·3, 0·2) | 0·0 (-0·4, 0·3) | -0·2 (-0·7, 0·2) |
|  | MW | 37 | -2·4 (-3·4, -1·6) | -2·6 (-3·3, -1·9) | -2·4 (-3·1, -2·0) | -2·4 (-3·2, -1·8) | 0·0 (-0·3, 0·2) | 0·1 (-0·3, 0·3) | 0·1 (-0·4, 0·5) | -0·1 (-0·7, 0·2) |
|  | SW | 40 | -3·4 (-4·5, -2·3) | -3·6 (-4·6, -2·5) | -3·3 (-4·4, -2·5) | -3·2 (-4·1, -2·4) | -0·1 (-0·3, 0·1) | 0·1 (-0·3, 0·4) | 0·0 (-0·3, 0·3) | 0·1 (-0·5, 0·7) |
|  | NO | 20 | -3·7 (-4·1, -2·8) | -3·4 (-4·4, -2·7) | -3·2 (-4·2, -2·7) | -3·2 (-4·2, -2·5) | -0·1 (-0·3, 0·1) | 0·1 (-0·1, 0·3) | -0·1 (-0·3, 0·2) | 0·0 (-0·4, 0·5) |
| Kampala | NW | 122 | -1·5 (-2·4, -0·5) | -1·8 (-2·5, -0·7) | -1·8 (-2·6, -0·9) | -1·6 (-2·6, -1·1) | -0·2 (-0·5, 0·1) | 0·0 (-0·3, 0·2) | 0·0 (-0·3, 0·3) | -0·3 (-0·7, 0·2) |
|  | MW | 82 | -2·0 (-2·8, -1·4) | -2·3 (-3·1, -1·7) | -2·4 (-3·1, -1·7) | -2·4 (-3·1, -1·6) | -0·3 (-0·5, 0·0) | 0·0 (-0·2, 0·2) | 0·0 (-0·3, 0·2) | -0·2 (-0·7, 0·1) |
|  | SW | 77 | -3·4 (-4·1, -2·2) | -3·5 (-4·3, -2·6) | -3·4 (-4·3, -2·8) | -3·5 (-4·2, -2·8) | -0·1 (-0·5, 0·2) | 0·0 (-0·2, 0·3) | 0·1 (-0·2, 0·4) | 0·0 (-0·6, 0·4) |
|  | NO | 103 | -3·3 (-4·2, -2·5) | -3·2 (-4·1, -2·7) | -3·5 (-4·0, -2·4) | -3·4 (-4·2, -2·4) | -0·1 (-0·3, 0·2) | 0·2 (-0·2, 0·3) | 0·0 (-0·3, 0·4) | 0·1 (-0·3, 0·6) |
| Kilifi | NW | 105 | -1·1 (-1·8, -0·2) | -1·4 (-2·1, -0·5) | -1·2 (-1·9, -0·4) | -1·3 (-2·0, -0·6) | -0·2 (-0·4, 0·1) | 0·0 (-0·2, 0·3) | -0·2 (-0·4, 0·1) | -0·3 (-0·6, 0·1) |
|  | MW | 31 | -1·6 (-2·4, -1·0) | -2·0 (-2·6, -1·2) | -2·4 (-2·8, -1·4) | -2·1 (-2·6, -1·2) | -0·4 (-0·6, -0·1) | -0·1 (-0·2, 0·0) | 0·2 (-0·2, 0·5) | -0·2 (-0·5, 0·1) |
|  | SW | 47 | -2·8 (-4·0, -1·5) | -2·9 (-4·3, -1·1) | -3·1 (-4·1, -1·9) | -3·0 (-4·2, -1·7) | -0·1 (-0·3, 0·1) | -0·1 (-0·2, 0·1) | 0·0 (-0·3, 0·3) | -0·2 (-0·5, 0·1) |
|  | NO | 17 | -2·5 (-3·5, -2·1) | -2·9 (-3·3, -2·2) | -2·8 (-3·5, -2·2) | -2·9 (-3·6, -2·0) | -0·1 (-0·4, 0·1) | 0·0 (-0·2, 0·2) | 0·0 (-0·2, 0·2) | -0·1 (-0·3, 0·5) |
| Migori | NW | 67 | -1·0 (-1·9, -0·1) | -1·2 (-2·1, -0·7) | -1·5 (-2·1, -0·4) | -1·5 (-2·5, -0·7) | -0·3 (-0·5, 0·0) | -0·1 (-0·5, 0·3) | -0·2 (-0·6, 0·2) | -0·5 (-1·1, 0·0) |
|  | MW | 29 | -1·6 (-2·3, -0·7) | -2·0 (-2·4, -1·3) | -1·7 (-2·1, -1·4) | -1·7 (-2·3, -1·1) | -0·4 (-0·5, -0·1) | 0·0 (-0·4, 0·2) | 0·0 (-0·5, 0·2) | -0·3 (-1·1, 0·0) |
|  | SW | 36 | -2·9 (-3·6, -1·5) | -3·2 (-4·6, -2·4) | -3·0 (-3·9, -2·0) | -2·6 (-3·5, -1·8) | -0·3 (-0·5, -0·1) | 0·1 (-0·2, 0·3) | 0·1 (-0·2, 0·4) | -0·2 (-0·6, 0·2) |
|  | NO | 35 | -3·0 (-4·4, -2·3) | -2·8 (-4·1, -2·4) | -3·0 (-4·3, -2·6) | -2·9 (-3·9, -2·2) | -0·2 (-0·3, 0·2) | -0·1 (-0·2, 0·2) | 0·1 (-0·2, 0·3) | -0·1 (-0·7, 0·5) |
| Nairobi | NW | 77 | -1·2 (-2·0, -0·2) | -1·3 (-2·1, -0·5) | -1·5 (-2·2, -0·6) | -1·5 (-2·4, -0·6) | -0·3 (-0·7, -0·1) | -0·1 (-0·3, 0·1) | -0·1 (-0·4, 0·2) | -0·3 (-0·8, 0·0) |
|  | MW | 45 | -1·5 (-2·3, -0·9) | -1·6 (-2·5, -1·2) | -1·5 (-2·7, -1·3) | -1·6 (-2·6, -1·3) | -0·2 (-0·5, -0·1) | -0·1 (-0·3, 0·2) | -0·1 (-0·3, 0·2) | -0·3 (-0·6, 0·2) |
|  | SW | 70 | -2·6 (-3·5, -1·9) | -2·8 (-3·6, -2·1) | -2·7 (-3·6, -2·0) | -2·4 (-3·3, -1·8) | -0·2 (-0·4, 0·1) | 0·1 (-0·2, 0·4) | 0·1 (-0·3, 0·5) | 0·1 (-0·4, 0·5) |
|  | NO | 10 | -2·3 (-2·6, -2·0) | -2·6 (-3·1, -2·2) | -2·6 (-3·3, -2·3) | -2·7 (-3·5, -1·8) | -0·2 (-0·3, 0·1) | -0·1 (-0·1, 0·0) | 0·1 (-0·2, 0·5) | -0·1 (-0·9, 0·2) |
| Karachi | NW | 128 | -1·7 (-2·6, -0·9) | -2·0 (-2·9, -1·2) | -2·1 (-2·9, -1·1) | -2·4 (-3·3, -1·4) | -0·3 (-0·6, 0·0) | -0·1 (-0·4, 0·2) | -0·2 (-0·4, 0·2) | -0·6 (-1·1, -0·1) |
|  | MW | 64 | -1·9 (-3·0, -1·1) | -2·1 (-3·2, -1·4) | -2·3 (-3·4, -1·5) | -2·5 (-3·5, -1·6) | -0·2 (-0·5, 0·1) | -0·2 (-0·4, 0·1) | -0·2 (-0·4, 0·0) | -0·5 (-1·1, 0·0) |
|  | SW | 72 | -3·1 (-3·8, -2·4) | -3·3 (-4·2, -2·5) | -3·6 (-4·2, -2·7) | -3·2 (-4·2, -2·7) | -0·2 (-0·6, 0·0) | -0·1 (-0·4, 0·2) | -0·1 (-0·4, 0·4) | -0·2 (-0·9, 0·3) |
|  | NO | 18 | -2·7 (-3·9, -1·7) | -2·8 (-3·8, -2·1) | -3·5 (-3·9, -2·2) | -2·9 (-3·9, -2·5) | -0·2 (-0·7, 0·0) | -0·2 (-0·3, -0·1) | 0·0 (-0·5, 0·4) | 0·0 (-1·2, 0·3) |
| Matlab | NW | 89 | -1·2 (-2·0, -0·6) | -1·5 (-2·2, -0·6) | -1·4 (-2·0, -0·6) | -1·4 (-2·0, -0·6) | -0·1 (-0·4, 0·1) | 0·0 (-0·2, 0·3) | 0·0 (-0·2, 0·2) | -0·1 (-0·4, 0·2) |
|  | MW | 100 | -2·2 (-2·7, -1·3) | -2·3 (-3·0, -1·5) | -2·2 (-3·0, -1·4) | -2·3 (-3·0, -1·4) | -0·2 (-0·4, 0·0) | 0·0 (-0·2, 0·1) | -0·1 (-0·3, 0·2) | -0·2 (-0·6, 0·1) |
|  | SW | 89 | -2·4 (-3·2, -1·8) | -2·6 (-3·4, -2·1) | -2·7 (-3·2, -2·0) | -2·6 (-3·4, -2·1) | -0·2 (-0·4, -0·1) | 0·0 (-0·2, 0·2) | 0·1 (-0·3, 0·3) | -0·2 (-0·5, 0·1) |
|  | NO | 9 | -0·5 (-3·1, -0·3) | -0·9 (-3·3, -0·5) | -1·3 (-2·5, -0·5) | -1·0 (-2·7, -0·9) | -0·2 (-0·5, 0·1) | 0·1 (-0·4, 0·5) | 0·0 (-0·3, 0·2) | -0·1 (-0·5, 0·0) |
| Dhaka | NW | 116 | -1·2 (-2·3, -0·3) | -1·5 (-2·4, -0·6) | -1·5 (-2·5, -0·5) | -1·5 (-2·5, -0·5) | -0·2 (-0·4, 0·2) | 0·0 (-0·2, 0·3) | 0·0 (-0·3, 0·2) | -0·2 (-0·6, 0·2) |
|  | MW | 97 | -2·3 (-3·3, -1·5) | -2·5 (-3·4, -1·6) | -2·5 (-3·1, -1·6) | -2·2 (-2·9, -1·6) | -0·2 (-0·4, 0·2) | 0·0 (-0·3, 0·3) | 0·1 (-0·1, 0·5) | 0·0 (-0·4, 0·5) |
|  | SW | 138 | -2·6 (-3·5, -2·1) | -2·9 (-3·6, -2·3) | -2·8 (-3·7, -2·2) | -2·8 (-3·6, -2·1) | -0·3 (-0·5, 0·0) | 0·0 (-0·2, 0·3) | 0·1 (-0·2, 0·3) | -0·2 (-0·5, 0·3) |
|  | NO | 16 | -3·4 (-4·1, -2·8) | -3·4 (-3·9, -3·0) | -2·8 (-3·8, -2·6) | -3·1 (-3·8, -2·2) | -0·1 (-0·6, 0·2) | 0·3 (0·0, 0·5) | 0·4 (-0·3, 0·6) | 0·4 (0·1, 1·1) |

Data presented as median (IQR). Groups: NW, no wasting; MW, moderate wasting; SW, severe wasting; NO, nutritional oedema. Growth metrics: LAZ, length-for-age z-score.

### **Supplemental Table 17.** Summary of absolute weight-for-age z-score (WAZ) at each timepoint and differences between time points split by nutritional groups and site.

|  |  |  | Weight-for-age | | | | Delta weight-for-age | | | |
| --- | --- | --- | --- | --- | --- | --- | --- | --- | --- | --- |
|  | | n | Discharge | 45-days | 90-days | 180-days | △Discharge-to-D45 | △D45-to-D90 | △D90-to-D180 | Total growth |
| Banfora | NW | 118 | -0·9 (-1·5, -0·4) | -0·8 (-1·3, -0·3) | -0·8 (-1·3, -0·3) | -0·8 (-1·3, -0·2) | 0·1 (-0·2, 0·4) | 0·0 (-0·2, 0·3) | -0·1 (-0·3, 0·2) | 0·1 (-0·3, 0·6) |
|  | MW | 87 | -1·9 (-2·4, -1·4) | -1·9 (-2·3, -1·4) | -1·8 (-2·4, -1·2) | -1·5 (-2·2, -1·0) | 0·0 (-0·3, 0·3) | 0·1 (-0·2, 0·4) | 0·1 (0·0, 0·5) | 0·3 (-0·1, 0·7) |
|  | SW | 113 | -3·6 (-4·5, -2·9) | -3·0 (-4·0, -2·2) | -2·8 (-3·6, -2·1) | -2·5 (-3·1, -1·7) | 0·5 (0·0, 1·0) | 0·1 (-0·1, 0·5) | 0·3 (0·0, 0·7) | 0·9 (0·4, 1·8) |
|  | NO | 30 | -3·2 (-3·6, -2·3) | -2·1 (-2·7, -1·6) | -2·0 (-2·9, -1·5) | -1·5 (-2·1, -1·0) | 0·8 (0·3, 1·5) | 0·1 (-0·2, 0·2) | 0·4 (0·1, 1·0) | 1·6 (0·6, 2·1) |
| Blantyre | NW | 138 | -1·0 (-1·7, -0·1) | -0·8 (-1·6, -0·1) | -0·7 (-1·5, -0·1) | -0·9 (-1·5, -0·2) | 0·1 (-0·2, 0·4) | 0·1 (-0·1, 0·3) | -0·1 (-0·3, 0·2) | 0·1 (-0·3, 0·5) |
|  | MW | 37 | -2·7 (-3·4, -2·3) | -2·0 (-2·8, -1·7) | -2·0 (-2·6, -1·5) | -1·9 (-2·4, -1·4) | 0·5 (0·1, 0·8) | 0·1 (-0·2, 0·3) | -0·1 (-0·3, 0·5) | 0·4 (0·2, 1·1) |
|  | SW | 40 | -4·0 (-4·6, -3·5) | -3·2 (-4·2, -2·7) | -2·6 (-3·4, -2·0) | -2·7 (-3·6, -2·0) | 0·6 (0·1, 1·2) | 0·5 (0·2, 0·9) | 0·1 (-0·2, 0·5) | 1·3 (0·3, 2·3) |
|  | NO | 20 | -3·9 (-4·5, -2·7) | -2·1 (-3·6, -1·8) | -2·2 (-3·1, -1·7) | -1·9 (-2·9, -1·2) | 1·1 (0·6, 1·6) | 0·0 (-0·2, 0·5) | 0·4 (0·0, 0·6) | 1·4 (1·1, 2·3) |
| Kampala | NW | 122 | -1·0 (-1·9, -0·4) | -0·8 (-1·7, -0·3) | -1·0 (-1·6, -0·3) | -0·9 (-1·4, -0·4) | 0·1 (-0·1, 0·4) | 0·0 (-0·3, 0·2) | 0·0 (-0·3, 0·3) | 0·1 (-0·2, 0·4) |
|  | MW | 82 | -2·3 (-2·8, -1·9) | -2·0 (-2·7, -1·3) | -1·9 (-2·4, -1·4) | -1·7 (-2·3, -1·1) | 0·3 (0·1, 0·6) | 0·1 (-0·1, 0·4) | 0·1 (-0·2, 0·3) | 0·5 (0·2, 0·9) |
|  | SW | 77 | -3·7 (-4·6, -3·2) | -3·4 (-4·1, -2·5) | -3·1 (-3·9, -2·2) | -2·6 (-3·6, -2·0) | 0·5 (0·0, 0·7) | 0·3 (0·0, 0·5) | 0·3 (-0·2, 0·7) | 1·0 (0·4, 1·6) |
|  | NO | 103 | -3·2 (-4·2, -2·3) | -2·5 (-3·2, -1·5) | -2·2 (-3·1, -1·3) | -1·8 (-2·9, -0·8) | 0·8 (0·3, 1·2) | 0·3 (-0·1, 0·6) | 0·3 (-0·2, 0·7) | 1·3 (0·5, 2·0) |
| Kilifi | NW | 105 | -0·9 (-1·7, -0·1) | -0·9 (-1·6, 0·2) | -0·9 (-1·5, 0·3) | -0·8 (-1·6, -0·1) | 0·1 (-0·2, 0·3) | 0·0 (-0·2, 0·1) | 0·0 (-0·3, 0·2) | 0·0 (-0·4, 0·5) |
|  | MW | 31 | -2·6 (-3·0, -2·1) | -2·1 (-2·9, -1·6) | -2·1 (-2·8, -1·5) | -2·0 (-2·5, -1·5) | 0·2 (0·0, 0·6) | 0·1 (-0·2, 0·3) | 0·0 (-0·2, 0·3) | 0·5 (0·2, 0·8) |
|  | SW | 47 | -3·5 (-4·5, -3·1) | -3·2 (-4·0, -2·5) | -3·0 (-3·7, -2·5) | -2·9 (-3·7, -2·0) | 0·1 (-0·2, 1·0) | 0·2 (-0·1, 0·4) | 0·2 (-0·3, 0·4) | 0·7 (0·0, 1·3) |
|  | NO | 17 | -3·1 (-4·2, -1·7) | -2·2 (-3·2, -1·3) | -2·3 (-3·1, -1·4) | -1·5 (-2·5, -0·9) | 0·9 (-0·1, 1·0) | 0·2 (-0·1, 0·4) | 0·6 (0·3, 0·8) | 1·3 (0·5, 1·9) |
| Migori | NW | 67 | -0·7 (-1·4, 0·1) | -0·7 (-1·2, 0·2) | -0·4 (-1·3, 0·4) | -0·5 (-1·3, 0·1) | 0·0 (-0·2, 0·4) | 0·1 (-0·2, 0·3) | 0·0 (-0·4, 0·2) | 0·1 (-0·3, 0·4) |
|  | MW | 29 | -2·1 (-2·8, -1·5) | -1·7 (-2·4, -1·4) | -1·8 (-2·1, -1·0) | -1·5 (-1·9, -0·8) | 0·1 (-0·2, 0·7) | 0·1 (-0·1, 0·5) | 0·1 (-0·1, 0·4) | 0·4 (-0·2, 0·9) |
|  | SW | 36 | -3·5 (-5·1, -2·6) | -3·3 (-4·1, -2·7) | -2·9 (-3·9, -2·3) | -2·3 (-3·3, -1·6) | 0·4 (-0·1, 1·1) | 0·3 (-0·1, 0·7) | 0·6 (0·3, 1·1) | 1·2 (0·5, 2·0) |
|  | NO | 35 | -3·3 (-4·3, -2·4) | -2·6 (-3·7, -2·0) | -2·4 (-3·3, -1·6) | -1·9 (-2·7, -0·6) | 0·5 (-0·2, 1·0) | 0·4 (0·0, 0·9) | 0·3 (0·1, 1·1) | 1·3 (0·8, 1·9) |
| Nairobi | NW | 77 | -0·9 (-1·6, -0·1) | -0·8 (-1·3, 0·0) | -0·8 (-1·6, 0·0) | -0·8 (-1·8, -0·2) | 0·0 (-0·2, 0·2) | -0·1 (-0·3, 0·1) | -0·1 (-0·4, 0·2) | -0·2 (-0·6, 0·3) |
|  | MW | 45 | -2·2 (-2·8, -1·9) | -2·3 (-2·8, -1·8) | -2·1 (-2·4, -1·5) | -1·9 (-2·4, -1·5) | 0·2 (-0·2, 0·4) | 0·2 (0·0, 0·5) | 0·1 (-0·1, 0·4) | 0·5 (0·0, 1·0) |
|  | SW | 70 | -3·7 (-4·5, -2·9) | -3·3 (-3·8, -2·4) | -2·9 (-4·1, -2·2) | -2·9 (-3·4, -1·8) | 0·5 (0·1, 0·9) | 0·2 (-0·2, 0·4) | 0·1 (-0·1, 0·5) | 0·7 (0·0, 1·5) |
|  | NO | 10 | -2·4 (-3·6, -1·5) | -2·2 (-2·7, -1·8) | -2·0 (-3·1, -1·7) | -1·7 (-2·5, -1·2) | 0·3 (0·0, 0·5) | 0·3 (0·1, 0·4) | 0·5 (0·1, 0·8) | 1·1 (0·6, 1·5) |
| Karachi | NW | 128 | -1·7 (-2·6, -0·9) | -1·5 (-2·3, -0·7) | -1·5 (-2·2, -0·5) | -1·4 (-2·3, -0·5) | 0·1 (-0·3, 0·5) | 0·1 (-0·2, 0·5) | 0·0 (-0·3, 0·4) | 0·3 (-0·3, 0·7) |
|  | MW | 64 | -2·3 (-3·2, -1·7) | -2·2 (-3·0, -1·5) | -2·1 (-2·7, -1·5) | -1·7 (-2·4, -1·3) | 0·3 (-0·2, 0·7) | 0·2 (-0·2, 0·5) | 0·2 (-0·1, 0·6) | 0·4 (-0·1, 1·1) |
|  | SW | 72 | -4·2 (-5·0, -3·4) | -3·6 (-4·4, -2·9) | -3·1 (-3·8, -2·5) | -2·8 (-3·5, -2·1) | 0·5 (0·0, 1·2) | 0·4 (0·1, 0·9) | 0·3 (-0·1, 0·8) | 1·3 (0·5, 2·2) |
|  | NO | 18 | -3·5 (-4·1, -2·9) | -2·9 (-3·7, -2·3) | -2·8 (-3·1, -2·0) | -2·1 (-2·9, -1·2) | 0·2 (-0·2, 0·7) | 0·1 (-0·1, 1·1) | 0·4 (-0·3, 0·8) | 0·9 (0·0, 2·0) |
| Matlab | NW | 89 | -1·6 (-2·2, -0·8) | -1·1 (-1·8, -0·4) | -1·0 (-1·5, -0·3) | -1·0 (-1·6, -0·4) | 0·3 (0·0, 0·6) | 0·1 (0·0, 0·3) | 0·0 (-0·3, 0·2) | 0·3 (0·0, 0·7) |
|  | MW | 100 | -2·8 (-3·2, -2·2) | -2·3 (-2·9, -1·7) | -2·3 (-2·9, -1·7) | -2·3 (-3·0, -1·6) | 0·4 (0·1, 0·7) | 0·0 (-0·3, 0·2) | 0·0 (-0·2, 0·2) | 0·3 (0·0, 0·7) |
|  | SW | 89 | -3·4 (-3·8, -3·2) | -3·0 (-3·4, -2·6) | -2·9 (-3·4, -2·5) | -2·9 (-3·4, -2·3) | 0·4 (0·2, 0·8) | 0·0 (-0·2, 0·2) | 0·0 (-0·2, 0·2) | 0·4 (0·2, 0·9) |
|  | NO | 9 | -1·6 (-3·0, -0·6) | -1·1 (-2·4, -0·6) | -0·8 (-1·8, -0·6) | -0·8 (-1·3, -0·4) | 0·3 (0·1, 0·5) | 0·3 (0·2, 0·7) | 0·0 (-0·2, 0·0) | 0·6 (0·3, 1·0) |
| Dhaka | NW | 116 | -1·5 (-2·2, -0·7) | -1·0 (-1·9, -0·3) | -1·0 (-1·7, -0·3) | -1·0 (-1·5, -0·3) | 0·3 (0·0, 0·6) | 0·0 (-0·2, 0·4) | 0·0 (-0·2, 0·3) | 0·3 (0·0, 0·8) |
|  | MW | 97 | -3·2 (-3·7, -2·5) | -2·6 (-3·3, -1·9) | -2·4 (-3·1, -1·8) | -2·3 (-2·9, -1·7) | 0·4 (0·1, 0·8) | 0·2 (-0·1, 0·5) | 0·1 (-0·1, 0·5) | 0·6 (0·2, 1·4) |
|  | SW | 138 | -3·7 (-4·3, -3·2) | -3·2 (-3·9, -2·7) | -3·2 (-3·8, -2·6) | -2·9 (-3·4, -2·4) | 0·4 (0·1, 0·7) | 0·2 (-0·1, 0·4) | 0·2 (-0·1, 0·5) | 0·8 (0·3, 1·4) |
|  | NO | 16 | -3·8 (-4·4, -3·4) | -3·2 (-4·3, -2·1) | -2·9 (-3·5, -1·9) | -2·4 (-2·7, -1·7) | 0·7 (-0·2, 1·4) | 0·4 (0·1, 0·7) | 0·4 (0·1, 0·7) | 1·7 (0·9, 2·0) |

Data presented as median (IQR). Groups: NW, no wasting; MW, moderate wasting; SW, severe wasting; NO, nutritional oedema. Growth metrics: WAZ, weight-for-age z-score.

### **Supplemental Table 18.** Summary of absolute mid upper arm circumference z-score (MUACZ) at each timepoint and differences between time points split by nutritional groups and site.

|  |  |  | MUAC, z-score | | | | Delta of MUAC z-score | | | |
| --- | --- | --- | --- | --- | --- | --- | --- | --- | --- | --- |
|  | | n | Discharge | 45-days | 90-days | 180-days | △Discharge-to-D45 | △D45-to-D90 | △D90-to-D180 | Total growth |
| Banfora | NW | 118 | -1·0 (-1·4, -0·5) | -0·8 (-1·2, -0·4) | -0·7 (-1·2, -0·3) | -0·8 (-1·3, -0·3) | 0·0 (-0·2, 0·4) | 0·1 (-0·2, 0·4) | -0·1 (-0·4, 0·3) | 0·1 (-0·3, 0·6) |
|  | MW | 87 | -1·9 (-2·4, -1·6) | -1·7 (-2·2, -1·3) | -1·6 (-2·2, -1·2) | -1·4 (-1·9, -1·0) | 0·2 (-0·1, 0·7) | 0·1 (-0·3, 0·4) | 0·1 (-0·2, 0·6) | 0·6 (0·2, 1·0) |
|  | SW | 113 | -3·4 (-4·3, -2·8) | -2·6 (-3·6, -2·0) | -2·4 (-3·2, -1·9) | -2·0 (-2·7, -1·4) | 0·7 (0·2, 1·4) | 0·1 (-0·2, 0·5) | 0·4 (0·0, 0·9) | 1·2 (0·5, 2·1) |
|  | NO | 30 | -3·5 (-3·9, -2·9) | -1·8 (-3·0, -1·4) | -1·7 (-2·9, -1·0) | -1·6 (-2·0, -0·8) | 1·4 (0·7, 1·8) | 0·2 (-0·3, 0·5) | 0·3 (-0·1, 0·9) | 1·7 (0·8, 2·6) |
| Blantyre | NW | 138 | -0·4 (-1·2, 0·2) | -0·2 (-0·9, 0·4) | 0·0 (-0·9, 0·5) | 0·0 (-0·6, 0·6) | 0·1 (-0·2, 0·6) | 0·2 (-0·2, 0·4) | 0·1 (-0·3, 0·5) | 0·4 (-0·2, 0·9) |
|  | MW | 37 | -1·9 (-2·3, -1·7) | -1·5 (-1·8, -0·7) | -1·1 (-1·5, -0·7) | -1·0 (-1·5, -0·6) | 0·5 (0·2, 1·2) | 0·3 (-0·1, 0·7) | 0·3 (-0·3, 0·7) | 1·0 (0·6, 1·6) |
|  | SW | 40 | -3·1 (-4·1, -2·5) | -2·4 (-3·1, -1·7) | -1·7 (-2·4, -1·0) | -1·6 (-2·2, -0·8) | 1·0 (0·2, 1·5) | 0·6 (0·2, 1·1) | 0·0 (-0·5, 0·7) | 1·7 (0·6, 2·5) |
|  | NO | 20 | -3·9 (-4·4, -2·3) | -1·8 (-2·7, -1·1) | -1·4 (-1·9, -0·8) | -0·7 (-1·8, -0·3) | 1·7 (1·0, 2·4) | 0·3 (-0·1, 0·9) | 0·5 (0·0, 0·8) | 2·2 (1·4, 3·1) |
| Kampala | NW | 122 | -1·0 (-1·4, -0·4) | -0·7 (-1·3, -0·1) | -0·7 (-1·2, -0·2) | -0·6 (-1·0, -0·1) | 0·3 (-0·1, 0·5) | 0·0 (-0·3, 0·4) | 0·1 (-0·3, 0·5) | 0·3 (-0·3, 0·8) |
|  | MW | 82 | -2·1 (-2·5, -1·8) | -1·6 (-2·2, -1·1) | -1·4 (-2·0, -0·8) | -1·2 (-1·7, -0·7) | 0·5 (0·2, 1·0) | 0·2 (-0·2, 0·7) | 0·1 (-0·3, 0·4) | 0·8 (0·3, 1·3) |
|  | SW | 77 | -3·2 (-4·0, -2·5) | -2·6 (-3·4, -1·8) | -2·1 (-3·0, -1·4) | -1·7 (-2·6, -1·1) | 0·7 (-0·1, 1·1) | 0·4 (0·0, 0·9) | 0·4 (-0·3, 0·8) | 1·3 (0·6, 2·0) |
|  | NO | 103 | -2·8 (-3·6, -1·6) | -1·7 (-2·4, -1·0) | -1·3 (-2·0, -0·6) | -0·8 (-1·7, -0·2) | 1·0 (0·5, 1·4) | 0·4 (0·0, 0·7) | 0·4 (-0·1, 0·8) | 1·8 (1·0, 2·4) |
| Kilifi | NW | 105 | -0·4 (-1·0, 0·3) | -0·2 (-0·7, 0·5) | -0·2 (-0·9, 0·6) | -0·3 (-0·9, 0·4) | 0·2 (-0·1, 0·5) | 0·0 (-0·3, 0·4) | -0·1 (-0·4, 0·2) | 0·1 (-0·5, 0·5) |
|  | MW | 31 | -2·0 (-2·3, -1·7) | -1·6 (-2·0, -1·1) | -1·5 (-1·9, -1·0) | -1·4 (-1·6, -0·9) | 0·5 (0·0, 0·7) | 0·1 (-0·2, 0·4) | 0·1 (-0·4, 0·3) | 0·7 (0·2, 1·0) |
|  | SW | 47 | -3·1 (-3·7, -2·5) | -2·3 (-3·1, -1·9) | -2·2 (-2·8, -1·5) | -2·0 (-2·9, -1·3) | 0·5 (-0·2, 1·3) | 0·2 (-0·3, 0·6) | 0·3 (-0·5, 0·7) | 1·1 (0·0, 1·8) |
|  | NO | 17 | -2·8 (-3·9, -1·0) | -1·5 (-2·0, -0·4) | -1·2 (-1·7, -0·5) | -1·1 (-1·3, -0·5) | 1·2 (0·5, 1·7) | 0·4 (-0·1, 0·8) | 0·4 (-0·2, 0·7) | 1·5 (1·1, 2·4) |
| Migori | NW | 67 | -0·8 (-1·2, -0·2) | -0·4 (-1·0, 0·2) | -0·1 (-0·8, 0·6) | 0·0 (-1·0, 0·5) | 0·2 (-0·1, 0·6) | 0·2 (-0·1, 0·5) | 0·1 (-0·2, 0·5) | 0·5 (0·0, 1·0) |
|  | MW | 29 | -2·1 (-2·4, -1·7) | -1·6 (-1·8, -1·1) | -1·1 (-1·3, -0·6) | -1·0 (-1·6, -0·4) | 0·5 (0·1, 1·0) | 0·4 (0·3, 0·8) | 0·2 (-0·1, 0·5) | 1·3 (0·6, 1·5) |
|  | SW | 36 | -3·7 (-4·8, -3·0) | -2·6 (-3·1, -2·2) | -2·2 (-3·1, -1·5) | -1·6 (-2·3, -0·9) | 1·2 (0·3, 2·0) | 0·6 (0·0, 1·0) | 0·8 (0·2, 1·3) | 2·1 (1·4, 2·7) |
|  | NO | 35 | -2·9 (-4·0, -2·2) | -2·0 (-2·6, -1·5) | -1·5 (-2·7, -0·8) | -1·0 (-1·6, -0·3) | 0·6 (0·0, 1·3) | 0·3 (0·1, 0·9) | 0·6 (0·1, 1·1) | 1·9 (1·2, 3·1) |
| Nairobi | NW | 77 | -0·6 (-1·2, 0·2) | -0·4 (-1·1, 0·3) | -0·4 (-0·9, 0·3) | -0·2 (-0·9, 0·5) | 0·0 (-0·3, 0·4) | 0·0 (-0·2, 0·2) | 0·3 (-0·1, 0·6) | 0·2 (-0·4, 1·0) |
|  | MW | 45 | -2·1 (-2·6, -1·8) | -1·9 (-2·5, -1·4) | -1·5 (-2·4, -1·2) | -1·4 (-2·1, -0·8) | 0·2 (-0·2, 0·7) | 0·4 (0·0, 0·6) | 0·2 (-0·1, 0·6) | 0·7 (0·3, 1·3) |
|  | SW | 70 | -3·0 (-4·0, -2·4) | -2·4 (-3·1, -1·7) | -2·2 (-3·0, -1·5) | -1·9 (-2·6, -1·3) | 0·5 (0·1, 1·1) | 0·2 (-0·1, 0·7) | 0·2 (0·0, 0·6) | 1·0 (0·2, 1·8) |
|  | NO | 10 | -1·5 (-2·4, -1·3) | -1·6 (-1·8, -1·2) | -1·0 (-1·3, -1·0) | -1·3 (-1·8, -0·7) | 0·1 (-0·1, 0·4) | 0·2 (0·1, 0·5) | 0·3 (-0·1, 0·6) | 0·7 (-0·3, 1·6) |
| Karachi | NW | 128 | -1·3 (-1·8, -0·7) | -1·1 (-1·6, -0·3) | -0·9 (-1·6, -0·1) | -0·6 (-1·3, 0·0) | 0·2 (-0·1, 0·5) | 0·2 (0·0, 0·5) | 0·1 (-0·1, 0·6) | 0·6 (0·1, 1·0) |
|  | MW | 64 | -2·3 (-2·6, -1·9) | -1·8 (-2·2, -1·3) | -1·6 (-2·0, -1·1) | -1·2 (-1·9, -0·7) | 0·4 (0·2, 1·0) | 0·3 (0·0, 0·4) | 0·3 (0·0, 0·5) | 0·9 (0·5, 1·5) |
|  | SW | 72 | -3·8 (-4·7, -3·2) | -3·0 (-3·7, -2·3) | -2·5 (-3·2, -1·7) | -2·0 (-2·6, -1·2) | 0·7 (0·4, 1·3) | 0·6 (0·2, 1·1) | 0·6 (0·0, 0·9) | 1·8 (1·3, 2·7) |
|  | NO | 18 | -3·2 (-3·6, -2·2) | -2·6 (-3·3, -1·5) | -1·9 (-2·6, -1·1) | -1·4 (-1·7, -0·3) | 0·7 (0·3, 0·8) | 0·3 (0·0, 0·9) | 0·4 (0·2, 1·1) | 1·7 (0·6, 3·0) |
| Matlab | NW | 89 | -1·2 (-1·7, -0·6) | -0·8 (-1·4, -0·3) | -0·6 (-1·1, 0·1) | -0·4 (-1·0, 0·1) | 0·3 (0·1, 0·6) | 0·2 (0·0, 0·4) | 0·1 (-0·1, 0·3) | 0·6 (0·3, 1·1) |
|  | MW | 100 | -2·3 (-2·7, -2·0) | -1·8 (-2·2, -1·3) | -1·7 (-2·0, -1·2) | -1·4 (-1·9, -1·0) | 0·6 (0·2, 0·9) | 0·1 (-0·1, 0·4) | 0·1 (-0·1, 0·4) | 0·8 (0·4, 1·4) |
|  | SW | 89 | -3·0 (-3·3, -2·7) | -2·3 (-2·7, -1·9) | -2·2 (-2·6, -1·7) | -1·9 (-2·3, -1·5) | 0·7 (0·3, 1·1) | 0·1 (-0·1, 0·3) | 0·1 (-0·1, 0·4) | 1·0 (0·7, 1·5) |
|  | NO | 9 | -1·3 (-1·4, -0·5) | -0·6 (-1·1, 0·0) | -0·2 (-1·1, -0·1) | -0·2 (-0·4, 0·5) | 0·6 (0·4, 0·7) | 0·3 (0·0, 0·5) | 0·3 (0·1, 0·4) | 1·1 (1·0, 1·2) |
| Dhaka | NW | 116 | -0·7 (-1·3, 0·0) | -0·4 (-1·1, 0·2) | -0·2 (-0·8, 0·4) | -0·3 (-0·8, 0·4) | 0·4 (0·0, 0·7) | 0·2 (-0·1, 0·4) | 0·0 (-0·3, 0·3) | 0·5 (0·1, 0·9) |
|  | MW | 97 | -2·3 (-2·9, -1·7) | -1·6 (-2·3, -1·1) | -1·5 (-2·0, -0·9) | -1·3 (-1·9, -0·7) | 0·6 (0·3, 1·0) | 0·3 (0·0, 0·6) | 0·1 (-0·3, 0·5) | 0·9 (0·5, 1·5) |
|  | SW | 138 | -3·1 (-3·5, -2·7) | -2·2 (-2·9, -1·8) | -2·1 (-2·5, -1·6) | -1·8 (-2·4, -1·4) | 0·7 (0·4, 1·1) | 0·2 (-0·1, 0·6) | 0·2 (-0·1, 0·6) | 1·2 (0·7, 1·9) |
|  | NO | 16 | -3·2 (-3·6, -2·2) | -2·1 (-3·4, -0·7) | -1·9 (-2·3, -1·1) | -1·1 (-1·9, -0·7) | 0·9 (0·0, 1·8) | 0·4 (-0·5, 0·6) | 0·4 (0·0, 0·9) | 1·5 (0·9, 2·4) |

Data presented as median (IQR). Groups: NW, no wasting; MW, moderate wasting; SW, severe wasting; NO, nutritional oedema. Growth metrics: MUACZ, mid upper arm circumference z-score.

### **Supplemental Table 19.** Summary absolute of weight-for-length z-score (WLZ) at each timepoint and differences between time points split by nutritional groups and site.

|  |  |  | Weight-for-length | | | | Delta of weight-for-length | | | |
| --- | --- | --- | --- | --- | --- | --- | --- | --- | --- | --- |
|  | | n | Discharge | 45-days | 90-days | 180-days | △Discharge-to-D45 | △D45-to-D90 | △D90-to-D180 | Total growth |
| Banfora | NW | 118 | -0·6 (-1·3, -0·1) | -0·4 (-0·9, 0·1) | -0·3 (-0·8, 0·1) | -0·3 (-1·1, 0·3) | 0·2 (-0·2, 0·7) | 0·1 (-0·2, 0·3) | -0·2 (-0·5, 0·4) | 0·3 (-0·2, 0·9) |
|  | MW | 87 | -1·7 (-2·0, -1·3) | -1·5 (-2·0, -1·0) | -1·2 (-1·9, -0·7) | -1·1 (-1·5, -0·5) | 0·2 (-0·1, 0·6) | 0·2 (-0·3, 0·5) | 0·2 (-0·1, 0·6) | 0·5 (0·1, 1·1) |
|  | SW | 113 | -3·1 (-3·9, -2·4) | -2·2 (-3·1, -1·4) | -1·8 (-2·9, -1·2) | -1·6 (-2·5, -0·8) | 0·8 (0·0, 1·5) | 0·2 (-0·3, 0·6) | 0·2 (-0·1, 0·8) | 1·2 (0·4, 2·3) |
|  | NO | 30 | -2·9 (-3·7, -2·1) | -1·3 (-2·2, -0·5) | -1·4 (-2·3, -0·2) | -0·5 (-1·8, 0·1) | 1·2 (0·5, 2·1) | 0·0 (-0·2, 0·4) | 0·4 (0·0, 1·2) | 1·9 (0·9, 2·9) |
| Blantyre | NW | 138 | -0·3 (-1·0, 0·6) | 0·0 (-0·6, 0·7) | 0·1 (-0·6, 0·8) | 0·0 (-0·7, 0·7) | 0·2 (-0·2, 0·7) | 0·1 (-0·2, 0·5) | -0·2 (-0·5, 0·2) | 0·3 (-0·4, 0·8) |
|  | MW | 37 | -1·9 (-2·4, -1·1) | -1·0 (-1·5, -0·6) | -1·0 (-1·5, -0·2) | -0·8 (-1·7, -0·5) | 0·6 (0·2, 1·0) | 0·2 (-0·3, 0·5) | 0·1 (-0·5, 0·5) | 0·4 (0·0, 1·4) |
|  | SW | 40 | -3·3 (-4·1, -2·5) | -2·1 (-3·1, -0·9) | -1·6 (-2·1, -0·7) | -1·5 (-2·1, -0·8) | 1·1 (0·3, 1·8) | 0·4 (0·0, 1·3) | 0·1 (-0·3, 0·6) | 1·7 (0·5, 2·8) |
|  | NO | 20 | -3·0 (-4·0, -2·0) | -1·1 (-1·8, -0·3) | -0·8 (-1·3, -0·5) | -0·1 (-1·0, 0·3) | 2·0 (0·9, 2·5) | 0·2 (-0·4, 0·7) | 0·7 (0·2, 1·0) | 2·3 (1·4, 3·3) |
| Kampala | NW | 122 | -0·3 (-0·9, 0·3) | -0·1 (-0·6, 0·5) | -0·1 (-0·8, 0·5) | -0·1 (-0·7, 0·4) | 0·3 (-0·1, 0·7) | 0·0 (-0·4, 0·4) | 0·0 (-0·4, 0·4) | 0·2 (-0·3, 0·8) |
|  | MW | 82 | -1·7 (-2·1, -1·2) | -1·0 (-1·7, -0·4) | -1·0 (-1·5, -0·5) | -0·8 (-1·4, 0·0) | 0·6 (0·1, 1·0) | 0·1 (-0·3, 0·5) | 0·1 (-0·3, 0·5) | 0·7 (0·4, 1·3) |
|  | SW | 77 | -2·7 (-3·3, -2·0) | -2·0 (-2·8, -1·2) | -1·7 (-2·3, -1·0) | -1·5 (-2·4, -0·6) | 0·7 (-0·2, 1·2) | 0·3 (-0·2, 0·8) | 0·2 (-0·3, 0·8) | 1·3 (0·4, 2·2) |
|  | NO | 103 | -2·1 (-2·8, -1·1) | -0·9 (-1·6, -0·3) | -0·6 (-1·5, 0·2) | -0·2 (-1·0, 0·6) | 1·1 (0·5, 1·6) | 0·3 (-0·2, 0·9) | 0·4 (-0·1, 0·8) | 1·7 (0·8, 2·4) |
| Kilifi | NW | 105 | -0·3 (-1·1, 0·4) | 0·0 (-0·7, 0·6) | -0·3 (-0·8, 0·7) | -0·2 (-1·0, 0·5) | 0·2 (-0·1, 0·6) | -0·1 (-0·4, 0·2) | 0·0 (-0·3, 0·4) | 0·0 (-0·4, 0·7) |
|  | MW | 31 | -2·3 (-2·5, -2·0) | -1·7 (-2·1, -0·8) | -1·4 (-1·9, -1·0) | -1·3 (-1·7, -0·9) | 0·6 (0·3, 1·1) | 0·1 (-0·3, 0·4) | 0·0 (-0·5, 0·3) | 0·7 (0·3, 1·2) |
|  | SW | 47 | -3·0 (-3·6, -2·4) | -2·4 (-3·2, -1·6) | -2·3 (-3·0, -1·2) | -1·9 (-2·6, -1·2) | 0·6 (-0·2, 1·3) | 0·3 (-0·1, 0·7) | 0·1 (-0·4, 0·5) | 1·1 (0·2, 1·6) |
|  | NO | 17 | -2·1 (-3·5, -0·7) | -1·0 (-2·0, -0·2) | -0·8 (-1·5, -0·1) | -0·2 (-0·8, 0·8) | 1·1 (0·0, 1·7) | 0·1 (0·0, 0·7) | 0·8 (0·3, 1·3) | 1·8 (1·1, 2·8) |
| Migori | NW | 67 | -0·2 (-0·9, 0·5) | 0·2 (-0·6, 0·7) | 0·3 (-0·6, 1·1) | 0·2 (-0·5, 0·9) | 0·3 (-0·1, 0·7) | 0·2 (-0·3, 0·5) | 0·0 (-0·5, 0·5) | 0·3 (-0·4, 1·0) |
|  | MW | 29 | -1·9 (-2·4, -1·1) | -1·1 (-1·5, -0·8) | -1·1 (-1·7, -0·2) | -0·7 (-1·5, -0·2) | 0·7 (0·1, 1·1) | 0·2 (-0·5, 0·8) | 0·1 (-0·2, 0·7) | 0·8 (0·0, 1·3) |
|  | SW | 36 | -3·2 (-4·0, -2·4) | -2·3 (-3·1, -1·4) | -1·8 (-2·7, -1·5) | -1·3 (-2·2, -0·5) | 0·8 (0·0, 2·0) | 0·1 (-0·2, 0·9) | 0·6 (0·1, 1·3) | 1·8 (1·1, 2·8) |
|  | NO | 35 | -2·8 (-3·7, -1·8) | -1·7 (-2·9, -1·0) | -0·9 (-1·9, -0·4) | -0·4 (-1·2, 0·3) | 0·5 (-0·4, 1·7) | 0·6 (0·1, 1·2) | 0·5 (0·1, 1·7) | 2·2 (1·4, 3·1) |
| Nairobi | NW | 77 | -0·1 (-1·1, 0·5) | -0·1 (-0·8, 0·8) | -0·1 (-0·9, 0·7) | -0·2 (-0·8, 0·6) | 0·2 (-0·2, 0·6) | 0·0 (-0·4, 0·3) | 0·0 (-0·4, 0·4) | 0·0 (-0·6, 0·6) |
|  | MW | 45 | -2·0 (-2·5, -1·7) | -1·9 (-2·4, -1·1) | -1·5 (-2·0, -0·9) | -1·3 (-2·0, -0·7) | 0·3 (-0·3, 0·7) | 0·4 (0·0, 0·7) | 0·2 (-0·2, 0·4) | 0·8 (0·1, 1·2) |
|  | SW | 70 | -3·1 (-3·7, -2·6) | -2·3 (-3·3, -1·5) | -2·4 (-3·0, -1·1) | -2·2 (-2·9, -1·1) | 0·7 (0·2, 1·5) | 0·0 (-0·3, 0·5) | 0·1 (-0·2, 0·6) | 0·7 (0·1, 2·0) |
|  | NO | 10 | -1·7 (-2·2, -1·1) | -1·3 (-1·6, -0·9) | -1·0 (-1·4, -0·6) | -0·4 (-1·0, -0·2) | 0·5 (-0·1, 0·8) | 0·3 (0·2, 0·5) | 0·7 (-0·2, 1·2) | 1·3 (0·5, 2·4) |
| Karachi | NW | 128 | -0·9 (-1·6, -0·2) | -0·4 (-1·2, 0·3) | -0·3 (-1·1, 0·4) | -0·3 (-1·2, 0·5) | 0·3 (-0·2, 0·9) | 0·2 (-0·1, 0·6) | 0·1 (-0·5, 0·6) | 0·5 (-0·3, 1·1) |
|  | MW | 64 | -1·9 (-2·5, -1·2) | -1·4 (-1·9, -0·8) | -1·0 (-1·7, -0·5) | -0·7 (-1·3, -0·1) | 0·5 (-0·2, 1·0) | 0·2 (-0·2, 0·8) | 0·3 (-0·1, 0·8) | 1·0 (0·2, 1·6) |
|  | SW | 72 | -3·3 (-3·9, -2·7) | -2·2 (-3·3, -1·5) | -1·9 (-2·6, -0·7) | -1·5 (-2·0, -0·5) | 1·0 (0·3, 1·8) | 0·7 (0·0, 1·2) | 0·5 (-0·1, 1·0) | 1·9 (0·9, 3·0) |
|  | NO | 18 | -2·6 (-3·3, -1·3) | -1·8 (-2·6, -0·7) | -1·2 (-1·5, -0·6) | -1·3 (-1·6, 0·4) | 0·5 (0·2, 1·2) | 0·4 (-0·2, 1·7) | 0·8 (-0·5, 1·1) | 1·4 (0·8, 2·6) |
| Matlab | NW | 89 | -1·1 (-1·5, -0·5) | -0·6 (-1·1, 0·3) | -0·4 (-1·0, 0·3) | -0·6 (-1·2, 0·0) | 0·4 (0·1, 0·8) | 0·1 (-0·2, 0·4) | -0·2 (-0·5, 0·2) | 0·5 (-0·1, 0·8) |
|  | MW | 100 | -2·3 (-2·6, -1·8) | -1·5 (-2·0, -1·1) | -1·7 (-2·1, -1·2) | -1·6 (-2·0, -1·1) | 0·7 (0·2, 1·1) | -0·1 (-0·3, 0·2) | 0·0 (-0·3, 0·3) | 0·6 (0·1, 1·2) |
|  | SW | 89 | -3·0 (-3·4, -2·6) | -2·2 (-2·6, -1·7) | -2·3 (-2·6, -1·8) | -2·2 (-2·7, -1·8) | 0·8 (0·4, 1·1) | 0·0 (-0·4, 0·2) | 0·0 (-0·4, 0·4) | 0·6 (0·3, 1·2) |
|  | NO | 9 | -0·7 (-1·8, -0·4) | -0·7 (-0·8, 0·2) | -0·1 (-0·7, 0·4) | -0·1 (-0·5, 0·0) | 0·7 (0·3, 0·9) | 0·2 (0·1, 0·6) | -0·4 (-0·5, 0·2) | 0·8 (0·3, 1·3) |
| Dhaka | NW | 116 | -0·8 (-1·4, -0·2) | -0·3 (-0·8, 0·4) | -0·3 (-0·8, 0·3) | -0·3 (-0·8, 0·2) | 0·4 (0·1, 0·9) | 0·0 (-0·3, 0·3) | 0·0 (-0·4, 0·4) | 0·5 (0·0, 1·0) |
|  | MW | 97 | -2·3 (-2·6, -1·8) | -1·7 (-2·1, -1·0) | -1·5 (-1·9, -1·0) | -1·5 (-2·0, -1·0) | 0·6 (0·1, 1·1) | 0·1 (-0·2, 0·6) | 0·0 (-0·5, 0·3) | 0·7 (0·2, 1·3) |
|  | SW | 138 | -3·1 (-3·6, -2·6) | -2·3 (-2·9, -1·8) | -2·3 (-2·9, -1·7) | -2·0 (-2·6, -1·4) | 0·7 (0·3, 1·2) | 0·2 (-0·3, 0·5) | 0·1 (-0·2, 0·5) | 0·9 (0·5, 1·9) |
|  | NO | 16 | -1·8 (-2·4, -1·1) | -0·9 (-2·9, 0·0) | -1·1 (-1·6, -0·4) | -0·9 (-1·3, -0·6) | 0·5 (-0·4, 1·9) | -0·1 (-0·9, 0·7) | 0·2 (-0·1, 0·7) | 1·0 (0·0, 1·9) |

Data presented as median (IQR). Groups: NW, no wasting; MW, moderate wasting; SW, severe wasting; NO, nutritional oedema. Growth metrics: WLZ, weight-for-length z-score.

**
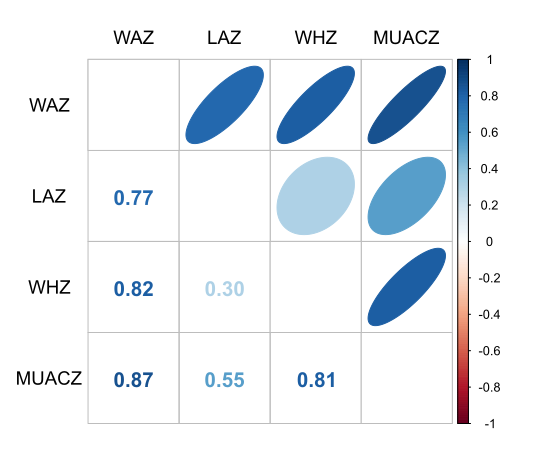
****Supplemental Figure 9**. Pairwise correlation plot between anthropometric measures. Scale bar indicates strength and direction of correlation (directional ovals of increasing blue shade indicate increasing positive correlation). The lower section shows the Pearson correlation coefficients (r). LAZ, length-for-age z-score; WAZ, weight-for-age z-score; MUACZ, mid-upper arm circumference z-scores; WLZ, weight-for-length z-score.


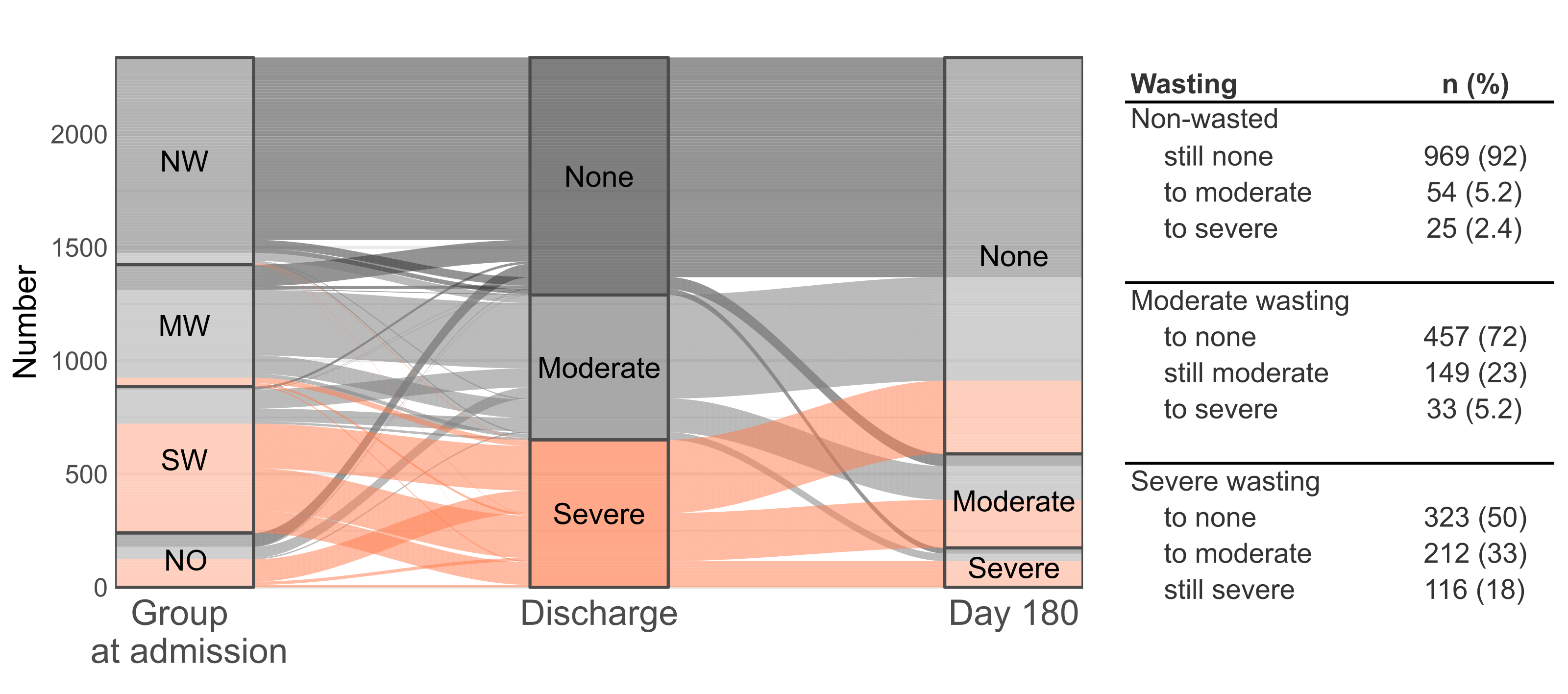


**Supplemental Figure 10.** Alluvial plots detailing the change in nutritional classification of children from discharge (center) to 180-days post discharge (left) for wasting based on WLZ. Classification is as follows: None ≥ -2 z-score (dark grey); Moderate < -2 to ≥ -3 z-score (light grey); Severe < -3 z-score (orange). Each flow line depicts how an individual child changes between initial group at admission (x-axis, left) and their nutritional classification at discharge (center), and 180-days post-discharge (right). Y-axis presents stacked child counts. Tables detail the number and percentage of children in each trajectory pattern seen between discharge and 180-days. WLZ, weight-for-length z-score. Groups: NW, no wasting; MW, moderate wasting; SW, severe wasting; NO, nutritional oedema.

### **Supplemental Table 20**. Change in LAZ in the 180-days post-discharge per nutritional group associated with anaemia or diarrhoea diagnosed at admission.

|  | **Base model** | | | **Anaemia** | | | **Diarrhoea** | | |
| --- | --- | --- | --- | --- | --- | --- | --- | --- | --- |
| *Predictors* | *Est.* | *95% CI* | *p* | *Est.* | *95% CI* | *p* | *Est.* | *95% CI* | *p* |
| Intercept [ref, NW] | -0·99 | -1·24, -0·73 | **<0·0001** | -0·88 | -1·16, -0·59 | **<0·0001** | -1·04 | -1·31, -0·78 | **<0·0001** |
| Slope early discharge [NW] | -0·13 | -0·15, -0·11 | **<0·0001** | -0·13 | -0·15, -0·11 | **<0·0001** | -0·13 | -0·15, -0·11 | **<0·0001** |
| Difference in slope early vs· late discharge [NW] | 0·11 | 0·09, 0·13 | **<0·0001** | 0·11 | 0·09, 0·13 | **<0·0001** | 0·11 | 0·09, 0·13 | **<0·0001** |
| Difference in intercept early discharge [MW - NW] | -0·68 | -0·83, -0·52 | **<0·0001** | -0·64 | -0·80, -0·48 | **<0·0001** | -0·69 | -0·84, -0·54 | **<0·0001** |
| Difference in intercept early discharge [SW - NW] | -1·57 | -1·71, -1·42 | **<0·0001** | -1·56 | -1·71, -1·41 | **<0·0001** | -1·58 | -1·73, -1·44 | **<0·0001** |
| Difference in intercept early discharge [NO - NW] | -1·6 | -1·81, -1·40 | **<0·0001** | -1·59 | -1·80, -1·37 | **<0·0001** | -1·61 | -1·82, -1·40 | **<0·0001** |
| Age, months | -0·01 | -0·02, -0·00 | **0·020** | -0·01 | -0·02, -0·00 | **0·020** | -0·01 | -0·02, -0·00 | **0·022** |
| Sex, male | -0·31 | -0·42, -0·21 | **<0·0001** | -0·28 | -0·38, -0·17 | **<0·0001** | -0·31 | -0·41, -0·21 | **<0·0001** |
| Difference in slope early discharge [MW - NW] | -0·02 | -0·05, 0·01 | 0·26 | -0·02 | -0·06, 0·01 | 0·18 | -0·02 | -0·05, 0·01 | 0·26 |
| Difference in slope early discharge [SW - NW] | 0·01 | -0·02, 0·04 | 0·51 | 0·01 | -0·02, 0·04 | 0·502 | 0·01 | -0·02, 0·04 | 0·511 |
| Difference in slope early discharge [NO - NW] | 0·07 | 0·03, 0·11 | **0·0011** | 0·07 | 0·02, 0·11 | **0·0033** | 0·07 | 0·03, 0·11 | **0·0011** |
| Difference in change of slope between early and late discharge [MW - NW] | 0·03 | -0·00, 0·07 | 0·050 | 0·04 | 0·00, 0·08 | **0·029** | 0·03 | -0·00, 0·07 | 0·050 |
| Difference in change of slope between early and late discharge [SW - NW] | 0·03 | -0·01, 0·06 | 0·10 | 0·03 | -0·01, 0·06 | 0·10 | 0·03 | -0·01, 0·06 | 0·10 |
| Difference in change of slope between early and late discharge [NO - NW] | -0·04 | -0·08, 0·01 | 0·13 | -0·03 | -0·08, 0·02 | 0·23 | -0·04 | -0·08, 0·01 | 0·13 |
| Diarrhoea |  |  |  |  |  |  | 0·12 | 0·01, 0·24 | **0·038** |
| Anemia [Mild] |  |  |  | -0·01 | -0·16, 0·15 | 0·94 |  |  |  |
| Anemia [Moderate / Severe] |  |  |  | -0·24 | -0·37, -0·10 | **0·0090** |  |  |  |
| **Random Effects** |  |  |  |  |  |  |  |  |  |
| σ^2^ | 0·07 | | | 0·07 | | | 0·07 | | |
| τ_00_ | 2·03 record_id | | | 2·01 record_id | | | 2·03 record_id | | |
|  | 0·10 site | | | 0·11 site | | | 0·10 site | | |
| τ_11_ | 0·04 record_id·time_months | | | 0·04 record_id·time_months | | | 0·04 record_id·time_months | | |
|  | 0·02 record_id·I(pmax(time_months - 1·5, 0)) | | | 0·02 record_id·I(pmax(time_months - 1·5, 0)) | | | 0·02 record_id·I(pmax(time_months - 1·5, 0)) | | |
| ρ_01_ | -0·41 record_id·time_months | | | -0·41 record_id·time_months | | | -0·41 record_id·time_months | | |
|  | 0·20 record_id·I(pmax(time_months - 1·5, 0)) | | | 0·19 record_id·I(pmax(time_months - 1·5, 0)) | | | 0·20 record_id·I(pmax(time_months - 1·5, 0)) | | |
| ICC | 0·96 | | | 0·97 | | | 0·96 | | |
| N | 9 _site_ | | | 9 _site_ | | | 9 _site_ | | |
|  | 2461 _record_id_ | | | 2357 _record_id_ | | | 2461 _record_id_ | | |
| Observations | 9425 | | | 9038 | | | 9425 | | |
| Marginal R^2^ / Conditional R^2^ | 0·196 / 0·972 | | | 0·201 / 0·972 | | | 0·197 / 0·972 | | |
| AIC | 17116·528 | | | 16388·101 | | | 17118·063 | | |
| AICc | 17116·636 | | | 16388·234 | | | 17118·18 | | |

Table presents results for both fixed and random terms of mixed piecewise models fit using *lme4* R package. A knot point was positioned at 45-days post-discharge which defines two discharge phases (i.e., early, before 45-days and, late, after 45-days). Time was coded in months (i.e., 1·5 months corresponds to 45-days; and 3 months to 90-days). Random structure included random slopes per participant and random intercepts for site with participants nested within. Models were fit with maximum likelihood for comparison with anova using Satterthwaite's approximation of degrees of freedom as implemented in the *lmerTest* R package (Kuznetsova, A. et al., 2017). Models were evaluated using fit metrics AIC, AICc, and BIC. Restricted maximum likelihood was used to fit final models. Groups: NW, no wasting; MW, moderate wasting; SW, severe wasting; NO, nutritional oedema. LAZ, length-for-age z-score.

### **Supplemental Table 21**. Change in LAZ in the 180-days post-discharge per nutritional group associated with sepsis or pneumonia diagnosed at admission.

|  | **Base model** | | | **Sepsis** | | | **Pneumonia** | | |
| --- | --- | --- | --- | --- | --- | --- | --- | --- | --- |
| *Predictors* | *Est.* | *95% CI* | *p* | *Est.* | *95% CI* | *p* | *Est.* | *95% CI* | *p* |
| Intercept [ref, NW] | -0·99 | -1·24, -0·73 | **<0·0001** | -1 | -1·26, -0·74 | **<0·0001** | -0·95 | -1·21, -0·70 | **<0·0001** |
| Slope early discharge [NW] | -0·13 | -0·15, -0·11 | **<0·0001** | -0·13 | -0·15, -0·11 | **<0·0001** | -0·13 | -0·15, -0·11 | **<0·0001** |
| Difference in slope early vs· late discharge [NW] | 0·11 | 0·09, 0·13 | **<0·0001** | 0·11 | 0·09, 0·13 | **<0·0001** | 0·11 | 0·09, 0·13 | **<0·0001** |
| Difference in intercept early discharge [MW - NW] | -0·68 | -0·83, -0·52 | **<0·0001** | -0·68 | -0·83, -0·53 | **<0·0001** | -0·68 | -0·83, -0·53 | **<0·0001** |
| Difference in intercept early discharge [SW - NW] | -1·57 | -1·71, -1·42 | **<0·0001** | -1·57 | -1·71, -1·42 | **<0·0001** | -1·58 | -1·72, -1·43 | **<0·0001** |
| Difference in intercept early discharge [NO - NW] | -1·6 | -1·81, -1·40 | **<0·0001** | -1·6 | -1·81, -1·39 | **<0·0001** | -1·62 | -1·83, -1·41 | **<0·0001** |
| Age, months | -0·01 | -0·02, -0·00 | **0·020** | -0·01 | -0·02, -0·00 | **0·020** | -0·01 | -0·02, -0·00 | **0·017** |
| Sex, male | -0·31 | -0·42, -0·21 | **<0·0001** | -0·31 | -0·42, -0·21 | **<0·0001** | -0·31 | -0·41, -0·21 | **<0·0001** |
| Difference in slope early discharge [MW - NW] | -0·02 | -0·05, 0·01 | 0·26 | -0·02 | -0·05, 0·01 | 0·26 | -0·02 | -0·05, 0·01 | 0·26 |
| Difference in slope early discharge [SW - NW] | 0·01 | -0·02, 0·04 | 0·51 | 0·01 | -0·02, 0·04 | 0·51 | 0·01 | -0·02, 0·04 | 0·511 |
| Difference in slope early discharge [NO - NW] | 0·07 | 0·03, 0·11 | **0·0011** | 0·07 | 0·03, 0·11 | **0·0011** | 0·07 | 0·03, 0·11 | **0·0011** |
| Difference in change of slope between early and late discharge [MW - NW] | 0·03 | -0·00, 0·07 | 0·050 | 0·03 | -0·00, 0·07 | 0·050 | 0·03 | -0·00, 0·07 | 0·050 |
| Difference in change of slope between early and late discharge [SW - NW] | 0·03 | -0·01, 0·06 | 0·10 | 0·03 | -0·01, 0·06 | 0·10 | 0·03 | -0·01, 0·06 | 0·10 |
| Difference in change of slope between early and late discharge [NO - NW] | -0·04 | -0·08, 0·01 | 0·13 | -0·04 | -0·08, 0·01 | 0·13 | -0·04 | -0·08, 0·01 | 0·13 |
| Sepsis |  |  |  | 0·08 | -0·08, 0·25 | 0·33 |  |  |  |
| Pneumonia |  |  |  |  |  |  | -0·07 | -0·19, 0·05 | 0·23 |
| **Random Effects** |  |  |  |  |  |  |  |  |  |
| σ^2^ | 0·07 | | | 0·07 | | | 0·07 | | |
| τ_00_ | 2·03 record_id | | | 2·03 record_id | | | 2·03 record_id | | |
|  | 0·10 site | | | 0·10 site | | | 0·10 site | | |
| τ_11_ | 0·04 record_id·time_months | | | 0·04 record_id·time_months | | | 0·04 record_id·time_months | | |
|  | 0·02 record_id·I(pmax(time_months - 1·5, 0)) | | | 0·02 record_id·I(pmax(time_months - 1·5, 0)) | | | 0·02 record_id·I(pmax(time_months - 1·5, 0)) | | |
| ρ_01_ | -0·41 record_id·time_months | | | -0·41 record_id·time_months | | | -0·41 record_id·time_months | | |
|  | 0·20 record_id·I(pmax(time_months - 1·5, 0)) | | | 0·20 record_id·I(pmax(time_months - 1·5, 0)) | | | 0·19 record_id·I(pmax(time_months - 1·5, 0)) | | |
| ICC | 0·96 | | | 0·97 | | | 0·96 | | |
| N | 9 _site_ | | | 9 _site_ | | | 9 _site_ | | |
|  | 2461 _record_id_ | | | 2461 _record_id_ | | | 2461 _record_id_ | | |
| Observations | 9425 | | | 9425 | | | 9425 | | |
| Marginal R^2^ / Conditional R^2^ | 0·196 / 0·972 | | | 0·196 / 0·972 | | | 0·197 / 0·972 | | |
| AIC | 17116·528 | | | 17120·703 | | | 17120·877 | | |
| AICc | 17116·636 | | | 17120·821 | | | 17120·994 | | |

Table presents results for both fixed and random terms of mixed piecewise models fit using *lme4* R package. A knot point was positioned at 45-days post-discharge which defines two discharge phases (i.e., early, before 45-days and, late, after 45-days). Time was coded in months (i.e., 1·5 months corresponds to 45-days; and 3 months to 90-days). Random structure included random slopes per participant and random intercepts for site with participants nested within. Models were fit with maximum likelihood for comparison with anova using Satterthwaite's approximation of degrees of freedom as implemented in the *lmerTest* R package (Kuznetsova, A. et al., 2017). Models were evaluated using fit metrics AIC, AICc, and BIC. Restricted maximum likelihood was used to fit final models. Groups: NW, no wasting; MW, moderate wasting; SW, severe wasting; NO, nutritional oedema. LAZ, length-for-age z-score.

### **Supplemental Table 22**. Change in LAZ in the 180-days post-discharge per nutritional group associated with exposure domains of illness severity at admission and at discharge.

|  | **Base model** | | | **Illness severity  at admission** | | | **Illness severity  at discharge** | | |
| --- | --- | --- | --- | --- | --- | --- | --- | --- | --- |
| *Predictors* | *Est.* | *95% CI* | *p* | *Est.* | *95% CI* | *p* | *Est.* | *95% CI* | *p* |
| Intercept [ref, NW] | -0·99 | -1·24, -0·73 | **<0·0001** | -1·01 | -1·28, -0·74 | **<0·0001** | -0·97 | -1·22, -0·71 | **<0·0001** |
| Slope early discharge [NW] | -0·13 | -0·15, -0·11 | **<0·0001** | -0·13 | -0·15, -0·11 | **<0·0001** | -0·13 | -0·15, -0·11 | **<0·0001** |
| Difference in slope early vs· late discharge [NW] | 0·11 | 0·09, 0·13 | **<0·0001** | 0·11 | 0·09, 0·13 | **<0·0001** | 0·11 | 0·09, 0·13 | **<0·0001** |
| Difference in intercept early discharge [MW - NW] | -0·68 | -0·83, -0·52 | **<0·0001** | -0·67 | -0·82, -0·52 | **<0·0001** | -0·68 | -0·83, -0·53 | **<0·0001** |
| Difference in intercept early discharge [SW - NW] | -1·57 | -1·71, -1·42 | **<0·0001** | -1·57 | -1·71, -1·42 | **<0·0001** | -1·57 | -1·71, -1·43 | **<0·0001** |
| Difference in intercept early discharge [NO - NW] | -1·6 | -1·81, -1·40 | **<0·0001** | -1·6 | -1·81, -1·39 | **<0·0001** | -1·61 | -1·82, -1·40 | **<0·0001** |
| Age, months | -0·01 | -0·02, -0·00 | **0·020** | -0·01 | -0·02, -0·00 | **0·014** | -0·01 | -0·02, -0·00 | **0·022** |
| Sex, male | -0·31 | -0·42, -0·21 | **<0·0001** | -0·31 | -0·41, -0·21 | **<0·0001** | -0·32 | -0·42, -0·21 | **<0·0001** |
| Difference in slope early discharge [MW - NW] | -0·02 | -0·05, 0·01 | 0·262 | -0·02 | -0·05, 0·01 | 0·262 | -0·02 | -0·05, 0·01 | 0·261 |
| Difference in slope early discharge [SW - NW] | 0·01 | -0·02, 0·04 | 0·51 | 0·01 | -0·02, 0·04 | 0·51 | 0·01 | -0·02, 0·04 | 0·511 |
| Difference in slope early discharge [NO - NW] | 0·07 | 0·03, 0·11 | **0·0011** | 0·07 | 0·03, 0·11 | **0·0011** | 0·07 | 0·03, 0·11 | **0·0012** |
| Difference in change of slope between early and late discharge [MW - NW] | 0·03 | -0·00, 0·07 | 0·05 | 0·03 | -0·00, 0·07 | 0·051 | 0·03 | -0·00, 0·07 | 0·05 |
| Difference in change of slope between early and late discharge [SW - NW] | 0·03 | -0·01, 0·06 | 0·101 | 0·03 | -0·01, 0·06 | 0·101 | 0·03 | -0·01, 0·06 | 0·101 |
| Difference in change of slope between early and late discharge [NO - NW] | -0·04 | -0·08, 0·01 | 0·13 | -0·04 | -0·08, 0·01 | 0·129 | -0·04 | -0·08, 0·01 | 0·13 |
| Illness severity at admission [medium] | | |  | 0·11 | -0·01, 0·24 | 0·073 |  |  |  |
| Illness severity at admission [high] | | |  | -0·04 | -0·17, 0·10 | 0·59 |  |  |  |
| Illness severity at discharge [medium or high] | | |  |  |  |  | -0·12 | -0·26, 0·03 | 0·116 |
| **Random Effects** |  |  |  |  |  |  |  |  |  |
| σ^2^ | 0·07 | | | 0·07 | | | 0·07 | | |
| τ_00_ | 2·03 record_id | | | 2·03 record_id | | | 2·03 record_id | | |
|  | 0·10 site | | | 0·10 site | | | 0·09 site | | |
| τ_11_ | 0·04 record_id·time_months | | | 0·04 record_id·time_months | | | 0·04 record_id·time_months | | |
|  | 0·02 record_id·I(pmax(time_months - 1·5, 0)) | | | 0·02 record_id·I(pmax(time_months - 1·5, 0)) | | | 0·02 record_id·I(pmax(time_months - 1·5, 0)) | | |
| ρ_01_ | -0·41 record_id·time_months | | | -0·41 record_id·time_months | | | -0·41 record_id·time_months | | |
|  | 0·20 record_id·I(pmax(time_months - 1·5, 0)) | | | 0·19 record_id·I(pmax(time_months - 1·5, 0)) | | | 0·19 record_id·I(pmax(time_months - 1·5, 0)) | | |
| ICC | 0·96 | | | 0·96 | | | 0·96 | | |
| N | 9 _site_ | | | 9 _site_ | | | 9 _site_ | | |
|  | 2461 _record_id_ | | | 2461 _record_id_ | | | 2461 _record_id_ | | |
| Observations | 9425 | | | 9425 | | | 9425 | | |
| Marginal R^2^ / Conditional R^2^ | 0·196 / 0·972 | | | 0·198 / 0·972 | | | 0·197 / 0·972 | | |
| AIC | 17116·528 | | | 17122·306 | | | 17119·423 | | |
| AICc | 17116·636 | | | 17122·434 | | | 17119·54 | | |

Table presents results for both fixed and random terms of mixed piecewise models fit using *lme4* R package. A knot point was positioned at 45-days post-discharge which defines two discharge phases (i.e., early, before 45-days and, late, after 45-days). Time was coded in months (i.e., 1·5 months corresponds to 45-days; and 3 months to 90-days). Random structure included random slopes per participant and random intercepts for site with participants nested within. Models were fit with maximum likelihood for comparison with anova using Satterthwaite's approximation of degrees of freedom as implemented in the *lmerTest* R package (Kuznetsova, A. et al., 2017). Models were evaluated using fit metrics AIC, AICc, and BIC. Restricted maximum likelihood was used to fit final models. Groups: NW, no wasting; MW, moderate wasting; SW, severe wasting; NO, nutritional oedema. LAZ, length-for-age z-score.

### **Supplemental Table 23**. Change in LAZ in the 180-days post-discharge per nutritional group associated with exposure domains of age-inappropriate nutrition and caregiver characteristics.

|  | **Base model** | | | **Age-inappropriate nutrition** | | | **Caregiver  characteristics** | | |
| --- | --- | --- | --- | --- | --- | --- | --- | --- | --- |
| *Predictors* | *Est.* | *95% CI* | *p* | *Est.* | *95% CI* | *p* | *Est.* | *95% CI* | *p* |
| Intercept [ref, NW] | -0·99 | -1·24, -0·73 | **<0·0001** | -0·95 | -1·21, -0·68 | **<0·0001** | -0·86 | -1·13, -0·59 | **<0·0001** |
| Slope early discharge [NW] | -0·13 | -0·15, -0·11 | **<0·0001** | -0·13 | -0·15, -0·11 | **<0·0001** | -0·13 | -0·15, -0·11 | **<0·0001** |
| Difference in slope early vs· late discharge [NW] | 0·11 | 0·09, 0·13 | **<0·0001** | 0·11 | 0·09, 0·13 | **<0·0001** | 0·11 | 0·09, 0·13 | **<0·0001** |
| Difference in intercept early discharge [MW - NW] | -0·68 | -0·83, -0·52 | **<0·0001** | -0·62 | -0·78, -0·47 | **<0·0001** | -0·68 | -0·83, -0·53 | **<0·0001** |
| Difference in intercept early discharge [SW - NW] | -1·57 | -1·71, -1·42 | **<0·0001** | -1·43 | -1·59, -1·28 | **<0·0001** | -1·56 | -1·70, -1·41 | **<0·0001** |
| Difference in intercept early discharge [NO - NW] | -1·6 | -1·81, -1·40 | **<0·0001** | -1·45 | -1·67, -1·24 | **<0·0001** | -1·6 | -1·81, -1·39 | **<0·0001** |
| Age, months | -0·01 | -0·02, -0·00 | **0·020** | -0·01 | -0·02, -0·00 | **0·023** | -0·01 | -0·02, -0·00 | **0·024** |
| Sex, male | -0·31 | -0·42, -0·21 | **<0·0001** | -0·32 | -0·43, -0·22 | **<0·0001** | -0·31 | -0·41, -0·21 | **<0·0001** |
| Difference in slope early discharge [MW - NW] | -0·02 | -0·05, 0·01 | 0·262 | -0·02 | -0·05, 0·01 | 0·261 | -0·02 | -0·05, 0·01 | 0·261 |
| Difference in slope early discharge [SW - NW] | 0·01 | -0·02, 0·04 | 0·51 | 0·01 | -0·02, 0·04 | 0·51 | 0·01 | -0·02, 0·04 | 0·512 |
| Difference in slope early discharge [NO - NW] | 0·07 | 0·03, 0·11 | **0·0011** | 0·07 | 0·03, 0·11 | **0·0012** | 0·07 | 0·03, 0·11 | **0·0012** |
| Difference in change of slope between early and late discharge [MW - NW] | 0·03 | -0·00, 0·07 | 0·05 | 0·03 | -0·00, 0·07 | 0·05 | 0·03 | -0·00, 0·07 | 0·05 |
| Difference in change of slope between early and late discharge [SW - NW] | 0·03 | -0·01, 0·06 | 0·101 | 0·03 | -0·01, 0·06 | 0·101 | 0·03 | -0·01, 0·06 | 0·1 |
| Difference in change of slope between early and late discharge [NO - NW] | -0·04 | -0·08, 0·01 | 0·13 | -0·04 | -0·08, 0·01 | 0·131 | -0·04 | -0·08, 0·01 | 0·131 |
| Age-inappropriate nutrition [medium] | | |  | -0·05 | -0·23, 0·12 | 0·545 |  |  |  |
| Age-inappropriate nutrition [high] | | |  | -0·33 | -0·46, -0·20 | **<0·0001** |  |  |  |
| Caregiver Characteristics [Moderatly adverse] | | |  |  |  |  | -0·2 | -0·34, -0·07 | **0·0022** |
| Caregiver Characteristics [Most adverse] | | |  |  |  |  | -0·24 | -0·38, -0·11 | **0·00048** |
| **Random Effects** |  |  |  |  |  |  |  |  |  |
| σ^2^ | 0·07 | | | 0·07 | | | 0·07 | | |
| τ_00_ | 2·03 record_id | | | 2·00 record_id | | | 2·02 record_id | | |
|  | 0·10 site | | | 0·11 site | | | 0·11 site | | |
| τ_11_ | 0·04 record_id·time_months | | | 0·04 record_id·time_months | | | 0·04 record_id·time_months | | |
|  | 0·02 record_id·I(pmax(time_months - 1·5, 0)) | | | 0·02 record_id·I(pmax(time_months - 1·5, 0)) | | | 0·02 record_id·I(pmax(time_months - 1·5, 0)) | | |
| ρ_01_ | -0·41 record_id·time_months | | | -0·41 record_id·time_months | | | -0·41 record_id·time_months | | |
|  | 0·20 record_id·I(pmax(time_months - 1·5, 0)) | | | 0·19 record_id·I(pmax(time_months - 1·5, 0)) | | | 0·19 record_id·I(pmax(time_months - 1·5, 0)) | | |
| ICC | 0·96 | | | 0·96 | | | 0·96 | | |
| N | 9 _site_ | | | 9 _site_ | | | 9 _site_ | | |
|  | 2461 _record_id_ | | | 2461 _record_id_ | | | 2461 _record_id_ | | |
| Observations | 9425 | | | 9425 | | | 9425 | | |
| Marginal R^2^ / Conditional R^2^ | 0·196 / 0·972 | | | 0·204 / 0·972 | | | 0·201 / 0·972 | | |
| AIC | 17116·528 | | | 17103·563 | | | 17112·918 | | |
| AICc | 17116·636 | | | 17103·69 | | | 17113·046 | | |

Table presents results for both fixed and random terms of mixed piecewise models fit using *lme4* R package. A knot point was positioned at 45-days post-discharge which defines two discharge phases (i.e., early, before 45-days and, late, after 45-days). Time was coded in months (i.e., 1·5 months corresponds to 45-days; and 3 months to 90-days). Random structure included random slopes per participant and random intercepts for site with participants nested within. Models were fit with maximum likelihood for comparison with anova using Satterthwaite's approximation of degrees of freedom as implemented in the *lmerTest* R package (Kuznetsova, A. et al., 2017). Models were evaluated using fit metrics AIC, AICc, and BIC. Restricted maximum likelihood was used to fit final models. Groups: NW, no wasting; MW, moderate wasting; SW, severe wasting; NO, nutritional oedema. LAZ, length-for-age z-score.

### **Supplemental Table 24**. Change in LAZ in the 180-days post-discharge per nutritional group associated with exposure domains of household-level exposures and access to health care.

|  | **Base model** | | | **Household-level  exposures** | | | **Access to  health care** | | |
| --- | --- | --- | --- | --- | --- | --- | --- | --- | --- |
| *Predictors* | *Est.* | *95% CI* | *p* | *Est.* | *95% CI* | *p* | *Est.* | *95% CI* | *p* |
| Intercept [ref, NW] | -0·99 | -1·24, -0·73 | **<0·0001** | -0·82 | -1·11, -0·52 | **<0·0001** | -1·02 | -1·29, -0·75 | **<0·0001** |
| Slope early discharge [NW] | -0·13 | -0·15, -0·11 | **<0·0001** | -0·13 | -0·15, -0·11 | **<0·0001** | -0·13 | -0·15, -0·11 | **<0·0001** |
| Difference in slope early vs· late discharge [NW] | 0·11 | 0·09, 0·13 | **<0·0001** | 0·11 | 0·09, 0·13 | **<0·0001** | 0·11 | 0·09, 0·13 | **<0·0001** |
| Difference in intercept early discharge [MW - NW] | -0·68 | -0·83, -0·52 | **<0·0001** | -0·66 | -0·81, -0·51 | **<0·0001** | -0·68 | -0·83, -0·53 | **<0·0001** |
| Difference in intercept early discharge [SW - NW] | -1·57 | -1·71, -1·42 | **<0·0001** | -1·55 | -1·70, -1·41 | **<0·0001** | -1·57 | -1·71, -1·42 | **<0·0001** |
| Difference in intercept early discharge [NO - NW] | -1·6 | -1·81, -1·40 | **<0·0001** | -1·55 | -1·76, -1·34 | **<0·0001** | -1·61 | -1·81, -1·40 | **<0·0001** |
| Age, months | -0·01 | -0·02, -0·00 | **0·020** | -0·01 | -0·02, -0·00 | **0·025** | -0·01 | -0·02, -0·00 | **0·018** |
| Sex, male | -0·31 | -0·42, -0·21 | **<0·0001** | -0·31 | -0·41, -0·21 | **<0·0001** | -0·31 | -0·41, -0·21 | **<0·0001** |
| Difference in slope early discharge [MW - NW] | -0·02 | -0·05, 0·01 | 0·262 | -0·02 | -0·05, 0·01 | 0·261 | -0·02 | -0·05, 0·01 | 0·261 |
| Difference in slope early discharge [SW - NW] | 0·01 | -0·02, 0·04 | 0·51 | 0·01 | -0·02, 0·04 | 0·511 | 0·01 | -0·02, 0·04 | 0·511 |
| Difference in slope early discharge [NO - NW] | 0·07 | 0·03, 0·11 | **0·0011** | 0·07 | 0·03, 0·11 | **0·0012** | 0·07 | 0·03, 0·11 | **0·0012** |
| Difference in change of slope between early and late discharge [MW - NW] | 0·03 | -0·00, 0·07 | 0·05 | 0·03 | -0·00, 0·07 | 0·05 | 0·03 | -0·00, 0·07 | 0·05 |
| Difference in change of slope between early and late discharge [SW - NW] | 0·03 | -0·01, 0·06 | 0·101 | 0·03 | -0·01, 0·06 | 0·1 | 0·03 | -0·01, 0·06 | 0·1 |
| Difference in change of slope between early and late discharge [NO - NW] | -0·04 | -0·08, 0·01 | 0·13 | -0·04 | -0·08, 0·01 | 0·131 | -0·04 | -0·08, 0·01 | 0·13 |
| Household-level exposures [Moderatly adverse] | | |  | -0·23 | -0·36, -0·09 | **0·00088** |  |  |  |
| Household-level exposures [Most adverse] | | |  | -0·33 | -0·49, -0·18 | **<0·0001** |  |  |  |
| Access to health care [Moderatly adverse] | | |  |  |  |  | 0·12 | -0·01, 0·24 | 0·073 |
| Access to health care [Least adverse] | | |  |  |  |  | -0·01 | -0·15, 0·14 | 0·937 |
| **Random Effects** |  |  |  |  |  |  |  |  |  |
| σ^2^ | 0·07 | | | 0·07 | | | 0·07 | | |
| τ_00_ | 2·03 record_id | | | 2·02 record_id | | | 2·03 record_id | | |
|  | 0·10 site | | | 0·14 site | | | 0·10 site | | |
| τ_11_ | 0·04 record_id·time_months | | | 0·04 record_id·time_months | | | 0·04 record_id·time_months | | |
|  | 0·02 record_id·I(pmax(time_months - 1·5, 0)) | | | 0·02 record_id·I(pmax(time_months - 1·5, 0)) | | | 0·02 record_id·I(pmax(time_months - 1·5, 0)) | | |
| ρ_01_ | -0·41 record_id·time_months | | | -0·42 record_id·time_months | | | -0·41 record_id·time_months | | |
|  | 0·20 record_id·I(pmax(time_months - 1·5, 0)) | | | 0·19 record_id·I(pmax(time_months - 1·5, 0)) | | | 0·19 record_id·I(pmax(time_months - 1·5, 0)) | | |
| ICC | 0·96 | | | 0·97 | | | 0·96 | | |
| N | 9 _site_ | | | 9 _site_ | | | 9 _site_ | | |
|  | 2461 _record_id_ | | | 2461 _record_id_ | | | 2461 _record_id_ | | |
| Observations | 9425 | | | 9425 | | | 9425 | | |
| Marginal R^2^ / Conditional R^2^ | 0·196 / 0·972 | | | 0·200 / 0·972 | | | 0·197 / 0·972 | | |
| AIC | 17116·528 | | | 17108·497 | | | 17123·183 | | |
| AICc | 17116·636 | | | 17108·625 | | | 17123·31 | | |

Table presents results for both fixed and random terms of mixed piecewise models fit using *lme4* R package. A knot point was positioned at 45-days post-discharge which defines two discharge phases (i.e., early, before 45-days and, late, after 45-days). Time was coded in months (i.e., 1·5 months corresponds to 45-days; and 3 months to 90-days). Random structure included random slopes per participant and random intercepts for site with participants nested within. Models were fit with maximum likelihood for comparison with anova using Satterthwaite's approximation of degrees of freedom as implemented in the *lmerTest* R package (Kuznetsova, A. et al., 2017). Models were evaluated using fit metrics AIC, AICc, and BIC. Restricted maximum likelihood was used to fit final models. Groups: NW, no wasting; MW, moderate wasting; SW, severe wasting; NO, nutritional oedema. LAZ, length-for-age z-score.

### **Supplemental Table 25**. Change in LAZ in the 180-days post-discharge per nutritional group associated with HIV and small birth size.

|  | **Base model** | | | **HIV status** | | | **Birth size** | | |
| --- | --- | --- | --- | --- | --- | --- | --- | --- | --- |
| *Predictors* | *Est.* | *95% CI* | *p* | *Est.* | *95% CI* | *p* | *Est.* | *95% CI* | *p* |
| Intercept [ref, NW] | -0·99 | -1·24, -0·73 | **<0·0001** | -0·96 | -1·21, -0·71 | **<0·0001** | -0·84 | -1·10, -0·59 | **<0·0001** |
| Slope early discharge [NW] | -0·13 | -0·15, -0·11 | **<0·0001** | -0·13 | -0·15, -0·11 | **<0·0001** | -0·13 | -0·15, -0·11 | **<0·0001** |
| Difference in slope early vs· late discharge [NW] | 0·11 | 0·09, 0·13 | **<0·0001** | 0·11 | 0·09, 0·13 | **<0·0001** | 0·11 | 0·09, 0·13 | **<0·0001** |
| Difference in intercept early discharge [MW - NW] | -0·68 | -0·83, -0·52 | **<0·0001** | -0·66 | -0·82, -0·51 | **<0·0001** | -0·63 | -0·78, -0·49 | **<0·0001** |
| Difference in intercept early discharge [SW - NW] | -1·57 | -1·71, -1·42 | **<0·0001** | -1·54 | -1·69, -1·40 | **<0·0001** | -1·51 | -1·65, -1·37 | **<0·0001** |
| Difference in intercept early discharge [NO - NW] | -1·6 | -1·81, -1·40 | **<0·0001** | -1·58 | -1·79, -1·38 | **<0·0001** | -1·59 | -1·79, -1·39 | **<0·0001** |
| Age, months | -0·01 | -0·02, -0·00 | **0·020** | -0·01 | -0·02, -0·00 | **0·018** | -0·01 | -0·02, -0·00 | **0·0024** |
| Sex, male | -0·31 | -0·42, -0·21 | **<0·0001** | -0·31 | -0·41, -0·21 | **<0·0001** | -0·33 | -0·43, -0·23 | **<0·0001** |
| Difference in slope early discharge [MW - NW] | -0·02 | -0·05, 0·01 | 0·262 | -0·02 | -0·05, 0·01 | 0·262 | -0·02 | -0·05, 0·01 | 0·256 |
| Difference in slope early discharge [SW - NW] | 0·01 | -0·02, 0·04 | 0·51 | 0·01 | -0·02, 0·04 | 0·509 | 0·01 | -0·02, 0·04 | 0·514 |
| Difference in slope early discharge [NO - NW] | 0·07 | 0·03, 0·11 | **0·0011** | 0·07 | 0·03, 0·11 | **0·0011** | 0·07 | 0·03, 0·11 | **0·0011** |
| Difference in change of slope between early and late discharge [MW - NW] | 0·03 | -0·00, 0·07 | 0·05 | 0·03 | -0·00, 0·07 | 0·051 | 0·04 | 0·00, 0·07 | **0·048** |
| Difference in change of slope between early and late discharge [SW - NW] | 0·03 | -0·01, 0·06 | 0·101 | 0·03 | -0·01, 0·06 | 0·101 | 0·03 | -0·01, 0·06 | 0·099 |
| Difference in change of slope between early and late discharge [NO - NW] | -0·04 | -0·08, 0·01 | 0·13 | -0·04 | -0·08, 0·01 | 0·13 | -0·04 | -0·08, 0·01 | 0·125 |
| HIV exposed |  |  |  | -0·25 | -0·46, -0·03 | 0·025 |  |  |  |
| HIV infected |  |  |  | -0·4 | -0·69, -0·11 | **0·0067** |  |  |  |
| Small birth size |  |  |  |  |  |  | -0·75 | -0·88, -0·62 | **<0·0001** |
|  |  |  |  |  |  |  |  |  |  |
| **Random Effects** |  |  |  |  |  |  |  |  |  |
| σ^2^ | 0·07 | | | 0·07 | | | 0·07 | | |
| τ_00_ | 2·03 record_id | | | 2·02 record_id | | | 1·89 record_id | | |
|  | 0·10 site | | | 0·10 site | | | 0·10 site | | |
| τ_11_ | 0·04 record_id·time_months | | | 0·04 record_id·time_months | | | 0·04 record_id·time_months | | |
|  | 0·02 record_id·I(pmax(time_months - 1·5, 0)) | | | 0·02 record_id·I(pmax(time_months - 1·5, 0)) | | | 0·02 record_id·I(pmax(time_months - 1·5, 0)) | | |
| ρ_01_ | -0·41 record_id·time_months | | | -0·41 record_id·time_months | | | -0·39 record_id·time_months | | |
|  | 0·20 record_id·I(pmax(time_months - 1·5, 0)) | | | 0·20 record_id·I(pmax(time_months - 1·5, 0)) | | | 0·18 record_id·I(pmax(time_months - 1·5, 0)) | | |
| ICC | 0·96 | | | 0·96 | | | 0·96 | | |
| N | 9 _site_ | | | 9 _site_ | | | 9 _site_ | | |
|  | 2461 _record_id_ | | | 2461 _record_id_ | | | 2461 _record_id_ | | |
| Observations | 9425 | | | 9425 | | | 9425 | | |
| Marginal R^2^ / Conditional R^2^ | 0·196 / 0·972 | | | 0·199 / 0·972 | | | 0·232 / 0·972 | | |
| AIC | 17116·528 | | | 17114·113 | | | 17006·766 | | |
| AICc | 17116·636 | | | 17114·24 | | | 17006·883 | | |

Table presents results for both fixed and random terms of mixed piecewise models fit using *lme4* R package. A knot point was positioned at 45-days post-discharge which defines two discharge phases (i.e., early, before 45-days and, late, after 45-days). Time was coded in months (i.e., 1·5 months corresponds to 45-days; and 3 months to 90-days). Random structure included random slopes per participant and random intercepts for site with participants nested within. Models were fit with maximum likelihood for comparison with anova using Satterthwaite's approximation of degrees of freedom as implemented in the *lmerTest* R package (Kuznetsova, A. et al., 2017). Models were evaluated using fit metrics AIC, AICc, and BIC. Restricted maximum likelihood was used to fit final models. Groups: NW, no wasting; MW, moderate wasting; SW, severe wasting; NO, nutritional oedema. LAZ, length-for-age z-score.

### **Supplemental Table 26**. Change in LAZ in the 180-days post-discharge per nutritional group associated with chronic medical conditions and prior hospitalisation.

|  | **Base model** | | | **Chronic medical conditions** | | | **Prior hospitalisation** | | |
| --- | --- | --- | --- | --- | --- | --- | --- | --- | --- |
| *Predictors* | *Est.* | *95% CI* | *p* | *Est.* | *95% CI* | *p* | *Est.* | *95% CI* | *p* |
| Intercept [ref, NW] | -0·99 | -1·24, -0·73 | **<0·0001** | -0·98 | -1·23, -0·73 | **<0·0001** | -0·97 | -1·23, -0·72 | **<0·0001** |
| Slope early discharge [NW] | -0·13 | -0·15, -0·11 | **<0·0001** | -0·13 | -0·15, -0·11 | **<0·0001** | -0·13 | -0·15, -0·11 | **<0·0001** |
| Difference in slope early vs· late discharge [NW] | 0·11 | 0·09, 0·13 | **<0·0001** | 0·11 | 0·09, 0·13 | **<0·0001** | 0·11 | 0·09, 0·13 | **<0·0001** |
| Difference in intercept early discharge [MW - NW] | -0·68 | -0·83, -0·52 | **<0·0001** | -0·67 | -0·82, -0·52 | **<0·0001** | -0·67 | -0·83, -0·52 | **<0·0001** |
| Difference in intercept early discharge [SW - NW] | -1·57 | -1·71, -1·42 | **<0·0001** | -1·56 | -1·70, -1·41 | **<0·0001** | -1·56 | -1·71, -1·42 | **<0·0001** |
| Difference in intercept early discharge [NO - NW] | -1·6 | -1·81, -1·40 | **<0·0001** | -1·6 | -1·81, -1·39 | **<0·0001** | -1·6 | -1·81, -1·40 | **<0·0001** |
| Age, months | -0·01 | -0·02, -0·00 | **0·020** | -0·01 | -0·02, -0·00 | **0·024** | -0·01 | -0·02, -0·00 | **0·026** |
| Sex, male | -0·31 | -0·42, -0·21 | **<0·0001** | -0·31 | -0·41, -0·21 | **<0·0001** | -0·31 | -0·41, -0·21 | **<0·0001** |
| Difference in slope early discharge [MW - NW] | -0·02 | -0·05, 0·01 | 0·262 | -0·02 | -0·05, 0·01 | 0·262 | -0·02 | -0·05, 0·01 | 0·261 |
| Difference in slope early discharge [SW - NW] | 0·01 | -0·02, 0·04 | 0·51 | 0·01 | -0·02, 0·04 | 0·51 | 0·01 | -0·02, 0·04 | 0·511 |
| Difference in slope early discharge [NO - NW] | 0·07 | 0·03, 0·11 | **0·0011** | 0·07 | 0·03, 0·11 | **0·0011** | 0·07 | 0·03, 0·11 | **0·0011** |
| Difference in change of slope between early and late discharge [MW - NW] | 0·03 | -0·00, 0·07 | 0·05 | 0·03 | -0·00, 0·07 | 0·05 | 0·03 | -0·00, 0·07 | 0·05 |
| Difference in change of slope between early and late discharge [SW - NW] | 0·03 | -0·01, 0·06 | 0·101 | 0·03 | -0·01, 0·06 | 0·101 | 0·03 | -0·01, 0·06 | 0·101 |
| Difference in change of slope between early and late discharge [NO - NW] | -0·04 | -0·08, 0·01 | 0·13 | -0·04 | -0·08, 0·01 | 0·129 | -0·04 | -0·08, 0·01 | 0·13 |
| Chronic medical condition |  |  |  | -0·23 | -0·44, -0·02 | **0·030** |  |  |  |
| Prior hospitalisation |  |  |  |  |  |  | -0·09 | -0·20, 0·03 | 0·15 |
| **Random Effects** |  |  |  |  |  |  |  |  |  |
| σ^2^ | 0·07 | | | 0·07 | | | 0·07 | | |
| τ_00_ | 2·03 record_id | | | 2·03 record_id | | | 2·02 record_id | | |
|  | 0·10 site | | | 0·09 site | | | 0·10 site | | |
| τ_11_ | 0·04 record_id·time_months | | | 0·04 record_id·time_months | | | 0·04 record_id·time_months | | |
|  | 0·02 record_id·I(pmax(time_months - 1·5, 0)) | | | 0·02 record_id·I(pmax(time_months - 1·5, 0)) | | | 0·02 record_id·I(pmax(time_months - 1·5, 0)) | | |
| ρ_01_ | -0·41 record_id·time_months | | | -0·41 record_id·time_months | | | -0·41 record_id·time_months | | |
|  | 0·20 record_id·I(pmax(time_months - 1·5, 0)) | | | 0·20 record_id·I(pmax(time_months - 1·5, 0)) | | | 0·19 record_id·I(pmax(time_months - 1·5, 0)) | | |
| ICC | 0·96 | | | 0·96 | | | 0·96 | | |
| N | 9 _site_ | | | 9 _site_ | | | 9 _site_ | | |
|  | 2461 _record_id_ | | | 2461 _record_id_ | | | 2461 _record_id_ | | |
| Observations | 9425 | | | 9425 | | | 9425 | | |
| Marginal R^2^ / Conditional R^2^ | 0·196 / 0·972 | | | 0·197 / 0·972 | | | 0·197 / 0·972 | | |
| AIC | 17116·528 | | | 17116·506 | | | 17120·26 | | |
| AICc | 17116·636 | | | 17116·623 | | | 17120·378 | | |

Table presents results for both fixed and random terms of mixed piecewise models fit using *lme4* R package. A knot point was positioned at 45-days post-discharge which defines two discharge phases (i.e., early, before 45-days and, late, after 45-days). Time was coded in months (i.e., 1·5 months corresponds to 45-days; and 3 months to 90-days). Random structure included random slopes per participant and random intercepts for site with participants nested within. Models were fit with maximum likelihood for comparison with anova using Satterthwaite's approximation of degrees of freedom as implemented in the *lmerTest* R package (Kuznetsova, A. et al., 2017). Models were evaluated using fit metrics AIC, AICc, and BIC. Restricted maximum likelihood was used to fit final models. Groups: NW, no wasting; MW, moderate wasting; SW, severe wasting; NO, nutritional oedema. LAZ, length-for-age z-score.

### **Supplemental Table 27**. Change in WAZ in the 180-days post-discharge per nutritional group associated with anaemia or diarrhoea diagnosed at admission.

|  | **Base model** | | | **Anaemia** | | | **Diarrhoea** | | |
| --- | --- | --- | --- | --- | --- | --- | --- | --- | --- |
| *Predictors* | *Est.* | *95% CI* | *p* | *Est.* | *95% CI* | *p* | *Est.* | *95% CI* | *p* |
| Intercept [ref, NW] | -1·1 | -1·27, -0·92 | **<0·0001** | -1 | -1·21, -0·80 | **<0·0001** | -1·15 | -1·33, -0·96 | **<0·0001** |
| Slope early discharge [NW] | 0·12 | 0·09, 0·15 | **<0·0001** | 0·12 | 0·09, 0·15 | **<0·0001** | 0·12 | 0·09, 0·15 | **<0·0001** |
| Difference in slope early vs· late discharge [NW] | -0·12 | -0·15, -0·09 | **<0·0001** | -0·12 | -0·15, -0·08 | **<0·0001** | -0·12 | -0·15, -0·08 | **<0·0001** |
| Difference in intercept early discharge [MW - NW] | -1·36 | -1·48, -1·24 | **<0·0001** | -1·34 | -1·47, -1·22 | **<0·0001** | -1·37 | -1·49, -1·25 | **<0·0001** |
| Difference in intercept early discharge [SW - NW] | -2·59 | -2·71, -2·48 | **<0·0001** | -2·59 | -2·70, -2·47 | **<0·0001** | -2·61 | -2·73, -2·49 | **<0·0001** |
| Difference in intercept early discharge [NO - NW] | -2·1 | -2·26, -1·93 | **<0·0001** | -2·08 | -2·25, -1·91 | **<0·0001** | -2·1 | -2·27, -1·93 | **<0·0001** |
| Age, months | 0·01 | -0·00, 0·02 | 0·057 | 0·01 | -0·00, 0·02 | 0·058 | 0·01 | -0·00, 0·02 | 0·052 |
| Sex, male | -0·25 | -0·33, -0·16 | **<0·0001** | -0·21 | -0·30, -0·13 | **<0·0001** | -0·25 | -0·33, -0·16 | **<0·0001** |
| Difference in slope early discharge [MW - NW] | 0·08 | 0·04, 0·13 | **0·0059** | 0·09 | 0·04, 0·13 | **0·0044** | 0·08 | 0·04, 0·13 | **0·0059** |
| Difference in slope early discharge [SW - NW] | 0·23 | 0·19, 0·28 | **<0·0001** | 0·23 | 0·19, 0·28 | **<0·0001** | 0·23 | 0·19, 0·28 | **<0·0001** |
| Difference in slope early discharge [NO - NW] | 0·32 | 0·26, 0·38 | **<0·0001** | 0·32 | 0·25, 0·38 | **<0·0001** | 0·32 | 0·26, 0·38 | **<0·0001** |
| Difference in change of slope between early and late discharge [MW - NW] | -0·03 | -0·08, 0·02 | 0·25 | -0·03 | -0·09, 0·02 | 0·22 | -0·03 | -0·08, 0·02 | 0·25 |
| Difference in change of slope between early and late discharge [SW - NW] | -0·14 | -0·19, -0·09 | **<0·0001** | -0·13 | -0·18, -0·08 | **<0·0001** | -0·14 | -0·19, -0·09 | **<0·0001** |
| Difference in change of slope between early and late discharge [NO - NW] | -0·2 | -0·27, -0·13 | **<0·0001** | -0·19 | -0·26, -0·12 | **<0·0001** | -0·2 | -0·27, -0·13 | **<0·0001** |
| Diarrhoea |  |  |  |  |  |  | 0·11 | 0·01, 0·20 | 0·026 |
| Anemia [Mild] |  |  |  | -0·03 | -0·16, 0·10 | 0·68 |  |  |  |
| Anemia [Moderate / Severe] |  |  |  | -0·19 | -0·31, -0·08 | **0·00095** |  |  |  |
| **Random Effects** |  |  |  |  |  |  |  |  |  |
| σ^2^ | 0·1 | | | 0·1 | | | 0·1 | | |
| τ_00_ | 1·24 record_id | | | 1·23 record_id | | | 1·25 record_id | | |
|  | 0·03 site | | | 0·04 site | | | 0·04 site | | |
| τ_11_ | 0·12 record_id·time_months | | | 0·12 record_id·time_months | | | 0·12 record_id·time_months | | |
|  | 0·12 record_id·I(pmax(time_months - 1·5, 0)) | | | 0·12 record_id·I(pmax(time_months - 1·5, 0)) | | | 0·12 record_id·I(pmax(time_months - 1·5, 0)) | | |
| ρ_01_ | -0·19 record_id·time_months | | | -0·19 record_id·time_months | | | -0·19 record_id·time_months | | |
|  | 0·05 record_id·I(pmax(time_months - 1·5, 0)) | | | 0·05 record_id·I(pmax(time_months - 1·5, 0)) | | | 0·05 record_id·I(pmax(time_months - 1·5, 0)) | | |
| ICC | 0·93 | | | 0·93 | | | 0·93 | | |
| N | 9 _site_ | | | 9 _site_ | | | 9 _site_ | | |
|  | 2461 _record_id_ | | | 2357 _record_id_ | | | 2461 _record_id_ | | |
| Observations | 9448 | | | 9059 | | | 9448 | | |
| Marginal R^2^ / Conditional R^2^ | 0·392 / 0·957 | | | 0·395 / 0·957 | | | 0·391 / 0·957 | | |
| AIC | 19680·404 | | | 18839·201 | | | 19681·737 | | |

Table presents results for both fixed and random terms of mixed piecewise models fit using *lme4* R package. A knot point was positioned at 45-days post-discharge which defines two discharge phases (i.e., early, before 45-days and, late, after 45-days). Time was coded in months (i.e., 1·5 months corresponds to 45-days; and 3 months to 90-days). Random structure included random slopes per participant and random intercepts for site with participants nested within. Models were fit with maximum likelihood for comparison with anova using Satterthwaite's approximation of degrees of freedom as implemented in the *lmerTest* R package (Kuznetsova, A. et al., 2017). Models were evaluated using fit metrics AIC, AICc, and BIC. Restricted maximum likelihood was used to fit final models. Groups: NW, no wasting; MW, moderate wasting; SW, severe wasting; NO, nutritional oedema. WAZ, weight-for-age z-score.

### **Supplemental Table 28**. Change in WAZ in the 180-days post-discharge per nutritional group associated with sepsis or pneumonia diagnosed at admission.

| WAZ |  |  |  |  |  |  |  |  |  |
| --- | --- | --- | --- | --- | --- | --- | --- | --- | --- |
|  | **Base model** | | | **Sepsis** | | | **Pneumonia** | | |
| *Predictors* | *Est.* | *95% CI* | *p* | *Est.* | *95% CI* | *p* | *Est.* | *95% CI* | *p* |
| Intercept [ref, NW] | -1·1 | -1·27, -0·92 | **<0·0001** | -1·09 | -1·26, -0·91 | **<0·0001** | -1·09 | -1·26, -0·91 | **<0·0001** |
| Slope early discharge [NW] | 0·12 | 0·09, 0·15 | **<0·0001** | 0·12 | 0·09, 0·15 | **<0·0001** | 0·12 | 0·09, 0·15 | **<0·0001** |
| Difference in slope early vs· late discharge [NW] | -0·12 | -0·15, -0·09 | **<0·0001** | -0·12 | -0·15, -0·09 | **<0·0001** | -0·12 | -0·15, -0·09 | **<0·0001** |
| Difference in intercept early discharge [MW - NW] | -1·36 | -1·48, -1·24 | **<0·0001** | -1·36 | -1·48, -1·24 | **<0·0001** | -1·36 | -1·48, -1·24 | **<0·0001** |
| Difference in intercept early discharge [SW - NW] | -2·59 | -2·71, -2·48 | **<0·0001** | -2·59 | -2·71, -2·48 | **<0·0001** | -2·6 | -2·71, -2·48 | **<0·0001** |
| Difference in intercept early discharge [NO - NW] | -2·1 | -2·26, -1·93 | **<0·0001** | -2·1 | -2·26, -1·93 | **<0·0001** | -2·1 | -2·27, -1·93 | **<0·0001** |
| Age, months | 0·01 | -0·00, 0·02 | 0·057 | 0·01 | -0·00, 0·02 | 0·056 | 0·01 | -0·00, 0·02 | 0·061 |
| Sex, male | -0·25 | -0·33, -0·16 | **<0·0001** | -0·25 | -0·33, -0·16 | **<0·0001** | -0·25 | -0·33, -0·16 | **<0·0001** |
| Difference in slope early discharge [MW - NW] | 0·08 | 0·04, 0·13 | **0·0059** | 0·08 | 0·04, 0·13 | **0·0059** | 0·08 | 0·04, 0·13 | **0·0059** |
| Difference in slope early discharge [SW - NW] | 0·23 | 0·19, 0·28 | **<0·0001** | 0·23 | 0·19, 0·28 | **<0·0001** | 0·23 | 0·19, 0·28 | **<0·0001** |
| Difference in slope early discharge [NO - NW] | 0·32 | 0·26, 0·38 | **<0·0001** | 0·32 | 0·26, 0·38 | **<0·0001** | 0·32 | 0·26, 0·38 | **<0·0001** |
| Difference in change of slope between early and late discharge [MW - NW] | -0·03 | -0·08, 0·02 | 0·25 | -0·03 | -0·08, 0·02 | 0·25 | -0·03 | -0·08, 0·02 | 0·25 |
| Difference in change of slope between early and late discharge [SW - NW] | -0·14 | -0·19, -0·09 | **<0·0001** | -0·14 | -0·19, -0·09 | **<0·0001** | -0·14 | -0·19, -0·09 | **<0·0001** |
| Difference in change of slope between early and late discharge [NO - NW] | -0·2 | -0·27, -0·13 | **<0·0001** | -0·2 | -0·27, -0·13 | **<0·0001** | -0·2 | -0·27, -0·13 | **<0·0001** |
| Sepsis |  |  |  | -0·06 | -0·20, 0·07 | 0·37 |  |  |  |
| Pneumonia |  |  |  |  |  |  | -0·02 | -0·12, 0·07 | 0·64 |
| **Random Effects** |  |  |  |  |  |  |  |  |  |
| σ^2^ | 0·1 | | | 0·1 | | | 0·1 | | |
| τ_00_ | 1·24 record_id | | | 1·24 record_id | | | 1·25 record_id | | |
|  | 0·03 site | | | 0·03 site | | | 0·03 site | | |
| τ_11_ | 0·12 record_id·time_months | | | 0·12 record_id·time_months | | | 0·12 record_id·time_months | | |
|  | 0·12 record_id·I(pmax(time_months - 1·5, 0)) | | | 0·12 record_id·I(pmax(time_months - 1·5, 0)) | | | 0·12 record_id·I(pmax(time_months - 1·5, 0)) | | |
| ρ_01_ | -0·19 record_id·time_months | | | -0·19 record_id·time_months | | | -0·19 record_id·time_months | | |
|  | 0·05 record_id·I(pmax(time_months - 1·5, 0)) | | | 0·05 record_id·I(pmax(time_months - 1·5, 0)) | | | 0·05 record_id·I(pmax(time_months - 1·5, 0)) | | |
| ICC | 0·93 | | | 0·93 | | | 0·93 | | |
| N | 9 _site_ | | | 9 _site_ | | | 9 _site_ | | |
|  | 2461 _record_id_ | | | 2461 _record_id_ | | | 2461 _record_id_ | | |
| Observations | 9448 | | | 9448 | | | 9448 | | |
| Marginal R^2^ / Conditional R^2^ | 0·392 / 0·957 | | | 0·392 / 0·957 | | | 0·392 / 0·957 | | |
| AIC | 19680·404 | | | 19685·097 | | | 19686·352 | | |

Table presents results for both fixed and random terms of mixed piecewise models fit using *lme4* R package. A knot point was positioned at 45-days post-discharge which defines two discharge phases (i.e., early, before 45-days and, late, after 45-days). Time was coded in months (i.e., 1·5 months corresponds to 45-days; and 3 months to 90-days). Random structure included random slopes per participant and random intercepts for site with participants nested within. Models were fit with maximum likelihood for comparison with anova using Satterthwaite's approximation of degrees of freedom as implemented in the *lmerTest* R package (Kuznetsova, A. et al., 2017). Models were evaluated using fit metrics AIC, AICc, and BIC. Restricted maximum likelihood was used to fit final models. Groups: NW, no wasting; MW, moderate wasting; SW, severe wasting; NO, nutritional oedema. WAZ, weight-for-age z-score.

### **Supplemental Table 29**. Change in WAZ in the 180-days post-discharge per nutritional group associated with exposure domains of illness severity at admission and at discharge.

|  | **Base model** | | | **Illness severity  at admission** | | | **Illness severity  at discharge** | | |
| --- | --- | --- | --- | --- | --- | --- | --- | --- | --- |
| *Predictors* | *Est.* | *95% CI* | *p* | *Est.* | *95% CI* | *p* | *Est.* | *95% CI* | *p* |
| Intercept [ref, NW] | -1·10 | -1·27, -0·92 | **<0·0001** | -1·17 | -1·36, -0·98 | **<0·0001** | -1·08 | -1·25, -0·90 | **<0·0001** |
| Slope early discharge [NW] | 0·12 | 0·09, 0·15 | **<0·0001** | 0·12 | 0·09, 0·15 | **<0·0001** | 0·12 | 0·09, 0·15 | **<0·0001** |
| Difference in slope early vs· late discharge [NW] | -0·12 | -0·15, -0·09 | **<0·0001** | -0·12 | -0·15, -0·09 | **<0·0001** | -0·12 | -0·15, -0·09 | **<0·0001** |
| Difference in intercept early discharge [MW - NW] | -1·36 | -1·48, -1·24 | **<0·0001** | -1·35 | -1·48, -1·23 | **<0·0001** | -1·36 | -1·48, -1·24 | **<0·0001** |
| Difference in intercept early discharge [SW - NW] | -2·59 | -2·71, -2·48 | **<0·0001** | -2·59 | -2·70, -2·47 | **<0·0001** | -2·6 | -2·71, -2·48 | **<0·0001** |
| Difference in intercept early discharge [NO - NW] | -2·1 | -2·26, -1·93 | **<0·0001** | -2·07 | -2·24, -1·90 | **<0·0001** | -2·1 | -2·27, -1·93 | **<0·0001** |
| Age, months | 0·01 | -0·00, 0·02 | 0·057 | 0·01 | -0·00, 0·02 | 0·06 | 0·01 | -0·00, 0·02 | 0·052 |
| Sex, male | -0·25 | -0·33, -0·16 | **<0·0001** | -0·24 | -0·33, -0·16 | **<0·0001** | -0·25 | -0·33, -0·16 | **<0·0001** |
| Difference in slope early discharge [MW - NW] | 0·08 | 0·04, 0·13 | **0·00059** | 0·08 | 0·04, 0·13 | **0·00059** | 0·08 | 0·04, 0·13 | **0·00059** |
| Difference in slope early discharge [SW - NW] | 0·23 | 0·19, 0·28 | **<0·0001** | 0·23 | 0·19, 0·28 | **<0·0001** | 0·23 | 0·19, 0·28 | **<0·0001** |
| Difference in slope early discharge [NO - NW] | 0·32 | 0·26, 0·38 | **<0·0001** | 0·32 | 0·26, 0·38 | **<0·0001** | 0·32 | 0·26, 0·38 | **<0·0001** |
| Difference in change of slope between early and late discharge [MW - NW] | -0·03 | -0·08, 0·02 | 0·252 | -0·03 | -0·08, 0·02 | 0·252 | -0·03 | -0·08, 0·02 | 0·253 |
| Difference in change of slope between early and late discharge [SW - NW] | -0·14 | -0·19, -0·09 | **<0·0001** | -0·14 | -0·19, -0·09 | **<0·0001** | -0·14 | -0·19, -0·09 | **<0·0001** |
| Difference in change of slope between early and late discharge [NO - NW] | -0·2 | -0·27, -0·13 | **<0·0001** | -0·20 | -0·27, -0·13 | **<0·0001** | -0·2 | -0·27, -0·13 | **<0·0001** |
| Illness severity at admission [medium] |  |  |  | 0·15 | 0·04, 0·25 | **0·0050** |  |  |  |
| Illness severity at admission [high] |  |  |  | 0·07 | -0·04, 0·18 | 0·239 |  |  |  |
| Illness severity at discharge [medium or high] |  |  |  |  |  |  | -0·1 | -0·22, 0·02 | 0·096 |
|  |  |  |  |  |  |  |  |  |  |
| **Random Effects** |  |  |  |  |  |  |  |  |  |
| σ^2^ | 0·1 | | | 0·1 | | | 0·1 | | |
| τ_00_ | 1·24 record_id | | | 1·24 record_id | | | 1·24 record_id | | |
|  | 0·03 site | | | 0·03 site | | | 0·03 site | | |
| τ_11_ | 0·12 record_id·time_months | | | 0·12 record_id·time_months | | | 0·12 record_id·time_months | | |
|  | 0·12 record_id·I(pmax(time_months - 1·5, 0)) | | | 0·12 record_id·I(pmax(time_months - 1·5, 0)) | | | 0·12 record_id·I(pmax(time_months - 1·5, 0)) | | |
| ρ_01_ | -0·19 record_id·time_months | | | -0·19 record_id·time_months | | | -0·19 record_id·time_months | | |
|  | 0·05 record_id·I(pmax(time_months - 1·5, 0)) | | | 0·05 record_id·I(pmax(time_months - 1·5, 0)) | | | 0·05 record_id·I(pmax(time_months - 1·5, 0)) | | |
| ICC | 0·93 | | | 0·93 | | | 0·93 | | |
| N | 9 _site_ | | | 9 _site_ | | | 9 _site_ | | |
|  | 2461 _record_id_ | | | 2461 _record_id_ | | | 2461 _record_id_ | | |
| Observations | 9448 | | | 9448 | | | 9448 | | |
| Marginal R^2^ / Conditional R^2^ | 0·392 / 0·957 | | | 0·394 / 0·957 | | | 0·392 / 0·957 | | |
| AIC | 19680·404 | | | 19684·713 | | | 19683·362 | | |
| AICc | 19680·512 | | | 19684·84 | | | 19683·479 | | |

Table presents results for both fixed and random terms of mixed piecewise models fit using *lme4* R package. A knot point was positioned at 45-days post-discharge which defines two discharge phases (i.e., early, before 45-days and, late, after 45-days). Time was coded in months (i.e., 1·5 months corresponds to 45-days; and 3 months to 90-days). Random structure included random slopes per participant and random intercepts for site with participants nested within. Models were fit with maximum likelihood for comparison with anova using Satterthwaite's approximation of degrees of freedom as implemented in the *lmerTest* R package (Kuznetsova, A. et al., 2017). Models were evaluated using fit metrics AIC, AICc, and BIC. Restricted maximum likelihood was used to fit final models. Groups: NW, no wasting; MW, moderate wasting; SW, severe wasting; NO, nutritional oedema. WAZ, weight-for-age z-score.

### **Supplemental Table 30**. Change in WAZ in the 180-days post-discharge per nutritional group associated with exposure domains of age-inappropriate nutrition and caregiver characteristics.

|  | **Base model** | | | **Age-inappropriate nutrition** | | | **Caregiver  characteristics** | | |
| --- | --- | --- | --- | --- | --- | --- | --- | --- | --- |
| *Predictors* | *Est.* | *95% CI* | *p* | *Est.* | *95% CI* | *p* | *Est.* | *95% CI* | *p* |
| Intercept [ref, NW] | -1·10 | -1·27, -0·92 | **<0·0001** | -1·04 | -1·23, -0·85 | **<0·0001** | -1·02 | -1·22, -0·83 | **<0·0001** |
| Slope early discharge [NW] | 0·12 | 0·09, 0·15 | **<0·0001** | 0·12 | 0·09, 0·15 | **<0·0001** | 0·12 | 0·09, 0·15 | **<0·0001** |
| Difference in slope early vs· late discharge [NW] | -0·12 | -0·15, -0·09 | **<0·0001** | -0·12 | -0·15, -0·09 | **<0·0001** | -0·12 | -0·15, -0·09 | **<0·0001** |
| Difference in intercept early discharge [MW - NW] | -1·36 | -1·48, -1·24 | **<0·0001** | -1·28 | -1·40, -1·16 | **<0·0001** | -1·36 | -1·48, -1·24 | **<0·0001** |
| Difference in intercept early discharge [SW - NW] | -2·59 | -2·71, -2·48 | **<0·0001** | -2·41 | -2·53, -2·29 | **<0·0001** | -2·59 | -2·70, -2·47 | **<0·0001** |
| Difference in intercept early discharge [NO - NW] | -2·1 | -2·26, -1·93 | **<0·0001** | -1·9 | -2·08, -1·73 | **<0·0001** | -2·09 | -2·26, -1·93 | **<0·0001** |
| Age, months | 0·01 | -0·00, 0·02 | 0·057 | 0·01 | 0·00, 0·02 | 0·038 | 0·01 | 0·00, 0·02 | 0·048 |
| Sex, male | -0·25 | -0·33, -0·16 | **<0·0001** | -0·26 | -0·35, -0·18 | **<0·0001** | -0·24 | -0·33, -0·16 | **<0·0001** |
| Difference in slope early discharge [MW - NW] | 0·08 | 0·04, 0·13 | **0·00059** | 0·08 | 0·04, 0·13 | **0·00059** | 0·08 | 0·04, 0·13 | **0·00059** |
| Difference in slope early discharge [SW - NW] | 0·23 | 0·19, 0·28 | **<0·0001** | 0·23 | 0·19, 0·28 | **<0·0001** | 0·23 | 0·19, 0·28 | **<0·0001** |
| Difference in slope early discharge [NO - NW] | 0·32 | 0·26, 0·38 | **<0·0001** | 0·32 | 0·26, 0·38 | **<0·0001** | 0·32 | 0·26, 0·38 | **<0·0001** |
| Difference in change of slope between early and late discharge [MW - NW] | -0·03 | -0·08, 0·02 | 0·252 | -0·03 | -0·08, 0·02 | 0·253 | -0·03 | -0·08, 0·02 | 0·252 |
| Difference in change of slope between early and late discharge [SW - NW] | -0·14 | -0·19, -0·09 | **<0·0001** | -0·14 | -0·19, -0·09 | **<0·0001** | -0·14 | -0·19, -0·09 | **<0·0001** |
| Difference in change of slope between early and late discharge [NO - NW] | -0·2 | -0·27, -0·13 | **<0·0001** | -0·2 | -0·27, -0·13 | **<0·0001** | -0·2 | -0·27, -0·13 | **<0·0001** |
| Age-inappropriate nutrition [medium] |  |  |  | -0·18 | -0·32, -0·03 | **0·015** |  |  |  |
| Age-inappropriate nutrition [high] |  |  |  | -0·43 | -0·54, -0·32 | **<0·0001** |  |  |  |
| Caregiver Characteristics [Moderatly adverse] |  |  |  |  |  |  | -0·1 | -0·21, 0·01 | 0·079 |
| Caregiver Characteristics [Most adverse] |  |  |  |  |  |  | -0·17 | -0·29, -0·06 | **0·0027** |
| **Random Effects** |  |  |  |  |  |  |  |  |  |
| σ^2^ | 0·1 | | | 0·1 | | | 0·1 | | |
| τ_00_ | 1·24 record_id | | | 1·20 record_id | | | 1·24 record_id | | |
|  | 0·03 site | | | 0·05 site | | | 0·05 site | | |
| τ_11_ | 0·12 record_id·time_months | | | 0·12 record_id·time_months | | | 0·12 record_id·time_months | | |
|  | 0·12 record_id·I(pmax(time_months - 1·5, 0)) | | | 0·12 record_id·I(pmax(time_months - 1·5, 0)) | | | 0·12 record_id·I(pmax(time_months - 1·5, 0)) | | |
| ρ_01_ | -0·19 record_id·time_months | | | -0·18 record_id·time_months | | | -0·19 record_id·time_months | | |
|  | 0·05 record_id·I(pmax(time_months - 1·5, 0)) | | | 0·04 record_id·I(pmax(time_months - 1·5, 0)) | | | 0·05 record_id·I(pmax(time_months - 1·5, 0)) | | |
| ICC | 0·93 | | | 0·93 | | | 0·93 | | |
| N | 9 _site_ | | | 9 _site_ | | | 9 _site_ | | |
|  | 2461 _record_id_ | | | 2461 _record_id_ | | | 2461 _record_id_ | | |
| Observations | 9448 | | | 9448 | | | 9448 | | |
| Marginal R^2^ / Conditional R^2^ | 0·392 / 0·957 | | | 0·401 / 0·957 | | | 0·392 / 0·957 | | |
| AIC | 19680·404 | | | 19635·788 | | | 19683·468 | | |
| AICc | 19680·512 | | | 19635·915 | | | 19683·596 | | |

Table presents results for both fixed and random terms of mixed piecewise models fit using *lme4* R package. A knot point was positioned at 45-days post-discharge which defines two discharge phases (i.e., early, before 45-days and, late, after 45-days). Time was coded in months (i.e., 1·5 months corresponds to 45-days; and 3 months to 90-days). Random structure included random slopes per participant and random intercepts for site with participants nested within. Models were fit with maximum likelihood for comparison with anova using Satterthwaite's approximation of degrees of freedom as implemented in the *lmerTest* R package (Kuznetsova, A. et al., 2017). Models were evaluated using fit metrics AIC, AICc, and BIC. Restricted maximum likelihood was used to fit final models. Groups: NW, no wasting; MW, moderate wasting; SW, severe wasting; NO, nutritional oedema. WAZ, weight-for-age z-score.

### **Supplemental Table 31**. Change in WAZ in the 180-days post-discharge per nutritional group associated with exposure domains of household-level exposures and access to health care.

|  | **Base model** | | | **Household-level  exposures** | | | **Access to  health care** | | |
| --- | --- | --- | --- | --- | --- | --- | --- | --- | --- |
| *Predictors* | *Est.* | *95% CI* | *p* | *Est.* | *95% CI* | *p* | *Est.* | *95% CI* | *p* |
| Intercept [ref, NW] | -1·10 | -1·27, -0·92 | **<0·0001** | -1·04 | -1·24, -0·85 | **<0·0001** | -1·1 | -1·29, -0·92 | **<0·0001** |
| Slope early discharge [NW] | 0·12 | 0·09, 0·15 | **<0·0001** | 0·12 | 0·09, 0·15 | **<0·0001** | 0·12 | 0·09, 0·15 | **<0·0001** |
| Difference in slope early vs· late discharge [NW] | -0·12 | -0·15, -0·09 | **<0·0001** | -0·12 | -0·15, -0·09 | **<0·0001** | -0·12 | -0·15, -0·09 | **<0·0001** |
| Difference in intercept early discharge [MW - NW] | -1·36 | -1·48, -1·24 | **<0·0001** | -1·35 | -1·47, -1·23 | **<0·0001** | -1·36 | -1·48, -1·24 | **<0·0001** |
| Difference in intercept early discharge [SW - NW] | -2·59 | -2·71, -2·48 | **<0·0001** | -2·59 | -2·70, -2·47 | **<0·0001** | -2·59 | -2·71, -2·48 | **<0·0001** |
| Difference in intercept early discharge [NO - NW] | -2·1 | -2·26, -1·93 | **<0·0001** | -2·08 | -2·25, -1·91 | **<0·0001** | -2·09 | -2·26, -1·93 | **<0·0001** |
| Age, months | 0·01 | -0·00, 0·02 | 0·057 | 0·01 | -0·00, 0·02 | 0·055 | 0·01 | -0·00, 0·02 | 0·061 |
| Sex, male | -0·25 | -0·33, -0·16 | **<0·0001** | -0·25 | -0·33, -0·16 | **<0·0001** | -0·25 | -0·33, -0·16 | **<0·0001** |
| Difference in slope early discharge [MW - NW] | 0·08 | 0·04, 0·13 | **0·00059** | 0·08 | 0·04, 0·13 | **0·00059** | 0·08 | 0·04, 0·13 | **0·00059** |
| Difference in slope early discharge [SW - NW] | 0·23 | 0·19, 0·28 | **<0·0001** | 0·23 | 0·19, 0·28 | **<0·0001** | 0·23 | 0·19, 0·28 | **<0·0001** |
| Difference in slope early discharge [NO - NW] | 0·32 | 0·26, 0·38 | **<0·0001** | 0·32 | 0·26, 0·38 | **<0·0001** | 0·32 | 0·26, 0·38 | **<0·0001** |
| Difference in change of slope between early and late discharge [MW - NW] | -0·03 | -0·08, 0·02 | 0·252 | -0·03 | -0·08, 0·02 | 0·252 | -0·03 | -0·08, 0·02 | 0·252 |
| Difference in change of slope between early and late discharge [SW - NW] | -0·14 | -0·19, -0·09 | **<0·0001** | -0·14 | -0·19, -0·09 | **<0·0001** | -0·14 | -0·19, -0·09 | **<0·0001** |
| Difference in change of slope between early and late discharge [NO - NW] | -0·2 | -0·27, -0·13 | **<0·0001** | -0·2 | -0·27, -0·13 | **<0·0001** | -0·2 | -0·27, -0·13 | **<0·0001** |
| Household-level exposures [Moderatly adverse] |  |  |  | -0·07 | -0·18, 0·04 | 0·201 |  |  |  |
| Household-level exposures [Most adverse] |  |  |  | -0·1 | -0·23, 0·03 | 0·121 |  |  |  |
| Access to health care [Moderatly adverse] |  |  |  |  |  |  | 0·04 | -0·07, 0·15 | 0·45 |
| Access to health care [Least adverse] |  |  |  |  |  |  | -0·02 | -0·13, 0·10 | 0·796 |
|  |  |  |  |  |  |  |  |  |  |
| **Random Effects** |  |  |  |  |  |  |  |  |  |
| σ^2^ | 0·1 | | | 0·1 | | | 0·1 | | |
| τ_00_ | 1·24 record_id | | | 1·24 record_id | | | 1·25 record_id | | |
|  | 0·03 site | | | 0·04 site | | | 0·03 site | | |
| τ_11_ | 0·12 record_id·time_months | | | 0·12 record_id·time_months | | | 0·12 record_id·time_months | | |
|  | 0·12 record_id·I(pmax(time_months - 1·5, 0)) | | | 0·12 record_id·I(pmax(time_months - 1·5, 0)) | | | 0·12 record_id·I(pmax(time_months - 1·5, 0)) | | |
| ρ_01_ | -0·19 record_id·time_months | | | -0·19 record_id·time_months | | | -0·19 record_id·time_months | | |
|  | 0·05 record_id·I(pmax(time_months - 1·5, 0)) | | | 0·05 record_id·I(pmax(time_months - 1·5, 0)) | | | 0·05 record_id·I(pmax(time_months - 1·5, 0)) | | |
| ICC | 0·93 | | | 0·93 | | | 0·93 | | |
| N | 9 _site_ | | | 9 _site_ | | | 9 _site_ | | |
|  | 2461 _record_id_ | | | 2461 _record_id_ | | | 2461 _record_id_ | | |
| Observations | 9448 | | | 9448 | | | 9448 | | |
| Marginal R^2^ / Conditional R^2^ | 0·392 / 0·957 | | | 0·390 / 0·957 | | | 0·392 / 0·957 | | |
| AIC | 19680·404 | | | 19689·814 | | | 19691·317 | | |
| AICc | 19680·512 | | | 19689·942 | | | 19691·444 | | |

Table presents results for both fixed and random terms of mixed piecewise models fit using *lme4* R package. A knot point was positioned at 45-days post-discharge which defines two discharge phases (i.e., early, before 45-days and, late, after 45-days). Time was coded in months (i.e., 1·5 months corresponds to 45-days; and 3 months to 90-days). Random structure included random slopes per participant and random intercepts for site with participants nested within. Models were fit with maximum likelihood for comparison with anova using Satterthwaite's approximation of degrees of freedom as implemented in the *lmerTest* R package (Kuznetsova, A. et al., 2017). Models were evaluated using fit metrics AIC, AICc, and BIC. Restricted maximum likelihood was used to fit final models. Groups: NW, no wasting; MW, moderate wasting; SW, severe wasting; NO, nutritional oedema. WAZ, weight-for-age z-score.

### **Supplemental Table 32**. Change in WAZ in the 180-days post-discharge per nutritional group associated with HIV and small birth size.

|  | **Base model** | | | **HIV status** | | | **Birth size** | | |
| --- | --- | --- | --- | --- | --- | --- | --- | --- | --- |
| *Predictors* | *Est.* | *95% CI* | *p* | *Est.* | *95% CI* | *p* | *Est.* | *95% CI* | *p* |
| Intercept [ref, NW] | -1·10 | -1·27, -0·92 | **<0·0001** | -1·08 | -1·26, -0·91 | **<0·0001** | -0·99 | -1·16, -0·82 | **<0·0001** |
| Slope early discharge [NW] | 0·12 | 0·09, 0·15 | **<0·0001** | 0·12 | 0·09, 0·15 | **<0·0001** | 0·12 | 0·09, 0·15 | **<0·0001** |
| Difference in slope early vs· late discharge [NW] | -0·12 | -0·15, -0·09 | **<0·0001** | -0·12 | -0·15, -0·09 | **<0·0001** | -0·12 | -0·15, -0·09 | **<0·0001** |
| Difference in intercept early discharge [MW - NW] | -1·36 | -1·48, -1·24 | **<0·0001** | -1·35 | -1·47, -1·23 | **<0·0001** | -1·32 | -1·44, -1·21 | **<0·0001** |
| Difference in intercept early discharge [SW - NW] | -2·59 | -2·71, -2·48 | **<0·0001** | -2·58 | -2·70, -2·46 | **<0·0001** | -2·55 | -2·66, -2·44 | **<0·0001** |
| Difference in intercept early discharge [NO - NW] | -2·1 | -2·26, -1·93 | **<0·0001** | -2·08 | -2·25, -1·92 | **<0·0001** | -2·09 | -2·25, -1·92 | **<0·0001** |
| Age, months | 0·01 | -0·00, 0·02 | 0·057 | 0·01 | -0·00, 0·02 | 0·059 | 0·01 | -0·00, 0·01 | 0·123 |
| Sex, male | -0·25 | -0·33, -0·16 | **<0·0001** | -0·25 | -0·33, -0·16 | **<0·0001** | -0·26 | -0·34, -0·18 | **<0·0001** |
| Difference in slope early discharge [MW - NW] | 0·08 | 0·04, 0·13 | **0·0059** | 0·08 | 0·04, 0·13 | **0·0059** | 0·08 | 0·04, 0·13 | **0·0061** |
| Difference in slope early discharge [SW - NW] | 0·23 | 0·19, 0·28 | **<0·0001** | 0·23 | 0·19, 0·28 | **<0·0001** | 0·23 | 0·19, 0·28 | **<0·0001** |
| Difference in slope early discharge [NO - NW] | 0·32 | 0·26, 0·38 | **<0·0001** | 0·32 | 0·26, 0·38 | **<0·0001** | 0·32 | 0·26, 0·38 | **<0·0001** |
| Difference in change of slope between early and late discharge [MW - NW] | -0·03 | -0·08, 0·02 | 0·252 | -0·03 | -0·08, 0·02 | 0·252 | -0·03 | -0·08, 0·02 | 0·258 |
| Difference in change of slope between early and late discharge [SW - NW] | -0·14 | -0·19, -0·09 | **<0·0001** | -0·14 | -0·19, -0·09 | **<0·0001** | -0·14 | -0·19, -0·09 | **<0·0001** |
| Difference in change of slope between early and late discharge [NO - NW] | -0·2 | -0·27, -0·13 | **<0·0001** | -0·2 | -0·27, -0·13 | **<0·0001** | -0·2 | -0·27, -0·13 | **<0·0001** |
| HIV exposed |  |  |  | -0·11 | -0·29, 0·07 | 0·232 |  |  |  |
| HIV infected |  |  |  | -0·24 | -0·48, 0·00 | 0·054 |  |  |  |
| Small birth size |  |  |  |  |  |  | -0·6 | -0·71, -0·49 | **<0·0001** |
| **Random Effects** |  |  |  |  |  |  |  |  |  |
| σ^2^ | 0·1 | | | 0·1 | | | 0·1 | | |
| τ_00_ | 1·24 record_id | | | 1·24 record_id | | | 1·18 record_id | | |
|  | 0·03 site | | | 0·04 site | | | 0·03 site | | |
| τ_11_ | 0·12 record_id·time_months | | | 0·12 record_id·time_months | | | 0·12 record_id·time_months | | |
|  | 0·12 record_id·I(pmax(time_months - 1·5, 0)) | | | 0·12 record_id·I(pmax(time_months - 1·5, 0)) | | | 0·12 record_id·I(pmax(time_months - 1·5, 0)) | | |
| ρ_01_ | -0·19 record_id·time_months | | | -0·19 record_id·time_months | | | -0·19 record_id·time_months | | |
|  | 0·05 record_id·I(pmax(time_months - 1·5, 0)) | | | 0·05 record_id·I(pmax(time_months - 1·5, 0)) | | | 0·05 record_id·I(pmax(time_months - 1·5, 0)) | | |
| ICC | 0·93 | | | 0·93 | | | 0·93 | | |
| N | 9 _site_ | | | 9 _site_ | | | 9 _site_ | | |
|  | 2461 _record_id_ | | | 2461 _record_id_ | | | 2461 _record_id_ | | |
| Observations | 9448 | | | 9448 | | | 9448 | | |
| Marginal R^2^ / Conditional R^2^ | 0·392 / 0·957 | | | 0·391 / 0·957 | | | 0·416 / 0·957 | | |
| AIC | 19680·404 | | | 19685·104 | | | 19576·535 | | |
| AICc | 19680·512 | | | 19685·231 | | | 19576·652 | | |

Table presents results for both fixed and random terms of mixed piecewise models fit using *lme4* R package. A knot point was positioned at 45-days post-discharge which defines two discharge phases (i.e., early, before 45-days and, late, after 45-days). Time was coded in months (i.e., 1·5 months corresponds to 45-days; and 3 months to 90-days). Random structure included random slopes per participant and random intercepts for site with participants nested within. Models were fit with maximum likelihood for comparison with anova using Satterthwaite's approximation of degrees of freedom as implemented in the *lmerTest* R package (Kuznetsova, A. et al., 2017). Models were evaluated using fit metrics AIC, AICc, and BIC. Restricted maximum likelihood was used to fit final models. Groups: NW, no wasting; MW, moderate wasting; SW, severe wasting; NO, nutritional oedema. WAZ, weight-for-age z-score.

### **Supplemental Table 33**. Change in WAZ in the 180-days post-discharge per nutritional group associated with chronic medical conditions and prior hospitalisation.

|  | **Base model** | | | **Chronic medical condition** | | | **Prior hospitalisation** | | |
| --- | --- | --- | --- | --- | --- | --- | --- | --- | --- |
| *Predictors* | *Est.* | *95% CI* | *p* | *Est.* | *95% CI* | *p* | *Est.* | *95% CI* | *p* |
| Intercept [ref, NW] | -1·10 | -1·27, -0·92 | **<0·0001** | -1·09 | -1·26, -0·92 | **<0·0001** | -1·08 | -1·25, -0·91 | **<0·0001** |
| Slope early discharge [NW] | 0·12 | 0·09, 0·15 | **<0·0001** | 0·12 | 0·09, 0·15 | **<0·0001** | 0·12 | 0·09, 0·15 | **<0·0001** |
| Difference in slope early vs· late discharge [NW] | -0·12 | -0·15, -0·09 | **<0·0001** | -0·12 | -0·15, -0·09 | **<0·0001** | -0·12 | -0·15, -0·09 | **<0·0001** |
| Difference in intercept early discharge [MW - NW] | -1·36 | -1·48, -1·24 | **<0·0001** | -1·35 | -1·47, -1·23 | **<0·0001** | -1·36 | -1·48, -1·23 | **<0·0001** |
| Difference in intercept early discharge [SW - NW] | -2·59 | -2·71, -2·48 | **<0·0001** | -2·58 | -2·70, -2·47 | **<0·0001** | -2·59 | -2·70, -2·47 | **<0·0001** |
| Difference in intercept early discharge [NO - NW] | -2·1 | -2·26, -1·93 | **<0·0001** | -2·09 | -2·26, -1·93 | **<0·0001** | -2·1 | -2·26, -1·93 | **<0·0001** |
| Age, months | 0·01 | -0·00, 0·02 | 0·057 | 0·01 | 0·00, 0·02 | 0·043 | 0·01 | 0·00, 0·02 | 0·038 |
| Sex, male | -0·25 | -0·33, -0·16 | **<0·0001** | -0·25 | -0·33, -0·16 | **<0·0001** | -0·24 | -0·33, -0·16 | **<0·0001** |
| Difference in slope early discharge [MW - NW] | 0·08 | 0·04, 0·13 | **0·0059** | 0·08 | 0·04, 0·13 | **0·0059** | 0·08 | 0·04, 0·13 | **0·0059** |
| Difference in slope early discharge [SW - NW] | 0·23 | 0·19, 0·28 | **<0·0001** | 0·23 | 0·19, 0·28 | **<0·0001** | 0·23 | 0·19, 0·28 | **<0·0001** |
| Difference in slope early discharge [NO - NW] | 0·32 | 0·26, 0·38 | **<0·0001** | 0·32 | 0·26, 0·38 | **<0·0001** | 0·32 | 0·26, 0·38 | **<0·0001** |
| Difference in change of slope between early and late discharge [MW - NW] | -0·03 | -0·08, 0·02 | 0·252 | -0·03 | -0·08, 0·02 | 0·253 | -0·03 | -0·08, 0·02 | 0·253 |
| Difference in change of slope between early and late discharge [SW - NW] | -0·14 | -0·19, -0·09 | **<0·0001** | -0·14 | -0·19, -0·09 | **<0·0001** | -0·14 | -0·19, -0·09 | **<0·0001** |
| Difference in change of slope between early and late discharge [NO - NW] | -0·2 | -0·27, -0·13 | **<0·0001** | -0·2 | -0·27, -0·13 | **<0·0001** | -0·2 | -0·27, -0·13 | **<0·0001** |
| Chronic medical condition |  |  |  | -0·29 | -0·46, -0·12 | **0·0011** |  |  |  |
| Prior hospitalisation |  |  |  |  |  |  | -0·12 | -0·21, -0·02 | **0·020** |
| **Random Effects** |  |  |  |  |  |  |  |  |  |
| σ^2^ | 0·1 | | | 0·1 | | | 0·1 | | |
| τ_00_ | 1·24 record_id | | | 1·24 record_id | | | 1·24 record_id | | |
|  | 0·03 site | | | 0·03 site | | | 0·03 site | | |
| τ_11_ | 0·12 record_id·time_months | | | 0·12 record_id·time_months | | | 0·12 record_id·time_months | | |
|  | 0·12 record_id·I(pmax(time_months - 1·5, 0)) | | | 0·12 record_id·I(pmax(time_months - 1·5, 0)) | | | 0·12 record_id·I(pmax(time_months - 1·5, 0)) | | |
| ρ_01_ | -0·19 record_id·time_months | | | -0·19 record_id·time_months | | | -0·19 record_id·time_months | | |
|  | 0·05 record_id·I(pmax(time_months - 1·5, 0)) | | | 0·05 record_id·I(pmax(time_months - 1·5, 0)) | | | 0·05 record_id·I(pmax(time_months - 1·5, 0)) | | |
| ICC | 0·93 | | | 0·93 | | | 0·93 | | |
| N | 9 _site_ | | | 9 _site_ | | | 9 _site_ | | |
|  | 2461 _record_id_ | | | 2461 _record_id_ | | | 2461 _record_id_ | | |
| Observations | 9448 | | | 9448 | | | 9448 | | |
| Marginal R^2^ / Conditional R^2^ | 0·392 / 0·957 | | | 0·394 / 0·957 | | | 0·393 / 0·957 | | |
| AIC | 19680·404 | | | 19674·755 | | | 19681·199 | | |
| AICc | 19680·512 | | | 19674·872 | | | 19681·317 | | |

Table presents results for both fixed and random terms of mixed piecewise models fit using *lme4* R package. A knot point was positioned at 45-days post-discharge which defines two discharge phases (i.e., early, before 45-days and, late, after 45-days). Time was coded in months (i.e., 1·5 months corresponds to 45-days; and 3 months to 90-days). Random structure included random slopes per participant and random intercepts for site with participants nested within. Models were fit with maximum likelihood for comparison with anova using Satterthwaite's approximation of degrees of freedom as implemented in the *lmerTest* R package (Kuznetsova, A. et al., 2017). Models were evaluated using fit metrics AIC, AICc, and BIC. Restricted maximum likelihood was used to fit final models. Groups: NW, no wasting; MW, moderate wasting; SW, severe wasting; NO, nutritional oedema. WAZ, weight-for-age z-score.

### **Supplemental Table 34**. Change in MUACZ in the 180-days post-discharge per nutritional group associated with anaemia or diarrhoea diagnosed at admission.

|  | **Base model** | | | **Anemia, admission** | | | **Diarrhoea** | | |
| --- | --- | --- | --- | --- | --- | --- | --- | --- | --- |
| *Predictors* | *Est.* | *95% CI* | *p* | *Est.* | *95% CI* | *p* | *Est.* | *95% CI* | *p* |
| Intercept [ref, NW] | -0·88 | -1·03, -0·73 | **<0·0001** | -0·78 | -0·94, -0·62 | **<0·0001** | -0·9 | -1·06, -0·75 | **<0·001** |
| Slope early discharge [NW] | 0·17 | 0·13, 0·20 | **<0·0001** | 0·16 | 0·13, 0·20 | **<0·0001** | 0·17 | 0·13, 0·20 | **<0·0001** |
| Difference in slope early vs· late discharge [NW] | -0·13 | -0·17, -0·09 | **<0·0001** | -0·12 | -0·16, -0·08 | **<0·0001** | -0·13 | -0·17, -0·09 | **<0·0001** |
| Difference in intercept early discharge [MW - NW] | -1·4 | -1·50, -1·29 | **<0·0001** | -1·39 | -1·50, -1·28 | **<0·0001** | -1·4 | -1·51, -1·30 | **<0·0001** |
| Difference in intercept early discharge [SW - NW] | -2·55 | -2·65, -2·45 | **<0·0001** | -2·55 | -2·66, -2·45 | **<0·0001** | -2·56 | -2·66, -2·45 | **<0·0001** |
| Difference in intercept early discharge [NO - NW] | -2·13 | -2·28, -1·99 | **<0·0001** | -2·13 | -2·28, -1·99 | **<0·0001** | -2·13 | -2·28, -1·99 | **<0·0001** |
| Age, months | 0·02 | 0·01, 0·02 | **<0·0001** | 0·02 | 0·01, 0·02 | **<0·0001** | 0·02 | 0·01, 0·02 | **<0·0001** |
| Sex, male | -0·19 | -0·26, -0·12 | **<0·0001** | -0·16 | -0·23, -0·08 | **<0·0001** | -0·19 | -0·26, -0·12 | **<0·0001** |
| Difference in slope early discharge [MW - NW] | 0·19 | 0·14, 0·25 | **<0·0001** | 0·2 | 0·14, 0·26 | **<0·0001** | 0·19 | 0·14, 0·25 | **<0·0001** |
| Difference in slope early discharge [SW - NW] | 0·37 | 0·32, 0·43 | **<0·0001** | 0·37 | 0·32, 0·43 | **<0·0001** | 0·37 | 0·32, 0·43 | **<0·0001** |
| Difference in slope early discharge [NO - NW] | 0·5 | 0·42, 0·57 | **<0·0001** | 0·5 | 0·43, 0·58 | **<0·0001** | 0·5 | 0·42, 0·57 | **<0·0001** |
| Difference in change of slope between early and late discharge [MW - NW] | -0·15 | -0·21, -0·08 | **<0·0001** | -0·16 | -0·22, -0·09 | **<0·0001** | -0·15 | -0·21, -0·08 | **<0·0001** |
| Difference in change of slope between early and late discharge [SW - NW] | -0·28 | -0·34, -0·22 | **<0·0001** | -0·28 | -0·34, -0·22 | **<0·0001** | -0·28 | -0·34, -0·22 | **<0·0001** |
| Difference in change of slope between early and late discharge [NO - NW] | -0·37 | -0·45, -0·29 | **<0·0001** | -0·37 | -0·46, -0·29 | **<0·0001** | -0·37 | -0·45, -0·29 | **<0·0001** |
| Diarrhoea |  |  |  |  |  |  | 0·05 | -0·03, 0·13 | 0·23 |
| Anemia [Mild] |  |  |  | -0·04 | -0·14, 0·07 | 0·50 |  |  |  |
| Anemia [Moderate / Severe] |  |  |  | -0·19 | -0·29, -0·09 | **<0·0001** |  |  |  |
| **Random Effects** |  |  |  |  |  |  |  |  |  |
| σ^2^ | 0·14 | | | 0·14 | | | 0·14 | | |
| τ_00_ | 0·87 record_id | | | 0·85 record_id | | | 0·87 record_id | | |
|  | 0·02 site | | | 0·02 site | | | 0·02 site | | |
| τ_11_ | 0·15 record_id·time_months | | | 0·15 record_id·time_months | | | 0·15 record_id·time_months | | |
|  | 0·17 record_id·I(pmax(time_months - 1·5, 0)) | | | 0·17 record_id·I(pmax(time_months - 1·5, 0)) | | | 0·17 record_id·I(pmax(time_months - 1·5, 0)) | | |
| ρ_01_ | -0·20 record_id·time_months | | | -0·20 record_id·time_months | | | -0·20 record_id·time_months | | |
|  | 0·04 record_id·I(pmax(time_months - 1·5, 0)) | | | 0·04 record_id·I(pmax(time_months - 1·5, 0)) | | | 0·04 record_id·I(pmax(time_months - 1·5, 0)) | | |
| ICC | 0·87 | | | 0·87 | | | 0·87 | | |
| N | 9 _site_ | | | 9 _site_ | | | 9 _site_ | | |
|  | 2461 _record_id_ | | | 2357 _record_id_ | | | 2461 _record_id_ | | |
| Observations | 9364 | | | 8975 | | | 9364 | | |
| Marginal R^2^ / Conditional R^2^ | 0·436 / 0·927 | | | 0·443 / 0·925 | | | 0·435 / 0·927 | | |
| AIC | 20474·307 | | | 19607·917 | | | 20479·482 | | |
| AICc | 20474.416 | | | 19608.049 | | | 20479.603 | | |

Table presents results for both fixed and random terms of mixed piecewise models fit using *lme4* R package. A knot point was positioned at 45-days post-discharge which defines two discharge phases (i.e., early, before 45-days and, late, after 45-days). Time was coded in months (i.e., 1·5 months corresponds to 45-days; and 3 months to 90-days). Random structure included random slopes per participant and random intercepts for site with participants nested within. Models were fit with maximum likelihood for comparison with anova using Satterthwaite's approximation of degrees of freedom as implemented in the *lmerTest* R package (Kuznetsova, A. et al., 2017). Models were evaluated using fit metrics AIC, AICc, and BIC. Restricted maximum likelihood was used to fit final models. Groups: NW, no wasting; MW, moderate wasting; SW, severe wasting; NO, nutritional oedema. MUACZ, mid-upper arm circumference z-score.

### **Supplemental Table 35**. Change in MUACZ in the 180-days post-discharge per nutritional group associated with sepsis or pneumonia diagnosed at admission.

|  | **Base model** | | | **Sepsis** | | | **Pneumonia** | | |
| --- | --- | --- | --- | --- | --- | --- | --- | --- | --- |
| *Predictors* | *Est.* | *95% CI* | *p* | *Est.* | *95% CI* | *p* | *Est.* | *95% CI* | *p* |
| Intercept [ref, NW] | -0·88 | -1·03, -0·73 | **<0·0001** | -0·87 | -1·02, -0·71 | **<0·0001** | -0·89 | -1·04, -0·74 | **<0·0001** |
| Slope early discharge [NW] | 0·17 | 0·13, 0·20 | **<0·0001** | 0·17 | 0·13, 0·20 | **<0·0001** | 0·17 | 0·13, 0·20 | **<0·0001** |
| Difference in slope early vs· late discharge [NW] | -0·13 | -0·17, -0·09 | **<0·0001** | -0·13 | -0·17, -0·09 | **<0·0001** | -0·13 | -0·17, -0·09 | **<0·0001** |
| Difference in intercept early discharge [MW - NW] | -1·4 | -1·50, -1·29 | **<0·0001** | -1·4 | -1·50, -1·29 | **<0·0001** | -1·4 | -1·50, -1·29 | **<0·0001** |
| Difference in intercept early discharge [SW - NW] | -2·55 | -2·65, -2·45 | **<0·0001** | -2·55 | -2·65, -2·45 | **<0·0001** | -2·55 | -2·65, -2·44 | **<0·0001** |
| Difference in intercept early discharge [NO - NW] | -2·13 | -2·28, -1·99 | **<0·0001** | -2·13 | -2·28, -1·99 | **<0·0001** | -2·13 | -2·27, -1·98 | **<0·0001** |
| Age, months | 0·02 | 0·01, 0·02 | **<0·0001** | 0·02 | 0·01, 0·02 | **<0·0001** | 0·02 | 0·01, 0·02 | **<0·0001** |
| Sex, male | -0·19 | -0·26, -0·12 | **<0·0001** | -0·19 | -0·26, -0·12 | **<0·0001** | -0·19 | -0·26, -0·12 | **<0·0001** |
| Difference in slope early discharge [MW - NW] | 0·19 | 0·14, 0·25 | **<0·0001** | 0·19 | 0·14, 0·25 | **<0·0001** | 0·19 | 0·14, 0·25 | **<0·0001** |
| Difference in slope early discharge [SW - NW] | 0·37 | 0·32, 0·43 | **<0·0001** | 0·37 | 0·32, 0·43 | **<0·0001** | 0·37 | 0·32, 0·43 | **<0·0001** |
| Difference in slope early discharge [NO - NW] | 0·5 | 0·42, 0·57 | **<0·0001** | 0·5 | 0·42, 0·57 | **<0·0001** | 0·5 | 0·42, 0·57 | **<0·0001** |
| Difference in change of slope between early and late discharge [MW - NW] | -0·15 | -0·21, -0·08 | **<0·0001** | -0·15 | -0·21, -0·08 | **<0·0001** | -0·15 | -0·21, -0·08 | **<0·0001** |
| Difference in change of slope between early and late discharge [SW - NW] | -0·28 | -0·34, -0·22 | **<0·0001** | -0·28 | -0·34, -0·22 | **<0·0001** | -0·28 | -0·34, -0·22 | **<0·0001** |
| Difference in change of slope between early and late discharge [NO - NW] | -0·37 | -0·45, -0·29 | **<0·0001** | -0·37 | -0·45, -0·29 | **<0·0001** | -0·37 | -0·45, -0·29 | **<0·0001** |
| Sepsis |  |  |  | -0·12 | -0·24, -0·01 | 0·033 |  |  |  |
| Pneumonia |  |  |  |  |  |  | 0·02 | -0·06, 0·11 | 0·55 |
| **Random Effects** |  |  |  |  |  |  |  |  |  |
| σ^2^ | 0·14 | | | 0·14 | | | 0·14 | | |
| τ_00_ | 0·87 record_id | | | 0·87 record_id | | | 0·87 record_id | | |
|  | 0·02 site | | | 0·03 site | | | 0·03 site | | |
| τ_11_ | 0·15 record_id·time_months | | | 0·15 record_id·time_months | | | 0·15 record_id·time_months | | |
|  | 0·17 record_id·I(pmax(time_months - 1·5, 0)) | | | 0·17 record_id·I(pmax(time_months - 1·5, 0)) | | | 0·17 record_id·I(pmax(time_months - 1·5, 0)) | | |
| ρ_01_ | -0·20 record_id·time_months | | | -0·19 record_id·time_months | | | -0·20 record_id·time_months | | |
|  | 0·04 record_id·I(pmax(time_months - 1·5, 0)) | | | 0·04 record_id·I(pmax(time_months - 1·5, 0)) | | | 0·04 record_id·I(pmax(time_months - 1·5, 0)) | | |
| ICC | 0·87 | | | 0·87 | | | 0·87 | | |
| N | 9 _site_ | | | 9 _site_ | | | 9 _site_ | | |
|  | 2461 _record_id_ | | | 2461 _record_id_ | | | 2461 _record_id_ | | |
| Observations | 9364 | | | 9364 | | | 9364 | | |
| Marginal R^2^ / Conditional R^2^ | 0·436 / 0·927 | | | 0·435 / 0·927 | | | 0·436 / 0·927 | | |
| AIC | 20474·307 | | | 20475·629 | | | 20480·484 | | |
| AICc | 20474.416 | | | 20475.747 | | | 20480.604 | | |

Table presents results for both fixed and random terms of mixed piecewise models fit using *lme4* R package. A knot point was positioned at 45-days post-discharge which defines two discharge phases (i.e., early, before 45-days and, late, after 45-days). Time was coded in months (i.e., 1·5 months corresponds to 45-days; and 3 months to 90-days). Random structure included random slopes per participant and random intercepts for site with participants nested within. Models were fit with maximum likelihood for comparison with anova using Satterthwaite's approximation of degrees of freedom as implemented in the *lmerTest* R package (Kuznetsova, A. et al., 2017). Models were evaluated using fit metrics AIC, AICc, and BIC. Restricted maximum likelihood was used to fit final models. Groups: NW, no wasting; MW, moderate wasting; SW, severe wasting; NO, nutritional oedema. MUACZ, mid-upper arm circumference z-score.

### **Supplemental Table 36**. Change in MUACZ in the 180-days post-discharge per nutritional group associated with exposure domains of illness severity at admission and at discharge.

|  | **Base model** | | | **Illness severity  at admission** | | | **Illness severity  at discharge** | | |
| --- | --- | --- | --- | --- | --- | --- | --- | --- | --- |
| *Predictors* | *Est.* | *95% CI* | *p* | *Est.* | *95% CI* | *p* | *Est.* | *95% CI* | *p* |
| Intercept [ref, NW] | -0·88 | -1·03, -0·73 | **<0·0001** | -0·99 | -1·14, -0·83 | **<0·0001** | -0·88 | -1·02, -0·73 | **<0·0001** |
| Slope early discharge [NW] | 0·17 | 0·13, 0·20 | **<0·0001** | 0·17 | 0·13, 0·20 | **<0·0001** | 0·17 | 0·13, 0·20 | **<0·0001** |
| Difference in slope early vs· late discharge [NW] | -0·13 | -0·17, -0·09 | **<0·0001** | -0·13 | -0·17, -0·09 | **<0·0001** | -0·13 | -0·17, -0·09 | **<0·0001** |
| Difference in intercept early discharge [MW - NW] | -1·4 | -1·50, -1·29 | **<0·0001** | -1·39 | -1·50, -1·29 | **<0·0001** | -1·4 | -1·50, -1·29 | **<0·0001** |
| Difference in intercept early discharge [SW - NW] | -2·55 | -2·65, -2·45 | **<0·0001** | -2·54 | -2·64, -2·44 | **<0·0001** | -2·55 | -2·65, -2·45 | **<0·0001** |
| Difference in intercept early discharge [NO - NW] | -2·13 | -2·28, -1·99 | **<0·0001** | -2·1 | -2·24, -1·95 | **<0·0001** | -2·13 | -2·28, -1·99 | **<0·0001** |
| Age, months | 0·02 | 0·01, 0·02 | **<0·0001** | 0·02 | 0·01, 0·02 | **<0·0001** | 0·02 | 0·01, 0·02 | **<0·0001** |
| Sex, male | -0·19 | -0·26, -0·12 | **<0·0001** | -0·18 | -0·25, -0·11 | **<0·0001** | -0·19 | -0·26, -0·12 | **<0·0001** |
| Difference in slope early discharge [MW - NW] | 0·19 | 0·14, 0·25 | **<0·0001** | 0·19 | 0·14, 0·25 | **<0·0001** | 0·19 | 0·14, 0·25 | **<0·0001** |
| Difference in slope early discharge [SW - NW] | 0·37 | 0·32, 0·43 | **<0·0001** | 0·37 | 0·32, 0·43 | **<0·0001** | 0·37 | 0·32, 0·43 | **<0·0001** |
| Difference in slope early discharge [NO - NW] | 0·5 | 0·42, 0·57 | **<0·0001** | 0·5 | 0·42, 0·57 | **<0·0001** | 0·5 | 0·42, 0·57 | **<0·0001** |
| Difference in change of slope between early and late discharge [MW - NW] | -0·15 | -0·21, -0·08 | **<0·0001** | -0·15 | -0·21, -0·09 | **<0·0001** | -0·15 | -0·21, -0·08 | **<0·0001** |
| Difference in change of slope between early and late discharge [SW - NW] | -0·28 | -0·34, -0·22 | **<0·0001** | -0·28 | -0·34, -0·22 | **<0·0001** | -0·28 | -0·34, -0·22 | **<0·0001** |
| Difference in change of slope between early and late discharge [NO - NW] | -0·37 | -0·45, -0·29 | **<0·0001** | -0·37 | -0·46, -0·29 | **<0·0001** | -0·37 | -0·45, -0·29 | **<0·0001** |
| Illness severity at admission [medium] | |  |  | 0·17 | 0·08, 0·25 | **0.00010** |  |  |  |
| Illness severity at admission [high] | |  |  | 0·13 | 0·04, 0·22 | **0·0058** |  |  |  |
| Illness severity at discharge [medium or high] | | |  |  |  |  | -0·02 | -0·12, 0·08 | 0·69 |
| **Random Effects** |  |  |  |  |  |  |  |  |  |
| σ^2^ | 0·14 | | | 0·14 | | | 0·14 | | |
| τ_00_ | 0·87 record_id | | | 0·87 record_id | | | 0·87 record_id | | |
|  | 0·02 site | | | 0·02 site | | | 0·02 site | | |
| τ_11_ | 0·15 record_id·time_months | | | 0·15 record_id·time_months | | | 0·15 record_id·time_months | | |
|  | 0·17 record_id·I(pmax(time_months - 1·5, 0)) | | | 0·17 record_id·I(pmax(time_months - 1·5, 0)) | | | 0·17 record_id·I(pmax(time_months - 1·5, 0)) | | |
| ρ_01_ | -0·20 record_id·time_months | | | -0·20 record_id·time_months | | | -0·20 record_id·time_months | | |
|  | 0·04 record_id·I(pmax(time_months - 1·5, 0)) | | | 0·04 record_id·I(pmax(time_months - 1·5, 0)) | | | 0·04 record_id·I(pmax(time_months - 1·5, 0)) | | |
| ICC | 0·87 | | | 0·87 | | | 0·87 | | |
| N | 9 _site_ | | | 9 _site_ | | | 9 _site_ | | |
|  | 2461 _record_id_ | | | 2461 _record_id_ | | | 2461 _record_id_ | | |
| Observations | 9364 | | | 9364 | | | 9364 | | |
| Marginal R^2^ / Conditional R^2^ | 0·436 / 0·927 | | | 0·439 / 0·927 | | | 0·436 / 0·927 | | |
| AIC | 20474·307 | | | 20471·248 | | | 20480·257 | | |
| AICc | 20474.416 | | | 20471.376 | | | 20480.375 | | |

Table presents results for both fixed and random terms of mixed piecewise models fit using *lme4* R package. A knot point was positioned at 45-days post-discharge which defines two discharge phases (i.e., early, before 45-days and, late, after 45-days). Time was coded in months (i.e., 1·5 months corresponds to 45-days; and 3 months to 90-days). Random structure included random slopes per participant and random intercepts for site with participants nested within. Models were fit with maximum likelihood for comparison with anova using Satterthwaite's approximation of degrees of freedom as implemented in the *lmerTest* R package (Kuznetsova, A. et al., 2017). Models were evaluated using fit metrics AIC, AICc, and BIC. Restricted maximum likelihood was used to fit final models. Groups: NW, no wasting; MW, moderate wasting; SW, severe wasting; NO, nutritional oedema. MUACZ, mid-upper arm circumference z-score.

### **Supplemental Table 37**. Change in MUACZ in the 180-days post-discharge per nutritional group associated with exposure domains of age-inappropriate nutrition and caregiver characteristics.

|  | **Base model** | | | **Age-inappropriate nutrition** | | | **Caregiver  characteristics** | | |
| --- | --- | --- | --- | --- | --- | --- | --- | --- | --- |
| *Predictors* | *Est.* | *95% CI* | *p* | *Est.* | *95% CI* | *p* | *Est.* | *95% CI* | *p* |
| Intercept [ref, NW] | -0·88 | -1·03, -0·73 | **<0·0001** | -0·82 | -0·96, -0·69 | **<0·0001** | -0·81 | -0·96, -0·66 | **<0·0001** |
| Slope early discharge [NW] | 0·17 | 0·13, 0·20 | **<0·0001** | 0·17 | 0·13, 0·20 | **<0·0001** | 0·17 | 0·13, 0·20 | **<0·0001** |
| Difference in slope early vs· late discharge [NW] | -0·13 | -0·17, -0·09 | **<0·0001** | -0·13 | -0·17, -0·09 | **<0·0001** | -0·13 | -0·17, -0·09 | **<0·0001** |
| Difference in intercept early discharge [MW - NW] | -1·4 | -1·50, -1·29 | **<0·0001** | -1·32 | -1·43, -1·21 | **<0·0001** | -1·4 | -1·51, -1·29 | **<0·0001** |
| Difference in intercept early discharge [SW - NW] | -2·55 | -2·65, -2·45 | **<0·0001** | -2·37 | -2·48, -2·26 | **<0·0001** | -2·54 | -2·64, -2·44 | **<0·0001** |
| Difference in intercept early discharge [NO - NW] | -2·13 | -2·28, -1·99 | **<0·0001** | -1·94 | -2·09, -1·80 | **<0·0001** | -2·13 | -2·27, -1·98 | **<0·0001** |
| Age, months | 0·02 | 0·01, 0·02 | **<0·0001** | 0·02 | 0·01, 0·02 | **<0·0001** | 0·02 | 0·01, 0·02 | **<0·0001** |
| Sex, male | -0·19 | -0·26, -0·12 | **<0·0001** | -0·2 | -0·27, -0·13 | **<0·0001** | -0·18 | -0·25, -0·11 | **<0·0001** |
| Difference in slope early discharge [MW - NW] | 0·19 | 0·14, 0·25 | **<0·0001** | 0·19 | 0·14, 0·25 | **<0·0001** | 0·19 | 0·14, 0·25 | **<0·0001** |
| Difference in slope early discharge [SW - NW] | 0·37 | 0·32, 0·43 | **<0·0001** | 0·37 | 0·32, 0·43 | **<0·0001** | 0·37 | 0·32, 0·43 | **<0·0001** |
| Difference in slope early discharge [NO - NW] | 0·5 | 0·42, 0·57 | **<0·0001** | 0·5 | 0·42, 0·57 | **<0·0001** | 0·5 | 0·42, 0·57 | **<0·0001** |
| Difference in change of slope between early and late discharge [MW - NW] | -0·15 | -0·21, -0·08 | **<0·0001** | -0·15 | -0·21, -0·08 | **<0·0001** | -0·15 | -0·21, -0·08 | **<0·0001** |
| Difference in change of slope between early and late discharge [SW - NW] | -0·28 | -0·34, -0·22 | **<0·0001** | -0·28 | -0·34, -0·22 | **<0·0001** | -0·28 | -0·34, -0·22 | **<0·0001** |
| Difference in change of slope between early and late discharge [NO - NW] | -0·37 | -0·45, -0·29 | **<0·0001** | -0·37 | -0·45, -0·29 | **<0·0001** | -0·37 | -0·45, -0·29 | **<0·0001** |
| Age-inappropriate nutrition [medium] | |  |  | -0·2 | -0·32, -0·08 | **0.00081** |  |  |  |
| Age-inappropriate nutrition [high] | |  |  | -0·41 | -0·50, -0·32 | **<0·0001** |  |  |  |
| Caregiver Characteristics [Moderatly adverse] | | |  |  |  |  | -0·11 | -0·20, -0·02 | **0·014** |
| Caregiver Characteristics [Most adverse] | |  |  |  |  |  | -0·14 | -0·24, -0·05 | **0·0030** |
| Household-level exposures [Moderatly adverse] | | |  |  |  |  |  |  |  |
| Household-level exposures [Most adverse] | |  |  |  |  |  |  |  |  |
| Access to health care [Moderatly adverse] | |  |  |  |  |  |  |  |  |
| Access to health care [Least adverse] | |  |  |  |  |  |  |  |  |
| **Random Effects** |  |  |  |  |  |  |  |  |  |
| σ^2^ | 0·14 | | | 0·14 | | | 0·14 | | |
| τ_00_ | 0·87 record_id | | | 0·83 record_id | | | 0·87 record_id | | |
|  | 0·02 site | | | 0·02 site | | | 0·02 site | | |
| τ_11_ | 0·15 record_id·time_months | | | 0·15 record_id·time_months | | | 0·15 record_id·time_months | | |
|  | 0·17 record_id·I(pmax(time_months - 1·5, 0)) | | | 0·17 record_id·I(pmax(time_months - 1·5, 0)) | | | 0·17 record_id·I(pmax(time_months - 1·5, 0)) | | |
| ρ_01_ | -0·20 record_id·time_months | | | -0·18 record_id·time_months | | | -0·20 record_id·time_months | | |
|  | 0·04 record_id·I(pmax(time_months - 1·5, 0)) | | | 0·03 record_id·I(pmax(time_months - 1·5, 0)) | | | 0·04 record_id·I(pmax(time_months - 1·5, 0)) | | |
| ICC | 0·87 | | | 0·87 | | | 0·87 | | |
| N | 9 _site_ | | | 9 _site_ | | | 9 _site_ | | |
|  | 2461 _record_id_ | | | 2461 _record_id_ | | | 2461 _record_id_ | | |
| Observations | 9364 | | | 9364 | | | 9364 | | |
| Marginal R^2^ / Conditional R^2^ | 0·436 / 0·927 | | | 0·449 / 0·927 | | | 0·437 / 0·927 | | |
| AIC | 20474·307 | | | 20408·891 | | | 20476·775 | | |
| AICc | 20474.416 | | | 20409.02 | | | 20476.906 | | |

Table presents results for both fixed and random terms of mixed piecewise models fit using *lme4* R package. A knot point was positioned at 45-days post-discharge which defines two discharge phases (i.e., early, before 45-days and, late, after 45-days). Time was coded in months (i.e., 1·5 months corresponds to 45-days; and 3 months to 90-days). Random structure included random slopes per participant and random intercepts for site with participants nested within. Models were fit with maximum likelihood for comparison with anova using Satterthwaite's approximation of degrees of freedom as implemented in the *lmerTest* R package (Kuznetsova, A. et al., 2017). Models were evaluated using fit metrics AIC, AICc, and BIC. Restricted maximum likelihood was used to fit final models. Groups: NW, no wasting; MW, moderate wasting; SW, severe wasting; NO, nutritional oedema. MUACZ, mid-upper arm circumference z-score.

**Supplemental Table 38**. Change in MUACZ in the 180-days post-discharge per nutritional group associated with exposure domains of household-level exposures and access to health care.

|  | **Base model** | | | **Household-level  exposures** | | | **Access to  health care** | | | |
| --- | --- | --- | --- | --- | --- | --- | --- | --- | --- | --- |
| *Predictors* | *Est.* | *95% CI* | *p* | *Est.* | *95% CI* | *p* | *Est.* | *95% CI* | *p* |  |
| Intercept [ref, NW] | -0·88 | -1·03, -0·73 | **<0·0001** | -0·84 | -1·00, -0·68 | **<0·0001** | -0·85 | -1·01, -0·70 | **<0·0001** |  |
| Slope early discharge [NW] | 0·17 | 0·13, 0·20 | **<0·0001** | 0·17 | 0·13, 0·20 | **<0·0001** | 0·17 | 0·13, 0·20 | **<0·0001** |  |
| Difference in slope early vs· late discharge [NW] | -0·13 | -0·17, -0·09 | **<0·0001** | -0·13 | -0·17, -0·09 | **<0·0001** | -0·13 | -0·17, -0·09 | **<0·0001** |  |
| Difference in intercept early discharge [MW - NW] | -1·4 | -1·50, -1·29 | **<0·0001** | -1·39 | -1·50, -1·29 | **<0·0001** | -1·4 | -1·50, -1·29 | **<0·0001** |  |
| Difference in intercept early discharge [SW - NW] | -2·55 | -2·65, -2·45 | **<0·0001** | -2·54 | -2·65, -2·44 | **<0·0001** | -2·55 | -2·65, -2·45 | **<0·0001** |  |
| Difference in intercept early discharge [NO - NW] | -2·13 | -2·28, -1·99 | **<0·0001** | -2·12 | -2·26, -1·97 | **<0·0001** | -2·12 | -2·27, -1·98 | **<0·0001** |  |
| Age, months | 0·02 | 0·01, 0·02 | **<0·0001** | 0·02 | 0·01, 0·02 | **<0·0001** | 0·02 | 0·01, 0·02 | **<0·0001** |  |
| Sex, male | -0·19 | -0·26, -0·12 | **<0·0001** | -0·19 | -0·26, -0·12 | **<0·0001** | -0·19 | -0·26, -0·12 | **<0·0001** |  |
| Difference in slope early discharge [MW - NW] | 0·19 | 0·14, 0·25 | **<0·0001** | 0·19 | 0·14, 0·25 | **<0·0001** | 0·19 | 0·14, 0·25 | **<0·0001** |  |
| Difference in slope early discharge [SW - NW] | 0·37 | 0·32, 0·43 | **<0·0001** | 0·37 | 0·32, 0·43 | **<0·0001** | 0·37 | 0·32, 0·43 | **<0·0001** |  |
| Difference in slope early discharge [NO - NW] | 0·5 | 0·42, 0·57 | **<0·0001** | 0·5 | 0·42, 0·57 | **<0·0001** | 0·5 | 0·42, 0·57 | **<0·0001** |  |
| Difference in change of slope between early and late discharge [MW - NW] | -0·15 | -0·21, -0·08 | **<0·0001** | -0·15 | -0·21, -0·08 | **<0·0001** | -0·15 | -0·21, -0·08 | **<0·0001** |  |
| Difference in change of slope between early and late discharge [SW - NW] | -0·28 | -0·34, -0·22 | **<0·0001** | -0·28 | -0·34, -0·22 | **<0·0001** | -0·28 | -0·34, -0·22 | **<0·0001** |  |
| Difference in change of slope between early and late discharge [NO - NW] | -0·37 | -0·45, -0·29 | **<0·0001** | -0·37 | -0·45, -0·29 | **<0·0001** | -0·37 | -0·45, -0·29 | **<0·0001** |  |
| Caregiver Characteristics [Moderatly adverse] | | |  |  |  |  |  |  |  |  |
| Caregiver Characteristics [Most adverse] | |  |  |  |  |  |  |  |  |  |
| Household-level exposures [Moderatly adverse] | | |  | -0·05 | -0·14, 0·04 | 0·27 |  |  |  |  |
| Household-level exposures [Most adverse] | |  |  | -0·08 | -0·18, 0·03 | 0·145 |  |  |  |  |
| Access to health care [Moderatly adverse] | |  |  |  |  |  | 0 | -0·09, 0·09 | 0·975 |  |
| Access to health care [Least adverse] | |  |  |  |  |  | -0·08 | -0·18, 0·02 | 0·122 |  |
| **Random Effects** |  |  |  |  |  |  |  |  |  |  |
| σ^2^ | 0·14 | | | 0·14 | | | 0·14 | | | |
| τ_00_ | 0·87 record_id | | | 0·87 record_id | | | 0·87 record_id | | | |
|  | 0·02 site | | | 0·03 site | | | 0·02 site | | | |
| τ_11_ | 0·15 record_id·time_months | | | 0·15 record_id·time_months | | | 0·15 record_id·time_months | | | |
|  | 0·17 record_id·I(pmax(time_months - 1·5, 0)) | | | 0·17 record_id·I(pmax(time_months - 1·5, 0)) | | | 0·17 record_id·I(pmax(time_months - 1·5, 0)) | | | |
| ρ_01_ | -0·20 record_id·time_months | | | -0·20 record_id·time_months | | | -0·19 record_id·time_months | | | |
|  | 0·04 record_id·I(pmax(time_months - 1·5, 0)) | | | 0·04 record_id·I(pmax(time_months - 1·5, 0)) | | | 0·04 record_id·I(pmax(time_months - 1·5, 0)) | | | |
| ICC | 0·87 | | | 0·87 | | | 0·87 | | | |
| N | 9 _site_ | | | 9 _site_ | | | 9 _site_ | | | |
|  | 2461 _record_id_ | | | 2461 _record_id_ | | | 2461 _record_id_ | | | |
| Observations | 9364 | | | 9364 | | | 9364 | | | |
| Marginal R^2^ / Conditional R^2^ | 0·436 / 0·927 | | | 0·435 / 0·927 | | | 0·437 / 0·927 | | | |
| AIC | 20474·307 | | | 20484·648 | | | 20483·959 | | | |
| AICc | 20474.416 | | | 20484.777 | | | 20484.087 | | | |

Table presents results for both fixed and random terms of mixed piecewise models fit using *lme4* R package. A knot point was positioned at 45-days post-discharge which defines two discharge phases (i.e., early, before 45-days and, late, after 45-days). Time was coded in months (i.e., 1·5 months corresponds to 45-days; and 3 months to 90-days). Random structure included random slopes per participant and random intercepts for site with participants nested within. Models were fit with maximum likelihood for comparison with anova using Satterthwaite's approximation of degrees of freedom as implemented in the *lmerTest* R package (Kuznetsova, A. et al., 2017). Models were evaluated using fit metrics AIC, AICc, and BIC. Restricted maximum likelihood was used to fit final models. Groups: NW, no wasting; MW, moderate wasting; SW, severe wasting; NO, nutritional oedema. MUACZ, mid-upper arm circumference z-score.

### **Supplemental Table 39**. Change in MUACZ in the 180-days post-discharge per nutritional group associated with HIV and small birth size.

|  | **Base model** | | | **HIV status** | | | **Birth size** | | |
| --- | --- | --- | --- | --- | --- | --- | --- | --- | --- |
| *Predictors* | *Est.* | *95% CI* | *p* | *Est.* | *95% CI* | *p* | *Est.* | *95% CI* | *p* |
| Intercept [ref, NW] | -0·88 | -1·03, -0·73 | **<0·0001** | -0·88 | -1·03, -0·72 | **<0·0001** | -0·83 | -0·99, -0·68 | **<0·0001** |
| Slope early discharge [NW] | 0·17 | 0·13, 0·20 | **<0·0001** | 0·17 | 0·13, 0·20 | **<0·0001** | 0·17 | 0·13, 0·20 | **<0·0001** |
| Difference in slope early vs· late discharge [NW] | -0·13 | -0·17, -0·09 | **<0·0001** | -0·13 | -0·17, -0·09 | **<0·0001** | -0·13 | -0·17, -0·09 | **<0·0001** |
| Difference in intercept early discharge [MW - NW] | -1·4 | -1·50, -1·29 | **<0·0001** | -1·39 | -1·50, -1·28 | **<0·0001** | -1·38 | -1·49, -1·28 | **<0·0001** |
| Difference in intercept early discharge [SW - NW] | -2·55 | -2·65, -2·45 | **<0·0001** | -2·54 | -2·64, -2·44 | **<0·0001** | -2·53 | -2·63, -2·43 | **<0·0001** |
| Difference in intercept early discharge [NO - NW] | -2·13 | -2·28, -1·99 | **<0·0001** | -2·12 | -2·27, -1·98 | **<0·0001** | -2·13 | -2·27, -1·98 | **<0·0001** |
| Age, months | 0·02 | 0·01, 0·02 | **<0·0001** | 0·02 | 0·01, 0·02 | **<0·0001** | 0·02 | 0·01, 0·02 | **<0·0001** |
| Sex, male | -0·19 | -0·26, -0·12 | **<0·0001** | -0·19 | -0·26, -0·12 | **<0·0001** | -0·19 | -0·26, -0·12 | **<0·0001** |
| Difference in slope early discharge [MW - NW] | 0·19 | 0·14, 0·25 | **<0·0001** | 0·19 | 0·14, 0·25 | **<0·0001** | 0·19 | 0·14, 0·25 | **<0·0001** |
| Difference in slope early discharge [SW - NW] | 0·37 | 0·32, 0·43 | **<0·0001** | 0·37 | 0·32, 0·43 | **<0·0001** | 0·37 | 0·32, 0·43 | **<0·0001** |
| Difference in slope early discharge [NO - NW] | 0·5 | 0·42, 0·57 | **<0·0001** | 0·5 | 0·42, 0·57 | **<0·0001** | 0·5 | 0·42, 0·57 | **<0·0001** |
| Difference in change of slope between early and late discharge [MW - NW] | -0·15 | -0·21, -0·08 | **<0·0001** | -0·15 | -0·21, -0·08 | **<0·0001** | -0·15 | -0·21, -0·08 | **<0·0001** |
| Difference in change of slope between early and late discharge [SW - NW] | -0·28 | -0·34, -0·22 | **<0·0001** | -0·28 | -0·34, -0·22 | **<0·0001** | -0·28 | -0·34, -0·22 | **<0·0001** |
| Difference in change of slope between early and late discharge [NO - NW] | -0·37 | -0·45, -0·29 | **<0·0001** | -0·37 | -0·45, -0·29 | **<0·0001** | -0·37 | -0·46, -0·29 | **<0·0001** |
| HIV exposed |  |  |  | 0·01 | -0·14, 0·16 | 0·92 |  |  |  |
| HIV infected |  |  |  | -0·26 | -0·46, -0·05 | **0·013** |  |  |  |
| Small birth size |  |  |  |  |  |  | -0·26 | -0·36, -0·17 | **<0·0001** |
| **Random Effects** |  |  |  |  |  |  |  |  |  |
| σ^2^ | 0·14 | | | 0·14 | | | 0·14 | | |
| τ_00_ | 0·87 record_id | | | 0·86 record_id | | | 0·86 record_id | | |
|  | 0·02 site | | | 0·03 site | | | 0·03 site | | |
| τ_11_ | 0·15 record_id·time_months | | | 0·15 record_id·time_months | | | 0·15 record_id·time_months | | |
|  | 0·17 record_id·I(pmax(time_months - 1·5, 0)) | | | 0·17 record_id·I(pmax(time_months - 1·5, 0)) | | | 0·17 record_id·I(pmax(time_months - 1·5, 0)) | | |
| ρ_01_ | -0·20 record_id·time_months | | | -0·19 record_id·time_months | | | -0·20 record_id·time_months | | |
|  | 0·04 record_id·I(pmax(time_months - 1·5, 0)) | | | 0·04 record_id·I(pmax(time_months - 1·5, 0)) | | | 0·04 record_id·I(pmax(time_months - 1·5, 0)) | | |
| ICC | 0·87 | | | 0·87 | | | 0·87 | | |
| N | 9 _site_ | | | 9 _site_ | | | 9 _site_ | | |
|  | 2461 _record_id_ | | | 2461 _record_id_ | | | 2461 _record_id_ | | |
| Observations | 9364 | | | 9364 | | | 9364 | | |
| Marginal R^2^ / Conditional R^2^ | 0·436 / 0·927 | | | 0·436 / 0·927 | | | 0·441 / 0·927 | | |
| AIC | 20474·307 | | | 20478·003 | | | 20450·689 | | |
| AICc | 20474.416 | | | 20478.133 | | | 20450.808 | | |

Table presents results for both fixed and random terms of mixed piecewise models fit using *lme4* R package. A knot point was positioned at 45-days post-discharge which defines two discharge phases (i.e., early, before 45-days and, late, after 45-days). Time was coded in months (i.e., 1·5 months corresponds to 45-days; and 3 months to 90-days). Random structure included random slopes per participant and random intercepts for site with participants nested within. Models were fit with maximum likelihood for comparison with anova using Satterthwaite's approximation of degrees of freedom as implemented in the *lmerTest* R package (Kuznetsova, A. et al., 2017). Models were evaluated using fit metrics AIC, AICc, and BIC. Restricted maximum likelihood was used to fit final models. Groups: NW, no wasting; MW, moderate wasting; SW, severe wasting; NO, nutritional oedema. MUACZ, mid-upper arm circumference z-score.

### **Supplemental Table 40**. Change in WAZ in the 180-days post-discharge per nutritional group associated with chronic medical conditions and prior hospitalisation.

|  | **Base model** | | | **Chronic medical condition** | | | **Prior hospitalisation** | | | |
| --- | --- | --- | --- | --- | --- | --- | --- | --- | --- | --- |
| *Predictors* | *Est.* | *95% CI* | *p* | *Est.* | *95% CI* | *p* | *Est.* | *95% CI* | *p* |  |
| Intercept [ref, NW] | -0·88 | -1·03, -0·73 | **<0·0001** | -0·87 | -1·02, -0·73 | **<0·0001** | -0·87 | -1·02, -0·73 | **<0·0001** |  |
| Slope early discharge [NW] | 0·17 | 0·13, 0·20 | **<0·0001** | 0·17 | 0·13, 0·20 | **<0·0001** | 0·17 | 0·13, 0·20 | **<0·0001** |  |
| Difference in slope early vs· late discharge [NW] | -0·13 | -0·17, -0·09 | **<0·0001** | -0·13 | -0·17, -0·09 | **<0·0001** | -0·13 | -0·17, -0·09 | **<0·0001** |  |
| Difference in intercept early discharge [MW - NW] | -1·4 | -1·50, -1·29 | **<0·0001** | -1·39 | -1·50, -1·29 | **<0·0001** | -1·4 | -1·50, -1·29 | **<0·0001** |  |
| Difference in intercept early discharge [SW - NW] | -2·55 | -2·65, -2·45 | **<0·0001** | -2·54 | -2·64, -2·44 | **<0·0001** | -2·55 | -2·65, -2·44 | **<0·0001** |  |
| Difference in intercept early discharge [NO - NW] | -2·13 | -2·28, -1·99 | **<0·0001** | -2·13 | -2·28, -1·99 | **<0·0001** | -2·13 | -2·28, -1·99 | **<0·0001** |  |
| Age, months | 0·02 | 0·01, 0·02 | **<0·0001** | 0·02 | 0·01, 0·02 | **<0·0001** | 0·02 | 0·01, 0·02 | **<0·0001** |  |
| Sex, male | -0·19 | -0·26, -0·12 | **<0·0001** | -0·19 | -0·26, -0·12 | **<0·0001** | -0·18 | -0·25, -0·11 | **<0·0001** |  |
| Difference in slope early discharge [MW - NW] | 0·19 | 0·14, 0·25 | **<0·0001** | 0·19 | 0·14, 0·25 | **<0·0001** | 0·19 | 0·14, 0·25 | **<0·0001** |  |
| Difference in slope early discharge [SW - NW] | 0·37 | 0·32, 0·43 | **<0·0001** | 0·37 | 0·32, 0·43 | **<0·0001** | 0·37 | 0·32, 0·43 | **<0·0001** |  |
| Difference in slope early discharge [NO - NW] | 0·5 | 0·42, 0·57 | **<0·0001** | 0·5 | 0·42, 0·57 | **<0·0001** | 0·5 | 0·42, 0·57 | **<0·0001** |  |
| Difference in change of slope between early and late discharge [MW - NW] | -0·15 | -0·21, -0·08 | **<0·0001** | -0·15 | -0·21, -0·08 | **<0·0001** | -0·15 | -0·21, -0·08 | **<0·0001** |  |
| Difference in change of slope between early and late discharge [SW - NW] | -0·28 | -0·34, -0·22 | **<0·0001** | -0·28 | -0·34, -0·22 | **<0·0001** | -0·28 | -0·34, -0·22 | **<0·0001** |  |
| Difference in change of slope between early and late discharge [NO - NW] | -0·37 | -0·45, -0·29 | **<0·0001** | -0·37 | -0·45, -0·29 | **<0·0001** | -0·37 | -0·45, -0·29 | **<0·0001** |  |
| Chronic medical condition |  |  |  | -0·17 | -0·31, -0·03 | **0·021** |  |  |  |  |
| Prior hospitalisation |  |  |  |  |  |  | -0·05 | -0·13, 0·03 | 0·21 |  |
| **Random Effects** |  |  |  |  |  |  |  |  |  |  |
| σ^2^ | 0·14 | | | 0·14 | | | 0·14 | | | |
| τ_00_ | 0·87 record_id | | | 0·87 record_id | | | 0·87 record_id | | | |
|  | 0·02 site | | | 0·02 site | | | 0·02 site | | | |
| τ_11_ | 0·15 record_id·time_months | | | 0·15 record_id·time_months | | | 0·15 record_id·time_months | | | |
|  | 0·17 record_id·I(pmax(time_months - 1·5, 0)) | | | 0·17 record_id·I(pmax(time_months - 1·5, 0)) | | | 0·17 record_id·I(pmax(time_months - 1·5, 0)) | | | |
| ρ_01_ | -0·20 record_id·time_months | | | -0·20 record_id·time_months | | | -0·20 record_id·time_months | | | |
|  | 0·04 record_id·I(pmax(time_months - 1·5, 0)) | | | 0·04 record_id·I(pmax(time_months - 1·5, 0)) | | | 0·04 record_id·I(pmax(time_months - 1·5, 0)) | | | |
| ICC | 0·87 | | | 0·87 | | | 0·87 | | | |
| N | 9 _site_ | | | 9 _site_ | | | 9 _site_ | | | |
|  | 2461 _record_id_ | | | 2461 _record_id_ | | | 2461 _record_id_ | | | |
| Observations | 9364 | | | 9364 | | | 9364 | | | |
| Marginal R^2^ / Conditional R^2^ | 0·436 / 0·927 | | | 0·436 / 0·927 | | | 0·436 / 0·927 | | | |
| AIC | 20474·307 | | | 20474·456 | | | 20479·272 | | | |
| AICc | 20474.416 | | | 20474.575 | | | 20479.39 | | | |

Table presents results for both fixed and random terms of mixed piecewise models fit using *lme4* R package. A knot point was positioned at 45-days post-discharge which defines two discharge phases (i.e., early, before 45-days and, late, after 45-days). Time was coded in months (i.e., 1·5 months corresponds to 45-days; and 3 months to 90-days). Random structure included random slopes per participant and random intercepts for site with participants nested within. Models were fit with maximum likelihood for comparison with anova using Satterthwaite's approximation of degrees of freedom as implemented in the *lmerTest* R package (Kuznetsova, A. et al., 2017). Models were evaluated using fit metrics AIC, AICc, and BIC. Restricted maximum likelihood was used to fit final models. Groups: NW, no wasting; MW, moderate wasting; SW, severe wasting; NO, nutritional oedema. MUACZ, mid-upper arm circumference z-score.

### **Supplemental Table 41**. Change in WLZ in the 180-days post-discharge per nutritional group associated with anaemia or diarrhoea diagnosed at admission.

|  | **Base model** | | | **Anaemia** | | | **Diarrhoea** | | |
| --- | --- | --- | --- | --- | --- | --- | --- | --- | --- |
| *Predictors* | *Est.* | *95% CI* | *p* | *Est.* | *95% CI* | *p* | *Est.* | *95% CI* | *p* |
| Intercept [ref, NW] | -0·28 | -0·42, -0·14 | **<0·0001** | -0·23 | -0·39, -0·08 | **0·0028** | -0·29 | -0·43, -0·14 | **0.00012** |
| Slope early discharge [NW] | 0·22 | 0·18, 0·26 | **<0·0001** | 0·22 | 0·18, 0·26 | **<0·0001** | 0·22 | 0·18, 0·26 | **<0·0001** |
| Difference in slope early vs· late discharge [NW] | -0·23 | -0·27, -0·18 | **<0·0001** | -0·23 | -0·27, -0·18 | **<0·0001** | -0·23 | -0·27, -0·18 | **<0·0001** |
| Difference in intercept early discharge [MW - NW] | -1·43 | -1·53, -1·32 | **<0·0001** | -1·43 | -1·54, -1·32 | **<0·0001** | -1·43 | -1·53, -1·32 | **<0·0001** |
| Difference in intercept early discharge [SW - NW] | -2·56 | -2·66, -2·46 | **<0·0001** | -2·55 | -2·65, -2·45 | **<0·0001** | -2·56 | -2·66, -2·46 | **<0·0001** |
| Difference in intercept early discharge [NO - NW] | -1·8 | -1·94, -1·65 | **<0·0001** | -1·78 | -1·93, -1·64 | **<0·0001** | -1·8 | -1·94, -1·65 | **<0·0001** |
| Age, months | -0·01 | -0·01, -0·00 | **0·026** | -0·01 | -0·01, -0·00 | **0·030** | -0·01 | -0·01, -0·00 | **0·027** |
| Sex, male | -0·2 | -0·27, -0·13 | **<0·0001** | -0·18 | -0·25, -0·10 | **<0·0001** | -0·2 | -0·27, -0·13 | **<0·0001** |
| Difference in slope early discharge [MW - NW] | 0·13 | 0·07, 0·20 | **<0·0001** | 0·15 | 0·08, 0·21 | **<0·0001** | 0·13 | 0·07, 0·20 | **<0·0001** |
| Difference in slope early discharge [SW - NW] | 0·37 | 0·30, 0·43 | **<0·0001** | 0·36 | 0·30, 0·42 | **<0·0001** | 0·37 | 0·30, 0·43 | **<0·0001** |
| Difference in slope early discharge [NO - NW] | 0·44 | 0·36, 0·53 | **<0·0001** | 0·44 | 0·35, 0·53 | **<0·0001** | 0·44 | 0·36, 0·53 | **<0·0001** |
| Difference in change of slope between early and late discharge [MW - NW] | -0·07 | -0·15, 0·00 | 0·054 | -0·08 | -0·16, -0·01 | 0·031 | -0·07 | -0·15, 0·00 | 0·054 |
| Difference in change of slope between early and late discharge [SW - NW] | -0·26 | -0·33, -0·18 | **<0·0001** | -0·25 | -0·32, -0·18 | **<0·0001** | -0·26 | -0·33, -0·18 | **<0·0001** |
| Difference in change of slope between early and late discharge [NO - NW] | -0·27 | -0·37, -0·17 | **<0·0001** | -0·27 | -0·37, -0·17 | **<0·0001** | -0·27 | -0·37, -0·17 | **<0·0001** |
| Diarrhoea |  |  |  |  |  |  | 0·01 | -0·06, 0·09 | 0·71 |
| Anemia [Mild] |  |  |  | -0·06 | -0·17, 0·05 | 0·26 |  |  |  |
| Anemia [Moderate / Severe] |  |  |  | -0·08 | -0·18, 0·01 | 0·093 |  |  |  |
| **Random Effects** |  |  |  |  |  |  |  |  |  |
| σ^2^ | 0·23 | | | 0·23 | | | 0·23 | | |
| τ_00_ | 0·77 record_id | | | 0·76 record_id | | | 0·77 record_id | | |
|  | 0·02 site | | | 0·02 site | | | 0·02 site | | |
| τ_11_ | 0·20 record_id·time_months | | | 0·20 record_id·time_months | | | 0·20 record_id·time_months | | |
|  | 0·22 record_id·I(pmax(time_months - 1·5, 0)) | | | 0·22 record_id·I(pmax(time_months - 1·5, 0)) | | | 0·22 record_id·I(pmax(time_months - 1·5, 0)) | | |
| ρ_01_ | -0·20 record_id·time_months | | | -0·20 record_id·time_months | | | -0·20 record_id·time_months | | |
|  | 0·09 record_id·I(pmax(time_months - 1·5, 0)) | | | 0·09 record_id·I(pmax(time_months - 1·5, 0)) | | | 0·09 record_id·I(pmax(time_months - 1·5, 0)) | | |
| ICC | 0·81 | | | 0·81 | | | 0·81 | | |
| N | 9 _site_ | | | 9 _site_ | | | 9 _site_ | | |
|  | 2461 _record_id_ | | | 2357 _record_id_ | | | 2461 _record_id_ | | |
| Observations | 9413 | | | 9029 | | | 9413 | | |
| Marginal R^2^ / Conditional R^2^ | 0·408 / 0·885 | | | 0·410 / 0·885 | | | 0·407 / 0·885 | | |
| AIC | 23228·917 | | | 22266·077 | | | 23235·352 | | |
| AICc | 23229.023 | | | 22266.21 | | | 23235.469 | | |

Table presents results for both fixed and random terms of mixed piecewise models fit using *lme4* R package. A knot point was positioned at 45-days post-discharge which defines two discharge phases (i.e., early, before 45-days and, late, after 45-days). Time was coded in months (i.e., 1·5 months corresponds to 45-days; and 3 months to 90-days). Random structure included random slopes per participant and random intercepts for site with participants nested within. Models were fit with maximum likelihood for comparison with anova using Satterthwaite's approximation of degrees of freedom as implemented in the *lmerTest* R package (Kuznetsova, A. et al., 2017). Models were evaluated using fit metrics AIC, AICc, and BIC. Restricted maximum likelihood was used to fit final models. Groups: NW, no wasting; MW, moderate wasting; SW, severe wasting; NO, nutritional oedema. WLZ, weight-for-length z-score.

### **Supplemental Table 42**. Change in WLZ in the 180-days post-discharge per nutritional group associated with sepsis or pneumonia diagnosed at admission.

|  | **Base model** | | | **Sepsis** | | | **Pneumonia** | | |
| --- | --- | --- | --- | --- | --- | --- | --- | --- | --- |
| *Predictors* | *Est.* | *95% CI* | *p* | *Est.* | *95% CI* | *p* | *Est.* | *95% CI* | *p* |
| Intercept [ref, NW] | -0·28 | -0·42, -0·14 | **<0·0001** | -0·26 | -0·41, -0·12 | **0.00040** | -0·3 | -0·44, -0·16 | **<0·0001** |
| Slope early discharge [NW] | 0·22 | 0·18, 0·26 | **<0·0001** | 0·22 | 0·18, 0·26 | **<0·0001** | 0·22 | 0·18, 0·26 | **<0·0001** |
| Difference in slope early vs· late discharge [NW] | -0·23 | -0·27, -0·18 | **<0·0001** | -0·23 | -0·27, -0·18 | **<0·0001** | -0·23 | -0·27, -0·18 | **<0·0001** |
| Difference in intercept early discharge [MW - NW] | -1·43 | -1·53, -1·32 | **<0·0001** | -1·42 | -1·53, -1·32 | **<0·0001** | -1·42 | -1·53, -1·32 | **<0·0001** |
| Difference in intercept early discharge [SW - NW] | -2·56 | -2·66, -2·46 | **<0·0001** | -2·56 | -2·66, -2·46 | **<0·0001** | -2·55 | -2·65, -2·45 | **<0·0001** |
| Difference in intercept early discharge [NO - NW] | -1·8 | -1·94, -1·65 | **<0·0001** | -1·8 | -1·94, -1·66 | **<0·0001** | -1·79 | -1·93, -1·64 | **<0·0001** |
| Age, months | -0·01 | -0·01, -0·00 | **0·026** | -0·01 | -0·01, -0·00 | **0·024** | -0·01 | -0·01, -0·00 | **0·032** |
| Sex, male | -0·2 | -0·27, -0·13 | **<0·0001** | -0·2 | -0·27, -0·13 | **<0·0001** | -0·2 | -0·27, -0·13 | **<0·0001** |
| Difference in slope early discharge [MW - NW] | 0·13 | 0·07, 0·20 | **<0·0001** | 0·13 | 0·07, 0·20 | **<0·0001** | 0·13 | 0·07, 0·20 | **<0·0001** |
| Difference in slope early discharge [SW - NW] | 0·37 | 0·30, 0·43 | **<0·0001** | 0·37 | 0·30, 0·43 | **<0·0001** | 0·37 | 0·30, 0·43 | **<0·0001** |
| Difference in slope early discharge [NO - NW] | 0·44 | 0·36, 0·53 | **<0·0001** | 0·44 | 0·36, 0·53 | **<0·0001** | 0·44 | 0·36, 0·53 | **<0·0001** |
| Difference in change of slope between early and late discharge [MW - NW] | -0·07 | -0·15, 0·00 | 0·054 | -0·07 | -0·15, 0·00 | 0·054 | -0·07 | -0·15, 0·00 | 0·054 |
| Difference in change of slope between early and late discharge [SW - NW] | -0·26 | -0·33, -0·18 | **<0·0001** | -0·26 | -0·33, -0·18 | **<0·0001** | -0·26 | -0·33, -0·19 | **<0·0001** |
| Difference in change of slope between early and late discharge [NO - NW] | -0·27 | -0·37, -0·17 | **<0·0001** | -0·27 | -0·37, -0·17 | **<0·0001** | -0·27 | -0·37, -0·17 | **<0·0001** |
| Sepsis |  |  |  | -0·12 | -0·23, -0·01 | **0·040** |  |  |  |
| Pneumonia |  |  |  |  |  |  | 0·05 | -0·03, 0·13 | 0·27 |
| Diarrhoea |  |  |  |  |  |  |  |  |  |
| Anemia [Mild] |  |  |  |  |  |  |  |  |  |
| Anemia [Moderate / Severe] |  |  |  |  |  |  |  |  |  |
| Caregiver BMI [Low] |  |  |  |  |  |  |  |  |  |
| Cargiver BMI [Overweight] |  |  |  |  |  |  |  |  |  |
| Caregiver BMI [Obese] |  |  |  |  |  |  |  |  |  |
| Caregiver stunted |  |  |  |  |  |  |  |  |  |
| **Random Effects** |  |  |  |  |  |  |  |  |  |
| σ^2^ | 0·23 | | | 0·23 | | | 0·23 | | |
| τ_00_ | 0·77 record_id | | | 0·76 record_id | | | 0·77 record_id | | |
|  | 0·02 site | | | 0·02 site | | | 0·02 site | | |
| τ_11_ | 0·20 record_id·time_months | | | 0·20 record_id·time_months | | | 0·20 record_id·time_months | | |
|  | 0·22 record_id·I(pmax(time_months - 1·5, 0)) | | | 0·22 record_id·I(pmax(time_months - 1·5, 0)) | | | 0·22 record_id·I(pmax(time_months - 1·5, 0)) | | |
| ρ_01_ | -0·20 record_id·time_months | | | -0·20 record_id·time_months | | | -0·20 record_id·time_months | | |
|  | 0·09 record_id·I(pmax(time_months - 1·5, 0)) | | | 0·09 record_id·I(pmax(time_months - 1·5, 0)) | | | 0·09 record_id·I(pmax(time_months - 1·5, 0)) | | |
| ICC | 0·81 | | | 0·81 | | | 0·81 | | |
| N | 9 _site_ | | | 9 _site_ | | | 9 _site_ | | |
|  | 2461 _record_id_ | | | 2461 _record_id_ | | | 2461 _record_id_ | | |
| Observations | 9413 | | | 9413 | | | 9413 | | |
| Marginal R^2^ / Conditional R^2^ | 0·408 / 0·885 | | | 0·407 / 0·885 | | | 0·408 / 0·885 | | |
| AIC | 23228·917 | | | 23230·534 | | | 23234·224 | | |
| AICc | 23229.023 | | | 23230.651 | | | 23234.339 | | |

Table presents results for both fixed and random terms of mixed piecewise models fit using *lme4* R package. A knot point was positioned at 45-days post-discharge which defines two discharge phases (i.e., early, before 45-days and, late, after 45-days). Time was coded in months (i.e., 1·5 months corresponds to 45-days; and 3 months to 90-days). Random structure included random slopes per participant and random intercepts for site with participants nested within. Models were fit with maximum likelihood for comparison with anova using Satterthwaite's approximation of degrees of freedom as implemented in the *lmerTest* R package (Kuznetsova, A. et al., 2017). Models were evaluated using fit metrics AIC, AICc, and BIC. Restricted maximum likelihood was used to fit final models. Groups: NW, no wasting; MW, moderate wasting; SW, severe wasting; NO, nutritional oedema. WLZ, weight-for-length z-score.

**Supplemental Table 43**. Change in WLZ in the 180-days post-discharge per nutritional group associated with exposure domains of illness severity at admission and at discharge.

|  | **Base model** | | | **Illness severity  at admission** | | | **Illness severity  at discharge** | | |
| --- | --- | --- | --- | --- | --- | --- | --- | --- | --- |
| *Predictors* | *Est.* | *95% CI* | *p* | *Est.* | *95% CI* | *p* | *Est.* | *95% CI* | *p* |
| Intercept [ref, NW] | -0·28 | -0·42, -0·14 | **<0·0001** | -0·38 | -0·53, -0·23 | **<0·0001** | -0·28 | -0·42, -0·13 | **0·00013** |
| Slope early discharge [NW] | 0·22 | 0·18, 0·26 | **<0·0001** | 0·22 | 0·18, 0·26 | **<0·0001** | 0·22 | 0·18, 0·26 | **<0·0001** |
| Difference in slope early vs· late discharge [NW] | -0·23 | -0·27, -0·18 | **<0·0001** | -0·23 | -0·27, -0·18 | **<0·0001** | -0·23 | -0·27, -0·18 | **<0·0001** |
| Difference in intercept early discharge [MW - NW] | -1·43 | -1·53, -1·32 | **<0·0001** | -1·42 | -1·53, -1·32 | **<0·0001** | -1·43 | -1·53, -1·32 | **<0·0001** |
| Difference in intercept early discharge [SW - NW] | -2·56 | -2·66, -2·46 | **<0·0001** | -2·55 | -2·65, -2·45 | **<0·0001** | -2·56 | -2·66, -2·46 | **<0·0001** |
| Difference in intercept early discharge [NO - NW] | -1·8 | -1·94, -1·65 | **<0·0001** | -1·76 | -1·91, -1·62 | **<0·0001** | -1·8 | -1·94, -1·65 | **<0·0001** |
| Age, months | -0·01 | -0·01, -0·00 | **0·026** | -0·01 | -0·01, -0·00 | **0·042** | -0·01 | -0·01, -0·00 | **0·027** |
| Sex, male | -0·2 | -0·27, -0·13 | **<0·0001** | -0·2 | -0·27, -0·13 | **<0·0001** | -0·2 | -0·27, -0·13 | **<0·0001** |
| Difference in slope early discharge [MW - NW] | 0·13 | 0·07, 0·20 | **<0·0001** | 0·13 | 0·07, 0·20 | **<0·0001** | 0·13 | 0·07, 0·20 | **<0·0001** |
| Difference in slope early discharge [SW - NW] | 0·37 | 0·30, 0·43 | **<0·0001** | 0·37 | 0·30, 0·43 | **<0·0001** | 0·37 | 0·30, 0·43 | **<0·0001** |
| Difference in slope early discharge [NO - NW] | 0·44 | 0·36, 0·53 | **<0·0001** | 0·44 | 0·36, 0·53 | **<0·0001** | 0·44 | 0·36, 0·53 | **<0·0001** |
| Difference in change of slope between early and late discharge [MW - NW] | -0·07 | -0·15, 0·00 | 0·054 | -0·07 | -0·15, 0·00 | 0·053 | -0·07 | -0·15, 0·00 | 0·054 |
| Difference in change of slope between early and late discharge [SW - NW] | -0·26 | -0·33, -0·18 | **<0·0001** | -0·26 | -0·33, -0·19 | **<0·0001** | -0·26 | -0·33, -0·18 | **<0·0001** |
| Difference in change of slope between early and late discharge [NO - NW] | -0·27 | -0·37, -0·17 | **<0·0001** | -0·27 | -0·37, -0·17 | **<0·0001** | -0·27 | -0·37, -0·17 | **<0·0001** |
| Illness severity at admission [medium] |  |  |  | 0·1 | 0·02, 0·19 | **0·016** |  |  |  |
| Illness severity at admission [high] |  |  |  | 0·16 | 0·07, 0·25 | **0·00074** |  |  |  |
| Illness severity at discharge [medium or high] |  |  |  |  |  |  | -0·02 | -0·13, 0·08 | 0·63 |
| **Random Effects** |  |  |  |  |  |  |  |  |  |
| σ^2^ | 0·23 | | | 0·23 | | | 0·23 | | |
| τ_00_ | 0·77 record_id | | | 0·76 record_id | | | 0·77 record_id | | |
|  | 0·02 site | | | 0·02 site | | | 0·02 site | | |
| τ_11_ | 0·20 record_id·time_months | | | 0·20 record_id·time_months | | | 0·20 record_id·time_months | | |
|  | 0·22 record_id·I(pmax(time_months - 1·5, 0)) | | | 0·22 record_id·I(pmax(time_months - 1·5, 0)) | | | 0·22 record_id·I(pmax(time_months - 1·5, 0)) | | |
| ρ_01_ | -0·20 record_id·time_months | | | -0·20 record_id·time_months | | | -0·20 record_id·time_months | | |
|  | 0·09 record_id·I(pmax(time_months - 1·5, 0)) | | | 0·09 record_id·I(pmax(time_months - 1·5, 0)) | | | 0·09 record_id·I(pmax(time_months - 1·5, 0)) | | |
| ICC | 0·81 | | | 0·8 | | | 0·81 | | |
| N | 9 _site_ | | | 9 _site_ | | | 9 _site_ | | |
|  | 2461 _record_id_ | | | 2461 _record_id_ | | | 2461 _record_id_ | | |
| Observations | 9413 | | | 9413 | | | 9413 | | |
| Marginal R^2^ / Conditional R^2^ | 0·408 / 0·885 | | | 0·411 / 0·885 | | | 0·408 / 0·885 | | |
| AIC | 23228·914 | | | 23229·797 | | | 23234·783 | | |
| AICc | 23229·023 | | | 23229·925 | | | 23234·902 | | |

Table presents results for both fixed and random terms of mixed piecewise models fit using *lme4* R package. A knot point was positioned at 45-days post-discharge which defines two discharge phases (i.e., early, before 45-days and, late, after 45-days). Time was coded in months (i.e., 1·5 months corresponds to 45-days; and 3 months to 90-days). Random structure included random slopes per participant and random intercepts for site with participants nested within. Models were fit with maximum likelihood for comparison with anova using Satterthwaite's approximation of degrees of freedom as implemented in the *lmerTest* R package (Kuznetsova, A. et al., 2017). Models were evaluated using fit metrics AIC, AICc, and BIC. Restricted maximum likelihood was used to fit final models. Groups: NW, no wasting; MW, moderate wasting; SW, severe wasting; NO, nutritional oedema. WLZ, weight-for-length z-score.

### **Supplemental Table 44**. Change in WLZ in the 180-days post-discharge per nutritional group associated with exposure domains of age-inappropriate nutrition and caregiver characteristics.

|  | **Base model** | | | **Age-inappropriate nutrition** | | | **Caregiver  characteristics** | | |
| --- | --- | --- | --- | --- | --- | --- | --- | --- | --- |
| *Predictors* | *Est.* | *95% CI* | *p* | *Est.* | *95% CI* | *p* | *Est.* | *95% CI* | *p* |
| Intercept [ref, NW] | -0·28 | -0·42, -0·14 | **<0·0001** | -0·23 | -0·38, -0·09 | **0·0020** | -0·29 | -0·44, -0·14 | **0·00014** |
| Slope early discharge [NW] | 0·22 | 0·18, 0·26 | **<0·0001** | 0·22 | 0·18, 0·26 | **<0·0001** | 0·22 | 0·18, 0·26 | **<0·0001** |
| Difference in slope early vs· late discharge [NW] | -0·23 | -0·27, -0·18 | **<0·0001** | -0·23 | -0·27, -0·18 | **<0·0001** | -0·23 | -0·27, -0·18 | **<0·0001** |
| Difference in intercept early discharge [MW - NW] | -1·43 | -1·53, -1·32 | **<0·0001** | -1·37 | -1·47, -1·26 | **<0·0001** | -1·42 | -1·53, -1·32 | **<0·0001** |
| Difference in intercept early discharge [SW - NW] | -2·56 | -2·66, -2·46 | **<0·0001** | -2·44 | -2·54, -2·33 | **<0·0001** | -2·56 | -2·66, -2·46 | **<0·0001** |
| Difference in intercept early discharge [NO - NW] | -1·8 | -1·94, -1·65 | **<0·0001** | -1·68 | -1·83, -1·53 | **<0·0001** | -1·79 | -1·94, -1·65 | **<0·0001** |
| Age, months | -0·01 | -0·01, -0·00 | **0·026** | -0·01 | -0·01, -0·00 | **0·027** | -0·01 | -0·01, -0·00 | **0·028** |
| Sex, male | -0·2 | -0·27, -0·13 | **<0·0001** | -0·21 | -0·28, -0·14 | **<0·0001** | -0·2 | -0·27, -0·13 | **<0·0001** |
| Difference in slope early discharge [MW - NW] | 0·13 | 0·07, 0·20 | **<0·0001** | 0·13 | 0·07, 0·20 | **<0·0001** | 0·13 | 0·07, 0·20 | **<0·0001** |
| Difference in slope early discharge [SW - NW] | 0·37 | 0·30, 0·43 | **<0·0001** | 0·37 | 0·30, 0·43 | **<0·0001** | 0·37 | 0·30, 0·43 | **<0·0001** |
| Difference in slope early discharge [NO - NW] | 0·44 | 0·36, 0·53 | **<0·0001** | 0·44 | 0·36, 0·53 | **<0·0001** | 0·44 | 0·36, 0·53 | **<0·0001** |
| Difference in change of slope between early and late discharge [MW - NW] | -0·07 | -0·15, 0·00 | 0·054 | -0·07 | -0·15, 0·00 | 0·054 | -0·07 | -0·15, 0·00 | 0·054 |
| Difference in change of slope between early and late discharge [SW - NW] | -0·26 | -0·33, -0·18 | **<0·0001** | -0·26 | -0·33, -0·19 | **<0·0001** | -0·26 | -0·33, -0·19 | **<0·0001** |
| Difference in change of slope between early and late discharge [NO - NW] | -0·27 | -0·37, -0·17 | **<0·0001** | -0·27 | -0·37, -0·17 | **<0·0001** | -0·27 | -0·37, -0·17 | **<0·0001** |
| Age-inappropriate nutrition [medium] |  |  |  | -0·24 | -0·35, -0·12 | **0·00010** |  |  |  |
| Age-inappropriate nutrition [high] |  |  |  | -0·26 | -0·35, -0·17 | **<0·0001** |  |  |  |
| Caregiver Characteristics [Moderatly adverse] |  |  |  |  |  |  | 0·06 | -0·03, 0·15 | 0·21 |
| Caregiver Characteristics [Most adverse] |  |  |  |  |  |  | -0·02 | -0·12, 0·07 | 0·63 |
| **Random Effects** |  |  |  |  |  |  |  |  |  |
| σ^2^ | 0·23 | | | 0·23 | | | 0·23 | | |
| τ_00_ | 0·77 record_id | | | 0·75 record_id | | | 0·77 record_id | | |
|  | 0·02 site | | | 0·02 site | | | 0·02 site | | |
| τ_11_ | 0·20 record_id·time_months | | | 0·20 record_id·time_months | | | 0·20 record_id·time_months | | |
|  | 0·22 record_id·I(pmax(time_months - 1·5, 0)) | | | 0·22 record_id·I(pmax(time_months - 1·5, 0)) | | | 0·22 record_id·I(pmax(time_months - 1·5, 0)) | | |
| ρ_01_ | -0·20 record_id·time_months | | | -0·19 record_id·time_months | | | -0·20 record_id·time_months | | |
|  | 0·09 record_id·I(pmax(time_months - 1·5, 0)) | | | 0·08 record_id·I(pmax(time_months - 1·5, 0)) | | | 0·09 record_id·I(pmax(time_months - 1·5, 0)) | | |
| ICC | 0·81 | | | 0·8 | | | 0·81 | | |
| N | 9 _site_ | | | 9 _site_ | | | 9 _site_ | | |
|  | 2461 _record_id_ | | | 2461 _record_id_ | | | 2461 _record_id_ | | |
| Observations | 9413 | | | 9413 | | | 9413 | | |
| Marginal R^2^ / Conditional R^2^ | 0·408 / 0·885 | | | 0·412 / 0·885 | | | 0·407 / 0·885 | | |
| AIC | 23228·914 | | | 23204·09 | | | 23238·72 | | |
| AICc | 23229·023 | | | 23204·218 | | | 23238·851 | | |

Table presents results for both fixed and random terms of mixed piecewise models fit using *lme4* R package. A knot point was positioned at 45-days post-discharge which defines two discharge phases (i.e., early, before 45-days and, late, after 45-days). Time was coded in months (i.e., 1·5 months corresponds to 45-days; and 3 months to 90-days). Random structure included random slopes per participant and random intercepts for site with participants nested within. Models were fit with maximum likelihood for comparison with anova using Satterthwaite's approximation of degrees of freedom as implemented in the *lmerTest* R package (Kuznetsova, A. et al., 2017). Models were evaluated using fit metrics AIC, AICc, and BIC. Restricted maximum likelihood was used to fit final models. Groups: NW, no wasting; MW, moderate wasting; SW, severe wasting; NO, nutritional oedema. WLZ, weight-for-length z-score.

### **Supplemental Table 45**. Change in WLZ in the 180-days post-discharge per nutritional group associated with exposure domains of household-level exposures and access to health care.

|  | **Base model** | | | **Household-level  exposures** | | | **Access to  health care** | | |
| --- | --- | --- | --- | --- | --- | --- | --- | --- | --- |
| *Predictors* | *Est.* | *95% CI* | *p* | *Est.* | *95% CI* | *p* | *Est.* | *95% CI* | *p* |
| Intercept [ref, NW] | -0·28 | -0·42, -0·14 | **<0·0001** | -0·32 | -0·46, -0·17 | **<0·0001** | -0·29 | -0·44, -0·14 | **0·00030** |
| Slope early discharge [NW] | 0·22 | 0·18, 0·26 | **<0·0001** | 0·22 | 0·18, 0·26 | **<0·0001** | 0·22 | 0·18, 0·26 | **<0·0001** |
| Difference in slope early vs· late discharge [NW] | -0·23 | -0·27, -0·18 | **<0·0001** | -0·23 | -0·27, -0·18 | **<0·0001** | -0·23 | -0·27, -0·18 | **<0·0001** |
| Difference in intercept early discharge [MW - NW] | -1·43 | -1·53, -1·32 | **<0·0001** | -1·43 | -1·53, -1·32 | **<0·0001** | -1·43 | -1·53, -1·32 | **<0·0001** |
| Difference in intercept early discharge [SW - NW] | -2·56 | -2·66, -2·46 | **<0·0001** | -2·56 | -2·66, -2·46 | **<0·0001** | -2·56 | -2·66, -2·46 | **<0·0001** |
| Difference in intercept early discharge [NO - NW] | -1·8 | -1·94, -1·65 | **<0·0001** | -1·81 | -1·95, -1·66 | **<0·0001** | -1·79 | -1·94, -1·65 | **<0·0001** |
| Age, months | -0·01 | -0·01, -0·00 | **0·026** | -0·01 | -0·01, -0·00 | **0·024** | -0·01 | -0·01, -0·00 | **0·025** |
| Sex, male | -0·2 | -0·27, -0·13 | **<0·0001** | -0·2 | -0·27, -0·13 | **<0·0001** | -0·2 | -0·27, -0·13 | **<0·0001** |
| Difference in slope early discharge [MW - NW] | 0·13 | 0·07, 0·20 | **<0·0001** | 0·13 | 0·07, 0·20 | **<0·0001** | 0·13 | 0·07, 0·20 | **<0·0001** |
| Difference in slope early discharge [SW - NW] | 0·37 | 0·30, 0·43 | **<0·0001** | 0·37 | 0·30, 0·43 | **<0·0001** | 0·37 | 0·30, 0·43 | **<0·0001** |
| Difference in slope early discharge [NO - NW] | 0·44 | 0·36, 0·53 | **<0·0001** | 0·44 | 0·36, 0·53 | **<0·0001** | 0·44 | 0·36, 0·53 | **<0·0001** |
| Difference in change of slope between early and late discharge [MW - NW] | -0·07 | -0·15, 0·00 | 0·054 | -0·07 | -0·15, 0·00 | 0·054 | -0·07 | -0·15, 0·00 | 0·054 |
| Difference in change of slope between early and late discharge [SW - NW] | -0·26 | -0·33, -0·18 | **<0·0001** | -0·26 | -0·33, -0·18 | **<0·0001** | -0·26 | -0·33, -0·18 | **<0·0001** |
| Difference in change of slope between early and late discharge [NO - NW] | -0·27 | -0·37, -0·17 | **<0·0001** | -0·27 | -0·37, -0·17 | **<0·0001** | -0·27 | -0·37, -0·17 | **<0·0001** |
| Household-level exposures [Moderatly adverse] |  |  |  | 0·05 | -0·04, 0·15 | 0·25 |  |  |  |
| Household-level exposures [Most adverse] |  |  |  | 0·08 | -0·03, 0·18 | 0·14 |  |  |  |
| Access to health care [Moderatly adverse] |  |  |  |  |  |  | 0·02 | -0·07, 0·11 | 0·65 |
| Access to health care [Least adverse] |  |  |  |  |  |  | 0·02 | -0·08, 0·12 | 0·72 |
|  |  |  |  |  |  |  |  |  |  |
| **Random Effects** |  |  |  |  |  |  |  |  |  |
| σ^2^ | 0·23 | | | 0·23 | | | 0·23 | | |
| τ_00_ | 0·77 record_id | | | 0·77 record_id | | | 0·77 record_id | | |
|  | 0·02 site | | | 0·02 site | | | 0·02 site | | |
| τ_11_ | 0·20 record_id·time_months | | | 0·20 record_id·time_months | | | 0·20 record_id·time_months | | |
|  | 0·22 record_id·I(pmax(time_months - 1·5, 0)) | | | 0·22 record_id·I(pmax(time_months - 1·5, 0)) | | | 0·22 record_id·I(pmax(time_months - 1·5, 0)) | | |
| ρ_01_ | -0·20 record_id·time_months | | | -0·20 record_id·time_months | | | -0·20 record_id·time_months | | |
|  | 0·09 record_id·I(pmax(time_months - 1·5, 0)) | | | 0·09 record_id·I(pmax(time_months - 1·5, 0)) | | | 0·09 record_id·I(pmax(time_months - 1·5, 0)) | | |
| ICC | 0·81 | | | 0·81 | | | 0·81 | | |
| N | 9 _site_ | | | 9 _site_ | | | 9 _site_ | | |
|  | 2461 _record_id_ | | | 2461 _record_id_ | | | 2461 _record_id_ | | |
| Observations | 9413 | | | 9413 | | | 9413 | | |
| Marginal R^2^ / Conditional R^2^ | 0·408 / 0·885 | | | 0·409 / 0·885 | | | 0·408 / 0·885 | | |
| AIC | 23228·914 | | | 23239·459 | | | 23241·577 | | |
| AICc | 23229·023 | | | 23239·587 | | | 23241·705 | | |

Table presents results for both fixed and random terms of mixed piecewise models fit using *lme4* R package. A knot point was positioned at 45-days post-discharge which defines two discharge phases (i.e., early, before 45-days and, late, after 45-days). Time was coded in months (i.e., 1·5 months corresponds to 45-days; and 3 months to 90-days). Random structure included random slopes per participant and random intercepts for site with participants nested within. Models were fit with maximum likelihood for comparison with anova using Satterthwaite's approximation of degrees of freedom as implemented in the *lmerTest* R package (Kuznetsova, A. et al., 2017). Models were evaluated using fit metrics AIC, AICc, and BIC. Restricted maximum likelihood was used to fit final models. Groups: NW, no wasting; MW, moderate wasting; SW, severe wasting; NO, nutritional oedema. WLZ, weight-for-length z-score.

**Supplemental Table 46**. Change in WLZ in the 180-days post-discharge per nutritional group associated with HIV and small birth size.

|  | **Base model** | | | **HIV status** | | | **Birth size** | | |
| --- | --- | --- | --- | --- | --- | --- | --- | --- | --- |
| *Predictors* | *Est.* | *95% CI* | *p* | *Est.* | *95% CI* | *p* | *Est.* | *95% CI* | *p* |
| Intercept [ref, NW] | -0·28 | -0·42, -0·14 | **<0·0001** | -0·28 | -0·42, -0·14 | **<0·0001** | -0·27 | -0·41, -0·13 | **0.00016** |
| Slope early discharge [NW] | 0·22 | 0·18, 0·26 | **<0·0001** | 0·22 | 0·18, 0·26 | **<0·0001** | 0·22 | 0·18, 0·26 | **<0·0001** |
| Difference in slope early vs· late discharge [NW] | -0·23 | -0·27, -0·18 | **<0·0001** | -0·23 | -0·27, -0·18 | **<0·0001** | -0·23 | -0·27, -0·18 | **<0·0001** |
| Difference in intercept early discharge [MW - NW] | -1·43 | -1·53, -1·32 | **<0·0001** | -1·43 | -1·53, -1·32 | **<0·0001** | -1·42 | -1·53, -1·32 | **<0·0001** |
| Difference in intercept early discharge [SW - NW] | -2·56 | -2·66, -2·46 | **<0·0001** | -2·56 | -2·66, -2·46 | **<0·0001** | -2·56 | -2·66, -2·46 | **<0·0001** |
| Difference in intercept early discharge [NO - NW] | -1·8 | -1·94, -1·65 | **<0·0001** | -1·8 | -1·94, -1·65 | **<0·0001** | -1·79 | -1·94, -1·65 | **<0·0001** |
| Age, months | -0·01 | -0·01, -0·00 | **0·026** | -0·01 | -0·01, -0·00 | **0·026** | -0·01 | -0·01, -0·00 | **0·023** |
| Sex, male | -0·2 | -0·27, -0·13 | **<0·0001** | -0·2 | -0·27, -0·13 | **<0·0001** | -0·2 | -0·27, -0·13 | **<0·0001** |
| Difference in slope early discharge [MW - NW] | 0·13 | 0·07, 0·20 | **<0·0001** | 0·13 | 0·07, 0·20 | **<0·0001** | 0·13 | 0·07, 0·20 | **<0·0001** |
| Difference in slope early discharge [SW - NW] | 0·37 | 0·30, 0·43 | **<0·0001** | 0·37 | 0·30, 0·43 | **<0·0001** | 0·37 | 0·30, 0·43 | **<0·0001** |
| Difference in slope early discharge [NO - NW] | 0·44 | 0·36, 0·53 | **<0·0001** | 0·44 | 0·36, 0·53 | **<0·0001** | 0·44 | 0·36, 0·53 | **<0·0001** |
| Difference in change of slope between early and late discharge [MW - NW] | -0·07 | -0·15, 0·00 | 0·054 | -0·07 | -0·15, 0·00 | 0·054 | -0·07 | -0·15, 0·00 | 0·054 |
| Difference in change of slope between early and late discharge [SW - NW] | -0·26 | -0·33, -0·18 | **<0·0001** | -0·26 | -0·33, -0·18 | **<0·0001** | -0·26 | -0·33, -0·18 | **<0·0001** |
| Difference in change of slope between early and late discharge [NO - NW] | -0·27 | -0·37, -0·17 | **<0·0001** | -0·27 | -0·37, -0·17 | **<0·0001** | -0·27 | -0·37, -0·17 | **<0·0001** |
| HIV exposed |  |  |  | 0·06 | -0·08, 0·21 | 0·40 |  |  |  |
| HIV infected |  |  |  | -0·02 | -0·22, 0·18 | 0·84 |  |  |  |
| Small birth size |  |  |  |  |  |  | -0·05 | -0·14, 0·05 | 0·31 |
| **Random Effects** |  |  |  |  |  |  |  |  |  |
| σ^2^ | 0·23 | | | 0·23 | | | 0·23 | | |
| τ_00_ | 0·77 record_id | | | 0·77 record_id | | | 0·77 record_id | | |
|  | 0·02 site | | | 0·02 site | | | 0·02 site | | |
| τ_11_ | 0·20 record_id·time_months | | | 0·20 record_id·time_months | | | 0·20 record_id·time_months | | |
|  | 0·22 record_id·I(pmax(time_months - 1·5, 0)) | | | 0·22 record_id·I(pmax(time_months - 1·5, 0)) | | | 0·22 record_id·I(pmax(time_months - 1·5, 0)) | | |
| ρ_01_ | -0·20 record_id·time_months | | | -0·20 record_id·time_months | | | -0·20 record_id·time_months | | |
|  | 0·09 record_id·I(pmax(time_months - 1·5, 0)) | | | 0·09 record_id·I(pmax(time_months - 1·5, 0)) | | | 0·09 record_id·I(pmax(time_months - 1·5, 0)) | | |
| ICC | 0·81 | | | 0·81 | | | 0·81 | | |
| N | 9 _site_ | | | 9 _site_ | | | 9 _site_ | | |
|  | 2461 _record_id_ | | | 2461 _record_id_ | | | 2461 _record_id_ | | |
| Observations | 9413 | | | 9413 | | | 9413 | | |
| Marginal R^2^ / Conditional R^2^ | 0·408 / 0·885 | | | 0·408 / 0·885 | | | 0·408 / 0·885 | | |
| AIC | 23228·914 | | | 23238·196 | | | 23234·154 | | |
| AICc | 23229.023 | | | 23238.324 | | | 23234.271 | | |

Table presents results for both fixed and random terms of mixed piecewise models fit using *lme4* R package. A knot point was positioned at 45-days post-discharge which defines two discharge phases (i.e., early, before 45-days and, late, after 45-days). Time was coded in months (i.e., 1·5 months corresponds to 45-days; and 3 months to 90-days). Random structure included random slopes per participant and random intercepts for site with participants nested within. Models were fit with maximum likelihood for comparison with anova using Satterthwaite's approximation of degrees of freedom as implemented in the *lmerTest* R package (Kuznetsova, A. et al., 2017). Models were evaluated using fit metrics AIC, AICc, and BIC. Restricted maximum likelihood was used to fit final models. Groups: NW, no wasting; MW, moderate wasting; SW, severe wasting; NO, nutritional oedema. WLZ, weight-for-length z-score.

### **Supplemental Table 47**. Change in WLZ in the 180-days post-discharge per nutritional group associated with chronic medical conditions and prior hospitalisation.

|  | **Base model** | | | **Chronic medical condition** | | | **Prior hospitalisation** | | |
| --- | --- | --- | --- | --- | --- | --- | --- | --- | --- |
| *Predictors* | *Est.* | *95% CI* | *p* | *Est.* | *95% CI* | *p* | *Est.* | *95% CI* | *p* |
| Intercept [ref, NW] | -0·28 | -0·42, -0·14 | **<0·0001** | -0·27 | -0·41, -0·13 | **0.00012** | -0·28 | -0·42, -0·14 | **0.00011** |
| Slope early discharge [NW] | 0·22 | 0·18, 0·26 | **<0·0001** | 0·22 | 0·18, 0·26 | **<0·0001** | 0·22 | 0·18, 0·26 | **<0·0001** |
| Difference in slope early vs· late discharge [NW] | -0·23 | -0·27, -0·18 | **<0·0001** | -0·23 | -0·27, -0·18 | **<0·0001** | -0·23 | -0·27, -0·18 | **<0·0001** |
| Difference in intercept early discharge [MW - NW] | -1·43 | -1·53, -1·32 | **<0·0001** | -1·42 | -1·53, -1·32 | **<0·0001** | -1·43 | -1·53, -1·32 | **<0·0001** |
| Difference in intercept early discharge [SW - NW] | -2·56 | -2·66, -2·46 | **<0·0001** | -2·55 | -2·65, -2·46 | **<0·0001** | -2·56 | -2·66, -2·46 | **<0·0001** |
| Difference in intercept early discharge [NO - NW] | -1·8 | -1·94, -1·65 | **<0·0001** | -1·79 | -1·94, -1·65 | **<0·0001** | -1·8 | -1·94, -1·65 | **<0·0001** |
| Age, months | -0·01 | -0·01, -0·00 | **0·026** | -0·01 | -0·01, -0·00 | **0·031** | -0·01 | -0·01, -0·00 | **0·028** |
| Sex, male | -0·2 | -0·27, -0·13 | **<0·0001** | -0·2 | -0·27, -0·13 | **<0·0001** | -0·2 | -0·27, -0·13 | **<0·0001** |
| Difference in slope early discharge [MW - NW] | 0·13 | 0·07, 0·20 | **<0·0001** | 0·13 | 0·07, 0·20 | **<0·0001** | 0·13 | 0·07, 0·20 | **<0·0001** |
| Difference in slope early discharge [SW - NW] | 0·37 | 0·30, 0·43 | **<0·0001** | 0·37 | 0·30, 0·43 | **<0·0001** | 0·37 | 0·30, 0·43 | **<0·0001** |
| Difference in slope early discharge [NO - NW] | 0·44 | 0·36, 0·53 | **<0·0001** | 0·44 | 0·36, 0·53 | **<0·0001** | 0·44 | 0·36, 0·53 | **<0·0001** |
| Difference in change of slope between early and late discharge [MW - NW] | -0·07 | -0·15, 0·00 | 0·054 | -0·07 | -0·15, 0·00 | 0·054 | -0·07 | -0·15, 0·00 | 0·054 |
| Difference in change of slope between early and late discharge [SW - NW] | -0·26 | -0·33, -0·18 | **<0·0001** | -0·26 | -0·33, -0·19 | **<0·0001** | -0·26 | -0·33, -0·18 | **<0·0001** |
| Difference in change of slope between early and late discharge [NO - NW] | -0·27 | -0·37, -0·17 | **<0·0001** | -0·27 | -0·37, -0·17 | **<0·0001** | -0·27 | -0·37, -0·17 | **<0·0001** |
| Chronic medical condition |  |  |  | -0·16 | -0·31, -0·02 | 0·025 |  |  |  |
| Prior hospitalisation |  |  |  |  |  |  | -0·01 | -0·09, 0·07 | 0·76 |
| **Random Effects** |  |  |  |  |  |  |  |  |  |
| σ^2^ | 0·23 | | | 0·23 | | | 0·23 | | |
| τ_00_ | 0·77 record_id | | | 0·77 record_id | | | 0·77 record_id | | |
|  | 0·02 site | | | 0·02 site | | | 0·02 site | | |
| τ_11_ | 0·20 record_id·time_months | | | 0·20 record_id·time_months | | | 0·20 record_id·time_months | | |
|  | 0·22 record_id·I(pmax(time_months - 1·5, 0)) | | | 0·22 record_id·I(pmax(time_months - 1·5, 0)) | | | 0·22 record_id·I(pmax(time_months - 1·5, 0)) | | |
| ρ_01_ | -0·20 record_id·time_months | | | -0·20 record_id·time_months | | | -0·20 record_id·time_months | | |
|  | 0·09 record_id·I(pmax(time_months - 1·5, 0)) | | | 0·09 record_id·I(pmax(time_months - 1·5, 0)) | | | 0·09 record_id·I(pmax(time_months - 1·5, 0)) | | |
| ICC | 0·81 | | | 0·81 | | | 0·81 | | |
| N | 9 _site_ | | | 9 _site_ | | | 9 _site_ | | |
|  | 2461 _record_id_ | | | 2461 _record_id_ | | | 2461 _record_id_ | | |
| Observations | 9413 | | | 9413 | | | 9413 | | |
| Marginal R^2^ / Conditional R^2^ | 0·408 / 0·885 | | | 0·408 / 0·885 | | | 0·408 / 0·885 | | |
| AIC | 23228·914 | | | 23229·331 | | | 23235·358 | | |
| AICc | 23229.023 | | | 23229.449 | | | 23235.471 | | |

Table presents results for both fixed and random terms of mixed piecewise models fit using *lme4* R package. A knot point was positioned at 45-days post-discharge which defines two discharge phases (i.e., early, before 45-days and, late, after 45-days). Time was coded in months (i.e., 1·5 months corresponds to 45-days; and 3 months to 90-days). Random structure included random slopes per participant and random intercepts for site with participants nested within. Models were fit with maximum likelihood for comparison with anova using Satterthwaite's approximation of degrees of freedom as implemented in the *lmerTest* R package (Kuznetsova, A. et al., 2017). Models were evaluated using fit metrics AIC, AICc, and BIC. Restricted maximum likelihood was used to fit final models. Groups: NW, no wasting; MW, moderate wasting; SW, severe wasting; NO, nutritional oedema. WLZ, weight-for-length z-score.

**Supplemental Table 48**. Final multivariable models presenting differences in WAZ, LAZ, MUACZ and WLZ in the 180-days post-discharge per nutritional group associated with variables selected as showing an association with growth.

|  | **LAZ** | | | **WAZ** | | |
| --- | --- | --- | --- | --- | --- | --- |
| *Predictors* | *Est.* | *95% CI* | *p* | *Est.* | *95% CI* | *p* |
| Intercept [ref, NW] | -0·44 | -0·77, -0·10 | **0·011** | -0·86 | -1·08, -0·63 | **<0·00001** |
| Slope early discharge [NW] | -0·13 | -0·15, -0·11 | **<0·00001** | 0·12 | 0·09, 0·15 | **<0·00001** |
| Difference in slope early vs· late discharge [NW] | 0·11 | 0·09, 0·13 | **<0·00001** | -0·12 | -0·15, -0·09 | **<0·00001** |
| Difference in intercept early discharge [MW - NW] | -0·54 | -0·69, -0·40 | **<0·00001** | -1·22 | -1·34, -1·11 | **<0·00001** |
| Difference in intercept early discharge [SW - NW] | -1·36 | -1·50, -1·21 | **<0·00001** | -2·36 | -2·48, -2·24 | **<0·00001** |
| Difference in intercept early discharge [NO - NW] | -1·36 | -1·57, -1·15 | **<0·00001** | -1·86 | -2·03, -1·69 | **<0·00001** |
| Age-inappropriate nutrition [medium] | -0·07 | -0·24, 0·09 | 0·38 | -0·19 | -0·33, -0·05 | **0·0088** |
| Age-inappropriate nutrition [high] | -0·33 | -0·46, -0·20 | **<0·00001** | -0·4 | -0·51, -0·29 | **<0·00001** |
| Caregiver Characteristics [Moderatly adverse] | -0·19 | -0·32, -0·06 | **0·0037** | -0·1 | -0·20, 0·01 | 0·073 |
| Caregiver Characteristics [Most adverse] | -0·2 | -0·33, -0·07 | **0·0032** | -0·16 | -0·27, -0·05 | **0·0053** |
| Household-level exposures [Moderatly adverse] | -0·25 | -0·38, -0·11 | **0·00023** |  |  |  |
| Household-level exposures [Most adverse] | -0·34 | -0·49, -0·19 | **<0·00001** |  |  |  |
| Small birth size | -0·74 | -0·87, -0·61 | **<0·00001** | -0·58 | -0·69, -0·47 | **<0·00001** |
| Anemia [Mild] | 0·0 | -0·15, 0·15 | 0·98 | -0·01 | -0·13, 0·11 | 0·88 |
| Anemia [Moderate or Severe] | -0·18 | -0·31, -0·05 | **0·0086** | -0·14 | -0·25, -0·03 | **0·012** |
| Age, months | -0·01 | -0·02, -0·00 | **0·0055** | 0·01 | -0·00, 0·01 | 0·056 |
| Sex, male | -0·31 | -0·41, -0·21 | **<0·00001** | -0·25 | -0·33, -0·17 | **<0·00001** |
| Difference in slope early discharge [MW - NW] | -0·02 | -0·05, 0·01 | 0·25 | 0·08 | 0·04, 0·13 | **0·00062** |
| Difference in slope early discharge [SW - NW] | 0·01 | -0·02, 0·04 | 0·52 | 0·23 | 0·19, 0·28 | **<0·00001** |
| Difference in slope early discharge [NO - NW] | 0·07 | 0·03, 0·11 | **0·0012** | 0·32 | 0·26, 0·38 | **<0·00001** |
| Difference in change of slope between early and late discharge [MW - NW] | 0·04 | 0·00, 0·07 | **0·047** | -0·03 | -0·08, 0·02 | 0·26 |
| Difference in change of slope between early and late discharge [SW - NW] | 0·03 | -0·01, 0·06 | 0·097 | -0·14 | -0·19, -0·09 | **<0·00001** |
| Difference in change of slope between early and late discharge [NO - NW] | -0·04 | -0·08, 0·01 | 0·13 | -0·2 | -0·27, -0·13 | **<0·00001** |
| Illness severity at admission [medium] |  |  |  | 0·13 | 0·03, 0·23 | **0·0093** |
| Illness severity at admission [high] |  |  |  | 0·06 | -0·05, 0·16 | 0·31 |
| Chronic medical condition |  |  |  | -0·2 | -0·37, -0·04 | **0·017** |
| **Random Effects** |  |  |  |  |  |  |
| σ^2^ | 0·07 | | | 0·1 | | |
| τ_00_ | 1·84 record_id | | | 1·13 record_id | | |
|  | 0·17 site | | | 0·05 site | | |
| τ_11_ | 0·04 record_id·time_months | | | 0·12 record_id·time_months | | |
|  | 0·02 record_id·I(pmax(time_months - 1·5, 0)) | | | 0·12 record_id·I(pmax(time_months - 1·5, 0)) | | |
| ρ_01_ | -0·38 record_id·time_months | | | -0·18 record_id·time_months | | |
|  | 0·17 record_id·I(pmax(time_months - 1·5, 0)) | | | 0·04 record_id·I(pmax(time_months - 1·5, 0)) | | |
| ICC | 0·96 | | | 0·93 | | |
| N | 9 _site_ | | | 9 _site_ | | |
|  | 2461 _record_id_ | | | 2461 _record_id_ | | |
| Observations | 9425 | | | 9448 | | |
| Marginal R^2^ / Conditional R^2^ | 0·249 / 0·973 | | | 0·427 / 0·958 | | |
| AIC | 16979·386 | | | 19541·227 | | |
| AICc | 16979·598 | | | 19541·451 | | |

Table presents results for both fixed and random effects of mixed piecewise models with a knot point at 45-days post-discharge which defines two discharge phases (i.e., early, before 45-days and, late, after 45-days post-discharge). Random structure accounted for clustering of sites and of repeated measures within participants. Nutritional strata represent children admitted to hospital with no wasting (NW), moderate wasting (MW), severe wasting (SW) or with nutritional oedema (NO). LAZ, length-for-age z-score; WAZ, weight-for-age z-score; MUACZ, mid-upper arm circumference z-scores; WLZ, weight-for-length z-score.

**Supplemental Table 49**. Final multivariable models presenting differences in WAZ, LAZ, MUACZ and WLZ in the 180-days post-discharge per nutritional strata associated with variables selected as showing an association with growth.

|  | **MUACZ** | | | **WLZ** | | |
| --- | --- | --- | --- | --- | --- | --- |
| *Predictors* | *Est.* | *95% CI* | *p* | *Est.* | *95% CI* | *p* |
| Intercept [ref, NW] | -0·74 | -0·91, -0·57 | **<0·00001** | -0·32 | -0·48, -0·16 | **<0·00001** |
| Slope early discharge [NW] | 0·17 | 0·13, 0·20 | **<0·00001** | 0·22 | 0·18, 0·26 | **<0·00001** |
| Difference in slope early vs· late discharge [NW] | -0·13 | -0·17, -0·09 | **<0·00001** | -0·23 | -0·27, -0·18 | **<0·00001** |
| Difference in intercept early discharge [MW - NW] | -1·29 | -1·39, -1·18 | **<0·00001** | -1·36 | -1·47, -1·26 | **<0·00001** |
| Difference in intercept early discharge [SW - NW] | -2·35 | -2·45, -2·24 | **<0·00001** | -2·43 | -2·54, -2·33 | **<0·00001** |
| Difference in intercept early discharge [NO - NW] | -1·89 | -2·04, -1·74 | **<0·00001** | -1·65 | -1·80, -1·50 | **<0·00001** |
| Age-inappropriate nutrition [medium] | -0·2 | -0·32, -0·09 | **0·00062** | -0·23 | -0·35, -0·11 | **0·00016** |
| Age-inappropriate nutrition [high] | -0·4 | -0·49, -0·31 | **<0·00001** | -0·25 | -0·35, -0·16 | **<0·00001** |
| Caregiver Characteristics [Moderatly adverse] | -0·1 | -0·19, -0·02 | **0·021** |  |  |  |
| Caregiver Characteristics [Most adverse] | -0·13 | -0·22, -0·03 | **0·0072** |  |  |  |
| Small birth size | -0·24 | -0·33, -0·15 | **<0·00001** |  |  |  |
| Anemia [Mild] | -0·02 | -0·13, 0·08 | 0·66 |  |  |  |
| Anemia [Moderate or Severe] | -0·16 | -0·25, -0·06 | **0·00091** |  |  |  |
| Age, months | 0·02 | 0·01, 0·02 | **<0·00001** | -0·01 | -0·01, -0·00 | **0·041** |
| Sex, male | -0·18 | -0·25, -0·12 | **<0·00001** | -0·21 | -0·28, -0·14 | **<0·00001** |
| Difference in slope early discharge [MW - NW] | 0·19 | 0·14, 0·25 | **<0·00001** | 0·13 | 0·07, 0·20 | **<0·00001** |
| Difference in slope early discharge [SW - NW] | 0·37 | 0·32, 0·43 | **<0·00001** | 0·37 | 0·30, 0·43 | **<0·00001** |
| Difference in slope early discharge [NO - NW] | 0·5 | 0·42, 0·57 | **<0·00001** | 0·44 | 0·36, 0·53 | **<0·00001** |
| Difference in change of slope between early and late discharge [MW - NW] | -0·15 | -0·21, -0·08 | **<0·00001** | -0·07 | -0·15, 0·00 | 0·053 |
| Difference in change of slope between early and late discharge [SW - NW] | -0·28 | -0·34, -0·22 | **<0·00001** | -0·26 | -0·33, -0·19 | **<0·00001** |
| Difference in change of slope between early and late discharge [NO - NW] | -0·37 | -0·45, -0·29 | **<0·00001** | -0·27 | -0·37, -0·17 | **<0·00001** |
| Illness severity at admission [medium] | 0·16 | 0·07, 0·24 | **0·00025** | 0·1 | 0·01, 0·18 | **0·024** |
| Illness severity at admission [high] | 0·12 | 0·03, 0·21 | **0·0083** | 0·14 | 0·05, 0·23 | **0·0020** |
| Chronic medical condition |  |  |  |  |  |  |
| **Random Effects** |  |  |  |  |  |  |
| σ^2^ | 0·14 | | | 0·23 | | |
| τ_00_ | 0·81 record_id | | | 0·75 record_id | | |
|  | 0·02 site | | | 0·03 site | | |
| τ_11_ | 0·15 record_id·time_months | | | 0·20 record_id·time_months | | |
|  | 0·17 record_id·I(pmax(time_months - 1·5, 0)) | | | 0·22 record_id·I(pmax(time_months - 1·5, 0)) | | |
| ρ_01_ | -0·18 record_id·time_months | | | -0·19 record_id·time_months | | |
|  | 0·03 record_id·I(pmax(time_months - 1·5, 0)) | | | 0·08 record_id·I(pmax(time_months - 1·5, 0)) | | |
| ICC | 0·87 | | | 0·81 | | |
| N | 9 _site_ | | | 9 _site_ | | |
|  | 2461 _record_id_ | | | 2461 _record_id_ | | |
| Observations | 9364 | | | 9413 |  |  |
| Marginal R^2^ / Conditional R^2^ | 0·460 / 0·927 | | | 0·413 / 0·886 | | |
| AIC | 20384·975 | | | 23206·67 | | |
| AICc | 20385·188 | | | 23206·82 | | |

Table presents results for both fixed and random effects of mixed piecewise models with a knot point at 45-days post-discharge which defines two discharge phases (i.e., early, before 45-days and, late, after 45-days post-discharge). Random structure accounted for clustering of sites and of repeated measures within participants. Nutritional strata represent children admitted to hospital with no wasting (NW), moderate wasting (MW), severe wasting (SW) or with nutritional oedema (NO). LAZ, length-for-age z-score; WAZ, weight-for-age z-score; MUACZ, mid-upper arm circumference z-scores; WLZ, weight-for-length z-score.

**
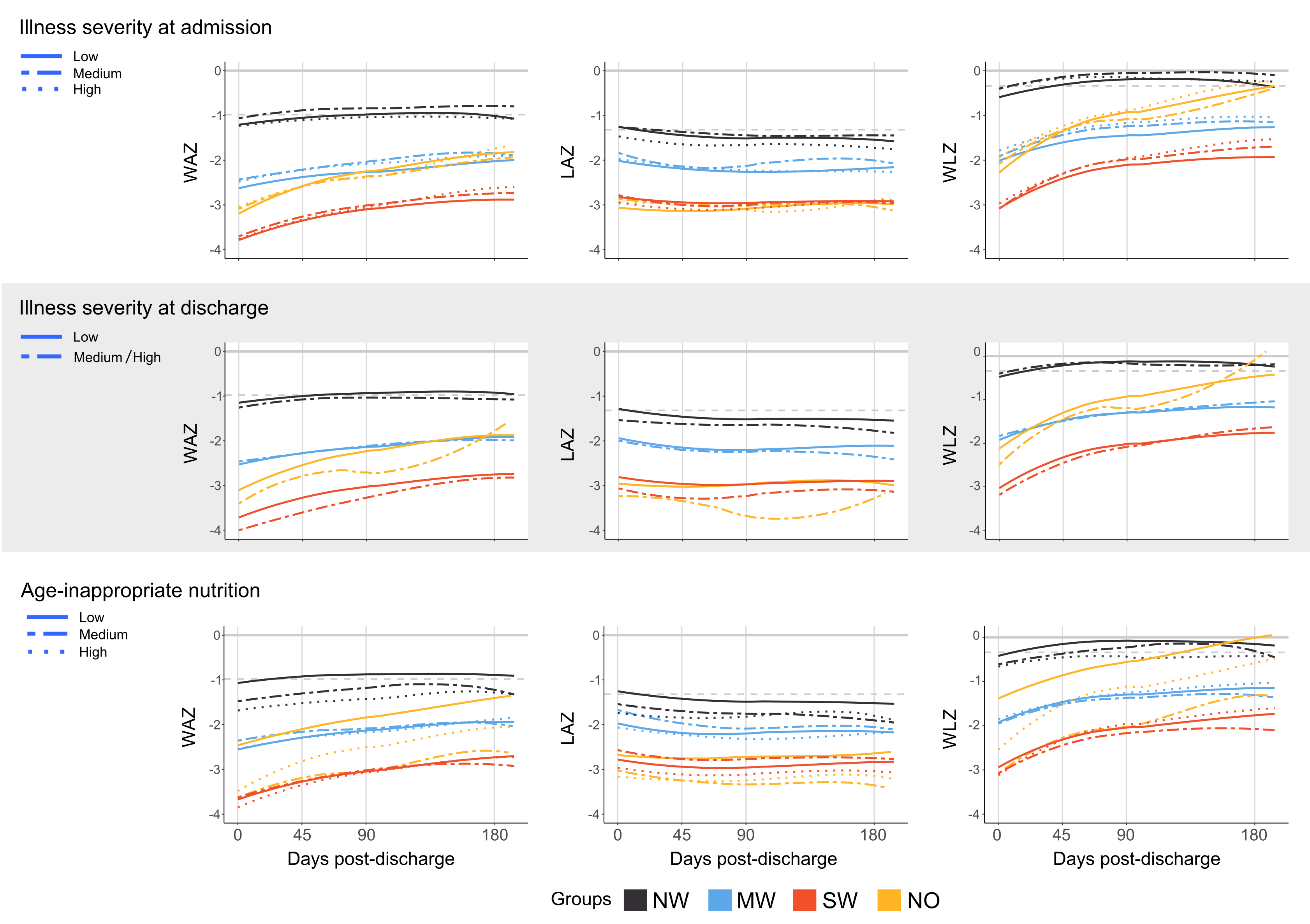
**

**Supplemental Figure 11**. Growth trajectory of children admitted to hospital with acute illness split by nutritional groups and by latent domains constructed to represent illness severity at admission, illness severity at discharge, and age-inappropriate nutrition. Group trajectories fitted by locally estimated scatterplot smoothing (LOESS) with lines colored by nutritional category as per legend at bottom, and line type (i.e., solid, long dashed or dotted) represent the domain levels as indicated by legend at left. Light gray dotted line shows average of community participants). Groups: NW, no wasting; MW, moderate wasting; SW, severe wasting; NO, nutritional oedema. LAZ, length-for-age z-score; WAZ, weight-for-age z-score; WLZ, weight-for-length z-score.

**
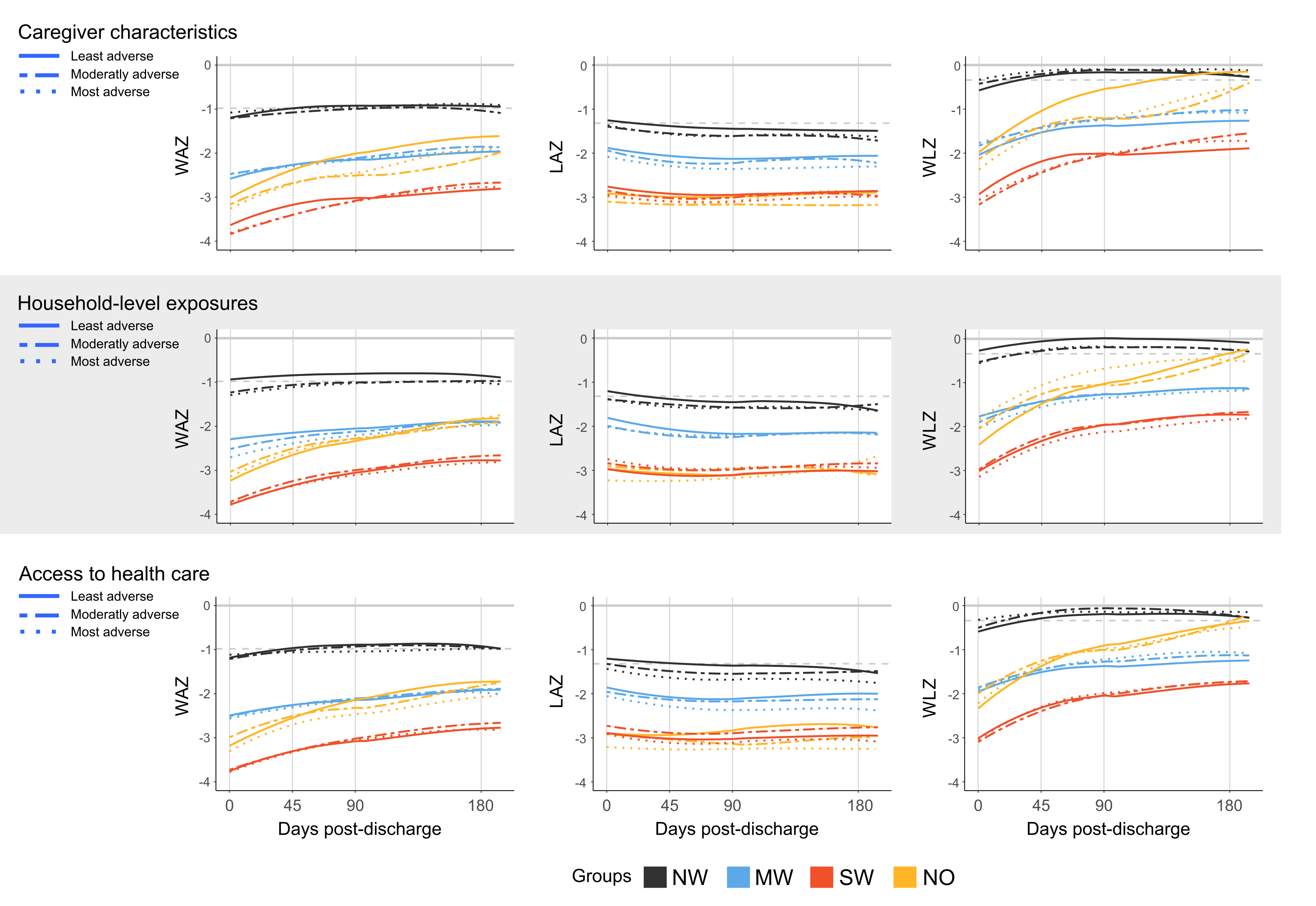
**

**Supplemental Figure 12**. Growth trajectory of children admitted to hospital with acute illness split by nutritional groups and by latent domains constructed to represent caregiver characteristics, household-level exposures, and access to health care. Group trajectories fitted by locally estimated scatterplot smoothing (LOESS) with lines colored by nutritional category as per legend at bottom, and line type (i.e., solid, long dashed or dotted) represent the domain levels as indicated by legend at left. Light gray dotted line shows average of community participants. Groups: NW, no wasting; MW, moderate wasting; SW, severe wasting; NO, nutritional oedema. LAZ, length-for-age z-score; WAZ, weight-for-age z-score; WLZ, weight-for-length z-score.

**
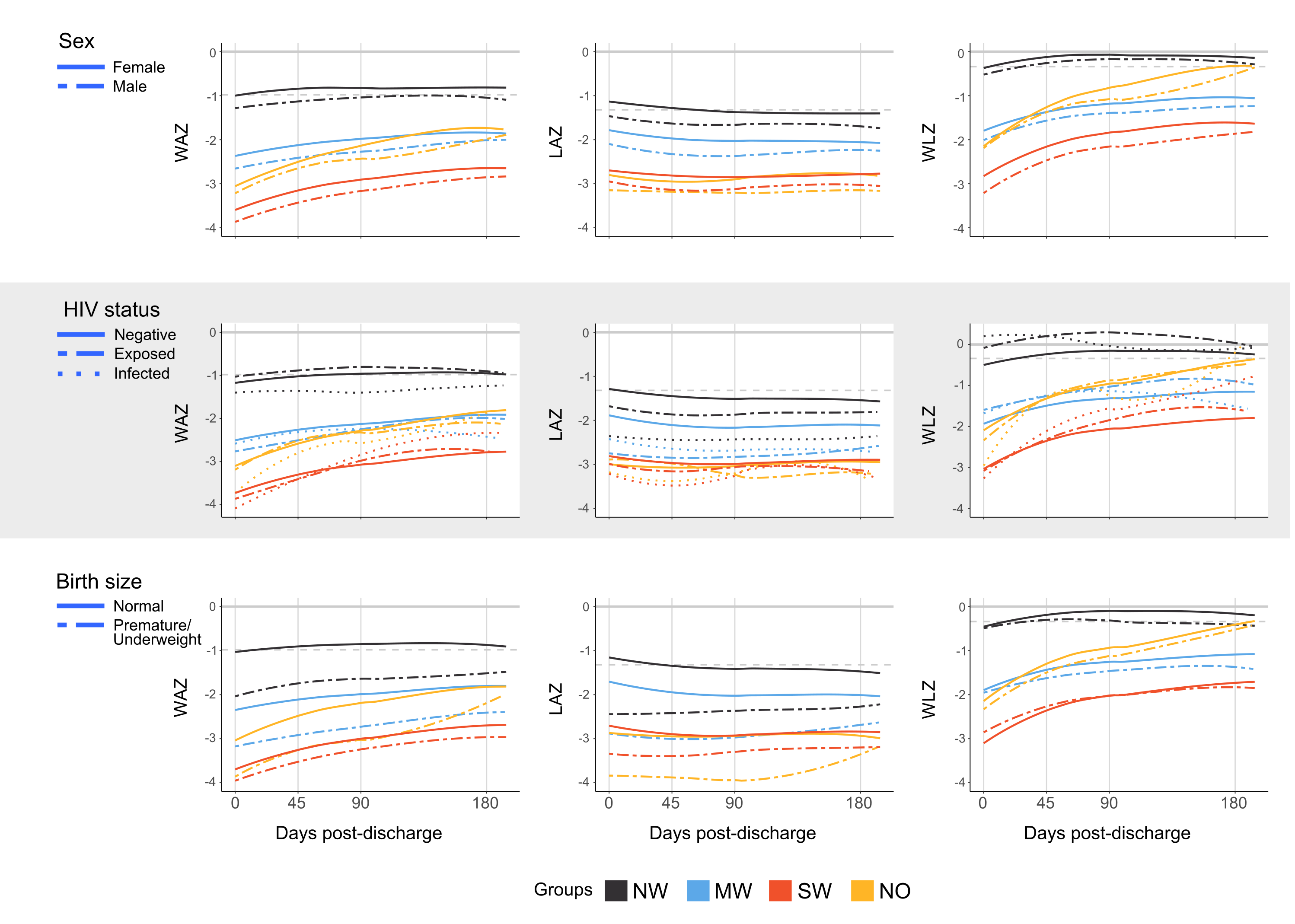
**

**Supplemental Figure 13**. Growth trajectory of children admitted to hospital with acute illness split by nutritional groups and by additional clinical variables. Variables include sex, HIV status, and birth size. Group trajectories fitted by locally estimated scatterplot smoothing (LOESS) with lines colored by nutritional category as per legend at bottom, and line type (i.e., solid, long dashed or dotted) represent the domain levels as indicated by legend at left. Light gray dotted line shows average of community participants. Groups: NW, no wasting; MW, moderate wasting; SW, severe wasting; NO, nutritional oedema. LAZ, length-for-age z-score; WAZ, weight-for-age z-score; WLZ, weight-for-length z-score.

**
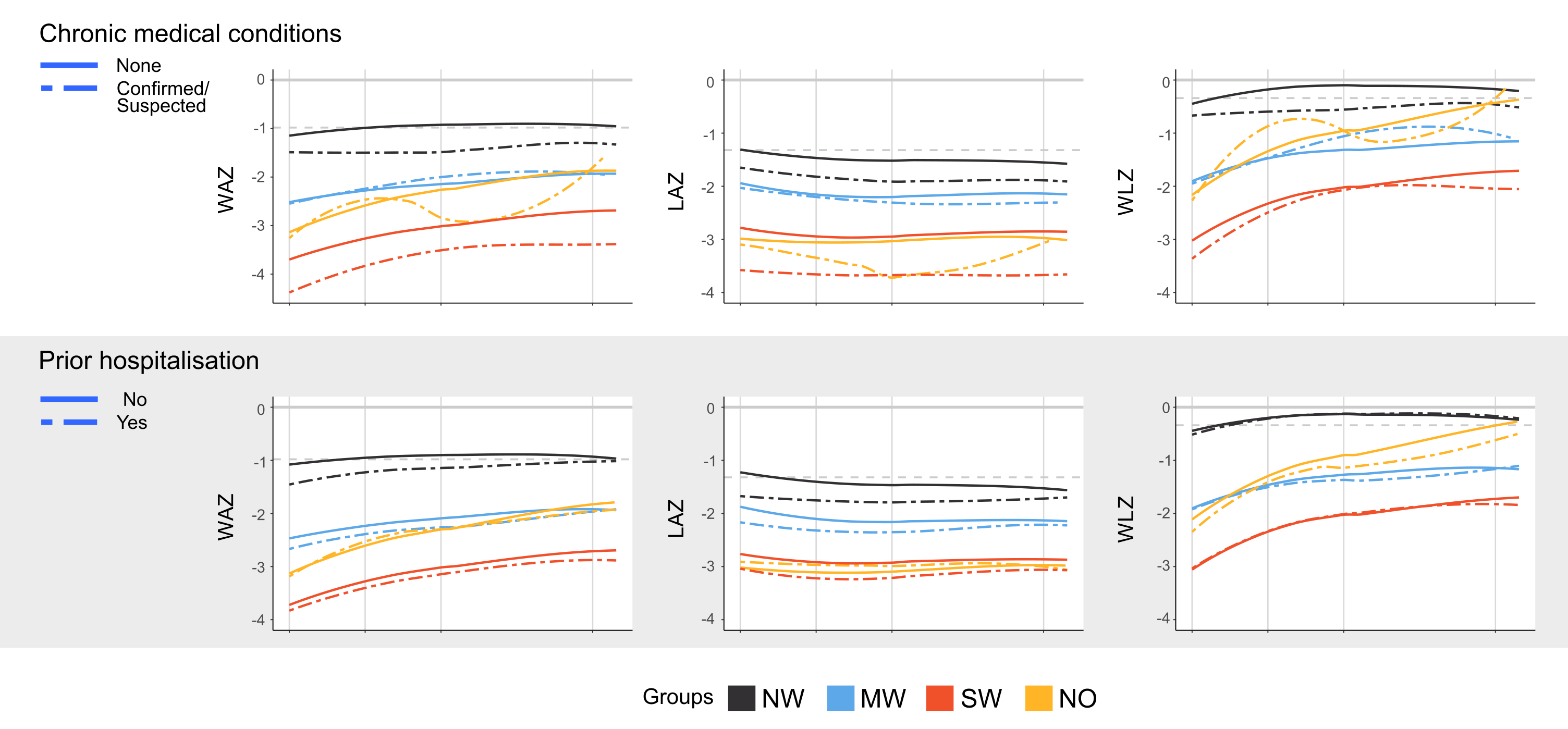
**

**Supplemental Figure 14**. Growth trajectory of children admitted to hospital with acute illness split by nutritional groups and by additional clinical variables including chronic medical conditions, and prior hospitalisation. Group trajectories fitted by locally estimated scatterplot smoothing (LOESS) with lines colored by nutritional category as per legend at bottom, and line type (i.e., solid, long dashed or dotted) represent the domain levels as indicated by legend at left. Light gray dotted line shows average of community participants. Groups: NW, no wasting; MW, moderate wasting; SW, severe wasting; NO, nutritional oedema. LAZ, length-for-age z-score; WAZ, weight-for-age z-score; WLZ, weight-for-length z-score.

**Supplemental Table 50**. Counts and percentages of children classified within each level of latent domain variables split by nutritional group.

| Illness severity at admission | | |  | Illness severity at discharge | | |
| --- | --- | --- | --- | --- | --- | --- |
|  |  | **n (%)** |  |  |  | **n (%)** |
| NW | Low | 299 (31) |  | NW | Low | 786 (82) |
|  | Medium | 323 (34) |  |  | Medium or High | 174 (18) |
|  | High | 338 (35) |  |  |  |  |
| MW | Low | 212 (37) |  | MW | Low | 482 (84) |
|  | Medium | 174 (30) |  |  | Medium or High | 90 (16) |
|  | High | 186 (33) |  |  |  |  |
| SW | Low | 273 (40) |  | SW | Low | 594 (87) |
|  | Medium | 220 (32) |  |  | Medium or High | 88 (13) |
|  | High | 189 (28) |  |  |  |  |
| NO | Low | 146 (57) |  | NO | Low | 219 (85) |
|  | Medium | 74 (29) |  |  | Medium or High | 39 (15) |
|  | High | 38 (15) |  |  |  |  |
|  |  |  |  |  |  |  |
| Age-inappropriate nutrition | | |  | Caregiver characteristics | | |
|  |  | **n (%)** |  |  |  | **n (%)** |
| NW | Low | 786 (82) |  | NW | Least adverse | 392 (41) |
|  | Medium | 64 (6·7) |  |  | Moderately adverse | 307 (32) |
|  | High | 110 (11) |  |  | Most adverse | 261 (27) |
| MW | Low | 335 (59) |  | MW | Least adverse | 259 (45) |
|  | Medium | 90 (16) |  |  | Moderately adverse | 175 (31) |
|  | High | 147 (26) |  |  | Most adverse | 138 (24) |
| SW | Low | 242 (35) |  | SW | Least adverse | 259 (38) |
|  | Medium | 101 (15) |  |  | Moderately adverse | 243 (36) |
|  | High | 339 (50) |  |  | Most adverse | 180 (26) |
| NO | Low | 89 (34) |  | NO | Least adverse | 88 (34) |
|  | Medium | 10 (3·9) |  |  | Moderately adverse | 74 (29) |
|  | High | 159 (62) |  |  | Most adverse | 96 (37) |
|  |  |  |  |  |  |  |
| Household-level exposures | | |  | Access to health care | | |
|  |  | **n (%)** |  |  |  | **n (%)** |
| NW | Least adverse | 282 (29) |  | NW | Least adverse | 277 (29) |
|  | Moderately adverse | 297 (31) |  |  | Moderately adverse | 327 (34) |
|  | Most adverse | 381 (40) |  |  | Most adverse | 356 (37) |
| MW | Least adverse | 164 (29) |  | MW | Least adverse | 211 (37) |
|  | Moderately adverse | 197 (34) |  |  | Moderately adverse | 194 (34) |
|  | Most adverse | 211 (37) |  |  | Most adverse | 167 (29) |
| SW | Least adverse | 194 (28) |  | SW | Least adverse | 251 (37) |
|  | Moderately adverse | 226 (33) |  |  | Moderately adverse | 229 (34) |
|  | Most adverse | 262 (38) |  |  | Most adverse | 202 (30) |
| NO | Least adverse | 115 (45) |  | NO | Least adverse | 85 (33) |
|  | Moderately adverse | 96 (37) |  |  | Moderately adverse | 98 (38) |
|  | Most adverse | 47 (18) |  |  | Most adverse | 75 (29) |

Domain variables are defined in Table 1. Groups: NW, no wasting; MW, moderate wasting; SW, severe wasting; NO, nutritional oedema.


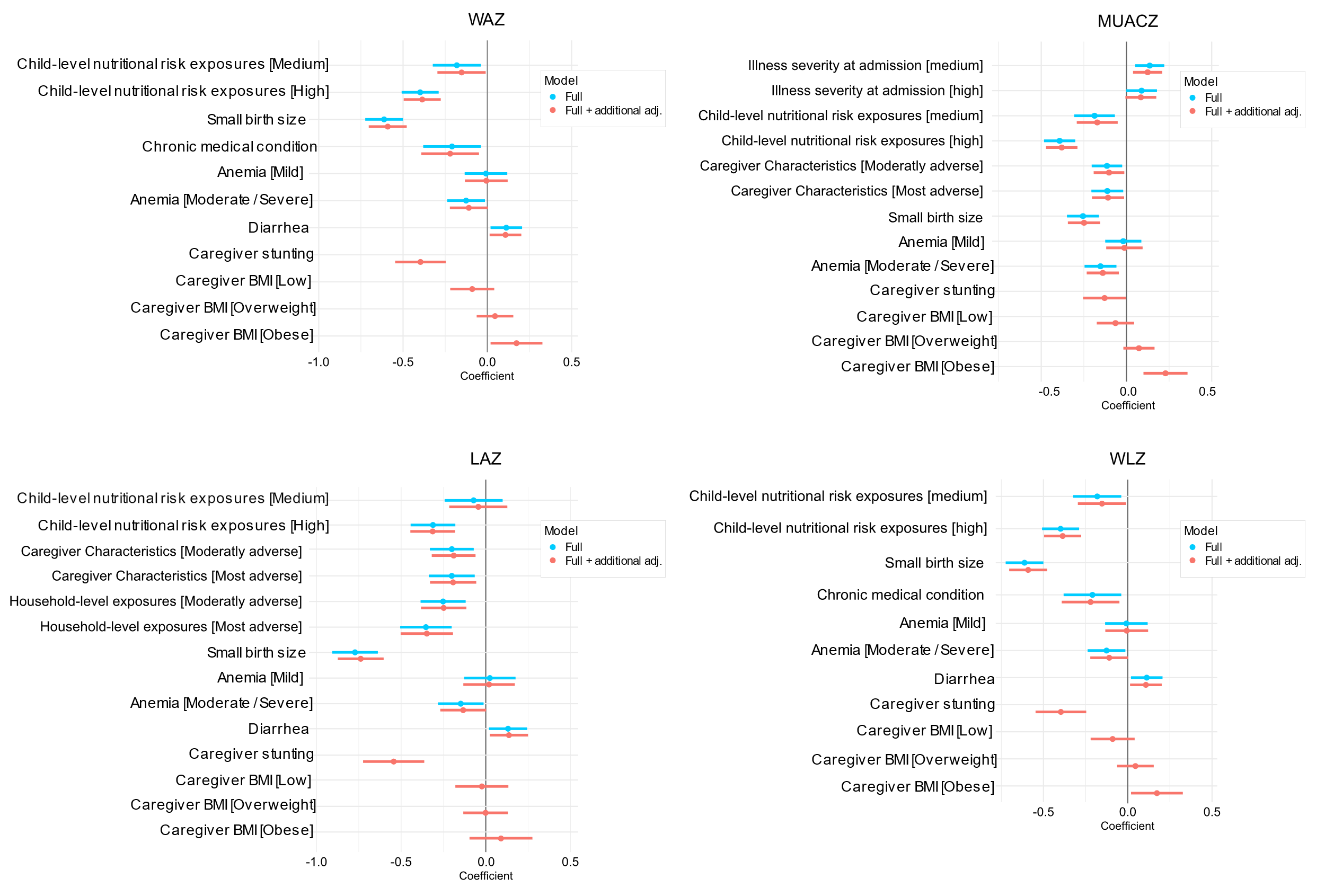


**Supplemental Figure 15**. Forest plots of coefficients present the fixed effects of the final multivariable models with or without further adjustment for caregiver stunting and caregiver BMI. Estimates were derived from mixed effects piecewise models with a knot point at 45-days post-discharge defining two discharge phases (i.e., early, before 45-days and, late, after 45-days post-discharge). Random structure accounted for clustering of sites and of repeated measures within participants. Nutritional strata represent children admitted to hospital with no wasting (NW), moderate wasting (MW), severe wasting (SW) or with nutritional oedema (NO). LAZ, length-for-age z-score; WAZ, weight-for-age z-score; MUACZ, mid-upper arm circumference z-scores; WLZ, weight-for-length z-score.
